# Nourishing Kidney Promoting Ovulation Decoction (NKPOD) Attenuates Polycystic Ovary Syndrome by Downregulating miRNA-224

**DOI:** 10.1155/2023/9402155

**Published:** 2023-04-20

**Authors:** Yinhua Guo, Xingli Tong, Peipei Tang, Wenting Zuo, Yong Tan

**Affiliations:** Department of Reproductive Medicine, Jiangsu Hospital of Traditional Chinese Medicine, Affiliated Hospital of Nanjing University of Traditional Chinese Medicine, Nanjing 210000, Jiangsu, China

## Abstract

**Background:**

Currently, exploring effective agents is urgently required for polycystic ovary syndrome (PCOS) treatment. Although nourishing kidney promoting ovulation decoction (NKPOD) as a traditional Chinese medicine decoction is widely employed to increase pregnancy rates, whether NKPOD attenuates ovulation disorders in PCOS patients remains unknown. Here, we aim to explore the clinical significance and the underlying mechanisms of NKPOD in ovulation disorders.

**Methods:**

PCOS patients were recruited to confirm the clinical significance of NKPOD in attenuating ovulation disorder. Subsequently, regulation targets of NKPOD were identified through network pharmacology analysis. Additionally, a series of experiments were performed to observe the impacts of NKPOD on miRNA-224 transcription through transcription factor AR.

**Results:**

In this study, NKPOD administration improved hormone dysregulation and reproductive outcomes in PCOS patients. Interestingly, 100 potential targets related to NKPOD and PCOS were screened, and transcription regulation was observed to be the most enriched function. Mechanistically, NKPOD inhibited miRNA-224 transcription through reducing AR expression, in which AR as a transcription factor directly regulated miRNA-224 transcription.

**Conclusions:**

Collectively, these findings highlight the therapeutic effect of NKPOD on PCOS, which could provide promising therapeutic agents for PCOS.

## 1. Introduction

Polycystic ovary syndrome (PCOS) is an endocrine disease which is characterized by anovulatory cycles/oligo, polycystic ovaries, hirsutism, and insulin resistance [1]. Accumulating evidence indicates that arrested follicular growth and abnormality of early follicle development are critical factors in the pathogenesis of PCOS [2–4]. At present, approximately 5% to 20% of females will develop PCOS during their reproductive age [2, 5]. Mounting evidence indicates that PCOS increases the risk of type 2 diabetes, cardiovascular disease, obesity, metabolic syndrome, and endometrial cancer [6–8]. Additionally, pregnant women with PCOS are more vulnerable to miscarriages and ovarian hyperstimulation syndrome [9]. Therefore, exploring effective agents is urgently required for the treatment of PCOS.

Traditional Chinese medicine (TCM) has been widely applicable to treatment for PCOS [10], and nourishing kidney promoting ovulation decoction (NKPOD), which is composed of twelve kinds of Chinese herbal medicines, is one of them, including Angelicae sinensis Radix, Paeoniae Radix alba, Rhizoma Dioscoreae, Rehmanniae Radix Preparata, Cortex Moutan, Poria, Semen Cuscutae, Dipsaci Radix, Cuscutae Semen, Cornu Cervi, Faeces Trogopterori, and Carthami flos. Modern pharmacological studies have shown that some active ingredients in NKPOD play a role in regulating oocyte competency and embryo development [10–12]. For example, animal experiments showed that Bu-shen-zhu-yun decoction including Angelicae sinensis Radix significantly alleviated pathological changes in the ovary, altered hormone levels of serum, and reduced the apoptotic rate of granulosa cells [13], while an experimental study demonstrated that Cuscuta-Salvia ameliorated the pathological changes in the liver, ovaries, and adipose tissue [14]. Multicomponents and multiple targeting characteristics of NKPOD play a common role in the protective effect of PCOS; thus, expanding the knowledge of NKPOD in PCOS treatment will be interesting.

During the development of the oocyte and embryo, numerous miRNAs are produced, and they function through posttranscriptional gene regulation [15, 16]. Several previous studies have demonstrated that miRNA-224 is a marker of PCOS and regulates oocyte competency and embryo development [17, 18]. As an example, miRNA-224 is involved in the growth of follicular granulosa cell by transforming growth factor-*β*1 and estradiol (E2) [19]. Another research of miRNA-224 revealed bisphenol-A exposure changes in serum E2 levels and follicle-stimulating hormone levels through transcriptional regulation on miRNA-224 in preantral ovarian granulosa cells [18]. These pieces of evidence suggest that miRNA-224 could be involved in later development of follicles.

In this study, we explore and present the NKPOD-treatment-mechanism relationships with clinical research, network pharmacology methods, and *in vitro* experiments. We use these methods to give a dependable result of the NKPOD on attenuating polycystic ovary syndrome. Furthermore, we investigate whether NKPOD could regulate the expression of miRNA-224 and the underlying mechanism.

## 2. Methods

### 2.1. Clinical Samples

From January 2018 to June 2020, the PCOS patients (age <38 years) who underwent *in vitro* fertilization and embryo transfer and intracytoplasmic sperm injection (IVF-ET/ICSI)-assisted pregnancy in the Reproductive Medicine Department of Jiangsu Traditional Chinese Medicine Hospital were collected. The basic clinical characteristics, embryo laboratory indexes, and the OHSS incidence of the two groups were analyzed and compared (Table 1). The diagnostic criteria for PCOS referred to as PCOS formulated by experts of the European Society of Human Reproduction and Embryology and the American Society of Reproductive Medicine (ESHRE/ASRM) in Rotterdam in 2003 were as follows: (a) clinical manifestations of hyperandrogenemia and/or hyperandrogen, (b) anovulation or rare ovulation, and (c) ultrasound showing ovarian polycystic changes (ovarian volume greater than 10 ml in one or both ovaries and/or > 12 follicles with a diameter of 2–9 mm). After excluding Cushing's syndrome, congenital adrenocortical hyperplasia, androgen-secreting tumors, and other diseases, if 2 of the above three are consistent, it can be diagnosed as polycystic ovary syndrome. Among them, hyperandrogenemia is based on the fact that the fasting testosterone value in the morning of menstruation D3 is higher than the upper limit of normal. The standard of rare ovulation was a menstrual cycle >35 days. The ultrasound image of the polycystic ovary is based on the standard that the number of bilateral ovarian sinus follicles (AFC) shown by transvaginal ultrasound is greater than 12. The selected patients with normal ovarian function were younger than 38 years and had regular menstrual cycles (28–30 d), normal basic reproductive hormones, and a number of follicles in both ovarian sinuses (unilateral AFC = 6–10). The two groups had no ovarian cysts, hyperprolactinemia, ovarian tumors, endometriosis, ovarian surgery, chemotherapy and radiotherapy, hypothyroidism/hyperthyroidism, and other endocrine diseases, and the chromosomes of the couple were normal. The Ethics Committee of the Jiangsu Hospital of Traditional Chinese Medicine (2019NL-KS74) approved this study. Written informed consent was obtained from all participants included in the study.

### 2.2. NKPOD Administration

NKPOD that met the standards set by the Chinese Pharmacopoeia (2015 Edition) [20] contained Angelicae sinensis Radix (10 g), Paeoniae Radix alba (10 g), Rhizoma Dioscoreae (10 g), Rehmanniae Radix Preparata (10 g), Cortex Moutan (10 g), Poria (10 g), Semen Cuscutae (9 g), Dipsaci Radix (10 g), Cuscutae Semen (10 g), Cornu Cervi (10 g), Faeces Trogopterori (10 g), and Carthami flos (5 g). All components were provided by Jiangsu Traditional Chinese Medicine Hospital. All the components were soaked in cold water for 30 minutes; then, NKPOD was soaked in water to a final volume of 1 L. All the components were extracted by boiling in 400 mL water for 1 h.

### 2.3. Sample Collection and Detection

For collection of serum, blood was collected on the 2nd–5th day of the menstrual cycle and the elbow vein blood of the patient was extracted. After centrifugation (2,000 × *g* for 5 min; 10,000 × *g* for 5 min) at 4°C, the serum was taken and stored in the −80°C refrigerator for testing. For collection of follicular fluid, the diameter of the selected experimental follicles was more than 18 mm. The follicular fluid was obtained when the follicles were punctured, and the eggs were taken under the guidance of vaginal B-ultrasound. About 4 ml of clear follicular fluid without blood pollution was left. After centrifugation, the supernatant was left and stored in the refrigerator at −80°C for testing. For collection of granulosa cells, the eggs were punctured according to the egg taken as the standard, the follicular fluid was retained, human lymphocyte separation solution was added, the granulosa cell layer between the two liquid levels was absorbed after centrifugation, PBS was added, the supernatant was discarded after centrifugation, and the lower layer had granulosa cells. Granulosa cells for experimental research and oocytes for IVF-ET/ICSI were collected. The granulosa cells were grown in DMEM/F12 (1 : 1; Thermo Fisher Scientific, Inc., Waltham, MA, USA) supplemented with 10% fetal bovine serum (FBS) (Thermo Fisher Scientific) and 1% antibiotics (Thermo Fisher Scientific), including penicillin (100 U/ml) and streptomycin (100 g/ml), at 37°C with 5% CO_2_. The serum levels of luteinizing hormone (LH), E2, testosterone (T), follicle-stimulating hormone (FSH), and anti-Mullerian hormone (AMH) were measured by electrochemiluminescence; additionally, clinical information about participants was collected, including stimulation duration of gonadotropin (Gn), total Gn doses, the number of oocytes punctured and oocytes retrieved, the number of 2PN and 2PN cleavage numbers, and the number of embryos transferred and high-quality embryos. The number of dominant follicles, ovulation, pregnancies, and abortions was also recorded. D3 embryo grades were as follows [21]: (a) grade I: an embryo with blastomeres of equal size; in addition, no cytoplasmic fragmentation was found; (b) grade II: an embryo with blastomeres of equal size and minor; in addition, cytoplasmic fragmentation covers ≤10% of the embryo surface; (c) grade III: an embryo with blastomeres of equal or unequal size; in addition, cytoplasmic fragmentation covers >10% of the embryo surface; (d) grade IV: an embryo with few blastomeres of any size; in addition, severe fragmentation covers ≥50% of the embryo surface; (e) grade V: an embryo with few blastomeres of any size; in addition, severe fragmentation covers ≥50% of the embryo surface; grades I and II are called high-quality embryos, and grades I ∼ III are available embryos.

### 2.4. Plasmid Construction and Transfection

The full-length cDNA of AR was cloned into pcDNA3.1 (Thermo Fisher Scientific, Inc., Waltham, MA, USA). The corresponding empty vector (pcDNA3.1-NC) and AR plasmid (pcDNA3.1-AR) were transfected into granulosa cells using Lipofectamine 3000 reagent (Thermo Fisher Scientific). G418 treatment (2 mg/ml) was performed to structure stably transfected cells. Subsequently, granulosa cells were subjected to qRT-PCR analysis to evaluate AR expression.

### 2.5. Pharmacological Network Analysis

The components potential targets of Angelicae sinensis Radix, Paeoniae Radix alba, Rhizoma Dioscoreae, Rehmanniae Radix Preparata, Cortex Moutan, Poria, Semen Cuscutae, Dipsaci Radix, Cuscutae Semen, Cornu Cervi, Faeces Trogopterori, and Carthami flos were identified by the Traditional Chinese Medicine Systems Pharmacology Database and Analysis Platform (TCMSP, https://tcmsp-e.com/) with drug likeness (DL ≥ 0.1) and oral bioavailability (OB ≥ 30%). The potential targets of Angelicae sinensis Radix, Paeoniae Radix Alba, Rhizoma Dioscoreae, Rehmanniae Radix Preparata, Cortex Moutan, Poria, Semen Cuscutae, Dipsaci Radix, Cuscutae Semen, Cornu Cervi, Faeces Trogopterori, and Carthami flos were also obtained from the TCMSP (https://tcmsp-e.com/). Subsequently, PCOS-related genes were obtained from the GeneCards database (https://www.genecards.org/) and the Comparative Toxicogenomics Database (CTD, https://ctdbase.org/). Using Cytoscape software (version 3.8.2, Bethesda, MD, USA), we visualized the composition-target network. In addition, protein-protein interactions (confidence score >0.9) were selected using STRING (https://www.string-db.org/) and Cytoscape software. The core targets were analyzed using plug-in components (CytoNCA) through analysing betweenness, bloseness, degrees, eigenvectors, LAC, and networks. The visualization of gene ontology (GO) terms was performed by using the clusterProfiler package in R.

### 2.6. Bioinformatic Analysis

The transcription factors for miRNA-224 and predicted sites of AR-binding in miRNA-224 promoters were acquired through TransmiRNA v2.0 (https://www.cuilab.cn/transmiRNA) and hTFtarget (https://bioinfo.life.hust.edu.cn/hTFtarget#!/). The DNA motif of AR was predicted from JASPAR (https://jaspar.genereg.net/).

### 2.7. Western Blot Assay

Total proteins from granulosa cells were extracted and separated by SDSPAGE gels (10%) and then transferred to PVDF membranes (0.22 *μ*m, Merck Millipore, Billerica, Massachusetts, USA). After blocking with skim milk powder (5%), the membranes incubated with the androgen receptor (AR) antibody (NO. ab108341, Abcam, Cambridge, UK), p-AR antibody (NO. ab45089, Abcam), or glyceraldehyde-3-phosphate dehydrogenase (GAPDH) antibody (NO. ab181602, Abcam) at 4°C overnight. Next, secondary antibodies (NO. ab205718, ab205719, Abcam) were incubated. An ECL detection system (Bio-Rad, Hercules, USA) was used to measure the protein bands. GAPDH was used as a control.

### 2.8. Real-Time Quantitative PCR (RT-qPCR)

Total RNA was extracted from granulosa cells using Magnetic Tissue/Cell/Blood Total RNA Kit (Tiangen Biochemical Technology Co., Ltd, Beijing, China). Then, RNA was reverse-transcribed using QuantScript RT Kit (Tiangen) by following the standard protocols. Real-time PCR was carried out using ReliaTM SYBR QPCR Mix (Nanjing Detai Bioengineering Co., Ltd, Nanjing, China). The level of miRNA-224 was normalized to U6, and the relative level was calculated. U6 primers were as follows: forward: 5′-GCT TCG GCA GCA CAT ATA CTA A-3′ and reverse: 5′-AAC GCT TCA CGA ATT TGC GT-3′. miRNA-224 primers were as follows: forward: 5′-TCA AGT CAC TAG TGG TTC CGT -3′ and reverse: 5′-GGC TTT GTA GTC ACT AGG GCA-3′. miRNA-224-3p primers were as follows: forward: 5′- TGA TGT GGG TGC TGG TGT C -3′ and reverse: 5′- TTG TGT TGG GGC AGT ACT G -3′. miRNA-224-5p primers were as follows: forward: 5′- CTG GTA GGT AAG TCA CTA -3′ and reverse: 5′- TCA ACT GGT GTC GTG GAG -3′.

### 2.9. Dual‐Luciferase Assay

The wild-type reporter of miRNA-224 promoters (pmiRNAGLO-WT) or the mutant reporter (pmiRNAGLO- MUT) was cotransfected with pcDNA3.1-NC or pcDNA3.1-AR into HEK293T cells. After transfection for 24 h, HEK293T cells were washed with PBS and subjected to the dual‐luciferase reporter assay system (Promega) for luciferase level measurement. Luciferase activities were normalized to that of Renilla.

### 2.10. Chromatin Immunoprecipitation (ChIP)

By following the manufacturer's instructions, ChIP assays were conducted using ChIP Assay Kit (Beyotime, Haimen, China). Granulosa cells were crosslinked with formaldehyde and sonicated to an average length of 200–1000 bp. Subsequently, IP was performed with an AR antibody (Abcam) or IgG. Finally, precipitated DNA was detected by qRT-PCR.

### 2.11. Statistical Analysis

Quantitative data are presented as the mean ± SD and analyzed by using SPSS software (version 22.0, IBM, Chicago, USA). Normality of distribution was assessed using the Kolmogorov–Smirnov test. The Mann–Whitney *U* tests or Student's *t*-test was performed for two-group comparisons. Differences between the two groups after treatment were measured using covariance analysis. The threshold for statistical significance was set at *P* < 0.05.

## 3. Results

### 3.1. NKPOD Attenuates Ovulation Disorders in PCOS Patients

**Figure 1:**
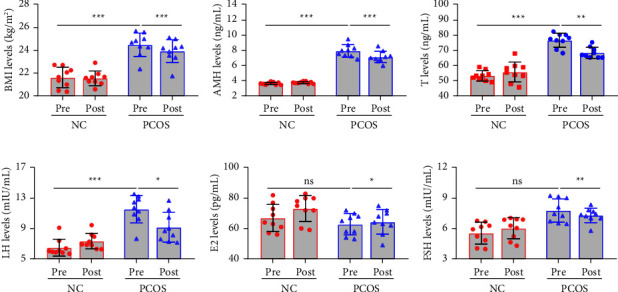
NKPOD administration alleviates hormone dysregulation in PCOS patients. (a) The changes in the BMI of PCOS patients receiving NKPOD administration. (b) The changes in anti-Müllerian hormone (AMH) of PCOS patients with NKPOD treatment. (c) After NKPOD treatment, the levels of testosterone (T) in PCOS patients. (d) The changes in luteinizing hormone (LH) of PCOS patients receiving NKPOD administration. (e) The changes in estradiol (E2) of PCOS patients receiving NKPOD administration. (f) The changes in follicle-stimulating hormone (FSH) in PCOS patients receiving NKPOD administration. ns represents (*P* > 0.05), ^*∗*^(*P* < 0.05), ^*∗∗*^(*P* < 0.01), and ^*∗∗∗*^(*P* < 0.001).

In this study, we investigated the function of NKPOD in regulating ovulation disorders in PCOS patients. Interestingly, NKPOD administration resulted in a significant decrease of BIM in PCOS patients (*P* < 0.001; Figure 1(a)). PCOS patients displayed higher levels of AMH, while the increase was partly reversed by NKPOD administration (*P* < 0.001; Figure 1(b)). The influence of NKPOD on T levels in PCOS patients was verified; the results demonstrated that NKPOD administration significantly impaired PCOS-mediated promotion of serum T levels (*P* < 0.01; Figure 1(c)). Similar results were observed in LH levels (*P* < 0.05; Figure 1(d)). To further explore the effects of NKPOD on attenuating ovulation disorders, we confirmed the serum of E2. Interestingly, higher E2 levels were observed in PCOS patients receiving NKPOD administration (*P* < 0.05; Figure 1(e)). Moreover, we found that the increased FSH levels in PCOS patients were partially reversed by NKPOD (*P* < 0.01; Figure 1(f)). Collectively, these data demonstrate that NKPOD exerts a therapeutic effect on PCOS.

### 3.2. NKPOD Improves Reproductive Outcomes in PCOS Patients

**Figure 2:**
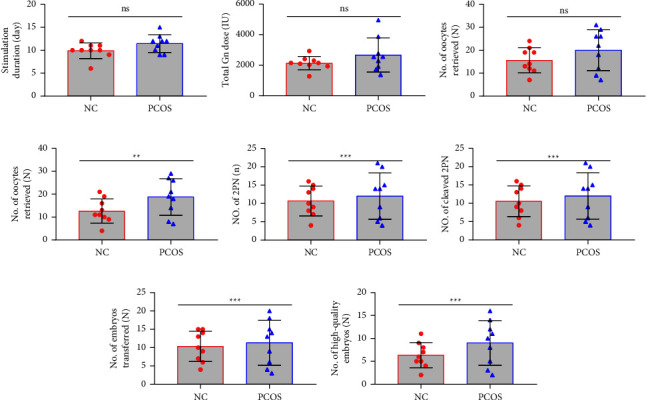
NKPOD changes the pregnancy outcome in PCOS patients. The changes in stimulation duration of Gn (a) and total Gn doses (b) in PCOS patients with NKPOD treatment. (c, d) The effect of NKPOD treatment on the number of oocytes punctured and oocytes retrieved in PCOS patients. The influence of NKPOD treatment on the number of 2PN (e) and 2PN cleavage numbers (f) in PCOS patients with NKPOD treatment. The influence of NKPOD treatment on the number of embryos transferred (g) and number of high-quality embryos (h) in PCOS patients receiving NKPOD administration. ns represents (*P* > 0.05), ^*∗∗*^(*P* < 0.01), and ^*∗∗∗*^(*P* < 0.001).

Subsequently, we further elucidated whether NKPOD influences reproductive outcomes in PCOS patients. Stimulation duration and total Gn doses were not changed in PCOS patients with NKPOD treatment (*P* > 0.05; Figures 2(a) and 2(b)). Notably, after exposure to NKPOD, we noted that the changes in the number of oocytes punctured and oocytes retrieved were not significant compared with those in the NC group (*P* > 0.05; Figure 2(c)). However, a higher number of oocytes were found in PCOS patients with NKPOD exposure (*P* < 0.05; Figure 2(d)). Furthermore, we noted that NKPOD administration significantly increased the number of 2PN and 2PN cleavage numbers (*P* < 0.05; Figures 2(e) and 2(f)). Consistent with 2PN, comparisons analysis demonstrated that NKPOD administration had significant effects on increasing the number of embryos transferred and high-quality embryos (*P* < 0.05; Figures 2(g) and 2(h)). We further evaluated the influences of NKPOD on reproductive outcomes and found that NKPOD treatment led to a significant increase in pregnancy rates in PCOS patients (*P* < 0.05; Table 2). Furthermore, we assessed the safety of NKPOD administration. Importantly, all PCOS patients were examined for blood, urine, stool routine, ECG, and liver and kidney function after each course of treatment. The results showed that there were no obvious abnormalities and that there were no serious adverse reactions in all patients. Taken together, our findings highlight the important role of NKPOD in improving reproductive outcomes in PCOS patients.

### 3.3. NKPOD Component-Target Network Construction

**Figure 3:**
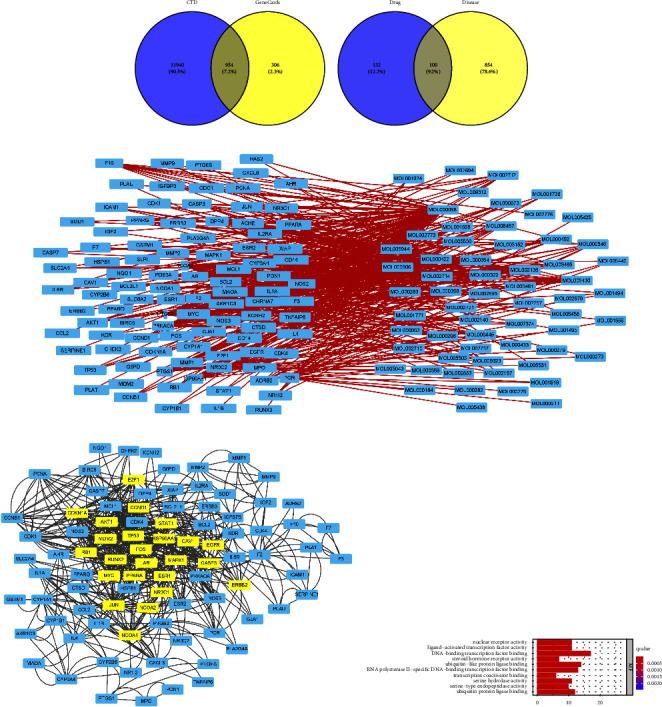
Network pharmacology was constructed to elucidate the candidate targets. (a) The Venn diagram of candidate genes related to PCOS. (b) The Venn diagram of candidate targets related to PCOS and NKPOD. (c) The component-target network. The left nodes represent bioactive compounds. The right nodes represent the intersection targets of PCOS and NKPOD. The edges represent the interactions among them. (d) The protein-protein interaction network. The yellow nodes represent core nodes. (e) The GO enrichment analysis of the 100 putative targets.

To elucidate the underlying molecular mechanism, in which NKPOD attenuating ovulation disorders, we investigated the targets of NKPOD through network pharmacology. The components and potential targets of Angelicae sinensis Radix, Paeoniae Radix alba, Rhizoma Dioscoreae, Rehmanniae Radix Preparata, Cortex Moutan, Poria, Semen Cuscutae, Dipsaci Radix, Cuscutae Semen, Cornu Cervi, Faeces Trogopterori, and Carthami flos, which constitute NKPOD, were identified, as displayed in Table S1. After screening with OB and DL, a total of 63 components were obtained from the TCMSP. 1857 possible targets of these components were also obtained from the TCMSP (Table S1). Subsequently, a total of 954 genes related to PCOS were obtained from the GeneCards database (1260 genes) and CTD (12894 genes), as shown in Figure 3(a) and Table S2. Interestingly, a total of 954 genes, which were both related to NKPOD and PCOS, were confirmed (Figure 3(b) and Table S3). As demonstrated in Figure 3(c), we structured the compound-target network, including 161 nodes, 61 bioactive compounds and 100 targets. Subsequently, the proposed protein-protein interactions of 100 targets are constructed and displayed in Figure 3(d). After hiding disconnected nodes, 91 nodes and 686 edges were found in the network. To investigate the crucial nodes, betweenness, closeness, degrees, eigenvectors, LAC, and networks were used to identify 24 nodes and 240 edges. To investigate the potential functions of these targets, we performed functional enrichment analysis and found 1867 GO biological terms (FDR cutoff = 0.01, Table S4 and Figure 3(e)). The results of Figure 3(e) revealed the top 10 enriched GO-BP terms, including nuclear receptor activity, ligand-activated transcription factor activity, DNA-binding transcription factor binding, steroid hormone receptor activity, ubiquitin-like protein ligase binding, RNA polymerase II-specificDNA-binding transcription factor binding, transcription coactivator-binding serine hydrolase activity, serine-type endopeptidase activity, and ubiquitin protein ligase binding.

### 3.4. NKPOD Inhibits AR Expression

**Figure 4:**
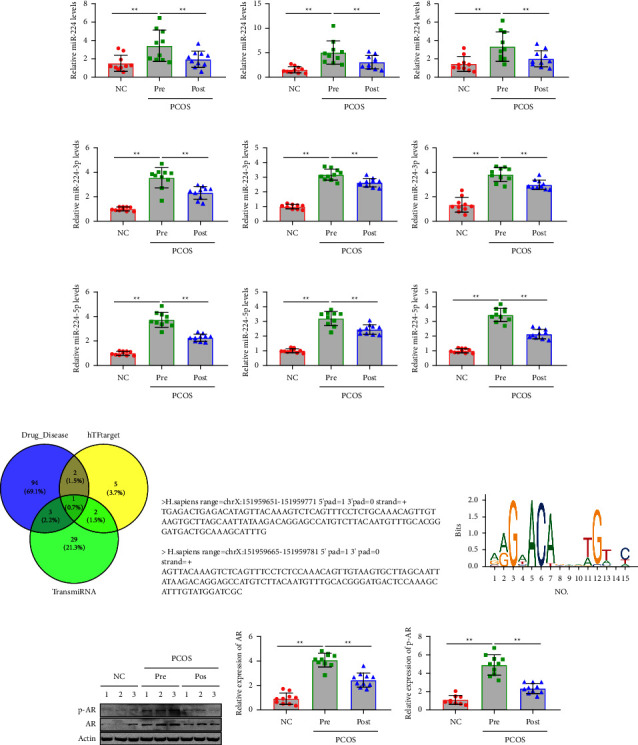
NKPOD regulates miRNA-224 transcription through AR. qRT-PCR detected relative miRNA-224 levels in granulosa cells (a), follicular fluid (b), and blood (c). qRT-PCR detected relative miRNA-224-3p levels in granulosa cells (d), follicular fluid (e), and blood (f). qRT-PCR detected relative miRNA-224-5p levels in granulosa cells (g), follicular fluid (h), and blood (i). (j) The Venn diagram of candidate genes related to NKPOD targets and transcription factors for miRNA-224. (k, l) The DNA motif of AR predicted from JASPAR (https://jaspar.genereg.net/) and 2 predicted binding sites of AR in miRNA-224 promoters. The regulation of NKPOD on AR expression (m, n) and AR phosphorylation (m, o). ^*∗∗*^(*P* < 0.01).

To investigate the possible mechanism of NKPOD on attenuating ovulation disorders, we measured miRNA-224 abundance in PCOS patients. As expected, significant increased miRNA-224 abundance was discovered in follicular fluid, granulosa cells, and serum of PCOS patients (Figures 4(a)–4(c)). Particularly, NKPOD showed a stronger ability to lower miRNA-224 abundance in follicular fluid, granulosa cells, and serum of PCOS patients (Figures 4(a)–4(c)). Similar results were obtained for miRNA-224-3p (Figures 4(d)–4(f)) and miRNA-224-5p (Figures 4(g)–4(i)). Taken together, these results imply that NKPOD inhibits miRNA-224 transcription. To understand the mechanism by which NKPOD regulated miRNA-224 transcription, we investigated the transcription factor for miRNA-224. Interestingly, AR was identified as the only target of miRNA-224 transcription factors and NKPOD regulatory targets (Figure 4(j)). The predicted sites of AR-binding in miRNA-224 promoters by TransmiRNA v2.0 (https://www.cuilab.cn/transmiRNA) are shown in Figure 4(k). Additionally, the motif of AR predicated by the JASPAR database (https://jaspar.genereg.net/) is displayed in Figure 4(l). Next, we explored the effects of NKPOD on the expression of AR in granulosa cells; western blotting was performed and displayed that AR expression was significantly upregulated in PCOS patients, whereas the increased AR expression was inhibited by NKPOD treatment (Figures 4(m) and 4(n)). Interestingly, the inhibition effect of NKPOD on phosphorylation of AR was determined, in which the elevated p-AR/AR levels in granulosa cells of PCOS patients were significantly attenuated by NKPOD treatment (Figures 4(m) and 4(o)).

### 3.5. miRNA-224 Is Confirmed as the Target of AR

**Figure 5:**
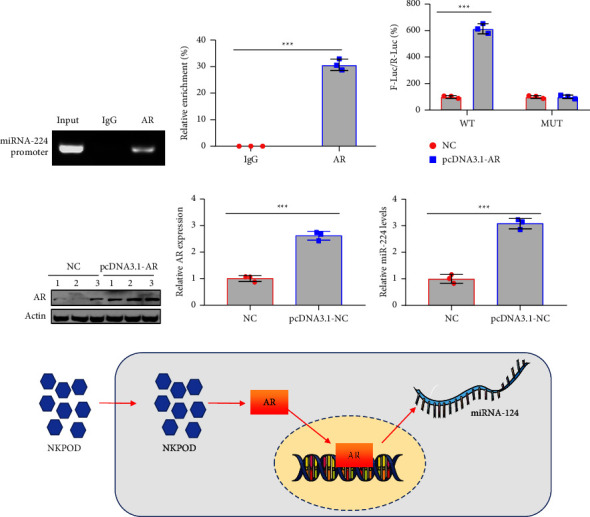
AR is the transcription factor for miRNA-224. (a, b) The affinity of AR in the promoter region of miRNA-224 assessed by the ChIP assay. (c) Luciferase activity detection verified the binding of AR to the miRNA-224 promoter. (d, e) The expression of AR in granulosa cells after transcription of pcDNA3.1-AR. (f) The levels of miRNA-224 in granulosa cells with AR overexpression. (g) A working model of NKPOD on attenuating polycystic ovary syndrome in PCOS patients via activating miRNA-224 transcription through AR. ^*∗∗∗*^(*P* < 0.001).

To further understand the mechanism by which AR regulated miRNA-224 transcription, the ChIP assay was used, and we pointed out that the miRNA-224 promoter was strongly enriched in the AR pellet in granulosa cells (Figures 5(a) and 5(b)). Subsequently, we performed the luciferase reporter assay through constructing the wild-type and mutant-binding sequences (Figure 5(c)). The results showed that relative luciferase activity was dramatically enhanced in the miRNA-224promoter-WT + AR overexpression group, but there was no apparent change in the miRNA-224promoter-MUT + AR overexpression group (Figures 5(d) and 5(e)). Next, we further investigated the regulation of AR on miRNA-224 transcription. The effects of AR overexpression on miRNA-224 transcription were further unveiled. qRT-PCR results showed that miRNA-224 expression was significantly upregulated in granulosa cells transfected with pcDNA3.1-AR compared with the cells transfected with pcDNA3.1-NC (Figure 5(f)). To sum up, AR is involved in miRNA-224 transcription.

## 4. Disscussion

Meta-analyses and systematic reviews of observational studies and trials with a small sample size have demonstrated the effectiveness of TCM in improving PCOS's clinical outcomes [12, 22, 23]. Although some components of NKPOD, as a traditional Chinese medicine decoction, have been widely used in the treatment of infertility and have shown some efficacy [23–25], pronounced knowledge gaps remain existed regarding the therapeutic effect of NKPOD on PCOS through systematic research and regulatory mechanisms. In the present study, we discovered that NKPOD administration can alleviate hormone and ovulatory aberrations caused by PCOS, including significantly reducing the levels of BMI, AMH, T, LH, and FSH and increasing the abundance of E2. Furthermore, we discovered that NKPOD treatment contributed to gestation in PCOS patients, increasing the number of oocytes, number of 2PN and 2PN cleavage numbers, and number of embryos transferred and high-quality embryos as well as pregnancy rates. Subsequently, we explored the regulatory mechanism and found that NKPOD inhibited AR expression, which indicates that AR expression is positively correlated with miRNA-224 transcription. In particular, AR directly regulated miRNA-224 transcription through binding with the promoter of miRNA-224. This study provides novel insights into understanding the efficacy and mechanism of NKPOD in improving ovulation disorders and provides an experimental basis for the clinical application of NKPOD in PCOS (Figure 5(g)).

Accumulating evidence indicates that an intrinsic abnormality of early follicle development and arrested follicular growth are critical factors in the pathogenesis of PCOS [8, 26]. Furthermore, hormonal perturbation will have a profound impact on adult endocrine and reproductive status [27, 28]. However, currently, there are still no effective drugs for PCOS, so it is of great significance to find new drugs for the treatment of PCOS. In this study, we sought to investigate the effect of NKPOD on hormonal levels of PCOS patients. Interestingly, our study provided clear evidence that NKPOD administration can alleviate the hormone dysregulation caused by PCOS, including significantly reducing the levels of BMI, AMH, T, LH, and FSH and increasing the abundance of E2. These beneficial effects provide a theoretical basis for the application of NKPOD in PCOS treatment. Subsequently, we first explored the benefit of NKPOD on the outcome of IVF-ET/ICSI. Interestingly, although there is no difference in some indicators, including stimulation duration of Gn, total Gn doses, and the number of oocytes punctured and oocytes retrieved, we found that PCOS patients with NKPOD treatment can obtain a higher number of oocytes, 2PN, 2PN cleavage numbers, embryos transferred, and high-quality embryos as well as higher pregnancy rates. At present, despite increased efforts to treat PCOS, the prognosis for improved fertility is usually poor in a considerable number of patients; the beneficial effect indicates that NKPOD is a promising treatment option for PCOS. Additionally, no obvious adverse reaction and higher pregnancy rates show the superiority of NKPOD treatment of PCOS, for PCOS ovulation disorder infertility TCM provides objective basis.

NKPOD consists of 12 ingredients: Angelicae sinensis Radix, Paeoniae Radix alba, Rhizoma Dioscoreae, Rehmanniae Radix Preparata, Cortex Moutan, Poria, Semen Cuscutae, Dipsaci Radix, Cuscutae Semen, Cornu Cervi, Faeces Trogopterori, and Carthami flos. Although the regulatory targets of some ingredients have been clarified, the regulatory relationship of NKPOD-target-PCOS has not been clarified. In recent years, network pharmacology has attracted global attention. Interestingly, network pharmacology provides an effective method for component screening and prediction of drug targets. In our study, we present the NKPOD-target relationships, which give a dependable result of the NKPOD effects on PCOS. A total of 100 potential targets including 24 core targets were identified to be associated with PCOS. Among the 24 core targets, some of them such as AR [29], epidermal growth factor receptor (EGFR) [30], and RAC-alpha serine/threonine-protein kinase (AKT1) [31] have previously been confirmed through experiments. For example, Jiang et al. [32] reported that miRNA-93 promotes ovarian granulosa cell proliferation through targeting cyclin-dependent kinase inhibitor 1A in PCOS. However, some targets such as peroxisome proliferator-activated receptor alpha and heat shock protein HSP 90-alpha have not yet been confirmed with regard to PCOS through experiments. Therefore, these putative targets that have been discovered provide a foundation for future research. Interestingly, through GO enrichment analysis, we noted that the 100 putative targets were mainly involved in the regulation of gene transcription. These potential targets and molecular function provide a basis for further research and the feasibility of TCM theory in guiding clinical NKPOD application.

At present, the pathogenesis of PCOS has not been fully elucidated. Changes in miRNAs are obvious pathology in PCOS progression, and miRNAs markers have been used to detect and study this disease [33, 34]. miRNA‐224 is one of the u-regulated miRNAs located in an intron of a transforming growth factor-*β* (TGF-*β*) responsive gene and gamma-aminobutyric acid receptor subunit epsilon [35, 36]. There are numerous *in vitro* studies that have shown the potential role of miRNA-224 in the ovaries. miRNA-224 has a regulatory effect on cumulus expansion [17, 37]. It has been previously reported that miRNA-224 negatively affected ovulation in the mouse model by decreasing the expression of Ptx3 and Smad4 [32]. Notably, in this study, negative regulation of NKPOD on miRNA-224, miRNA-224-3p, and miRNA-224-5p was noticed. Considering the regulation of NKPOD on putative targets including transcription factors as well as the function of transcription factor activity, we speculated that NKPOD may regulate miRNA-224 abundance by regulating the activity of transcription factors. Using bioinformatic methods, we discovered only one common target (AR), which is related to the transcription factor of miRNA-224, NKPOD targets, and PCOS-related genes. Due to the relationship between AR and PCOS [38], we believe that the regulation of miRNA-224 abundance by NKPOD requires the involvement of AR. As expected, AR was verified as the transcription factor activating miRNA-224 transcription. Mechanism investigation further validated that NKPOD administration eliminated the stability of AR. The combined data elucidate a crucial mechanism of NKPOD in improving ovulation disorder in PCOS patients.

However, we are aware of some limitations in this study. First, the sample size of PCOS patients was relatively limited, and larger population is necessary to validate the role of NKPOD. Furthermore, the regulation of NKPOD on other targets lacks experimental data support, so it will be interesting to study them in the future. In summary, to our knowledge, this is the first report that generally investigates the clinical implication and mechanism of NKPOD in PCOS. The results present that NKPOD improves ovulation disorder in PCOS patients through AR-mediated transcriptional inhibition of miRNA. These findings provide novel insights into understanding the efficacy and mechanism of NKPOD in improving ovulation disorder and provide an experimental basis for the clinical application of NKPOD in PCOS.

## Figures and Tables

**Table 1:** Comparison of general information in the two groups.

Item	NC group (*n* = 9)	PCOS group (*n* = 9)	P value
Age (year)	30.11 ± 3.59	29.78 ± 5.38	0.88
Primary infertility	7	7	0.71
Secondary infertility	2	2	
Course of disease (year)	2.56 ± 1.33	2.78 ± 1.64	0.76

**Table 2:** Comparison of ovulation and pregnancy between the two groups.

Item	NC group (*n* = 9)	PCOS group (*n* = 9)	*P* value
Ovulation	1	6	0.016
Gestation	1	5	0.046
Abortion	0	0	1

## Data Availability

The data used to support the findings of this study are available from the corresponding author upon request.

## Supplementary Materials

Supplementary Materials Table S1: the components and possible targets of NKPOD. Table S2: the common targets of the GeneCards Database and CTD. Table S3: the common targets related to NKPOD and PCOS. Table S4: GO biological terms.
