# Supplementary material for: Nourishing Kidney Promoting Ovulation Decoction (NKPOD) Attenuates Polycystic Ovary Syndrome by Downregulating miRNA-224
Source: Evid Based Complement Alternat Med. 2023 Apr 20;2023:9402155. doi: 10.1155/2023/9402155 (PMC10139811; doi:10.1155/2023/9402155)
Supplement: Supplementary Materials — Table S1: the components and possible targets of NKPOD. Table S2: the common targets of the GeneCards Database and CTD. Table S3: the common targets related to NKPOD and PCOS. Table S4: GO biological terms. [file 9402155.f1.zip › Table S1.pdf]

Table S1 The components and possible targets of NKPOD

**Drug**	**MolId**	**MolName**
Chibaishao	MOL001918	paeoniflorgenone
Chibaishao	MOL001919	(3S,5R,8R,9R,10S,14S)-3,17-dihydroxy-4,4,[illegible]
Chibaishao	MOL001919	(3S,5R,8R,9R,10S,14S)-3,17-dihydroxy-4,4,[illegible]
Chibaishao	MOL001924	paeoniflorin
Chibaishao	MOL001924	paeoniflorin
Chibaishao	MOL001924	paeoniflorin
Chibaishao	MOL001924	paeoniflorin
Chibaishao	MOL000211	Mairin
Chibaishao	MOL000358	beta-sitosterol
Chibaishao	MOL000358	beta-sitosterol
Chibaishao	MOL000358	beta-sitosterol
Chibaishao	MOL000358	beta-sitosterol
Chibaishao	MOL000358	beta-sitosterol
Chibaishao	MOL000358	beta-sitosterol
Chibaishao	MOL000358	beta-sitosterol
Chibaishao	MOL000358	beta-sitosterol
Chibaishao	MOL000358	beta-sitosterol
Chibaishao	MOL000358	beta-sitosterol
Chibaishao	MOL000358	beta-sitosterol
Chibaishao	MOL000358	beta-sitosterol
Chibaishao	MOL000358	beta-sitosterol
Chibaishao	MOL000358	beta-sitosterol
Chibaishao	MOL000358	beta-sitosterol
Chibaishao	MOL000358	beta-sitosterol
Chibaishao	MOL000358	beta-sitosterol
Chibaishao	MOL000358	beta-sitosterol
Chibaishao	MOL000358	beta-sitosterol
Chibaishao	MOL000358	beta-sitosterol
Chibaishao	MOL000358	beta-sitosterol
Chibaishao	MOL000358	beta-sitosterol
Chibaishao	MOL000358	beta-sitosterol
Chibaishao	MOL000358	beta-sitosterol
Chibaishao	MOL000358	beta-sitosterol
Chibaishao	MOL000358	beta-sitosterol
Chibaishao	MOL000358	beta-sitosterol
Chibaishao	MOL000358	beta-sitosterol
Chibaishao	MOL000358	beta-sitosterol
Chibaishao	MOL000358	beta-sitosterol
Chibaishao	MOL000358	beta-sitosterol
Chibaishao	MOL000358	beta-sitosterol
Chibaishao	MOL000358	beta-sitosterol
Chibaishao	MOL000358	beta-sitosterol
Chibaishao	MOL000358	beta-sitosterol
Chibaishao	MOL000358	beta-sitosterol
Chibaishao	MOL000359	sitosterol
Chibaishao	MOL000359	sitosterol
Chibaishao	MOL000359	sitosterol
Chibaishao	MOL000422	kaempferol
Chibaishao	MOL000422	kaempferol
Chibaishao	MOL000422	kaempferol
Chibaishao	MOL000422	kaempferol
Chibaishao	MOL000422	kaempferol
Chibaishao	MOL000422	kaempferol
Chibaishao	MOL000422	kaempferol

Chibaishao	MOL000422	kaempferol
Chibaishao	MOL000422	kaempferol
Chibaishao	MOL000422	kaempferol
Chibaishao	MOL000422	kaempferol
Chibaishao	MOL000422	kaempferol
Chibaishao	MOL000422	kaempferol
Chibaishao	MOL000422	kaempferol
Chibaishao	MOL000422	kaempferol
Chibaishao	MOL000422	kaempferol
Chibaishao	MOL000422	kaempferol
Chibaishao	MOL000422	kaempferol
Chibaishao	MOL000422	kaempferol
Chibaishao	MOL000422	kaempferol
Chibaishao	MOL000422	kaempferol
Chibaishao	MOL000422	kaempferol
Chibaishao	MOL000422	kaempferol
Chibaishao	MOL000422	kaempferol
Chibaishao	MOL000422	kaempferol
Chibaishao	MOL000422	kaempferol
Chibaishao	MOL000422	kaempferol
Chibaishao	MOL000422	kaempferol
Chibaishao	MOL000422	kaempferol
Chibaishao	MOL000422	kaempferol
Chibaishao	MOL000422	kaempferol
Chibaishao	MOL000422	kaempferol
Chibaishao	MOL000422	kaempferol
Chibaishao	MOL000422	kaempferol
Chibaishao	MOL000422	kaempferol
Chibaishao	MOL000422	kaempferol
Chibaishao	MOL000422	kaempferol
Chibaishao	MOL000422	kaempferol
Chibaishao	MOL000422	kaempferol
Chibaishao	MOL000422	kaempferol
Chibaishao	MOL000422	kaempferol
Chibaishao	MOL000422	kaempferol
Chibaishao	MOL000422	kaempferol
Chibaishao	MOL000422	kaempferol
Chibaishao	MOL000422	kaempferol
Chibaishao	MOL000422	kaempferol
Chibaishao	MOL000422	kaempferol
Chibaishao	MOL000422	kaempferol
Chibaishao	MOL000422	kaempferol
Chibaishao	MOL000422	kaempferol
Chibaishao	MOL000422	kaempferol
Chibaishao	MOL000422	kaempferol
Chibaishao	MOL000422	kaempferol
Chibaishao	MOL000422	kaempferol
Chibaishao	MOL000422	kaempferol
Chibaishao	MOL000422	kaempferol
Chibaishao	MOL000422	kaempferol
Chibaishao	MOL000422	kaempferol
Chibaishao	MOL000422	kaempferol
Chibaishao	MOL000422	kaempferol
Chibaishao	MOL000422	kaempferol
Chibaishao	MOL000422	kaempferol
Chibaishao	MOL000492	(+)-catechin
Chibaishao	MOL000492	(+)-catechin
Chibaishao	MOL000492	(+)-catechin

Chibaishao	MOL000492	(+)-catechin
Chibaishao	MOL000492	(+)-catechin
Chibaishao	MOL000492	(+)-catechin
Chibaishao	MOL000492	(+)-catechin
Chibaishao	MOL000492	(+)-catechin
Chibaishao	MOL000492	(+)-catechin
Chibaishao	MOL000492	(+)-catechin
Chibaishao	MOL000492	(+)-catechin
Chuanduan	MOL003152	Gentisin
Chuanduan	MOL003152	Gentisin
Chuanduan	MOL003152	Gentisin
Chuanduan	MOL003152	Gentisin
Chuanduan	MOL003152	Gentisin
Chuanduan	MOL003152	Gentisin
Chuanduan	MOL003152	Gentisin
Chuanduan	MOL003152	Gentisin
Chuanduan	MOL003152	Gentisin
Chuanduan	MOL003152	Gentisin
Chuanduan	MOL003152	Gentisin
Chuanduan	MOL003152	Gentisin
Chuanduan	MOL003152	Gentisin
Chuanduan	MOL003152	Gentisin
Chuanduan	MOL000358	beta-sitosterol
Chuanduan	MOL000358	beta-sitosterol
Chuanduan	MOL000358	beta-sitosterol
Chuanduan	MOL000358	beta-sitosterol
Chuanduan	MOL000358	beta-sitosterol
Chuanduan	MOL000358	beta-sitosterol
Chuanduan	MOL000358	beta-sitosterol
Chuanduan	MOL000358	beta-sitosterol
Chuanduan	MOL000358	beta-sitosterol
Chuanduan	MOL000358	beta-sitosterol
Chuanduan	MOL000358	beta-sitosterol
Chuanduan	MOL000358	beta-sitosterol
Chuanduan	MOL000358	beta-sitosterol
Chuanduan	MOL000358	beta-sitosterol
Chuanduan	MOL000358	beta-sitosterol
Chuanduan	MOL000358	beta-sitosterol
Chuanduan	MOL000358	beta-sitosterol
Chuanduan	MOL000358	beta-sitosterol
Chuanduan	MOL000358	beta-sitosterol
Chuanduan	MOL000358	beta-sitosterol
Chuanduan	MOL000358	beta-sitosterol
Chuanduan	MOL000358	beta-sitosterol
Chuanduan	MOL000358	beta-sitosterol
Chuanduan	MOL000358	beta-sitosterol
Chuanduan	MOL000358	beta-sitosterol
Chuanduan	MOL000358	beta-sitosterol
Chuanduan	MOL000358	beta-sitosterol
Chuanduan	MOL000358	beta-sitosterol
Chuanduan	MOL000358	beta-sitosterol
Chuanduan	MOL000358	beta-sitosterol
Chuanduan	MOL000358	beta-sitosterol
Chuanduan	MOL000358	beta-sitosterol
Chuanduan	MOL000358	beta-sitosterol
Chuanduan	MOL000358	beta-sitosterol
Chuanduan	MOL000358	beta-sitosterol
Chuanduan	MOL000358	beta-sitosterol

Chuanduan	MOL000358	beta-sitosterol
Chuanduan	MOL000358	beta-sitosterol
Chuanduan	MOL000359	sitosterol
Chuanduan	MOL000359	sitosterol
Chuanduan	MOL000359	sitosterol
Chuanduan	MOL009312	(E,E)-3,5-Di-O-caffeoylquinic acid
Chuanduan	MOL009312	(E,E)-3,5-Di-O-caffeoylquinic acid
Chuanduan	MOL009323	Sylvestroside III_qt
Chuanduan	MOL009323	Sylvestroside III_qt
Chuanduan	MOL009323	Sylvestroside III_qt
Chuanduan	MOL009323	Sylvestroside III_qt
Chuanduan	MOL009323	Sylvestroside III_qt
Chuanduan	MOL009323	Sylvestroside III_qt
Chuanxiong	MOL001494	Mandenol
Chuanxiong	MOL001494	Mandenol
Chuanxiong	MOL001494	Mandenol
Chuanxiong	MOL002135	Myricanone
Chuanxiong	MOL002135	Myricanone
Chuanxiong	MOL002135	Myricanone
Chuanxiong	MOL002135	Myricanone
Chuanxiong	MOL002135	Myricanone
Chuanxiong	MOL002135	Myricanone
Chuanxiong	MOL002135	Myricanone
Chuanxiong	MOL002135	Myricanone
Chuanxiong	MOL002135	Myricanone
Chuanxiong	MOL002135	Myricanone
Chuanxiong	MOL002135	Myricanone
Chuanxiong	MOL002135	Myricanone
Chuanxiong	MOL002135	Myricanone
Chuanxiong	MOL002135	Myricanone
Chuanxiong	MOL002135	Myricanone
Chuanxiong	MOL002135	Myricanone
Chuanxiong	MOL002135	Myricanone
Chuanxiong	MOL002135	Myricanone
Chuanxiong	MOL002135	Myricanone
Chuanxiong	MOL002135	Myricanone
Chuanxiong	MOL002135	Myricanone
Chuanxiong	MOL002135	Myricanone
Chuanxiong	MOL002135	Myricanone
Chuanxiong	MOL002135	Myricanone
Chuanxiong	MOL002135	Myricanone
Chuanxiong	MOL002140	Perlolyrine
Chuanxiong	MOL002140	Perlolyrine
Chuanxiong	MOL002140	Perlolyrine
Chuanxiong	MOL002140	Perlolyrine
Chuanxiong	MOL002157	wallichilide
Chuanxiong	MOL002157	wallichilide
Chuanxiong	MOL002157	wallichilide
Chuanxiong	MOL002157	wallichilide
Chuanxiong	MOL000359	sitosterol
Chuanxiong	MOL000359	sitosterol
Chuanxiong	MOL000359	sitosterol
Chuanxiong	MOL000433	FA
Chuanxiong	MOL000433	FA
Chuanxiong	MOL000433	FA
Danpi	MOL000211	Mairin
Danpi	MOL000359	sitosterol
Danpi	MOL000359	sitosterol

Danpi	MOL000359	sitosterol
Danpi	MOL000422	kaempferol
Danpi	MOL000422	kaempferol
Danpi	MOL000422	kaempferol
Danpi	MOL000422	kaempferol
Danpi	MOL000422	kaempferol
Danpi	MOL000422	kaempferol
Danpi	MOL000422	kaempferol
Danpi	MOL000422	kaempferol
Danpi	MOL000422	kaempferol
Danpi	MOL000422	kaempferol
Danpi	MOL000422	kaempferol
Danpi	MOL000422	kaempferol
Danpi	MOL000422	kaempferol
Danpi	MOL000422	kaempferol
Danpi	MOL000422	kaempferol
Danpi	MOL000422	kaempferol
Danpi	MOL000422	kaempferol
Danpi	MOL000422	kaempferol
Danpi	MOL000422	kaempferol
Danpi	MOL000422	kaempferol
Danpi	MOL000422	kaempferol
Danpi	MOL000422	kaempferol
Danpi	MOL000422	kaempferol
Danpi	MOL000422	kaempferol
Danpi	MOL000422	kaempferol
Danpi	MOL000422	kaempferol
Danpi	MOL000422	kaempferol
Danpi	MOL000422	kaempferol
Danpi	MOL000422	kaempferol
Danpi	MOL000422	kaempferol
Danpi	MOL000422	kaempferol
Danpi	MOL000422	kaempferol
Danpi	MOL000422	kaempferol
Danpi	MOL000422	kaempferol
Danpi	MOL000422	kaempferol
Danpi	MOL000422	kaempferol
Danpi	MOL000422	kaempferol
Danpi	MOL000422	kaempferol
Danpi	MOL000422	kaempferol
Danpi	MOL000422	kaempferol
Danpi	MOL000422	kaempferol
Danpi	MOL000422	kaempferol
Danpi	MOL000422	kaempferol
Danpi	MOL000422	kaempferol
Danpi	MOL000422	kaempferol
Danpi	MOL000422	kaempferol
Danpi	MOL000422	kaempferol
Danpi	MOL000422	kaempferol
Danpi	MOL000422	kaempferol
Danpi	MOL000422	kaempferol
Danpi	MOL000422	kaempferol
Danpi	MOL000422	kaempferol
Danpi	MOL000422	kaempferol
Danpi	MOL000422	kaempferol
Danpi	MOL000422	kaempferol
Danpi	MOL000422	kaempferol
Danpi	MOL000422	kaempferol

Danpi	MOL000422	kaempferol
Danpi	MOL000422	kaempferol
Danpi	MOL000422	kaempferol
Danpi	MOL000422	kaempferol
Danpi	MOL000422	kaempferol
Danpi	MOL000492	(+)-catechin
Danpi	MOL000492	(+)-catechin
Danpi	MOL000492	(+)-catechin
Danpi	MOL000492	(+)-catechin
Danpi	MOL000492	(+)-catechin
Danpi	MOL000492	(+)-catechin
Danpi	MOL000492	(+)-catechin
Danpi	MOL000492	(+)-catechin
Danpi	MOL000492	(+)-catechin
Danpi	MOL000492	(+)-catechin
Danpi	MOL000492	(+)-catechin
Danpi	MOL007374	5-[[5-(4-methoxyphenyl)-2-furyl]methyler
Danpi	MOL007374	5-[[5-(4-methoxyphenyl)-2-furyl]methyler
Danpi	MOL007374	5-[[5-(4-methoxyphenyl)-2-furyl]methyler
Danpi	MOL007374	5-[[5-(4-methoxyphenyl)-2-furyl]methyler
Danpi	MOL007374	5-[[5-(4-methoxyphenyl)-2-furyl]methyler
Danpi	MOL000098	quercetin
Danpi	MOL000098	quercetin
Danpi	MOL000098	quercetin
Danpi	MOL000098	quercetin
Danpi	MOL000098	quercetin
Danpi	MOL000098	quercetin
Danpi	MOL000098	quercetin
Danpi	MOL000098	quercetin
Danpi	MOL000098	quercetin
Danpi	MOL000098	quercetin
Danpi	MOL000098	quercetin
Danpi	MOL000098	quercetin
Danpi	MOL000098	quercetin
Danpi	MOL000098	quercetin
Danpi	MOL000098	quercetin
Danpi	MOL000098	quercetin
Danpi	MOL000098	quercetin
Danpi	MOL000098	quercetin
Danpi	MOL000098	quercetin
Danpi	MOL000098	quercetin
Danpi	MOL000098	quercetin
Danpi	MOL000098	quercetin
Danpi	MOL000098	quercetin
Danpi	MOL000098	quercetin
Danpi	MOL000098	quercetin
Danpi	MOL000098	quercetin
Danpi	MOL000098	quercetin
Danpi	MOL000098	quercetin
Danpi	MOL000098	quercetin
Danpi	MOL000098	quercetin
Danpi	MOL000098	quercetin
Danpi	MOL000098	quercetin
Danpi	MOL000098	quercetin
Danpi	MOL000098	quercetin
Danpi	MOL000098	quercetin
Danpi	MOL000098	quercetin
Danpi	MOL000098	quercetin

Danpi	MOL000098	quercetin
Danpi	MOL000098	quercetin
Danpi	MOL000098	quercetin
Danpi	MOL000098	quercetin
Danpi	MOL000098	quercetin
Danpi	MOL000098	quercetin
Danpi	MOL000098	quercetin
Danpi	MOL000098	quercetin
Danpi	MOL000098	quercetin
Danpi	MOL000098	quercetin
Danpi	MOL000098	quercetin
Danpi	MOL000098	quercetin
Danpi	MOL000098	quercetin
Danpi	MOL000098	quercetin
Danpi	MOL000098	quercetin
Danpi	MOL000098	quercetin
Danpi	MOL000098	quercetin
Danpi	MOL000098	quercetin
Danpi	MOL000098	quercetin
Danpi	MOL000098	quercetin
Danpi	MOL000098	quercetin
Danpi	MOL000098	quercetin
Danpi	MOL000098	quercetin
Danpi	MOL000098	quercetin
Danpi	MOL000098	quercetin
Danpi	MOL000098	quercetin
Danpi	MOL000098	quercetin
Danpi	MOL000098	quercetin
Danpi	MOL000098	quercetin
Danpi	MOL000098	quercetin
Danpi	MOL000098	quercetin
Danpi	MOL000098	quercetin
Danpi	MOL000098	quercetin
Danpi	MOL000098	quercetin
Danpi	MOL000098	quercetin
Danpi	MOL000098	quercetin
Danpi	MOL000098	quercetin
Danpi	MOL000098	quercetin
Danpi	MOL000098	quercetin
Danpi	MOL000098	quercetin
Danpi	MOL000098	quercetin
Danpi	MOL000098	quercetin
Danpi	MOL000098	quercetin
Danpi	MOL000098	quercetin
Danpi	MOL000098	quercetin
Danpi	MOL000098	quercetin
Danpi	MOL000098	quercetin
Danpi	MOL000098	quercetin
Danpi	MOL000098	quercetin
Danpi	MOL000098	quercetin
Danpi	MOL000098	quercetin
Danpi	MOL000098	quercetin
Danpi	MOL000098	quercetin
Danpi	MOL000098	quercetin
Danpi	MOL000098	quercetin
Danpi	MOL000098	quercetin
Danpi	MOL000098	quercetin
Danpi	MOL000098	quercetin

Danpi	MOL000098	quercetin
Danpi	MOL000098	quercetin
Danpi	MOL000098	quercetin
Danpi	MOL000098	quercetin
Danpi	MOL000098	quercetin
Danpi	MOL000098	quercetin
Danpi	MOL000098	quercetin
Danpi	MOL000098	quercetin
Danpi	MOL000098	quercetin
Danpi	MOL000098	quercetin
Danpi	MOL000098	quercetin
Danpi	MOL000098	quercetin
Danpi	MOL000098	quercetin
Danpi	MOL000098	quercetin
Danpi	MOL000098	quercetin
Danpi	MOL000098	quercetin
Danpi	MOL000098	quercetin
Danpi	MOL000098	quercetin
Danpi	MOL000098	quercetin
Danpi	MOL000098	quercetin
Danpi	MOL000098	quercetin
Danpi	MOL000098	quercetin
Danpi	MOL000098	quercetin
Danpi	MOL000098	quercetin
Danpi	MOL000098	quercetin
Danpi	MOL000098	quercetin
Danpi	MOL000098	quercetin
Danpi	MOL000098	quercetin
Danpi	MOL000098	quercetin
Danpi	MOL000098	quercetin
Danpi	MOL000098	quercetin
Danpi	MOL000098	quercetin
Danpi	MOL000098	quercetin
Danpi	MOL000098	quercetin
Danpi	MOL000098	quercetin
Danpi	MOL000098	quercetin
Danpi	MOL000098	quercetin
Danpi	MOL000098	quercetin
Danpi	MOL000098	quercetin
Danpi	MOL000098	quercetin
Danpi	MOL000098	quercetin
Danpi	MOL000098	quercetin
Danpi	MOL000098	quercetin
Danpi	MOL000098	quercetin
Danpi	MOL000098	quercetin
Danpi	MOL000098	quercetin
Danpi	MOL000098	quercetin
Danpi	MOL000098	quercetin
Danpi	MOL000098	quercetin
Danpi	MOL000098	quercetin
Danpi	MOL000098	quercetin
Danpi	MOL000098	quercetin
Danpi	MOL000098	quercetin
Fuling	MOL000273	(2R)-2-[(3S,5R,10S,13R,14R,16R,17R)-3,16-
Fuling	MOL000273	(2R)-2-[(3S,5R,10S,13R,14R,16R,17R)-3,16-
Fuling	MOL000275	trametenolic acid
Fuling	MOL000279	Cerevisterol
Fuling	MOL000282	ergosta-7,22E-dien-3beta-ol

Fuling	MOL000283	Ergosterol peroxide
Fuling	MOL000296	hederagenin
Fuling	MOL000296	hederagenin
Fuling	MOL000296	hederagenin
Fuling	MOL000296	hederagenin
Fuling	MOL000296	hederagenin
Fuling	MOL000296	hederagenin
Fuling	MOL000296	hederagenin
Fuling	MOL000296	hederagenin
Fuling	MOL000296	hederagenin
Fuling	MOL000296	hederagenin
Fuling	MOL000296	hederagenin
Fuling	MOL000296	hederagenin
Fuling	MOL000296	hederagenin
Fuling	MOL000296	hederagenin
Fuling	MOL000296	hederagenin
Fuling	MOL000296	hederagenin
Fuling	MOL000296	hederagenin
Fuling	MOL000296	hederagenin
Fuling	MOL000296	hederagenin
Fuling	MOL000296	hederagenin
Fuling	MOL000296	hederagenin
Fuling	MOL000296	hederagenin
Fuling	MOL000296	hederagenin
Fuling	MOL000296	hederagenin
Honghua	MOL001771	poriferast-5-en-3beta-ol
Honghua	MOL001771	poriferast-5-en-3beta-ol
Honghua	MOL002694	4-[(E)-4-(3,5-dimethoxy-4-oxo-1-cyclohe
Honghua	MOL002694	4-[(E)-4-(3,5-dimethoxy-4-oxo-1-cyclohe
Honghua	MOL002694	4-[(E)-4-(3,5-dimethoxy-4-oxo-1-cyclohe
Honghua	MOL002695	lignan
Honghua	MOL002695	lignan
Honghua	MOL002695	lignan
Honghua	MOL002695	lignan
Honghua	MOL002695	lignan
Honghua	MOL002695	lignan
Honghua	MOL002695	lignan
Honghua	MOL002695	lignan
Honghua	MOL002710	Pyrethrin II
Honghua	MOL002710	Pyrethrin II
Honghua	MOL002712	6-Hydroxykaempferol
Honghua	MOL002712	6-Hydroxykaempferol
Honghua	MOL002712	6-Hydroxykaempferol
Honghua	MOL002712	6-Hydroxykaempferol
Honghua	MOL002712	6-Hydroxykaempferol
Honghua	MOL002712	6-Hydroxykaempferol
Honghua	MOL002712	6-Hydroxykaempferol
Honghua	MOL002712	6-Hydroxykaempferol
Honghua	MOL002712	6-Hydroxykaempferol
Honghua	MOL002712	6-Hydroxykaempferol
Honghua	MOL002714	baicalein
Honghua	MOL002714	baicalein
Honghua	MOL002714	baicalein
Honghua	MOL002714	baicalein
Honghua	MOL002714	baicalein
Honghua	MOL002714	baicalein
Honghua	MOL002714	baicalein
Honghua	MOL002714	baicalein

Honghua	MOL002714	baicalein
Honghua	MOL002714	baicalein
Honghua	MOL002714	baicalein
Honghua	MOL002714	baicalein
Honghua	MOL002714	baicalein
Honghua	MOL002714	baicalein
Honghua	MOL002714	baicalein
Honghua	MOL002714	baicalein
Honghua	MOL002714	baicalein
Honghua	MOL002714	baicalein
Honghua	MOL002714	baicalein
Honghua	MOL002714	baicalein
Honghua	MOL002714	baicalein
Honghua	MOL002714	baicalein
Honghua	MOL002714	baicalein
Honghua	MOL002714	baicalein
Honghua	MOL002714	baicalein
Honghua	MOL002714	baicalein
Honghua	MOL002714	baicalein
Honghua	MOL002714	baicalein
Honghua	MOL002714	baicalein
Honghua	MOL002714	baicalein
Honghua	MOL002714	baicalein
Honghua	MOL002714	baicalein
Honghua	MOL002714	baicalein
Honghua	MOL002714	baicalein
Honghua	MOL002714	baicalein
Honghua	MOL002714	baicalein
Honghua	MOL002714	baicalein
Honghua	MOL002717	qt_carthamone
Honghua	MOL002717	qt_carthamone
Honghua	MOL002717	qt_carthamone
Honghua	MOL002721	quercetagetin
Honghua	MOL002721	quercetagetin
Honghua	MOL002721	quercetagetin
Honghua	MOL002721	quercetagetin
Honghua	MOL002721	quercetagetin
Honghua	MOL002721	quercetagetin
Honghua	MOL002721	quercetagetin
Honghua	MOL002721	quercetagetin
Honghua	MOL002757	7,8-dimethyl-1H-pyrimido[5,6-g]quinoxali
Honghua	MOL002757	7,8-dimethyl-1H-pyrimido[5,6-g]quinoxali
Honghua	MOL002757	7,8-dimethyl-1H-pyrimido[5,6-g]quinoxali
Honghua	MOL002757	7,8-dimethyl-1H-pyrimido[5,6-g]quinoxali
Honghua	MOL002757	7,8-dimethyl-1H-pyrimido[5,6-g]quinoxali
Honghua	MOL002773	beta-carotene
Honghua	MOL002773	beta-carotene
Honghua	MOL002773	beta-carotene
Honghua	MOL002773	beta-carotene
Honghua	MOL002773	beta-carotene
Honghua	MOL002773	beta-carotene
Honghua	MOL002773	beta-carotene
Honghua	MOL002773	beta-carotene
Honghua	MOL002773	beta-carotene
Honghua	MOL002773	beta-carotene
Honghua	MOL002773	beta-carotene
Honghua	MOL002773	beta-carotene
Honghua	MOL002773	beta-carotene

Honghua	MOL002773	beta-carotene
Honghua	MOL002773	beta-carotene
Honghua	MOL002773	beta-carotene
Honghua	MOL002773	beta-carotene
Honghua	MOL002773	beta-carotene
Honghua	MOL002773	beta-carotene
Honghua	MOL002773	beta-carotene
Honghua	MOL002776	Baicalin
Honghua	MOL002776	Baicalin
Honghua	MOL000358	beta-sitosterol
Honghua	MOL000358	beta-sitosterol
Honghua	MOL000358	beta-sitosterol
Honghua	MOL000358	beta-sitosterol
Honghua	MOL000358	beta-sitosterol
Honghua	MOL000358	beta-sitosterol
Honghua	MOL000358	beta-sitosterol
Honghua	MOL000358	beta-sitosterol
Honghua	MOL000358	beta-sitosterol
Honghua	MOL000358	beta-sitosterol
Honghua	MOL000358	beta-sitosterol
Honghua	MOL000358	beta-sitosterol
Honghua	MOL000358	beta-sitosterol
Honghua	MOL000358	beta-sitosterol
Honghua	MOL000358	beta-sitosterol
Honghua	MOL000358	beta-sitosterol
Honghua	MOL000358	beta-sitosterol
Honghua	MOL000358	beta-sitosterol
Honghua	MOL000358	beta-sitosterol
Honghua	MOL000358	beta-sitosterol
Honghua	MOL000358	beta-sitosterol
Honghua	MOL000358	beta-sitosterol
Honghua	MOL000358	beta-sitosterol
Honghua	MOL000358	beta-sitosterol
Honghua	MOL000358	beta-sitosterol
Honghua	MOL000358	beta-sitosterol
Honghua	MOL000358	beta-sitosterol
Honghua	MOL000358	beta-sitosterol
Honghua	MOL000358	beta-sitosterol
Honghua	MOL000358	beta-sitosterol
Honghua	MOL000358	beta-sitosterol
Honghua	MOL000358	beta-sitosterol
Honghua	MOL000358	beta-sitosterol
Honghua	MOL000358	beta-sitosterol
Honghua	MOL000358	beta-sitosterol
Honghua	MOL000358	beta-sitosterol
Honghua	MOL000358	beta-sitosterol
Honghua	MOL000358	beta-sitosterol
Honghua	MOL000422	kaempferol
Honghua	MOL000422	kaempferol
Honghua	MOL000422	kaempferol
Honghua	MOL000422	kaempferol
Honghua	MOL000422	kaempferol
Honghua	MOL000422	kaempferol
Honghua	MOL000422	kaempferol
Honghua	MOL000422	kaempferol
Honghua	MOL000422	kaempferol
Honghua	MOL000422	kaempferol
Honghua	MOL000422	kaempferol

Honghua	MOL000422	kaempferol
Honghua	MOL000422	kaempferol
Honghua	MOL000422	kaempferol
Honghua	MOL000422	kaempferol
Honghua	MOL000422	kaempferol
Honghua	MOL000422	kaempferol
Honghua	MOL000422	kaempferol
Honghua	MOL000422	kaempferol
Honghua	MOL000422	kaempferol
Honghua	MOL000422	kaempferol
Honghua	MOL000422	kaempferol
Honghua	MOL000422	kaempferol
Honghua	MOL000422	kaempferol
Honghua	MOL000422	kaempferol
Honghua	MOL000422	kaempferol
Honghua	MOL000422	kaempferol
Honghua	MOL000422	kaempferol
Honghua	MOL000422	kaempferol
Honghua	MOL000422	kaempferol
Honghua	MOL000422	kaempferol
Honghua	MOL000422	kaempferol
Honghua	MOL000422	kaempferol
Honghua	MOL000422	kaempferol
Honghua	MOL000422	kaempferol
Honghua	MOL000422	kaempferol
Honghua	MOL000422	kaempferol
Honghua	MOL000422	kaempferol
Honghua	MOL000422	kaempferol
Honghua	MOL000422	kaempferol
Honghua	MOL000422	kaempferol
Honghua	MOL000422	kaempferol
Honghua	MOL000422	kaempferol
Honghua	MOL000422	kaempferol
Honghua	MOL000422	kaempferol
Honghua	MOL000422	kaempferol
Honghua	MOL000422	kaempferol
Honghua	MOL000422	kaempferol
Honghua	MOL000422	kaempferol
Honghua	MOL000422	kaempferol
Honghua	MOL000422	kaempferol
Honghua	MOL000422	kaempferol
Honghua	MOL000422	kaempferol
Honghua	MOL000422	kaempferol
Honghua	MOL000422	kaempferol
Honghua	MOL000422	kaempferol
Honghua	MOL000422	kaempferol
Honghua	MOL000422	kaempferol
Honghua	MOL000422	kaempferol
Honghua	MOL000422	kaempferol
Honghua	MOL000422	kaempferol
Honghua	MOL000422	kaempferol
Honghua	MOL000449	Stigmasterol
Honghua	MOL000449	Stigmasterol
Honghua	MOL000449	Stigmasterol
Honghua	MOL000449	Stigmasterol
Honghua	MOL000449	Stigmasterol
Honghua	MOL000449	Stigmasterol
Honghua	MOL000449	Stigmasterol

Honghua	MOL000449	Stigmasterol
Honghua	MOL000449	Stigmasterol
Honghua	MOL000449	Stigmasterol
Honghua	MOL000449	Stigmasterol
Honghua	MOL000449	Stigmasterol
Honghua	MOL000449	Stigmasterol
Honghua	MOL000449	Stigmasterol
Honghua	MOL000449	Stigmasterol
Honghua	MOL000449	Stigmasterol
Honghua	MOL000449	Stigmasterol
Honghua	MOL000449	Stigmasterol
Honghua	MOL000449	Stigmasterol
Honghua	MOL000449	Stigmasterol
Honghua	MOL000449	Stigmasterol
Honghua	MOL000449	Stigmasterol
Honghua	MOL000449	Stigmasterol
Honghua	MOL000449	Stigmasterol
Honghua	MOL000449	Stigmasterol
Honghua	MOL000449	Stigmasterol
Honghua	MOL000449	Stigmasterol
Honghua	MOL000449	Stigmasterol
Honghua	MOL000449	Stigmasterol
Honghua	MOL000449	Stigmasterol
Honghua	MOL000449	Stigmasterol
Honghua	MOL000006	luteolin
Honghua	MOL000006	luteolin
Honghua	MOL000006	luteolin
Honghua	MOL000006	luteolin
Honghua	MOL000006	luteolin
Honghua	MOL000006	luteolin
Honghua	MOL000006	luteolin
Honghua	MOL000006	luteolin
Honghua	MOL000006	luteolin
Honghua	MOL000006	luteolin
Honghua	MOL000006	luteolin
Honghua	MOL000006	luteolin
Honghua	MOL000006	luteolin
Honghua	MOL000006	luteolin
Honghua	MOL000006	luteolin
Honghua	MOL000006	luteolin
Honghua	MOL000006	luteolin
Honghua	MOL000006	luteolin
Honghua	MOL000006	luteolin
Honghua	MOL000006	luteolin
Honghua	MOL000006	luteolin
Honghua	MOL000006	luteolin
Honghua	MOL000006	luteolin
Honghua	MOL000006	luteolin
Honghua	MOL000006	luteolin
Honghua	MOL000006	luteolin
Honghua	MOL000006	luteolin
Honghua	MOL000006	luteolin
Honghua	MOL000006	luteolin
Honghua	MOL000006	luteolin
Honghua	MOL000006	luteolin
Honghua	MOL000006	luteolin
Honghua	MOL000006	luteolin
Honghua	MOL000006	luteolin

Honghua	MOL000006	luteolin
Honghua	MOL000006	luteolin
Honghua	MOL000006	luteolin
Honghua	MOL000006	luteolin
Honghua	MOL000006	luteolin
Honghua	MOL000006	luteolin
Honghua	MOL000006	luteolin
Honghua	MOL000006	luteolin
Honghua	MOL000006	luteolin
Honghua	MOL000006	luteolin
Honghua	MOL000006	luteolin
Honghua	MOL000006	luteolin
Honghua	MOL000006	luteolin
Honghua	MOL000006	luteolin
Honghua	MOL000006	luteolin
Honghua	MOL000006	luteolin
Honghua	MOL000006	luteolin
Honghua	MOL000006	luteolin
Honghua	MOL000006	luteolin
Honghua	MOL000006	luteolin
Honghua	MOL000006	luteolin
Honghua	MOL000006	luteolin
Honghua	MOL000953	CLR
Honghua	MOL000953	CLR
Honghua	MOL000953	CLR
Honghua	MOL000953	CLR
Honghua	MOL000098	quercetin
Honghua	MOL000098	quercetin
Honghua	MOL000098	quercetin
Honghua	MOL000098	quercetin
Honghua	MOL000098	quercetin
Honghua	MOL000098	quercetin
Honghua	MOL000098	quercetin
Honghua	MOL000098	quercetin
Honghua	MOL000098	quercetin
Honghua	MOL000098	quercetin
Honghua	MOL000098	quercetin
Honghua	MOL000098	quercetin
Honghua	MOL000098	quercetin
Honghua	MOL000098	quercetin
Honghua	MOL000098	quercetin
Honghua	MOL000098	quercetin
Honghua	MOL000098	quercetin
Honghua	MOL000098	quercetin
Honghua	MOL000098	quercetin
Honghua	MOL000098	quercetin
Honghua	MOL000098	quercetin
Honghua	MOL000098	quercetin
Honghua	MOL000098	quercetin
Honghua	MOL000098	quercetin
Honghua	MOL000098	quercetin
Honghua	MOL000098	quercetin
Honghua	MOL000098	quercetin
Honghua	MOL000098	quercetin
Honghua	MOL000098	quercetin
Honghua	MOL000098	quercetin
Honghua	MOL000098	quercetin
Honghua	MOL000098	quercetin

Honghua	MOL000098	quercetin
Honghua	MOL000098	quercetin
Honghua	MOL000098	quercetin
Honghua	MOL000098	quercetin
Honghua	MOL000098	quercetin
Honghua	MOL000098	quercetin
Honghua	MOL000098	quercetin
Honghua	MOL000098	quercetin
Honghua	MOL000098	quercetin
Honghua	MOL000098	quercetin
Honghua	MOL000098	quercetin
Honghua	MOL000098	quercetin
Honghua	MOL000098	quercetin
Honghua	MOL000098	quercetin
Honghua	MOL000098	quercetin
Honghua	MOL000098	quercetin
Honghua	MOL000098	quercetin
Honghua	MOL000098	quercetin
Honghua	MOL000098	quercetin
Honghua	MOL000098	quercetin
Honghua	MOL000098	quercetin
Honghua	MOL000098	quercetin
Honghua	MOL000098	quercetin
Honghua	MOL000098	quercetin
Honghua	MOL000098	quercetin
Honghua	MOL000098	quercetin
Honghua	MOL000098	quercetin
Honghua	MOL000098	quercetin
Honghua	MOL000098	quercetin
Honghua	MOL000098	quercetin
Honghua	MOL000098	quercetin
Honghua	MOL000098	quercetin
Honghua	MOL000098	quercetin
Honghua	MOL000098	quercetin
Honghua	MOL000098	quercetin
Honghua	MOL000098	quercetin
Honghua	MOL000098	quercetin
Honghua	MOL000098	quercetin
Honghua	MOL000098	quercetin
Honghua	MOL000098	quercetin
Honghua	MOL000098	quercetin
Honghua	MOL000098	quercetin
Honghua	MOL000098	quercetin
Honghua	MOL000098	quercetin
Honghua	MOL000098	quercetin
Honghua	MOL000098	quercetin
Honghua	MOL000098	quercetin
Honghua	MOL000098	quercetin
Honghua	MOL000098	quercetin
Honghua	MOL000098	quercetin
Honghua	MOL000098	quercetin
Honghua	MOL000098	quercetin
Honghua	MOL000098	quercetin
Honghua	MOL000098	quercetin
Honghua	MOL000098	quercetin
Honghua	MOL000098	quercetin
Honghua	MOL000098	quercetin
Honghua	MOL000098	quercetin

Honghua	MOL000098	quercetin
Honghua	MOL000098	quercetin
Honghua	MOL000098	quercetin
Honghua	MOL000098	quercetin
Honghua	MOL000098	quercetin
Honghua	MOL000098	quercetin
Honghua	MOL000098	quercetin
Honghua	MOL000098	quercetin
Honghua	MOL000098	quercetin
Honghua	MOL000098	quercetin
Honghua	MOL000098	quercetin
Honghua	MOL000098	quercetin
Honghua	MOL000098	quercetin
Honghua	MOL000098	quercetin
Honghua	MOL000098	quercetin
Honghua	MOL000098	quercetin
Honghua	MOL000098	quercetin
Honghua	MOL000098	quercetin
Honghua	MOL000098	quercetin
Honghua	MOL000098	quercetin
Honghua	MOL000098	quercetin
Honghua	MOL000098	quercetin
Honghua	MOL000098	quercetin
Honghua	MOL000098	quercetin
Honghua	MOL000098	quercetin
Honghua	MOL000098	quercetin
Honghua	MOL000098	quercetin
Honghua	MOL000098	quercetin
Honghua	MOL000098	quercetin
Honghua	MOL000098	quercetin
Honghua	MOL000098	quercetin
Honghua	MOL000098	quercetin
Honghua	MOL000098	quercetin
Honghua	MOL000098	quercetin
Honghua	MOL000098	quercetin
Honghua	MOL000098	quercetin
Honghua	MOL000098	quercetin
Honghua	MOL000098	quercetin
Honghua	MOL000098	quercetin
Honghua	MOL000098	quercetin
Honghua	MOL000098	quercetin
Honghua	MOL000098	quercetin
Honghua	MOL000098	quercetin
Honghua	MOL000098	quercetin
Honghua	MOL000098	quercetin
Honghua	MOL000098	quercetin
Honghua	MOL000098	quercetin
Honghua	MOL000098	quercetin
Honghua	MOL000098	quercetin
Honghua	MOL000098	quercetin
Honghua	MOL000098	quercetin
Honghua	MOL000098	quercetin
Honghua	MOL000098	quercetin
Honghua	MOL000098	quercetin
Honghua	MOL000098	quercetin
Honghua	MOL000098	quercetin
Honghua	MOL000098	quercetin
Honghua	MOL000098	quercetin

Shanyao	MOL001559	piperlonguminine
Shanyao	MOL001559	piperlonguminine
Shanyao	MOL001559	piperlonguminine
Shanyao	MOL001559	piperlonguminine
Shanyao	MOL001559	piperlonguminine
Shanyao	MOL001559	piperlonguminine
Shanyao	MOL001559	piperlonguminine
Shanyao	MOL001559	piperlonguminine
Shanyao	MOL001559	piperlonguminine
Shanyao	MOL001559	piperlonguminine
Shanyao	MOL001559	piperlonguminine
Shanyao	MOL001736	(-)-taxifolin
Shanyao	MOL001736	(-)-taxifolin
Shanyao	MOL001736	(-)-taxifolin
Shanyao	MOL001736	(-)-taxifolin
Shanyao	MOL000322	Kadsurenone
Shanyao	MOL000322	Kadsurenone
Shanyao	MOL000322	Kadsurenone
Shanyao	MOL000322	Kadsurenone
Shanyao	MOL000322	Kadsurenone
Shanyao	MOL000322	Kadsurenone
Shanyao	MOL000322	Kadsurenone
Shanyao	MOL000322	Kadsurenone
Shanyao	MOL000322	Kadsurenone
Shanyao	MOL000322	Kadsurenone
Shanyao	MOL000322	Kadsurenone
Shanyao	MOL000322	Kadsurenone
Shanyao	MOL000322	Kadsurenone
Shanyao	MOL000322	Kadsurenone
Shanyao	MOL000322	Kadsurenone
Shanyao	MOL000322	Kadsurenone
Shanyao	MOL000322	Kadsurenone
Shanyao	MOL000322	Kadsurenone
Shanyao	MOL000322	Kadsurenone
Shanyao	MOL000322	Kadsurenone
Shanyao	MOL000322	Kadsurenone
Shanyao	MOL000322	Kadsurenone
Shanyao	MOL000322	Kadsurenone
Shanyao	MOL000322	Kadsurenone
Shanyao	MOL000322	Kadsurenone
Shanyao	MOL000322	Kadsurenone
Shanyao	MOL000322	Kadsurenone
Shanyao	MOL005430	hancinone C
Shanyao	MOL005430	hancinone C
Shanyao	MOL005430	hancinone C
Shanyao	MOL005430	hancinone C
Shanyao	MOL005430	hancinone C
Shanyao	MOL005430	hancinone C
Shanyao	MOL005430	hancinone C
Shanyao	MOL005430	hancinone C
Shanyao	MOL005430	hancinone C
Shanyao	MOL005430	hancinone C
Shanyao	MOL005430	hancinone C
Shanyao	MOL005430	hancinone C
Shanyao	MOL005430	hancinone C
Shanyao	MOL005430	hancinone C
Shanyao	MOL005430	hancinone C
Shanyao	MOL005430	hancinone C

Shanyao	MOL005430	hancinone C
Shanyao	MOL005430	hancinone C
Shanyao	MOL005430	hancinone C
Shanyao	MOL005430	hancinone C
Shanyao	MOL005430	hancinone C
Shanyao	MOL005430	hancinone C
Shanyao	MOL005435	24-Methylcholest-5-enyl-3belta-O-gluco
Shanyao	MOL005438	campesterol
Shanyao	MOL005440	Isofucosterol
Shanyao	MOL005440	Isofucosterol
Shanyao	MOL005440	Isofucosterol
Shanyao	MOL005440	Isofucosterol
Shanyao	MOL005440	Isofucosterol
Shanyao	MOL005440	Isofucosterol
Shanyao	MOL005440	Isofucosterol
Shanyao	MOL005440	Isofucosterol
Shanyao	MOL005440	Isofucosterol
Shanyao	MOL005440	Isofucosterol
Shanyao	MOL005440	Isofucosterol
Shanyao	MOL005440	Isofucosterol
Shanyao	MOL000449	Stigmasterol
Shanyao	MOL000449	Stigmasterol
Shanyao	MOL000449	Stigmasterol
Shanyao	MOL000449	Stigmasterol
Shanyao	MOL000449	Stigmasterol
Shanyao	MOL000449	Stigmasterol
Shanyao	MOL000449	Stigmasterol
Shanyao	MOL000449	Stigmasterol
Shanyao	MOL000449	Stigmasterol
Shanyao	MOL000449	Stigmasterol
Shanyao	MOL000449	Stigmasterol
Shanyao	MOL000449	Stigmasterol
Shanyao	MOL000449	Stigmasterol
Shanyao	MOL000449	Stigmasterol
Shanyao	MOL000449	Stigmasterol
Shanyao	MOL000449	Stigmasterol
Shanyao	MOL000449	Stigmasterol
Shanyao	MOL000449	Stigmasterol
Shanyao	MOL000449	Stigmasterol
Shanyao	MOL000449	Stigmasterol
Shanyao	MOL000449	Stigmasterol
Shanyao	MOL000449	Stigmasterol
Shanyao	MOL000449	Stigmasterol
Shanyao	MOL000449	Stigmasterol
Shanyao	MOL000449	Stigmasterol
Shanyao	MOL000449	Stigmasterol
Shanyao	MOL000449	Stigmasterol
Shanyao	MOL000449	Stigmasterol
Shanyao	MOL000449	Stigmasterol
Shanyao	MOL000449	Stigmasterol
Shanyao	MOL000449	Stigmasterol
Shanyao	MOL005458	Dioscoreside C_qt
Shanyao	MOL005458	Dioscoreside C_qt
Shanyao	MOL000546	diosgenin
Shanyao	MOL000546	diosgenin
Shanyao	MOL000546	diosgenin
Shanyao	MOL000546	diosgenin
Shanyao	MOL000546	diosgenin

Shanyao	MOL000546	diosgenin
Shanyao	MOL000546	diosgenin
Shanyao	MOL000546	diosgenin
Shanyao	MOL000546	diosgenin
Shanyao	MOL000546	diosgenin
Shanyao	MOL000546	diosgenin
Shanyao	MOL000546	diosgenin
Shanyao	MOL000546	diosgenin
Shanyao	MOL000546	diosgenin
Shanyao	MOL000546	diosgenin
Shanyao	MOL005465	AIDS180907
Shanyao	MOL005465	AIDS180907
Shanyao	MOL005465	AIDS180907
Shanyao	MOL005465	AIDS180907
Shanyao	MOL005465	AIDS180907
Shanyao	MOL005465	AIDS180907
Shanyao	MOL005465	AIDS180907
Shanyao	MOL005465	AIDS180907
Shanyao	MOL005465	AIDS180907
Shanyao	MOL005465	AIDS180907
Shanyao	MOL005465	AIDS180907
Shanyao	MOL005465	AIDS180907
Shanyao	MOL005465	AIDS180907
Shanyao	MOL000953	CLR
Shanyao	MOL000953	CLR
Shanyao	MOL000953	CLR
Shanyao	MOL000953	CLR
Shanyurou	MOL001494	Mandenol
Shanyurou	MOL001494	Mandenol
Shanyurou	MOL001494	Mandenol
Shanyurou	MOL001495	Ethyl linolenate
Shanyurou	MOL001495	Ethyl linolenate
Shanyurou	MOL001771	poriferast-5-en-3beta-ol
Shanyurou	MOL001771	poriferast-5-en-3beta-ol
Shanyurou	MOL002879	Diop
Shanyurou	MOL002879	Diop
Shanyurou	MOL002879	Diop
Shanyurou	MOL002883	Ethyl oleate (NF)
Shanyurou	MOL003137	Leucanthoside
Shanyurou	MOL003137	Leucanthoside
Shanyurou	MOL003137	Leucanthoside
Shanyurou	MOL000358	beta-sitosterol
Shanyurou	MOL000358	beta-sitosterol
Shanyurou	MOL000358	beta-sitosterol
Shanyurou	MOL000358	beta-sitosterol
Shanyurou	MOL000358	beta-sitosterol
Shanyurou	MOL000358	beta-sitosterol
Shanyurou	MOL000358	beta-sitosterol
Shanyurou	MOL000358	beta-sitosterol
Shanyurou	MOL000358	beta-sitosterol
Shanyurou	MOL000358	beta-sitosterol
Shanyurou	MOL000358	beta-sitosterol
Shanyurou	MOL000358	beta-sitosterol
Shanyurou	MOL000358	beta-sitosterol
Shanyurou	MOL000358	beta-sitosterol
Shanyurou	MOL000358	beta-sitosterol
Shanyurou	MOL000358	beta-sitosterol
Shanyurou	MOL000358	beta-sitosterol

Shanyurou	MOL000358	beta-sitosterol
Shanyurou	MOL000358	beta-sitosterol
Shanyurou	MOL000358	beta-sitosterol
Shanyurou	MOL000358	beta-sitosterol
Shanyurou	MOL000358	beta-sitosterol
Shanyurou	MOL000358	beta-sitosterol
Shanyurou	MOL000358	beta-sitosterol
Shanyurou	MOL000358	beta-sitosterol
Shanyurou	MOL000358	beta-sitosterol
Shanyurou	MOL000358	beta-sitosterol
Shanyurou	MOL000358	beta-sitosterol
Shanyurou	MOL000358	beta-sitosterol
Shanyurou	MOL000358	beta-sitosterol
Shanyurou	MOL000358	beta-sitosterol
Shanyurou	MOL000358	beta-sitosterol
Shanyurou	MOL000358	beta-sitosterol
Shanyurou	MOL000358	beta-sitosterol
Shanyurou	MOL000358	beta-sitosterol
Shanyurou	MOL000358	beta-sitosterol
Shanyurou	MOL000358	beta-sitosterol
Shanyurou	MOL000358	beta-sitosterol
Shanyurou	MOL000359	sitosterol
Shanyurou	MOL000359	sitosterol
Shanyurou	MOL000359	sitosterol
Shanyurou	MOL000449	Stigmasterol
Shanyurou	MOL000449	Stigmasterol
Shanyurou	MOL000449	Stigmasterol
Shanyurou	MOL000449	Stigmasterol
Shanyurou	MOL000449	Stigmasterol
Shanyurou	MOL000449	Stigmasterol
Shanyurou	MOL000449	Stigmasterol
Shanyurou	MOL000449	Stigmasterol
Shanyurou	MOL000449	Stigmasterol
Shanyurou	MOL000449	Stigmasterol
Shanyurou	MOL000449	Stigmasterol
Shanyurou	MOL000449	Stigmasterol
Shanyurou	MOL000449	Stigmasterol
Shanyurou	MOL000449	Stigmasterol
Shanyurou	MOL000449	Stigmasterol
Shanyurou	MOL000449	Stigmasterol
Shanyurou	MOL000449	Stigmasterol
Shanyurou	MOL000449	Stigmasterol
Shanyurou	MOL000449	Stigmasterol
Shanyurou	MOL000449	Stigmasterol
Shanyurou	MOL000449	Stigmasterol
Shanyurou	MOL000449	Stigmasterol
Shanyurou	MOL000449	Stigmasterol
Shanyurou	MOL000449	Stigmasterol
Shanyurou	MOL000449	Stigmasterol
Shanyurou	MOL000449	Stigmasterol
Shanyurou	MOL000449	Stigmasterol
Shanyurou	MOL000449	Stigmasterol
Shanyurou	MOL000449	Stigmasterol
Shanyurou	MOL000449	Stigmasterol
Shanyurou	MOL000449	Stigmasterol
Shanyurou	MOL005481	2,6,10,14,18-pentamethylicosa-2,6,10,14,1
Shanyurou	MOL005503	Cornudentanone
Shanyurou	MOL005503	Cornudentanone

Shanyurou	MOL005503	Cornudentanone
Shanyurou	MOL005530	Hydroxygenkwanin
Shanyurou	MOL005530	Hydroxygenkwanin
Shanyurou	MOL005530	Hydroxygenkwanin
Shanyurou	MOL005530	Hydroxygenkwanin
Shanyurou	MOL005530	Hydroxygenkwanin
Shanyurou	MOL005530	Hydroxygenkwanin
Shanyurou	MOL005530	Hydroxygenkwanin
Shanyurou	MOL005530	Hydroxygenkwanin
Shanyurou	MOL005530	Hydroxygenkwanin
Shanyurou	MOL005530	Hydroxygenkwanin
Shanyurou	MOL005531	Telocinobufagin
Shanyurou	MOL005531	Telocinobufagin
Shanyurou	MOL008457	Tetrahydroalstonine
Shanyurou	MOL008457	Tetrahydroalstonine
Shanyurou	MOL008457	Tetrahydroalstonine
Shanyurou	MOL008457	Tetrahydroalstonine
Shanyurou	MOL008457	Tetrahydroalstonine
Shanyurou	MOL008457	Tetrahydroalstonine
Shanyurou	MOL008457	Tetrahydroalstonine
Shanyurou	MOL008457	Tetrahydroalstonine
Shanyurou	MOL008457	Tetrahydroalstonine
Shanyurou	MOL008457	Tetrahydroalstonine
Shanyurou	MOL008457	Tetrahydroalstonine
Shanyurou	MOL008457	Tetrahydroalstonine
Shanyurou	MOL008457	Tetrahydroalstonine
Shanyurou	MOL008457	Tetrahydroalstonine
Shanyurou	MOL008457	Tetrahydroalstonine
Shanyurou	MOL008457	Tetrahydroalstonine
Shanyurou	MOL008457	Tetrahydroalstonine
Shanyurou	MOL008457	Tetrahydroalstonine
Shanyurou	MOL008457	Tetrahydroalstonine
Shanyurou	MOL008457	Tetrahydroalstonine
Shanyurou	MOL008457	Tetrahydroalstonine
Shanyurou	MOL008457	Tetrahydroalstonine
Shanyurou	MOL008457	Tetrahydroalstonine
Shanyurou	MOL008457	Tetrahydroalstonine
Shanyurou	MOL008457	Tetrahydroalstonine
Shanyurou	MOL008457	Tetrahydroalstonine
Shanyurou	MOL008457	Tetrahydroalstonine
Shanyurou	MOL008457	Tetrahydroalstonine
Shanzha	MOL000354	isorhamnetin
Shanzha	MOL000354	isorhamnetin
Shanzha	MOL000354	isorhamnetin
Shanzha	MOL000354	isorhamnetin
Shanzha	MOL000354	isorhamnetin
Shanzha	MOL000354	isorhamnetin
Shanzha	MOL000354	isorhamnetin
Shanzha	MOL000354	isorhamnetin
Shanzha	MOL000354	isorhamnetin
Shanzha	MOL000354	isorhamnetin
Shanzha	MOL000354	isorhamnetin
Shanzha	MOL000354	isorhamnetin
Shanzha	MOL000354	isorhamnetin
Shanzha	MOL000354	isorhamnetin
Shanzha	MOL000354	isorhamnetin
Shanzha	MOL000354	isorhamnetin
Shanzha	MOL000354	isorhamnetin

Shanzha	MOL000354	isorhamnetin
Shanzha	MOL000354	isorhamnetin
Shanzha	MOL000354	isorhamnetin
Shanzha	MOL000354	isorhamnetin
Shanzha	MOL000354	isorhamnetin
Shanzha	MOL000354	isorhamnetin
Shanzha	MOL000354	isorhamnetin
Shanzha	MOL000354	isorhamnetin
Shanzha	MOL000354	isorhamnetin
Shanzha	MOL000354	isorhamnetin
Shanzha	MOL000354	isorhamnetin
Shanzha	MOL000354	isorhamnetin
Shanzha	MOL000354	isorhamnetin
Shanzha	MOL000354	isorhamnetin
Shanzha	MOL000354	isorhamnetin
Shanzha	MOL000354	isorhamnetin
Shanzha	MOL000354	isorhamnetin
Shanzha	MOL000354	isorhamnetin
Shanzha	MOL000354	isorhamnetin
Shanzha	MOL000354	isorhamnetin
Shanzha	MOL000359	sitosterol
Shanzha	MOL000359	sitosterol
Shanzha	MOL000359	sitosterol
Shanzha	MOL000422	kaempferol
Shanzha	MOL000422	kaempferol
Shanzha	MOL000422	kaempferol
Shanzha	MOL000422	kaempferol
Shanzha	MOL000422	kaempferol
Shanzha	MOL000422	kaempferol
Shanzha	MOL000422	kaempferol
Shanzha	MOL000422	kaempferol
Shanzha	MOL000422	kaempferol
Shanzha	MOL000422	kaempferol
Shanzha	MOL000422	kaempferol
Shanzha	MOL000422	kaempferol
Shanzha	MOL000422	kaempferol
Shanzha	MOL000422	kaempferol
Shanzha	MOL000422	kaempferol
Shanzha	MOL000422	kaempferol
Shanzha	MOL000422	kaempferol
Shanzha	MOL000422	kaempferol
Shanzha	MOL000422	kaempferol
Shanzha	MOL000422	kaempferol
Shanzha	MOL000422	kaempferol
Shanzha	MOL000422	kaempferol
Shanzha	MOL000422	kaempferol
Shanzha	MOL000422	kaempferol
Shanzha	MOL000422	kaempferol
Shanzha	MOL000422	kaempferol
Shanzha	MOL000422	kaempferol
Shanzha	MOL000422	kaempferol
Shanzha	MOL000422	kaempferol
Shanzha	MOL000422	kaempferol
Shanzha	MOL000422	kaempferol
Shanzha	MOL000422	kaempferol
Shanzha	MOL000422	kaempferol
Shanzha	MOL000422	kaempferol

Shanzha	MOL000422	kaempferol
Shanzha	MOL000422	kaempferol
Shanzha	MOL000422	kaempferol
Shanzha	MOL000422	kaempferol
Shanzha	MOL000422	kaempferol
Shanzha	MOL000422	kaempferol
Shanzha	MOL000422	kaempferol
Shanzha	MOL000422	kaempferol
Shanzha	MOL000422	kaempferol
Shanzha	MOL000422	kaempferol
Shanzha	MOL000422	kaempferol
Shanzha	MOL000422	kaempferol
Shanzha	MOL000422	kaempferol
Shanzha	MOL000422	kaempferol
Shanzha	MOL000422	kaempferol
Shanzha	MOL000422	kaempferol
Shanzha	MOL000422	kaempferol
Shanzha	MOL000422	kaempferol
Shanzha	MOL000422	kaempferol
Shanzha	MOL000422	kaempferol
Shanzha	MOL000422	kaempferol
Shanzha	MOL000422	kaempferol
Shanzha	MOL000422	kaempferol
Shanzha	MOL000422	kaempferol
Shanzha	MOL000422	kaempferol
Shanzha	MOL000422	kaempferol
Shanzha	MOL000422	kaempferol
Shanzha	MOL000449	Stigmasterol
Shanzha	MOL000449	Stigmasterol
Shanzha	MOL000449	Stigmasterol
Shanzha	MOL000449	Stigmasterol
Shanzha	MOL000449	Stigmasterol
Shanzha	MOL000449	Stigmasterol
Shanzha	MOL000449	Stigmasterol
Shanzha	MOL000449	Stigmasterol
Shanzha	MOL000449	Stigmasterol
Shanzha	MOL000449	Stigmasterol
Shanzha	MOL000449	Stigmasterol
Shanzha	MOL000449	Stigmasterol
Shanzha	MOL000449	Stigmasterol
Shanzha	MOL000449	Stigmasterol
Shanzha	MOL000449	Stigmasterol
Shanzha	MOL000449	Stigmasterol
Shanzha	MOL000449	Stigmasterol
Shanzha	MOL000449	Stigmasterol
Shanzha	MOL000449	Stigmasterol
Shanzha	MOL000449	Stigmasterol
Shanzha	MOL000449	Stigmasterol
Shanzha	MOL000449	Stigmasterol
Shanzha	MOL000449	Stigmasterol
Shanzha	MOL000449	Stigmasterol
Shanzha	MOL000449	Stigmasterol
Shanzha	MOL000449	Stigmasterol
Shanzha	MOL000449	Stigmasterol
Shanzha	MOL000449	Stigmasterol
Shanzha	MOL000449	Stigmasterol
Shanzha	MOL000449	Stigmasterol
Shanzha	MOL000449	Stigmasterol

Shanzha	MOL000073	ent-Epicatechin
Shanzha	MOL000073	ent-Epicatechin
Shanzha	MOL000073	ent-Epicatechin
Shanzha	MOL000073	ent-Epicatechin
Shanzha	MOL000073	ent-Epicatechin
Shanzha	MOL000073	ent-Epicatechin
Shanzha	MOL000098	quercetin
Shanzha	MOL000098	quercetin
Shanzha	MOL000098	quercetin
Shanzha	MOL000098	quercetin
Shanzha	MOL000098	quercetin
Shanzha	MOL000098	quercetin
Shanzha	MOL000098	quercetin
Shanzha	MOL000098	quercetin
Shanzha	MOL000098	quercetin
Shanzha	MOL000098	quercetin
Shanzha	MOL000098	quercetin
Shanzha	MOL000098	quercetin
Shanzha	MOL000098	quercetin
Shanzha	MOL000098	quercetin
Shanzha	MOL000098	quercetin
Shanzha	MOL000098	quercetin
Shanzha	MOL000098	quercetin
Shanzha	MOL000098	quercetin
Shanzha	MOL000098	quercetin
Shanzha	MOL000098	quercetin
Shanzha	MOL000098	quercetin
Shanzha	MOL000098	quercetin
Shanzha	MOL000098	quercetin
Shanzha	MOL000098	quercetin
Shanzha	MOL000098	quercetin
Shanzha	MOL000098	quercetin
Shanzha	MOL000098	quercetin
Shanzha	MOL000098	quercetin
Shanzha	MOL000098	quercetin
Shanzha	MOL000098	quercetin
Shanzha	MOL000098	quercetin
Shanzha	MOL000098	quercetin
Shanzha	MOL000098	quercetin
Shanzha	MOL000098	quercetin
Shanzha	MOL000098	quercetin
Shanzha	MOL000098	quercetin
Shanzha	MOL000098	quercetin
Shanzha	MOL000098	quercetin
Shanzha	MOL000098	quercetin
Shanzha	MOL000098	quercetin
Shanzha	MOL000098	quercetin
Shanzha	MOL000098	quercetin
Shanzha	MOL000098	quercetin
Shanzha	MOL000098	quercetin
Shanzha	MOL000098	quercetin
Shanzha	MOL000098	quercetin
Shanzha	MOL000098	quercetin
Shanzha	MOL000098	quercetin
Shanzha	MOL000098	quercetin
Shanzha	MOL000098	quercetin
Shanzha	MOL000098	quercetin
Shanzha	MOL000098	quercetin

Shanzha	MOL000098	quercetin
Shanzha	MOL000098	quercetin
Shanzha	MOL000098	quercetin
Shanzha	MOL000098	quercetin
Shanzha	MOL000098	quercetin
Shanzha	MOL000098	quercetin
Shanzha	MOL000098	quercetin
Shanzha	MOL000098	quercetin
Shanzha	MOL000098	quercetin
Shanzha	MOL000098	quercetin
Shanzha	MOL000098	quercetin
Shanzha	MOL000098	quercetin
Shanzha	MOL000098	quercetin
Shanzha	MOL000098	quercetin
Shanzha	MOL000098	quercetin
Shanzha	MOL000098	quercetin
Shanzha	MOL000098	quercetin
Shanzha	MOL000098	quercetin
Shanzha	MOL000098	quercetin
Shanzha	MOL000098	quercetin
Shanzha	MOL000098	quercetin
Shanzha	MOL000098	quercetin
Shanzha	MOL000098	quercetin
Shanzha	MOL000098	quercetin
Shanzha	MOL000098	quercetin
Shanzha	MOL000098	quercetin
Shanzha	MOL000098	quercetin
Shanzha	MOL000098	quercetin
Shanzha	MOL000098	quercetin
Shanzha	MOL000098	quercetin
Shanzha	MOL000098	quercetin
Shanzha	MOL000098	quercetin
Shanzha	MOL000098	quercetin
Shanzha	MOL000098	quercetin
Shanzha	MOL000098	quercetin
Shanzha	MOL000098	quercetin
Shanzha	MOL000098	quercetin
Shanzha	MOL000098	quercetin
Shanzha	MOL000098	quercetin
Shanzha	MOL000098	quercetin
Shanzha	MOL000098	quercetin
Shanzha	MOL000098	quercetin
Shanzha	MOL000098	quercetin
Shanzha	MOL000098	quercetin
Shanzha	MOL000098	quercetin
Shanzha	MOL000098	quercetin
Shanzha	MOL000098	quercetin
Shanzha	MOL000098	quercetin
Shanzha	MOL000098	quercetin
Shanzha	MOL000098	quercetin
Shanzha	MOL000098	quercetin
Shanzha	MOL000098	quercetin
Shanzha	MOL000098	quercetin
Shanzha	MOL000098	quercetin
Shanzha	MOL000098	quercetin
Shanzha	MOL000098	quercetin
Shanzha	MOL000098	quercetin
Shanzha	MOL000098	quercetin

Shanzha	MOL000098	quercetin
Shanzha	MOL000098	quercetin
Shanzha	MOL000098	quercetin
Shanzha	MOL000098	quercetin
Shanzha	MOL000098	quercetin
Shanzha	MOL000098	quercetin
Shanzha	MOL000098	quercetin
Shanzha	MOL000098	quercetin
Shanzha	MOL000098	quercetin
Shanzha	MOL000098	quercetin
Shanzha	MOL000098	quercetin
Shanzha	MOL000098	quercetin
Shanzha	MOL000098	quercetin
Shanzha	MOL000098	quercetin
Shanzha	MOL000098	quercetin
Shanzha	MOL000098	quercetin
Shanzha	MOL000098	quercetin
Shanzha	MOL000098	quercetin
Shanzha	MOL000098	quercetin
Shanzha	MOL000098	quercetin
Shanzha	MOL000098	quercetin
Shanzha	MOL000098	quercetin
Shanzha	MOL000098	quercetin
Shanzha	MOL000098	quercetin
Shanzha	MOL000098	quercetin
Shanzha	MOL000098	quercetin
Shanzha	MOL000098	quercetin
Shanzha	MOL000098	quercetin
Shanzha	MOL000098	quercetin
Shanzha	MOL000098	quercetin
Shanzha	MOL000098	quercetin
Shanzha	MOL000098	quercetin
Shanzha	MOL000098	quercetin
Shanzha	MOL000098	quercetin
Shanzha	MOL000098	quercetin
Shanzha	MOL000098	quercetin
Shanzha	MOL000098	quercetin
Shanzha	MOL000098	quercetin
Shudihuang	MOL000359	sitosterol
Shudihuang	MOL000359	sitosterol
Shudihuang	MOL000359	sitosterol
Shudihuang	MOL000449	Stigmasterol
Shudihuang	MOL000449	Stigmasterol
Shudihuang	MOL000449	Stigmasterol
Shudihuang	MOL000449	Stigmasterol
Shudihuang	MOL000449	Stigmasterol
Shudihuang	MOL000449	Stigmasterol
Shudihuang	MOL000449	Stigmasterol
Shudihuang	MOL000449	Stigmasterol
Shudihuang	MOL000449	Stigmasterol
Shudihuang	MOL000449	Stigmasterol
Shudihuang	MOL000449	Stigmasterol
Shudihuang	MOL000449	Stigmasterol
Shudihuang	MOL000449	Stigmasterol
Shudihuang	MOL000449	Stigmasterol
Shudihuang	MOL000449	Stigmasterol
Shudihuang	MOL000449	Stigmasterol
Shudihuang	MOL000449	Stigmasterol

Shudihuang	MOL000449	Stigmasterol
Shudihuang	MOL000449	Stigmasterol
Shudihuang	MOL000449	Stigmasterol
Shudihuang	MOL000449	Stigmasterol
Shudihuang	MOL000449	Stigmasterol
Shudihuang	MOL000449	Stigmasterol
Shudihuang	MOL000449	Stigmasterol
Shudihuang	MOL000449	Stigmasterol
Shudihuang	MOL000449	Stigmasterol
Shudihuang	MOL000449	Stigmasterol
Shudihuang	MOL000449	Stigmasterol
Shudihuang	MOL000449	Stigmasterol
Shudihuang	MOL000449	Stigmasterol
Shudihuang	MOL000449	Stigmasterol
Tusizi	MOL001558	sesamin
Tusizi	MOL001558	sesamin
Tusizi	MOL001558	sesamin
Tusizi	MOL001558	sesamin
Tusizi	MOL001558	sesamin
Tusizi	MOL001558	sesamin
Tusizi	MOL001558	sesamin
Tusizi	MOL001558	sesamin
Tusizi	MOL001558	sesamin
Tusizi	MOL001558	sesamin
Tusizi	MOL001558	sesamin
Tusizi	MOL001558	sesamin
Tusizi	MOL001558	sesamin
Tusizi	MOL001558	sesamin
Tusizi	MOL001558	sesamin
Tusizi	MOL001558	sesamin
Tusizi	MOL001558	sesamin
Tusizi	MOL001558	sesamin
Tusizi	MOL001558	sesamin
Tusizi	MOL001558	sesamin
Tusizi	MOL001558	sesamin
Tusizi	MOL001558	sesamin
Tusizi	MOL001558	sesamin
Tusizi	MOL000184	NSC63551
Tusizi	MOL000354	isorhamnetin
Tusizi	MOL000354	isorhamnetin
Tusizi	MOL000354	isorhamnetin
Tusizi	MOL000354	isorhamnetin
Tusizi	MOL000354	isorhamnetin
Tusizi	MOL000354	isorhamnetin
Tusizi	MOL000354	isorhamnetin
Tusizi	MOL000354	isorhamnetin
Tusizi	MOL000354	isorhamnetin
Tusizi	MOL000354	isorhamnetin
Tusizi	MOL000354	isorhamnetin
Tusizi	MOL000354	isorhamnetin
Tusizi	MOL000354	isorhamnetin
Tusizi	MOL000354	isorhamnetin
Tusizi	MOL000354	isorhamnetin
Tusizi	MOL000354	isorhamnetin
Tusizi	MOL000354	isorhamnetin
Tusizi	MOL000354	isorhamnetin
Tusizi	MOL000354	isorhamnetin
Tusizi	MOL000354	isorhamnetin

Tusizi	MOL000354	isorhamnetin
Tusizi	MOL000354	isorhamnetin
Tusizi	MOL000354	isorhamnetin
Tusizi	MOL000354	isorhamnetin
Tusizi	MOL000354	isorhamnetin
Tusizi	MOL000354	isorhamnetin
Tusizi	MOL000354	isorhamnetin
Tusizi	MOL000354	isorhamnetin
Tusizi	MOL000354	isorhamnetin
Tusizi	MOL000354	isorhamnetin
Tusizi	MOL000354	isorhamnetin
Tusizi	MOL000354	isorhamnetin
Tusizi	MOL000354	isorhamnetin
Tusizi	MOL000354	isorhamnetin
Tusizi	MOL000354	isorhamnetin
Tusizi	MOL000354	isorhamnetin
Tusizi	MOL000354	isorhamnetin
Tusizi	MOL000358	beta-sitosterol
Tusizi	MOL000358	beta-sitosterol
Tusizi	MOL000358	beta-sitosterol
Tusizi	MOL000358	beta-sitosterol
Tusizi	MOL000358	beta-sitosterol
Tusizi	MOL000358	beta-sitosterol
Tusizi	MOL000358	beta-sitosterol
Tusizi	MOL000358	beta-sitosterol
Tusizi	MOL000358	beta-sitosterol
Tusizi	MOL000358	beta-sitosterol
Tusizi	MOL000358	beta-sitosterol
Tusizi	MOL000358	beta-sitosterol
Tusizi	MOL000358	beta-sitosterol
Tusizi	MOL000358	beta-sitosterol
Tusizi	MOL000358	beta-sitosterol
Tusizi	MOL000358	beta-sitosterol
Tusizi	MOL000358	beta-sitosterol
Tusizi	MOL000358	beta-sitosterol
Tusizi	MOL000358	beta-sitosterol
Tusizi	MOL000358	beta-sitosterol
Tusizi	MOL000358	beta-sitosterol
Tusizi	MOL000358	beta-sitosterol
Tusizi	MOL000358	beta-sitosterol
Tusizi	MOL000358	beta-sitosterol
Tusizi	MOL000358	beta-sitosterol
Tusizi	MOL000358	beta-sitosterol
Tusizi	MOL000358	beta-sitosterol
Tusizi	MOL000358	beta-sitosterol
Tusizi	MOL000358	beta-sitosterol
Tusizi	MOL000358	beta-sitosterol
Tusizi	MOL000358	beta-sitosterol
Tusizi	MOL000358	beta-sitosterol
Tusizi	MOL000358	beta-sitosterol
Tusizi	MOL000358	beta-sitosterol
Tusizi	MOL000358	beta-sitosterol
Tusizi	MOL000358	beta-sitosterol
Tusizi	MOL000358	beta-sitosterol
Tusizi	MOL000358	beta-sitosterol
Tusizi	MOL000422	kaempferol
Tusizi	MOL000422	kaempferol
Tusizi	MOL000422	kaempferol

Tusizi	MOL000422	kaempferol
Tusizi	MOL000422	kaempferol
Tusizi	MOL000422	kaempferol
Tusizi	MOL000422	kaempferol
Tusizi	MOL000422	kaempferol
Tusizi	MOL000422	kaempferol
Tusizi	MOL000422	kaempferol
Tusizi	MOL000422	kaempferol
Tusizi	MOL000422	kaempferol
Tusizi	MOL000422	kaempferol
Tusizi	MOL000422	kaempferol
Tusizi	MOL000422	kaempferol
Tusizi	MOL000422	kaempferol
Tusizi	MOL000422	kaempferol
Tusizi	MOL000422	kaempferol
Tusizi	MOL000422	kaempferol
Tusizi	MOL000422	kaempferol
Tusizi	MOL000422	kaempferol
Tusizi	MOL000422	kaempferol
Tusizi	MOL000422	kaempferol
Tusizi	MOL000422	kaempferol
Tusizi	MOL000422	kaempferol
Tusizi	MOL000422	kaempferol
Tusizi	MOL000422	kaempferol
Tusizi	MOL000422	kaempferol
Tusizi	MOL000422	kaempferol
Tusizi	MOL000422	kaempferol
Tusizi	MOL000422	kaempferol
Tusizi	MOL000422	kaempferol
Tusizi	MOL000422	kaempferol
Tusizi	MOL000422	kaempferol
Tusizi	MOL000422	kaempferol
Tusizi	MOL000422	kaempferol
Tusizi	MOL000422	kaempferol
Tusizi	MOL000422	kaempferol
Tusizi	MOL000422	kaempferol
Tusizi	MOL000422	kaempferol
Tusizi	MOL000422	kaempferol
Tusizi	MOL000422	kaempferol
Tusizi	MOL000422	kaempferol
Tusizi	MOL000422	kaempferol
Tusizi	MOL000422	kaempferol
Tusizi	MOL000422	kaempferol
Tusizi	MOL000422	kaempferol
Tusizi	MOL000422	kaempferol
Tusizi	MOL000422	kaempferol
Tusizi	MOL000422	kaempferol
Tusizi	MOL000422	kaempferol
Tusizi	MOL000422	kaempferol
Tusizi	MOL000422	kaempferol
Tusizi	MOL000422	kaempferol
Tusizi	MOL000422	kaempferol
Tusizi	MOL000422	kaempferol
Tusizi	MOL000422	kaempferol
Tusizi	MOL000422	kaempferol
Tusizi	MOL000422	kaempferol
Tusizi	MOL000422	kaempferol
Tusizi	MOL000422	kaempferol

Tusizi	MOL000422	kaempferol
Tusizi	MOL005043	campest-5-en-3beta-ol
Tusizi	MOL005440	Isofucosterol
Tusizi	MOL005440	Isofucosterol
Tusizi	MOL005440	Isofucosterol
Tusizi	MOL005440	Isofucosterol
Tusizi	MOL005440	Isofucosterol
Tusizi	MOL005440	Isofucosterol
Tusizi	MOL005440	Isofucosterol
Tusizi	MOL005440	Isofucosterol
Tusizi	MOL005440	Isofucosterol
Tusizi	MOL005440	Isofucosterol
Tusizi	MOL005440	Isofucosterol
Tusizi	MOL005440	Isofucosterol
Tusizi	MOL005944	matrine
Tusizi	MOL005944	matrine
Tusizi	MOL005944	matrine
Tusizi	MOL005944	matrine
Tusizi	MOL005944	matrine
Tusizi	MOL005944	matrine
Tusizi	MOL005944	matrine
Tusizi	MOL005944	matrine
Tusizi	MOL005944	matrine
Tusizi	MOL005944	matrine
Tusizi	MOL000953	CLR
Tusizi	MOL000953	CLR
Tusizi	MOL000953	CLR
Tusizi	MOL000953	CLR
Tusizi	MOL000098	quercetin
Tusizi	MOL000098	quercetin
Tusizi	MOL000098	quercetin
Tusizi	MOL000098	quercetin
Tusizi	MOL000098	quercetin
Tusizi	MOL000098	quercetin
Tusizi	MOL000098	quercetin
Tusizi	MOL000098	quercetin
Tusizi	MOL000098	quercetin
Tusizi	MOL000098	quercetin
Tusizi	MOL000098	quercetin
Tusizi	MOL000098	quercetin
Tusizi	MOL000098	quercetin
Tusizi	MOL000098	quercetin
Tusizi	MOL000098	quercetin
Tusizi	MOL000098	quercetin
Tusizi	MOL000098	quercetin
Tusizi	MOL000098	quercetin
Tusizi	MOL000098	quercetin
Tusizi	MOL000098	quercetin
Tusizi	MOL000098	quercetin
Tusizi	MOL000098	quercetin
Tusizi	MOL000098	quercetin
Tusizi	MOL000098	quercetin
Tusizi	MOL000098	quercetin
Tusizi	MOL000098	quercetin
Tusizi	MOL000098	quercetin
Tusizi	MOL000098	quercetin
Tusizi	MOL000098	quercetin
Tusizi	MOL000098	quercetin

Tusizi	MOL000098	quercetin
Tusizi	MOL000098	quercetin
Tusizi	MOL000098	quercetin
Tusizi	MOL000098	quercetin
Tusizi	MOL000098	quercetin
Tusizi	MOL000098	quercetin
Tusizi	MOL000098	quercetin
Tusizi	MOL000098	quercetin
Tusizi	MOL000098	quercetin
Tusizi	MOL000098	quercetin
Tusizi	MOL000098	quercetin
Tusizi	MOL000098	quercetin
Tusizi	MOL000098	quercetin
Tusizi	MOL000098	quercetin
Tusizi	MOL000098	quercetin
Tusizi	MOL000098	quercetin
Tusizi	MOL000098	quercetin
Tusizi	MOL000098	quercetin
Tusizi	MOL000098	quercetin
Tusizi	MOL000098	quercetin
Tusizi	MOL000098	quercetin
Tusizi	MOL000098	quercetin
Tusizi	MOL000098	quercetin
Tusizi	MOL000098	quercetin
Tusizi	MOL000098	quercetin
Tusizi	MOL000098	quercetin
Tusizi	MOL000098	quercetin
Tusizi	MOL000098	quercetin
Tusizi	MOL000098	quercetin
Tusizi	MOL000098	quercetin
Tusizi	MOL000098	quercetin
Tusizi	MOL000098	quercetin
Tusizi	MOL000098	quercetin
Tusizi	MOL000098	quercetin
Tusizi	MOL000098	quercetin
Tusizi	MOL000098	quercetin
Tusizi	MOL000098	quercetin
Tusizi	MOL000098	quercetin
Tusizi	MOL000098	quercetin
Tusizi	MOL000098	quercetin
Tusizi	MOL000098	quercetin
Tusizi	MOL000098	quercetin
Tusizi	MOL000098	quercetin
Tusizi	MOL000098	quercetin
Tusizi	MOL000098	quercetin
Tusizi	MOL000098	quercetin
Tusizi	MOL000098	quercetin
Tusizi	MOL000098	quercetin
Tusizi	MOL000098	quercetin
Tusizi	MOL000098	quercetin
Tusizi	MOL000098	quercetin
Tusizi	MOL000098	quercetin
Tusizi	MOL000098	quercetin
Tusizi	MOL000098	quercetin
Tusizi	MOL000098	quercetin
Tusizi	MOL000098	quercetin
Tusizi	MOL000098	quercetin
Tusizi	MOL000098	quercetin

Tusizi	MOL000098	quercetin
Tusizi	MOL000098	quercetin
Tusizi	MOL000098	quercetin
Tusizi	MOL000098	quercetin
Tusizi	MOL000098	quercetin
Tusizi	MOL000098	quercetin
Tusizi	MOL000098	quercetin
Tusizi	MOL000098	quercetin
Tusizi	MOL000098	quercetin
Tusizi	MOL000098	quercetin
Tusizi	MOL000098	quercetin
Tusizi	MOL000098	quercetin
Tusizi	MOL000098	quercetin
Tusizi	MOL000098	quercetin
Tusizi	MOL000098	quercetin
Tusizi	MOL000098	quercetin
Tusizi	MOL000098	quercetin
Tusizi	MOL000098	quercetin
Tusizi	MOL000098	quercetin
Tusizi	MOL000098	quercetin
Tusizi	MOL000098	quercetin
Tusizi	MOL000098	quercetin
Tusizi	MOL000098	quercetin
Tusizi	MOL000098	quercetin
Tusizi	MOL000098	quercetin
Tusizi	MOL000098	quercetin
Tusizi	MOL000098	quercetin
Tusizi	MOL000098	quercetin
Tusizi	MOL000098	quercetin
Tusizi	MOL000098	quercetin
Tusizi	MOL000098	quercetin
Tusizi	MOL000098	quercetin
Tusizi	MOL000098	quercetin
Tusizi	MOL000098	quercetin
Tusizi	MOL000098	quercetin
Tusizi	MOL000098	quercetin
Tusizi	MOL000098	quercetin
Tusizi	MOL000098	quercetin
Tusizi	MOL000098	quercetin
Tusizi	MOL000098	quercetin
Tusizi	MOL000098	quercetin
Tusizi	MOL000098	quercetin
Tusizi	MOL000098	quercetin
Tusizi	MOL000098	quercetin
Tusizi	MOL000098	quercetin
Tusizi	MOL000098	quercetin
Tusizi	MOL000098	quercetin
Tusizi	MOL000098	quercetin
Tusizi	MOL000098	quercetin
Tusizi	MOL000098	quercetin
Tusizi	MOL000098	quercetin
Tusizi	MOL000098	quercetin
Tusizi	MOL000098	quercetin
Tusizi	MOL000098	quercetin
Tusizi	MOL000098	quercetin
Tusizi	MOL000098	quercetin
Tusizi	MOL000098	quercetin
Tusizi	MOL000098	quercetin

Tusizi	MOL000098	quercetin
Tusizi	MOL000098	quercetin
Wulingzhi	HBIN005155	2α,19α-dihydroxyursolicacid
Wulingzhi	HBIN008021	3β,15xi,16-trihydroxy-isopimaricacid
Wulingzhi	HBIN010328	4'-dihydroxywogonin
Wulingzhi	HBIN011744	5-methoxy-7-hydroxycoumarin
Wulingzhi	HBIN012740	6-oxo purine
Wulingzhi	HBIN013922	9,10-dicarboxylic acid 3-carene
Wulingzhi	HBIN015193	allantoin
Wulingzhi	HBIN015335	alpha-agarofuran
Wulingzhi	HBIN017786	benzoic acid
Wulingzhi	HBIN017942	beta 2-bisabolene
Wulingzhi	HBIN017944	β2-sitosterol
Wulingzhi	HBIN019388	California laurel ketone
Wulingzhi	HBIN019948	catechol
Wulingzhi	HBIN022771	daucosterol
Wulingzhi	HBIN024266	Diterpene acid
Wulingzhi	HBIN028037	glucose
Wulingzhi	HBIN029304	Hexahydro Fani-one
Wulingzhi	HBIN031023	isophorone
Wulingzhi	HBIN031412	jacoumaric acid
Wulingzhi	HBIN031428	japondipsaponin e1
Wulingzhi	HBIN033622	l-tyrosine
Wulingzhi	HBIN035490	m-hydroxybenzoicacid
Wulingzhi	HBIN036866	N heptacosane
Wulingzhi	HBIN038272	Original catechol
Wulingzhi	HBIN040523	Pomolic acid
Wulingzhi	HBIN040532	ponalactone a
Wulingzhi	HBIN044156	sitostanol
Wulingzhi	HBIN045321	Table friedelinol
Wulingzhi	HBIN046507	tocopherylquinone
Wulingzhi	HBIN047083	trihydroxy isosterocholenic acid
Wulingzhi	HBIN047561	uracil
Wulingzhi	HBIN047573	urea
Wulingzhi	HBIN047577	Uric acid
Wulingzhi	HBIN047613	ursolic acid
Wulingzhi	HBIN047823	Verbena alkenyl ether
Wulingzhi	HBIN047828	verbenone
Wulingzhi	HBIN048047	vitamin c
Wulingzhi	HBIN048405	wulingzhic acid
Wulingzhi	HBIN048406	Wulingzhi diterpene acid

Target
Gamma-aminobutyric-acid receptor subunit alpha-1
Progesterone receptor
Mineralocorticoid receptor
Tumor necrosis factor
Interleukin-6
Monocyte differentiation antigen CD14
Lipopolysaccharide-binding protein
Progesterone receptor
Progesterone receptor
Nuclear receptor coactivator 2
Prostaglandin G/H synthase 1
Prostaglandin G/H synthase 2
Heat shock protein HSP 90-alpha
Phosphatidylinositol-4,5-bisphosphate 3-kinase catalytic subunit gamma isoform
Potassium voltage-gated channel subfamily H member 2
cAMP-dependent protein kinase catalytic subunit alpha
D(1A) dopamine receptor
Muscarinic acetylcholine receptor M3
Muscarinic acetylcholine receptor M1
Sodium channel protein type 5 subunit alpha
Gamma-aminobutyric-acid receptor subunit alpha-2
Muscarinic acetylcholine receptor M4
cGMP-inhibited 3',5'-cyclic phosphodiesterase A
5-hydroxytryptamine 2A receptor
Gamma-aminobutyric-acid receptor subunit alpha-5
Alpha-1A adrenergic receptor
Gamma-aminobutyric-acid receptor subunit alpha-3
Muscarinic acetylcholine receptor M2
Alpha-1B adrenergic receptor
Beta-2 adrenergic receptor
Neuronal acetylcholine receptor subunit alpha-2
Sodium-dependent serotonin transporter
Mu-type opioid receptor
Gamma-aminobutyric-acid receptor subunit alpha-1
Neuronal acetylcholine receptor subunit alpha-7
Cytochrome P450-cam
Apoptosis regulator Bcl-2
Apoptosis regulator BAX
Caspase-9
Transcription factor AP-1
Caspase-3
Caspase-8
Protein kinase C alpha type
Transforming growth factor beta-1
Serum paraoxonase/arylesterase 1
Microtubule-associated protein 2
Progesterone receptor
Nuclear receptor coactivator 2
Mineralocorticoid receptor
Nitric oxide synthase, inducible
Prostaglandin G/H synthase 1
Androgen receptor
Peroxisome proliferator-activated receptor gamma
Prostaglandin G/H synthase 2
Heat shock protein HSP 90-alpha
Phosphatidylinositol-4,5-bisphosphate 3-kinase catalytic subunit gamma isoform

cAMP-dependent protein kinase catalytic subunit alpha
Nuclear receptor coactivator 2
Dipeptidyl peptidase 4
Trypsin-1
Progesterone receptor
Prothrombin
Muscarinic acetylcholine receptor M1
Nitric-oxide synthase, endothelial
Gamma-aminobutyric-acid receptor subunit alpha-2
Acetylcholinesterase
Sodium-dependent noradrenaline transporter
Muscarinic acetylcholine receptor M2
Alpha-1B adrenergic receptor
Gamma-aminobutyric-acid receptor subunit alpha-1
DNA topoisomerase 2-alpha
Coagulation factor VII
Calmodulin
Transcription factor p65
Inhibitor of nuclear factor kappa-B kinase subunit beta
RAC-alpha serine/threonine-protein kinase
Apoptosis regulator Bcl-2
Apoptosis regulator BAX
Tumor necrosis factor
Transcription factor AP-1
Activator of 90 kDa heat shock protein ATPase homolog 1
Caspase-3
Mitogen-activated protein kinase 8
Xanthine dehydrogenase/oxidase
Interstitial collagenase
Signal transducer and activator of transcription 1-alpha/beta
Cell division control protein 2 homolog
Peroxisome proliferator-activated receptor gamma
Heme oxygenase 1
Cytochrome P450 3A4
null
Cytochrome P450 1A1
Intercellular adhesion molecule 1
E-selectin
Vascular cell adhesion protein 1
Nuclear receptor subfamily 1 group I member 2
Cytochrome P450 1B1
Arachidonate 5-lipoxygenase
Hyaluronan synthase 2
Aryl hydrocarbon receptor
26S proteasome non-ATPase regulatory subunit 3
Solute carrier family 2, facilitated glucose transporter member 4
Nuclear receptor subfamily 1 group I member 3
Insulin receptor
Type I iodothyronine deiodinase
Serine/threonine-protein phosphatase 2B catalytic subunit alpha isoform
Peroxidase C1A
Glutathione S-transferase Mu 1
Glutathione S-transferase Mu 2
Aldo-keto reductase family 1 member C3
Antileukoproteinase
Prostaglandin G/H synthase 1
Estrogen receptor
Prostaglandin G/H synthase 2

Heat shock protein HSP 90-alpha
Beta-lactamase
cAMP-dependent protein kinase catalytic subunit alpha
Nuclear receptor coactivator 2
Calmodulin
Retinoic acid receptor RXR-alpha
null
Hyaluronan synthase 2
Nitric oxide synthase, inducible
Prostaglandin G/H synthase 1
Prostaglandin G/H synthase 2
Aldose reductase
Estrogen receptor beta
Dipeptidyl peptidase 4
Mitogen-activated protein kinase 14
Glycogen synthase kinase-3 beta
Heat shock protein HSP 90-alpha
Cell division protein kinase 2
Serine/threonine-protein kinase Chk1
cAMP-dependent protein kinase catalytic subunit alpha
cAMP-dependent protein kinase inhibitor alpha
Phosphatidylinositol-4,5-bisphosphate 3-kinase catalytic subunit gamma isoform
Progesterone receptor
Nuclear receptor coactivator 2
Prostaglandin G/H synthase 1
Prostaglandin G/H synthase 2
Heat shock protein HSP 90-alpha
Phosphatidylinositol-4,5-bisphosphate 3-kinase catalytic subunit gamma isoform
Potassium voltage-gated channel subfamily H member 2
cAMP-dependent protein kinase catalytic subunit alpha
D(1A) dopamine receptor
Muscarinic acetylcholine receptor M3
Muscarinic acetylcholine receptor M1
Sodium channel protein type 5 subunit alpha
Gamma-aminobutyric-acid receptor subunit alpha-2
Muscarinic acetylcholine receptor M4
cGMP-inhibited 3',5'-cyclic phosphodiesterase A
5-hydroxytryptamine 2A receptor
Gamma-aminobutyric-acid receptor subunit alpha-5
Alpha-1A adrenergic receptor
Gamma-aminobutyric-acid receptor subunit alpha-3
Muscarinic acetylcholine receptor M2
Alpha-1B adrenergic receptor
Beta-2 adrenergic receptor
Neuronal acetylcholine receptor subunit alpha-2
Sodium-dependent serotonin transporter
Mu-type opioid receptor
Gamma-aminobutyric-acid receptor subunit alpha-1
Neuronal acetylcholine receptor subunit alpha-7
Cytochrome P450-cam
Apoptosis regulator Bcl-2
Apoptosis regulator BAX
Caspase-9
Transcription factor AP-1
Caspase-3
Caspase-8
Protein kinase C alpha type
Transforming growth factor beta-1

Serum paraoxonase/arylesterase 1
Microtubule-associated protein 2
Progesterone receptor
Nuclear receptor coactivator 2
Mineralocorticoid receptor
Coagulation factor X
Tyrosine-protein phosphatase non-receptor type 1
Prothrombin
Prostaglandin G/H synthase 2
Carbonic anhydrase 2
Dipeptidyl peptidase 4
Trypsin-1
Nuclear receptor coactivator 2
Prostaglandin G/H synthase 1
Prostaglandin G/H synthase 2
Nuclear receptor coactivator 2
Nitric oxide synthase, inducible
Prostaglandin G/H synthase 1
Prothrombin
Potassium voltage-gated channel subfamily H member 2
Estrogen receptor
Androgen receptor
Sodium channel protein type 5 subunit alpha
Peroxisome proliferator-activated receptor gamma
Prostaglandin G/H synthase 2
Coagulation factor VII
Vascular endothelial growth factor receptor 2
Retinoic acid receptor RXR-alpha
cGMP-inhibited 3',5'-cyclic phosphodiesterase A
Beta-2 adrenergic receptor
Estrogen receptor beta
Dipeptidyl peptidase 4
Mitogen-activated protein kinase 14
Glycogen synthase kinase-3 beta
Heat shock protein HSP 90-alpha
Cell division protein kinase 2
Serine/threonine-protein kinase Chk1
Ig gamma-1 chain C region
Proto-oncogene serine/threonine-protein kinase Pim-1
Cyclin-A2
Nuclear receptor coactivator 1
Prothrombin
Prostaglandin G/H synthase 2
Retinoic acid receptor RXR-alpha
cAMP-dependent protein kinase catalytic subunit alpha
Prostaglandin G/H synthase 2
Mineralocorticoid receptor
Glucocorticoid receptor
Nuclear receptor coactivator 2
Progesterone receptor
Nuclear receptor coactivator 2
Mineralocorticoid receptor
Cell division protein kinase 2
Prothrombin
Glycogen synthase kinase-3 beta
Progesterone receptor
Progesterone receptor
Nuclear receptor coactivator 2

Mineralocorticoid receptor
Nitric oxide synthase, inducible
Prostaglandin G/H synthase 1
Androgen receptor
Peroxisome proliferator-activated receptor gamma
Prostaglandin G/H synthase 2
Heat shock protein HSP 90-alpha
Phosphatidylinositol-4,5-bisphosphate 3-kinase catalytic subunit gamma isoform
cAMP-dependent protein kinase catalytic subunit alpha
Nuclear receptor coactivator 2
Dipeptidyl peptidase 4
Trypsin-1
Progesterone receptor
Prothrombin
Muscarinic acetylcholine receptor M1
Nitric-oxide synthase, endothelial
Gamma-aminobutyric-acid receptor subunit alpha-2
Acetylcholinesterase
Sodium-dependent noradrenaline transporter
Muscarinic acetylcholine receptor M2
Alpha-1B adrenergic receptor
Gamma-aminobutyric-acid receptor subunit alpha-1
DNA topoisomerase 2-alpha
Coagulation factor VII
Calmodulin
Transcription factor p65
Inhibitor of nuclear factor kappa-B kinase subunit beta
RAC-alpha serine/threonine-protein kinase
Apoptosis regulator Bcl-2
Apoptosis regulator BAX
Tumor necrosis factor
Transcription factor AP-1
Activator of 90 kDa heat shock protein ATPase homolog 1
Caspase-3
Mitogen-activated protein kinase 8
Xanthine dehydrogenase/oxidase
Interstitial collagenase
Signal transducer and activator of transcription 1-alpha/beta
Cell division control protein 2 homolog
Peroxisome proliferator-activated receptor gamma
Heme oxygenase 1
Cytochrome P450 3A4
null
Cytochrome P450 1A1
Intercellular adhesion molecule 1
E-selectin
Vascular cell adhesion protein 1
Nuclear receptor subfamily 1 group I member 2
Cytochrome P450 1B1
Arachidonate 5-lipoxygenase
Hyaluronan synthase 2
Aryl hydrocarbon receptor
26S proteasome non-ATPase regulatory subunit 3
Solute carrier family 2, facilitated glucose transporter member 4
Nuclear receptor subfamily 1 group I member 3
Insulin receptor
Type I iodothyronine deiodinase
Serine/threonine-protein phosphatase 2B catalytic subunit alpha isoform

Peroxidase C1A
Glutathione S-transferase Mu 1
Glutathione S-transferase Mu 2
Aldo-keto reductase family 1 member C3
Antileukoproteinase
Prostaglandin G/H synthase 1
Estrogen receptor
Prostaglandin G/H synthase 2
Heat shock protein HSP 90-alpha
Beta-lactamase
cAMP-dependent protein kinase catalytic subunit alpha
Nuclear receptor coactivator 2
Calmodulin
Retinoic acid receptor RXR-alpha
null
Hyaluronan synthase 2
Estrogen receptor
Glycogen synthase kinase-3 beta
Heat shock protein HSP 90-alpha
Cell division protein kinase 2
cAMP-dependent protein kinase catalytic subunit alpha
Prostaglandin G/H synthase 1
Androgen receptor
Peroxisome proliferator-activated receptor gamma
Prostaglandin G/H synthase 2
Heat shock protein HSP 90-alpha
Phosphatidylinositol-4,5-bisphosphate 3-kinase catalytic subunit gamma isoform
Nuclear receptor coactivator 2
Dipeptidyl peptidase 4
Aldose reductase
Trypsin-1
DNA topoisomerase 2-alpha
Prothrombin
Potassium voltage-gated channel subfamily H member 2
Sodium channel protein type 5 subunit alpha
Coagulation factor X
Beta-2 adrenergic receptor
Stromelysin-1
cAMP-dependent protein kinase catalytic subunit alpha
Coagulation factor VII
Nitric-oxide synthase, endothelial
Retinoic acid receptor RXR-alpha
Acetylcholinesterase
Gamma-aminobutyric-acid receptor subunit alpha-1
Amine oxidase [flavin-containing] B
Transcription factor p65
Epidermal growth factor receptor
RAC-alpha serine/threonine-protein kinase
null
G1/S-specific cyclin-D1
Apoptosis regulator Bcl-2
Bcl-2-like protein 1
Proto-oncogene c-Fos
Cyclin-dependent kinase inhibitor 1
Eukaryotic translation initiation factor 6
Apoptosis regulator BAX
Caspase-9
Urokinase-type plasminogen activator

72 kDa type IV collagenase
Matrix metalloproteinase-9
Mitogen-activated protein kinase 1
Interleukin-10
Retinoblastoma-associated protein
Tumor necrosis factor
Transcription factor AP-1
Interleukin-6
Cyclin-dependent kinase inhibitor 2A, isoforms 1/2/3
Activator of 90 kDa heat shock protein ATPase homolog 1
Caspase-3
Cellular tumor antigen p53
ETS domain-containing protein Elk-1
NF-kappa-B inhibitor alpha
Ornithine decarboxylase
Xanthine dehydrogenase/oxidase
Caspase-8
DNA topoisomerase 1
RAF proto-oncogene serine/threonine-protein kinase
Superoxide dismutase [Cu-Zn]
Protein kinase C alpha type
Interstitial collagenase
Hypoxia-inducible factor 1-alpha
Signal transducer and activator of transcription 1-alpha/beta
Protein CBFA2T1
Probable E3 ubiquitin-protein ligase HERC5
Cell division control protein 2 homolog
78 kDa glucose-regulated protein
Receptor tyrosine-protein kinase erbB-2
Peroxisome proliferator-activated receptor gamma
Acetyl-CoA carboxylase 1
Heme oxygenase 1
Cytochrome P450 3A4
Caveolin-1
Myc proto-oncogene protein
Tissue factor
Gap junction alpha-1 protein
Cytochrome P450 1A1
Intercellular adhesion molecule 1
Interleukin-1 beta
Small inducible cytokine A2
E-selectin
Vascular cell adhesion protein 1
Prostaglandin E2 receptor, EP3 subtype
Interleukin-8
Protein kinase C beta type
Baculoviral IAP repeat-containing protein 5
Dual oxidase 2
Nitric oxide synthase, endothelial
Heat shock protein beta-1
Transforming growth factor beta-1
Maltase-glucoamylase, intestinal
Interleukin-2
Nuclear receptor subfamily 1 group I member 2
Cytochrome P450 1B1
G2/mitotic-specific cyclin-B1
Tissue-type plasminogen activator
Thrombomodulin

Plasminogen activator inhibitor 1
Interferon gamma
Arachidonate 5-lipoxygenase
Phosphatidylinositol-3,4,5-trisphosphate 3-phosphatase and dual-specificity protein phosphatase
Interleukin-1 alpha
Myeloperoxidase
DNA topoisomerase 2-alpha
Neutrophil cytosol factor 1
ATP-binding cassette sub-family G member 2
Hyaluronan synthase 2
Nuclear factor erythroid 2-related factor 2
NAD(P)H dehydrogenase [quinone] 1
Poly [ADP-ribose] polymerase 1
Aryl hydrocarbon receptor
26S proteasome non-ATPase regulatory subunit 3
Solute carrier family 2, facilitated glucose transporter member 4
Collagen alpha-1(III) chain
DNA gyrase subunit B
C-X-C motif chemokine 11
C-X-C motif chemokine 2
DDB1- and CUL4-associated factor 5
Nuclear receptor subfamily 1 group I member 3
Serine/threonine-protein kinase Chk2
Insulin receptor
Claudin-4
Peroxisome proliferator-activated receptor alpha
Peroxisome proliferator-activated receptor delta
Heat shock factor protein 1
C-reactive protein
C-X-C motif chemokine 10
Inhibitor of nuclear factor kappa-B kinase subunit alpha
Osteopontin
Runt-related transcription factor 2
Ras association domain-containing protein 1
Transcription factor E2F1
Transcription factor E2F2
Prostatic acid phosphatase
Cathepsin D
Insulin-like growth factor-binding protein 3
Insulin-like growth factor II
CD40 ligand
Interferon regulatory factor 1
Receptor tyrosine-protein kinase erbB-3
Serum paraoxonase/arylesterase 1
Type I iodothyronine deiodinase
Procollagen C-endopeptidase enhancer 1
Puromycin-sensitive aminopeptidase
Hexokinase-2
Homeobox protein Nkx-3.1
Ras GTPase-activating protein 1
Peroxidase C1A
Glutathione S-transferase Mu 1
Glutathione S-transferase Mu 2
Mineralocorticoid receptor
Nuclear receptor coactivator 2
Mineralocorticoid receptor
Mineralocorticoid receptor
Progesterone receptor

Progesterone receptor
Progesterone receptor
Nuclear receptor coactivator 2
Muscarinic acetylcholine receptor M3
Muscarinic acetylcholine receptor M1
Gamma-aminobutyric-acid receptor subunit alpha-2
Gamma-aminobutyric-acid receptor subunit alpha-3
Muscarinic acetylcholine receptor M2
Alpha-1B adrenergic receptor
Gamma-aminobutyric-acid receptor subunit alpha-1
Glutamate receptor 2
Gamma-aminobutyric-acid receptor subunit alpha-6
Gamma-aminobutyric-acid receptor subunit alpha-5
Ig gamma-1 chain C region
null
null
Lysozyme
Nicotinate-nucleotide--dimethylbenzimidazole phosphoribosyltransferase
Prostaglandin G/H synthase 1
Sodium channel protein type 5 subunit alpha
Prostaglandin G/H synthase 2
Retinoic acid receptor RXR-alpha
cGMP-inhibited 3',5'-cyclic phosphodiesterase A
Sodium-dependent noradrenaline transporter
Cytochrome P450-cam
Progesterone receptor
Nuclear receptor coactivator 2
Prothrombin
Prostaglandin G/H synthase 2
Nuclear receptor coactivator 2
Estrogen receptor
Coagulation factor X
Prostaglandin G/H synthase 2
Carbonic anhydrase 2
DNA topoisomerase 2-alpha
Nuclear receptor coactivator 2
Calcium-activated potassium channel subunit alpha 1
Calmodulin
Prostaglandin G/H synthase 2
Nuclear receptor coactivator 2
Nitric oxide synthase, inducible
Prostaglandin G/H synthase 1
Androgen receptor
Peroxisome proliferator-activated receptor gamma
Prostaglandin G/H synthase 2
Heat shock protein HSP 90-alpha
Phosphatidylinositol-4,5-bisphosphate 3-kinase catalytic subunit gamma isoform
Trypsin-1
Nuclear receptor coactivator 2
Dipeptidyl peptidase 4
Prostaglandin G/H synthase 1
Androgen receptor
Prostaglandin G/H synthase 2
Heat shock protein HSP 90-alpha
cAMP-dependent protein kinase catalytic subunit alpha
Dipeptidyl peptidase 4
Phosphatidylinositol-4,5-bisphosphate 3-kinase catalytic subunit gamma isoform
cGMP-inhibited 3',5'-cyclic phosphodiesterase A

Trypsin-1
Nuclear receptor coactivator 2
Nuclear receptor coactivator 1
Calmodulin
Transcription factor p65
RAC-alpha serine/threonine-protein kinase
null
Apoptosis regulator Bcl-2
Proto-oncogene c-Fos
Apoptosis regulator BAX
Matrix metalloproteinase-9
Caspase-3
Cellular tumor antigen p53
Hypoxia-inducible factor 1-alpha
Fos-related antigen 1
Fos-related antigen 2
Cell division control protein 2 homolog
G2/mitotic-specific cyclin-B1
Myeloperoxidase
Aryl hydrocarbon receptor
Insulin-like growth factor II
Cytochrome c
Arachidonate 12-lipoxygenase, 12S-type
Nuclear factor of activated T-cells, cytoplasmic 1
Tudor domain-containing protein 7
Egl nine homolog 1
NADPH oxidase 5
Fatty acid-binding protein, epidermal
Apolipoprotein D
Prostaglandin G/H synthase 1
Prostaglandin G/H synthase 2
Heat shock protein HSP 90-alpha
Androgen receptor
Peroxisome proliferator-activated receptor gamma
Prostaglandin G/H synthase 2
Heat shock protein HSP 90-alpha
Phosphatidylinositol-4,5-bisphosphate 3-kinase catalytic subunit gamma isoform
Dipeptidyl peptidase 4
Trypsin-1
DNA topoisomerase 2-alpha
Prostaglandin G/H synthase 1
Prostaglandin G/H synthase 2
Gamma-aminobutyric-acid receptor subunit alpha-1
Heat shock protein HSP 90-alpha
cAMP-dependent protein kinase catalytic subunit alpha
RAC-alpha serine/threonine-protein kinase
null
Apoptosis regulator Bcl-2
Caspase-9
72 kDa type IV collagenase
Transcription factor AP-1
Caspase-3
Prostaglandin G/H synthase 2
Caspase-8
Interstitial collagenase
Heme oxygenase 1
Cytochrome P450 3A4
Cytochrome P450 2B1

Serum albumin
Caveolin-1
Catenin beta-1
Myc proto-oncogene protein
Caspase-7
Tissue factor
Gap junction alpha-1 protein
Coagulation factor X
Tyrosine-protein phosphatase non-receptor type 1
Progesterone receptor
Nuclear receptor coactivator 2
Prostaglandin G/H synthase 1
Prostaglandin G/H synthase 2
Heat shock protein HSP 90-alpha
Phosphatidylinositol-4,5-bisphosphate 3-kinase catalytic subunit gamma isoform
Potassium voltage-gated channel subfamily H member 2
cAMP-dependent protein kinase catalytic subunit alpha
D(1A) dopamine receptor
Muscarinic acetylcholine receptor M3
Muscarinic acetylcholine receptor M1
Sodium channel protein type 5 subunit alpha
Gamma-aminobutyric-acid receptor subunit alpha-2
Muscarinic acetylcholine receptor M4
cGMP-inhibited 3',5'-cyclic phosphodiesterase A
5-hydroxytryptamine 2A receptor
Gamma-aminobutyric-acid receptor subunit alpha-5
Alpha-1A adrenergic receptor
Gamma-aminobutyric-acid receptor subunit alpha-3
Muscarinic acetylcholine receptor M2
Alpha-1B adrenergic receptor
Beta-2 adrenergic receptor
Neuronal acetylcholine receptor subunit alpha-2
Sodium-dependent serotonin transporter
Mu-type opioid receptor
Gamma-aminobutyric-acid receptor subunit alpha-1
Neuronal acetylcholine receptor subunit alpha-7
Cytochrome P450-cam
Apoptosis regulator Bcl-2
Apoptosis regulator BAX
Caspase-9
Transcription factor AP-1
Caspase-3
Caspase-8
Protein kinase C alpha type
Transforming growth factor beta-1
Serum paraoxonase/arylesterase 1
Microtubule-associated protein 2
Nitric oxide synthase, inducible
Prostaglandin G/H synthase 1
Androgen receptor
Peroxisome proliferator-activated receptor gamma
Prostaglandin G/H synthase 2
Heat shock protein HSP 90-alpha
Phosphatidylinositol-4,5-bisphosphate 3-kinase catalytic subunit gamma isoform
cAMP-dependent protein kinase catalytic subunit alpha
Nuclear receptor coactivator 2
Dipeptidyl peptidase 4
Trypsin-1

Progesterone receptor
Prothrombin
Muscarinic acetylcholine receptor M1
Nitric-oxide synthase, endothelial
Gamma-aminobutyric-acid receptor subunit alpha-2
Acetylcholinesterase
Sodium-dependent noradrenaline transporter
Muscarinic acetylcholine receptor M2
Alpha-1B adrenergic receptor
Gamma-aminobutyric-acid receptor subunit alpha-1
DNA topoisomerase 2-alpha
Coagulation factor VII
Calmodulin
Transcription factor p65
Inhibitor of nuclear factor kappa-B kinase subunit beta
RAC-alpha serine/threonine-protein kinase
Apoptosis regulator Bcl-2
Apoptosis regulator BAX
Tumor necrosis factor
Transcription factor AP-1
Activator of 90 kDa heat shock protein ATPase homolog 1
Caspase-3
Mitogen-activated protein kinase 8
Xanthine dehydrogenase/oxidase
Interstitial collagenase
Signal transducer and activator of transcription 1-alpha/beta
Cell division control protein 2 homolog
Peroxisome proliferator-activated receptor gamma
Heme oxygenase 1
Cytochrome P450 3A4
null
Cytochrome P450 1A1
Intercellular adhesion molecule 1
E-selectin
Vascular cell adhesion protein 1
Nuclear receptor subfamily 1 group I member 2
Cytochrome P450 1B1
Arachidonate 5-lipoxygenase
Hyaluronan synthase 2
Aryl hydrocarbon receptor
26S proteasome non-ATPase regulatory subunit 3
Solute carrier family 2, facilitated glucose transporter member 4
Nuclear receptor subfamily 1 group I member 3
Insulin receptor
Type I iodothyronine deiodinase
Serine/threonine-protein phosphatase 2B catalytic subunit alpha isoform
Peroxidase C1A
Glutathione S-transferase Mu 1
Glutathione S-transferase Mu 2
Aldo-keto reductase family 1 member C3
Antileukoproteinase
Progesterone receptor
Mineralocorticoid receptor
Nuclear receptor coactivator 2
null
Ig gamma-1 chain C region
Retinoic acid receptor RXR-alpha
Nuclear receptor coactivator 1

Prostaglandin G/H synthase 1
Prostaglandin G/H synthase 2
Alpha-2A adrenergic receptor
Sodium-dependent noradrenaline transporter
Sodium-dependent dopamine transporter
Beta-2 adrenergic receptor
Aldose reductase
Urokinase-type plasminogen activator
Leukotriene A-4 hydrolase
Amine oxidase [flavin-containing] B
Amine oxidase [flavin-containing] A
cAMP-dependent protein kinase catalytic subunit alpha
Chymotrypsinogen B
Muscarinic acetylcholine receptor M3
Muscarinic acetylcholine receptor M1
Beta-1 adrenergic receptor
Sodium channel protein type 5 subunit alpha
5-hydroxytryptamine 2A receptor
Alpha-1A adrenergic receptor
Gamma-aminobutyric-acid receptor subunit alpha-3
Muscarinic acetylcholine receptor M2
Alpha-1B adrenergic receptor
Gamma-aminobutyric-acid receptor subunit alpha-1
Neuronal acetylcholine receptor subunit alpha-7
Prostaglandin G/H synthase 1
Androgen receptor
Prostaglandin G/H synthase 2
Heat shock protein HSP 90-alpha
Trypsin-1
Nuclear receptor coactivator 2
cAMP-dependent protein kinase catalytic subunit alpha
Dipeptidyl peptidase 4
Phosphatidylinositol-4,5-bisphosphate 3-kinase catalytic subunit gamma isoform
Transcription factor p65
Epidermal growth factor receptor
RAC-alpha serine/threonine-protein kinase
null
G1/S-specific cyclin-D1
Bcl-2-like protein 1
Cyclin-dependent kinase inhibitor 1
Caspase-9
72 kDa type IV collagenase
Matrix metalloproteinase-9
Mitogen-activated protein kinase 1
Interleukin-10
Retinoblastoma-associated protein
Cell division protein kinase 4
Tumor necrosis factor
Transcription factor AP-1
Interleukin-6
Caspase-3
Cellular tumor antigen p53
NF-kappa-B inhibitor alpha
Xanthine dehydrogenase/oxidase
DNA topoisomerase 1
E3 ubiquitin-protein ligase Mdm2
Amyloid beta A4 protein
Interstitial collagenase

Proliferating cell nuclear antigen
Receptor tyrosine-protein kinase erbB-2
Peroxisome proliferator-activated receptor gamma
Heme oxygenase 1
Caspase-7
Intercellular adhesion molecule 1
Induced myeloid leukemia cell differentiation protein Mcl-1
Baculoviral IAP repeat-containing protein 5
Interleukin-2
G2/mitotic-specific cyclin-B1
Tyrosinase
Interferon gamma
Interleukin-4
DNA topoisomerase 2-alpha
Baculoviral IAP repeat-containing protein 4
Solute carrier family 2, facilitated glucose transporter member 4
Insulin receptor
CD40 ligand
Prostaglandin E synthase
Kinetochore protein Nuf2
Adenylate cyclase type 2
Hepatocyte growth factor receptor
Progesterone receptor
Mineralocorticoid receptor
Cytochrome P450-cam
Nuclear receptor coactivator 2
Prostaglandin G/H synthase 1
Androgen receptor
Peroxisome proliferator-activated receptor gamma
Prostaglandin G/H synthase 2
Heat shock protein HSP 90-alpha
Phosphatidylinositol-4,5-bisphosphate 3-kinase catalytic subunit gamma isoform
Nuclear receptor coactivator 2
Dipeptidyl peptidase 4
Aldose reductase
Trypsin-1
DNA topoisomerase 2-alpha
Prothrombin
Potassium voltage-gated channel subfamily H member 2
Sodium channel protein type 5 subunit alpha
Coagulation factor X
Beta-2 adrenergic receptor
Stromelysin-1
cAMP-dependent protein kinase catalytic subunit alpha
Coagulation factor VII
Nitric-oxide synthase, endothelial
Retinoic acid receptor RXR-alpha
Acetylcholinesterase
Gamma-aminobutyric-acid receptor subunit alpha-1
Amine oxidase [flavin-containing] B
Transcription factor p65
Epidermal growth factor receptor
RAC-alpha serine/threonine-protein kinase
null
G1/S-specific cyclin-D1
Apoptosis regulator Bcl-2
Bcl-2-like protein 1
Proto-oncogene c-Fos

Cyclin-dependent kinase inhibitor 1
Eukaryotic translation initiation factor 6
Apoptosis regulator BAX
Caspase-9
Urokinase-type plasminogen activator
72 kDa type IV collagenase
Matrix metalloproteinase-9
Mitogen-activated protein kinase 1
Interleukin-10
Retinoblastoma-associated protein
Tumor necrosis factor
Transcription factor AP-1
Interleukin-6
Cyclin-dependent kinase inhibitor 2A, isoforms 1/2/3
Activator of 90 kDa heat shock protein ATPase homolog 1
Caspase-3
Cellular tumor antigen p53
ETS domain-containing protein Elk-1
NF-kappa-B inhibitor alpha
Ornithine decarboxylase
Xanthine dehydrogenase/oxidase
Caspase-8
DNA topoisomerase 1
RAF proto-oncogene serine/threonine-protein kinase
Superoxide dismutase [Cu-Zn]
Protein kinase C alpha type
Interstitial collagenase
Hypoxia-inducible factor 1-alpha
Signal transducer and activator of transcription 1-alpha/beta
Protein CBFA2T1
Probable E3 ubiquitin-protein ligase HERC5
Cell division control protein 2 homolog
78 kDa glucose-regulated protein
Receptor tyrosine-protein kinase erbB-2
Peroxisome proliferator-activated receptor gamma
Acetyl-CoA carboxylase 1
Heme oxygenase 1
Cytochrome P450 3A4
Caveolin-1
Myc proto-oncogene protein
Tissue factor
Gap junction alpha-1 protein
Cytochrome P450 1A1
Intercellular adhesion molecule 1
Interleukin-1 beta
Small inducible cytokine A2
E-selectin
Vascular cell adhesion protein 1
Prostaglandin E2 receptor, EP3 subtype
Interleukin-8
Protein kinase C beta type
Baculoviral IAP repeat-containing protein 5
Dual oxidase 2
Nitric oxide synthase, endothelial
Heat shock protein beta-1
Transforming growth factor beta-1
Maltase-glucoamylase, intestinal
Interleukin-2

Nuclear receptor subfamily 1 group I member 2
Cytochrome P450 1B1
G2/mitotic-specific cyclin-B1
Tissue-type plasminogen activator
Thrombomodulin
Plasminogen activator inhibitor 1
Interferon gamma
Arachidonate 5-lipoxygenase
Phosphatidylinositol-3,4,5-trisphosphate 3-phosphatase and dual-specificity protein phosphatase
Interleukin-1 alpha
Myeloperoxidase
DNA topoisomerase 2-alpha
Neutrophil cytosol factor 1
ATP-binding cassette sub-family G member 2
Hyaluronan synthase 2
Nuclear factor erythroid 2-related factor 2
NAD(P)H dehydrogenase [quinone] 1
Poly [ADP-ribose] polymerase 1
Aryl hydrocarbon receptor
26S proteasome non-ATPase regulatory subunit 3
Solute carrier family 2, facilitated glucose transporter member 4
Collagen alpha-1(III) chain
DNA gyrase subunit B
C-X-C motif chemokine 11
C-X-C motif chemokine 2
DDB1- and CUL4-associated factor 5
Nuclear receptor subfamily 1 group I member 3
Serine/threonine-protein kinase Chk2
Insulin receptor
Claudin-4
Peroxisome proliferator-activated receptor alpha
Peroxisome proliferator-activated receptor delta
Heat shock factor protein 1
C-reactive protein
C-X-C motif chemokine 10
Inhibitor of nuclear factor kappa-B kinase subunit alpha
Osteopontin
Runt-related transcription factor 2
Ras association domain-containing protein 1
Transcription factor E2F1
Transcription factor E2F2
Prostatic acid phosphatase
Cathepsin D
Insulin-like growth factor-binding protein 3
Insulin-like growth factor II
CD40 ligand
Interferon regulatory factor 1
Receptor tyrosine-protein kinase erbB-3
Serum paraoxonase/arylesterase 1
Type I iodothyronine deiodinase
Procollagen C-endopeptidase enhancer 1
Puromycin-sensitive aminopeptidase
Hexokinase-2
Homeobox protein Nkx-3.1
Ras GTPase-activating protein 1
Peroxidase C1A
Glutathione S-transferase Mu 1
Glutathione S-transferase Mu 2

Muscarinic acetylcholine receptor M1
Nitric-oxide synthase, endothelial
Retinoic acid receptor RXR-alpha
cGMP-inhibited 3',5'-cyclic phosphodiesterase A
Alpha-1B adrenergic receptor
Sodium-dependent dopamine transporter
Beta-2 adrenergic receptor
Sodium-dependent serotonin transporter
Leukotriene A-4 hydrolase
Amine oxidase [flavin-containing] B
Serum amyloid A protein
Prostaglandin G/H synthase 1
Prostaglandin G/H synthase 2
Heat shock protein HSP 90-alpha
Phosphatidylinositol-4,5-bisphosphate 3-kinase catalytic subunit gamma isoform
Prostaglandin G/H synthase 1
Muscarinic acetylcholine receptor M3
Prothrombin
Potassium voltage-gated channel subfamily H member 2
Muscarinic acetylcholine receptor M1
Sodium channel protein type 5 subunit alpha
Coagulation factor X
Muscarinic acetylcholine receptor M5
Prostaglandin G/H synthase 2
Carbonic anhydrase 2
Acetylcholinesterase
Muscarinic acetylcholine receptor M2
Alpha-1B adrenergic receptor
Beta-2 adrenergic receptor
Alpha-1D adrenergic receptor
DNA topoisomerase 2-alpha
Mu-type opioid receptor
Dipeptidyl peptidase 4
Beta-secretase 1
Heat shock protein HSP 90-alpha
Trypsin-1
Nuclear receptor coactivator 2
Nuclear receptor coactivator 1
Calcium-activated potassium channel subunit alpha 1
Calmodulin
Retinoic acid receptor RXR-alpha
cGMP-inhibited 3',5'-cyclic phosphodiesterase A
Nitric oxide synthase, inducible
Muscarinic acetylcholine receptor M3
Prothrombin
Potassium voltage-gated channel subfamily H member 2
Muscarinic acetylcholine receptor M1
Estrogen receptor
Sodium channel protein type 5 subunit alpha
Coagulation factor X
Prostaglandin G/H synthase 2
Coagulation factor VII
cGMP-inhibited 3',5'-cyclic phosphodiesterase A
Alpha-1B adrenergic receptor
Alpha-1D adrenergic receptor
DNA topoisomerase 2-alpha
Estrogen receptor beta
Dipeptidyl peptidase 4

Heat shock protein HSP 90-alpha
Trypsin-1
Nuclear receptor coactivator 2
Nuclear receptor coactivator 1
Calcium-activated potassium channel subunit alpha 1
Calmodulin
Progesterone receptor
Progesterone receptor
Progesterone receptor
Mineralocorticoid receptor
4-aminobutyrate aminotransferase, mitochondrial
Gamma-aminobutyric-acid receptor subunit alpha-1
null
null
null
Cytochrome P450-cam
Lysozyme
Bacillolysin
Nicotinate-nucleotide--dimethylbenzimidazole phosphoribosyltransferase
Nuclear receptor coactivator 2
Progesterone receptor
Mineralocorticoid receptor
Nuclear receptor coactivator 2
null
Ig gamma-1 chain C region
Retinoic acid receptor RXR-alpha
Nuclear receptor coactivator 1
Prostaglandin G/H synthase 1
Prostaglandin G/H synthase 2
Alpha-2A adrenergic receptor
Sodium-dependent noradrenaline transporter
Sodium-dependent dopamine transporter
Beta-2 adrenergic receptor
Aldose reductase
Urokinase-type plasminogen activator
Leukotriene A-4 hydrolase
Amine oxidase [flavin-containing] B
Amine oxidase [flavin-containing] A
cAMP-dependent protein kinase catalytic subunit alpha
Chymotrypsinogen B
Muscarinic acetylcholine receptor M3
Muscarinic acetylcholine receptor M1
Beta-1 adrenergic receptor
Sodium channel protein type 5 subunit alpha
5-hydroxytryptamine 2A receptor
Alpha-1A adrenergic receptor
Gamma-aminobutyric-acid receptor subunit alpha-3
Muscarinic acetylcholine receptor M2
Alpha-1B adrenergic receptor
Gamma-aminobutyric-acid receptor subunit alpha-1
Neuronal acetylcholine receptor subunit alpha-7
Glucocorticoid receptor
Nuclear receptor coactivator 2
Progesterone receptor
Mineralocorticoid receptor
Transcription factor p65
RAC-alpha serine/threonine-protein kinase
null

Cyclin-dependent kinase inhibitor 1
Cellular tumor antigen p53
Prostaglandin G/H synthase 2
Fatty acid synthase
Superoxide dismutase [Cu-Zn]
Hypoxia-inducible factor 1-alpha
Nuclear receptor subfamily 1 group I member 2
Cytosolic phospholipase A2
Canalicular multispecific organic anion transporter 1
Serine/threonine-protein kinase mTOR
Nitric oxide synthase, inducible
Estrogen receptor
Androgen receptor
Coagulation factor X
Prostaglandin G/H synthase 2
Coagulation factor VII
DNA topoisomerase 2-alpha
Glycogen synthase kinase-3 beta
Proto-oncogene serine/threonine-protein kinase Pim-1
Cyclin-A2
Nuclear receptor coactivator 2
Nuclear receptor coactivator 1
Calmodulin
Progesterone receptor
Mineralocorticoid receptor
Cytochrome P450-cam
Nuclear receptor coactivator 2
Prostaglandin G/H synthase 1
Prostaglandin G/H synthase 2
Nuclear receptor coactivator 2
Prostaglandin G/H synthase 1
Nuclear receptor coactivator 2
Progesterone receptor
Nuclear receptor coactivator 2
Sodium channel protein type 5 subunit alpha
Beta-2 adrenergic receptor
Muscarinic acetylcholine receptor M3
Nuclear receptor coactivator 2
DNA topoisomerase 2-alpha
Proto-oncogene serine/threonine-protein kinase Pim-1
Tyrosine-protein phosphatase non-receptor type 1
Progesterone receptor
Nuclear receptor coactivator 2
Prostaglandin G/H synthase 1
Prostaglandin G/H synthase 2
Heat shock protein HSP 90-alpha
Phosphatidylinositol-4,5-bisphosphate 3-kinase catalytic subunit gamma isoform
Potassium voltage-gated channel subfamily H member 2
cAMP-dependent protein kinase catalytic subunit alpha
D(1A) dopamine receptor
Muscarinic acetylcholine receptor M3
Muscarinic acetylcholine receptor M1
Sodium channel protein type 5 subunit alpha
Gamma-aminobutyric-acid receptor subunit alpha-2
Muscarinic acetylcholine receptor M4
cGMP-inhibited 3',5'-cyclic phosphodiesterase A
5-hydroxytryptamine 2A receptor
Gamma-aminobutyric-acid receptor subunit alpha-5

Alpha-1A adrenergic receptor
Gamma-aminobutyric-acid receptor subunit alpha-3
Muscarinic acetylcholine receptor M2
Alpha-1B adrenergic receptor
Beta-2 adrenergic receptor
Neuronal acetylcholine receptor subunit alpha-2
Sodium-dependent serotonin transporter
Mu-type opioid receptor
Gamma-aminobutyric-acid receptor subunit alpha-1
Neuronal acetylcholine receptor subunit alpha-7
Cytochrome P450-cam
Apoptosis regulator Bcl-2
Apoptosis regulator BAX
Caspase-9
Transcription factor AP-1
Caspase-3
Caspase-8
Protein kinase C alpha type
Transforming growth factor beta-1
Serum paraoxonase/arylesterase 1
Microtubule-associated protein 2
Progesterone receptor
Nuclear receptor coactivator 2
Mineralocorticoid receptor
Progesterone receptor
Mineralocorticoid receptor
Nuclear receptor coactivator 2
null
Ig gamma-1 chain C region
Retinoic acid receptor RXR-alpha
Nuclear receptor coactivator 1
Prostaglandin G/H synthase 1
Prostaglandin G/H synthase 2
Alpha-2A adrenergic receptor
Sodium-dependent noradrenaline transporter
Sodium-dependent dopamine transporter
Beta-2 adrenergic receptor
Aldose reductase
Urokinase-type plasminogen activator
Leukotriene A-4 hydrolase
Amine oxidase [flavin-containing] B
Amine oxidase [flavin-containing] A
cAMP-dependent protein kinase catalytic subunit alpha
Chymotrypsinogen B
Muscarinic acetylcholine receptor M3
Muscarinic acetylcholine receptor M1
Beta-1 adrenergic receptor
Sodium channel protein type 5 subunit alpha
5-hydroxytryptamine 2A receptor
Alpha-1A adrenergic receptor
Gamma-aminobutyric-acid receptor subunit alpha-3
Muscarinic acetylcholine receptor M2
Alpha-1B adrenergic receptor
Gamma-aminobutyric-acid receptor subunit alpha-1
Neuronal acetylcholine receptor subunit alpha-7
Prostaglandin G/H synthase 2
Prothrombin
Prostaglandin G/H synthase 2

Nuclear receptor coactivator 2
Nitric oxide synthase, inducible
Prostaglandin G/H synthase 1
Prostaglandin G/H synthase 2
Dipeptidyl peptidase 4
Heat shock protein HSP 90-alpha
cAMP-dependent protein kinase catalytic subunit alpha
Trypsin-1
Nuclear receptor coactivator 2
Calmodulin
Phosphatidylinositol-4,5-bisphosphate 3-kinase catalytic subunit gamma isoform
Mineralocorticoid receptor
Glucocorticoid receptor
Nitric oxide synthase, inducible
Prostaglandin G/H synthase 1
D(1A) dopamine receptor
Muscarinic acetylcholine receptor M3
Prothrombin
Potassium voltage-gated channel subfamily H member 2
Muscarinic acetylcholine receptor M1
Androgen receptor
Sodium channel protein type 5 subunit alpha
Peroxisome proliferator-activated receptor gamma
Coagulation factor X
Muscarinic acetylcholine receptor M5
Prostaglandin G/H synthase 2
Alpha-2C adrenergic receptor
Muscarinic acetylcholine receptor M4
Delta-type opioid receptor
Acetylcholinesterase
5-hydroxytryptamine 2A receptor
Alpha-1B adrenergic receptor
Beta-2 adrenergic receptor
Alpha-1D adrenergic receptor
Sodium-dependent serotonin transporter
Mu-type opioid receptor
Dipeptidyl peptidase 4
Heat shock protein HSP 90-alpha
cAMP-dependent protein kinase catalytic subunit alpha
Trypsin-1
Calmodulin
Nitric oxide synthase, inducible
Prostaglandin G/H synthase 1
Estrogen receptor
Androgen receptor
Peroxisome proliferator-activated receptor gamma
Prostaglandin G/H synthase 2
Tyrosine-protein phosphatase non-receptor type 1
Estrogen receptor beta
Dipeptidyl peptidase 4
Mitogen-activated protein kinase 14
Glycogen synthase kinase-3 beta
Heat shock protein HSP 90-alpha
Cell division protein kinase 2
Phosphatidylinositol-4,5-bisphosphate 3-kinase catalytic subunit gamma isoform
cAMP-dependent protein kinase catalytic subunit alpha
Trypsin-1
Proto-oncogene serine/threonine-protein kinase Pim-1

Cyclin-A2
Nuclear receptor coactivator 2
Calmodulin
Glycogen phosphorylase, muscle form
Peroxisome proliferator-activated receptor delta
Serine/threonine-protein kinase Chk1
Aldose reductase
Nuclear receptor coactivator 1
Coagulation factor VII
Prothrombin
Nitric-oxide synthase, endothelial
Acetylcholinesterase
Gamma-aminobutyric-acid receptor subunit alpha-1
Amine oxidase [flavin-containing] B
Glutamate receptor 2
Cytochrome P450-cam
Transcription factor p65
Xanthine dehydrogenase/oxidase
Neutrophil cytosol factor 1
Oxidized low-density lipoprotein receptor 1
Progesterone receptor
Nuclear receptor coactivator 2
Mineralocorticoid receptor
Nitric oxide synthase, inducible
Prostaglandin G/H synthase 1
Androgen receptor
Peroxisome proliferator-activated receptor gamma
Prostaglandin G/H synthase 2
Heat shock protein HSP 90-alpha
Phosphatidylinositol-4,5-bisphosphate 3-kinase catalytic subunit gamma isoform
cAMP-dependent protein kinase catalytic subunit alpha
Nuclear receptor coactivator 2
Dipeptidyl peptidase 4
Trypsin-1
Progesterone receptor
Prothrombin
Muscarinic acetylcholine receptor M1
Nitric-oxide synthase, endothelial
Gamma-aminobutyric-acid receptor subunit alpha-2
Acetylcholinesterase
Sodium-dependent noradrenaline transporter
Muscarinic acetylcholine receptor M2
Alpha-1B adrenergic receptor
Gamma-aminobutyric-acid receptor subunit alpha-1
DNA topoisomerase 2-alpha
Coagulation factor VII
Calmodulin
Transcription factor p65
Inhibitor of nuclear factor kappa-B kinase subunit beta
RAC-alpha serine/threonine-protein kinase
Apoptosis regulator Bcl-2
Apoptosis regulator BAX
Tumor necrosis factor
Transcription factor AP-1
Activator of 90 kDa heat shock protein ATPase homolog 1
Caspase-3
Mitogen-activated protein kinase 8
Xanthine dehydrogenase/oxidase

Interstitial collagenase
Signal transducer and activator of transcription 1-alpha/beta
Cell division control protein 2 homolog
Peroxisome proliferator-activated receptor gamma
Heme oxygenase 1
Cytochrome P450 3A4
null
Cytochrome P450 1A1
Intercellular adhesion molecule 1
E-selectin
Vascular cell adhesion protein 1
Nuclear receptor subfamily 1 group I member 2
Cytochrome P450 1B1
Arachidonate 5-lipoxygenase
Hyaluronan synthase 2
Aryl hydrocarbon receptor
26S proteasome non-ATPase regulatory subunit 3
Solute carrier family 2, facilitated glucose transporter member 4
Nuclear receptor subfamily 1 group I member 3
Insulin receptor
Type I iodothyronine deiodinase
Serine/threonine-protein phosphatase 2B catalytic subunit alpha isoform
Peroxidase C1A
Glutathione S-transferase Mu 1
Glutathione S-transferase Mu 2
Aldo-keto reductase family 1 member C3
Antileukoproteinase
Progesterone receptor
Mineralocorticoid receptor
Nuclear receptor coactivator 2
null
Ig gamma-1 chain C region
Retinoic acid receptor RXR-alpha
Nuclear receptor coactivator 1
Prostaglandin G/H synthase 1
Prostaglandin G/H synthase 2
Alpha-2A adrenergic receptor
Sodium-dependent noradrenaline transporter
Sodium-dependent dopamine transporter
Beta-2 adrenergic receptor
Aldose reductase
Urokinase-type plasminogen activator
Leukotriene A-4 hydrolase
Amine oxidase [flavin-containing] B
Amine oxidase [flavin-containing] A
cAMP-dependent protein kinase catalytic subunit alpha
Chymotrypsinogen B
Muscarinic acetylcholine receptor M3
Muscarinic acetylcholine receptor M1
Beta-1 adrenergic receptor
Sodium channel protein type 5 subunit alpha
5-hydroxytryptamine 2A receptor
Alpha-1A adrenergic receptor
Gamma-aminobutyric-acid receptor subunit alpha-3
Muscarinic acetylcholine receptor M2
Alpha-1B adrenergic receptor
Gamma-aminobutyric-acid receptor subunit alpha-1
Neuronal acetylcholine receptor subunit alpha-7

Prostaglandin G/H synthase 1
Estrogen receptor
Prostaglandin G/H synthase 2
Heat shock protein HSP 90-alpha
Beta-lactamase
cAMP-dependent protein kinase catalytic subunit alpha
Prostaglandin G/H synthase 1
Androgen receptor
Peroxisome proliferator-activated receptor gamma
Prostaglandin G/H synthase 2
Heat shock protein HSP 90-alpha
Phosphatidylinositol-4,5-bisphosphate 3-kinase catalytic subunit gamma isoform
Nuclear receptor coactivator 2
Dipeptidyl peptidase 4
Aldose reductase
Trypsin-1
DNA topoisomerase 2-alpha
Prothrombin
Potassium voltage-gated channel subfamily H member 2
Sodium channel protein type 5 subunit alpha
Coagulation factor X
Beta-2 adrenergic receptor
Stromelysin-1
cAMP-dependent protein kinase catalytic subunit alpha
Coagulation factor VII
Nitric-oxide synthase, endothelial
Retinoic acid receptor RXR-alpha
Acetylcholinesterase
Gamma-aminobutyric-acid receptor subunit alpha-1
Amine oxidase [flavin-containing] B
Transcription factor p65
Epidermal growth factor receptor
RAC-alpha serine/threonine-protein kinase
null
G1/S-specific cyclin-D1
Apoptosis regulator Bcl-2
Bcl-2-like protein 1
Proto-oncogene c-Fos
Cyclin-dependent kinase inhibitor 1
Eukaryotic translation initiation factor 6
Apoptosis regulator BAX
Caspase-9
Urokinase-type plasminogen activator
72 kDa type IV collagenase
Matrix metalloproteinase-9
Mitogen-activated protein kinase 1
Interleukin-10
Retinoblastoma-associated protein
Tumor necrosis factor
Transcription factor AP-1
Interleukin-6
Cyclin-dependent kinase inhibitor 2A, isoforms 1/2/3
Activator of 90 kDa heat shock protein ATPase homolog 1
Caspase-3
Cellular tumor antigen p53
ETS domain-containing protein Elk-1
NF-kappa-B inhibitor alpha
Ornithine decarboxylase

Xanthine dehydrogenase/oxidase
Caspase-8
DNA topoisomerase 1
RAF proto-oncogene serine/threonine-protein kinase
Superoxide dismutase [Cu-Zn]
Protein kinase C alpha type
Interstitial collagenase
Hypoxia-inducible factor 1-alpha
Signal transducer and activator of transcription 1-alpha/beta
Protein CBFA2T1
Probable E3 ubiquitin-protein ligase HERC5
Cell division control protein 2 homolog
78 kDa glucose-regulated protein
Receptor tyrosine-protein kinase erbB-2
Peroxisome proliferator-activated receptor gamma
Acetyl-CoA carboxylase 1
Heme oxygenase 1
Cytochrome P450 3A4
Caveolin-1
Myc proto-oncogene protein
Tissue factor
Gap junction alpha-1 protein
Cytochrome P450 1A1
Intercellular adhesion molecule 1
Interleukin-1 beta
Small inducible cytokine A2
E-selectin
Vascular cell adhesion protein 1
Prostaglandin E2 receptor, EP3 subtype
Interleukin-8
Protein kinase C beta type
Baculoviral IAP repeat-containing protein 5
Dual oxidase 2
Nitric oxide synthase, endothelial
Heat shock protein beta-1
Transforming growth factor beta-1
Maltase-glucoamylase, intestinal
Interleukin-2
Nuclear receptor subfamily 1 group I member 2
Cytochrome P450 1B1
G2/mitotic-specific cyclin-B1
Tissue-type plasminogen activator
Thrombomodulin
Plasminogen activator inhibitor 1
Interferon gamma
Arachidonate 5-lipoxygenase
Phosphatidylinositol-3,4,5-trisphosphate 3-phosphatase and dual-specificity protein phosphatase
Interleukin-1 alpha
Myeloperoxidase
DNA topoisomerase 2-alpha
Neutrophil cytosol factor 1
ATP-binding cassette sub-family G member 2
Hyaluronan synthase 2
Nuclear factor erythroid 2-related factor 2
NAD(P)H dehydrogenase [quinone] 1
Poly [ADP-ribose] polymerase 1
Aryl hydrocarbon receptor
26S proteasome non-ATPase regulatory subunit 3

Solute carrier family 2, facilitated glucose transporter member 4
Collagen alpha-1(III) chain
DNA gyrase subunit B
C-X-C motif chemokine 11
C-X-C motif chemokine 2
DDB1- and CUL4-associated factor 5
Nuclear receptor subfamily 1 group I member 3
Serine/threonine-protein kinase Chk2
Insulin receptor
Claudin-4
Peroxisome proliferator-activated receptor alpha
Peroxisome proliferator-activated receptor delta
Heat shock factor protein 1
C-reactive protein
C-X-C motif chemokine 10
Inhibitor of nuclear factor kappa-B kinase subunit alpha
Osteopontin
Runt-related transcription factor 2
Ras association domain-containing protein 1
Transcription factor E2F1
Transcription factor E2F2
Prostatic acid phosphatase
Cathepsin D
Insulin-like growth factor-binding protein 3
Insulin-like growth factor II
CD40 ligand
Interferon regulatory factor 1
Receptor tyrosine-protein kinase erbB-3
Serum paraoxonase/arylesterase 1
Type I iodothyronine deiodinase
Procollagen C-endopeptidase enhancer 1
Puromycin-sensitive aminopeptidase
Hexokinase-2
Homeobox protein Nkx-3.1
Ras GTPase-activating protein 1
Peroxidase C1A
Glutathione S-transferase Mu 1
Glutathione S-transferase Mu 2
Progesterone receptor
Nuclear receptor coactivator 2
Mineralocorticoid receptor
Progesterone receptor
Mineralocorticoid receptor
Nuclear receptor coactivator 2
null
Ig gamma-1 chain C region
Retinoic acid receptor RXR-alpha
Nuclear receptor coactivator 1
Prostaglandin G/H synthase 1
Prostaglandin G/H synthase 2
Alpha-2A adrenergic receptor
Sodium-dependent noradrenaline transporter
Sodium-dependent dopamine transporter
Beta-2 adrenergic receptor
Aldose reductase
Urokinase-type plasminogen activator
Leukotriene A-4 hydrolase
Amine oxidase [flavin-containing] B

Amine oxidase [flavin-containing] A
cAMP-dependent protein kinase catalytic subunit alpha
Chymotrypsinogen B
Muscarinic acetylcholine receptor M3
Muscarinic acetylcholine receptor M1
Beta-1 adrenergic receptor
Sodium channel protein type 5 subunit alpha
5-hydroxytryptamine 2A receptor
Alpha-1A adrenergic receptor
Gamma-aminobutyric-acid receptor subunit alpha-3
Muscarinic acetylcholine receptor M2
Alpha-1B adrenergic receptor
Gamma-aminobutyric-acid receptor subunit alpha-1
Neuronal acetylcholine receptor subunit alpha-7
Coagulation factor X
Prostaglandin G/H synthase 2
Sodium channel protein type 5 subunit alpha
G1/S-specific cyclin-D1
Interleukin-10
Fatty acid synthase
Acetyl-CoA carboxylase 1
Glucose-6-phosphate 1-dehydrogenase
Nitric oxide synthase, endothelial
Endothelin-converting enzyme 1
Medium-chain specific acyl-CoA dehydrogenase, mitochondrial
Cytochrome P450 2B6
UDP-glucuronosyltransferase 1-1
Sterol regulatory element-binding protein 1
NADPH oxidase 3
NADPH oxidase 1
Peroxisomal acyl-coenzyme A oxidase 1
ATP-citrate synthase
Peroxisomal bifunctional enzyme
Methylglutaconyl-CoA hydratase, mitochondrial
Trifunctional enzyme subunit beta, mitochondrial
2,4-dienoyl-CoA reductase, mitochondrial
3,2-trans-enoyl-CoA isomerase, mitochondrial
Progesterone receptor
Nitric oxide synthase, inducible
Prostaglandin G/H synthase 1
Estrogen receptor
Androgen receptor
Peroxisome proliferator-activated receptor gamma
Prostaglandin G/H synthase 2
Tyrosine-protein phosphatase non-receptor type 1
Estrogen receptor beta
Dipeptidyl peptidase 4
Mitogen-activated protein kinase 14
Glycogen synthase kinase-3 beta
Heat shock protein HSP 90-alpha
Cell division protein kinase 2
Phosphatidylinositol-4,5-bisphosphate 3-kinase catalytic subunit gamma isoform
cAMP-dependent protein kinase catalytic subunit alpha
Trypsin-1
Proto-oncogene serine/threonine-protein kinase Pim-1
Cyclin-A2
Nuclear receptor coactivator 2
Calmodulin

Glycogen phosphorylase, muscle form
Peroxisome proliferator-activated receptor delta
Serine/threonine-protein kinase Chk1
Aldose reductase
Nuclear receptor coactivator 1
Coagulation factor VII
Prothrombin
Nitric-oxide synthase, endothelial
Acetylcholinesterase
Gamma-aminobutyric-acid receptor subunit alpha-1
Amine oxidase [flavin-containing] B
Glutamate receptor 2
Cytochrome P450-cam
Transcription factor p65
Xanthine dehydrogenase/oxidase
Neutrophil cytosol factor 1
Oxidized low-density lipoprotein receptor 1
Progesterone receptor
Nuclear receptor coactivator 2
Prostaglandin G/H synthase 1
Prostaglandin G/H synthase 2
Heat shock protein HSP 90-alpha
Phosphatidylinositol-4,5-bisphosphate 3-kinase catalytic subunit gamma isoform
Potassium voltage-gated channel subfamily H member 2
cAMP-dependent protein kinase catalytic subunit alpha
D(1A) dopamine receptor
Muscarinic acetylcholine receptor M3
Muscarinic acetylcholine receptor M1
Sodium channel protein type 5 subunit alpha
Gamma-aminobutyric-acid receptor subunit alpha-2
Muscarinic acetylcholine receptor M4
cGMP-inhibited 3',5'-cyclic phosphodiesterase A
5-hydroxytryptamine 2A receptor
Gamma-aminobutyric-acid receptor subunit alpha-5
Alpha-1A adrenergic receptor
Gamma-aminobutyric-acid receptor subunit alpha-3
Muscarinic acetylcholine receptor M2
Alpha-1B adrenergic receptor
Beta-2 adrenergic receptor
Neuronal acetylcholine receptor subunit alpha-2
Sodium-dependent serotonin transporter
Mu-type opioid receptor
Gamma-aminobutyric-acid receptor subunit alpha-1
Neuronal acetylcholine receptor subunit alpha-7
Cytochrome P450-cam
Apoptosis regulator Bcl-2
Apoptosis regulator BAX
Caspase-9
Transcription factor AP-1
Caspase-3
Caspase-8
Protein kinase C alpha type
Transforming growth factor beta-1
Serum paraoxonase/arylesterase 1
Microtubule-associated protein 2
Nitric oxide synthase, inducible
Prostaglandin G/H synthase 1
Androgen receptor

Peroxisome proliferator-activated receptor gamma
Prostaglandin G/H synthase 2
Heat shock protein HSP 90-alpha
Phosphatidylinositol-4,5-bisphosphate 3-kinase catalytic subunit gamma isoform
cAMP-dependent protein kinase catalytic subunit alpha
Nuclear receptor coactivator 2
Dipeptidyl peptidase 4
Trypsin-1
Progesterone receptor
Prothrombin
Muscarinic acetylcholine receptor M1
Nitric-oxide synthase, endothelial
Gamma-aminobutyric-acid receptor subunit alpha-2
Acetylcholinesterase
Sodium-dependent noradrenaline transporter
Muscarinic acetylcholine receptor M2
Alpha-1B adrenergic receptor
Gamma-aminobutyric-acid receptor subunit alpha-1
DNA topoisomerase 2-alpha
Coagulation factor VII
Calmodulin
Transcription factor p65
Inhibitor of nuclear factor kappa-B kinase subunit beta
RAC-alpha serine/threonine-protein kinase
Apoptosis regulator Bcl-2
Apoptosis regulator BAX
Tumor necrosis factor
Transcription factor AP-1
Activator of 90 kDa heat shock protein ATPase homolog 1
Caspase-3
Mitogen-activated protein kinase 8
Xanthine dehydrogenase/oxidase
Interstitial collagenase
Signal transducer and activator of transcription 1-alpha/beta
Cell division control protein 2 homolog
Peroxisome proliferator-activated receptor gamma
Heme oxygenase 1
Cytochrome P450 3A4
null
Cytochrome P450 1A1
Intercellular adhesion molecule 1
E-selectin
Vascular cell adhesion protein 1
Nuclear receptor subfamily 1 group I member 2
Cytochrome P450 1B1
Arachidonate 5-lipoxygenase
Hyaluronan synthase 2
Aryl hydrocarbon receptor
26S proteasome non-ATPase regulatory subunit 3
Solute carrier family 2, facilitated glucose transporter member 4
Nuclear receptor subfamily 1 group I member 3
Insulin receptor
Type I iodothyronine deiodinase
Serine/threonine-protein phosphatase 2B catalytic subunit alpha isoform
Peroxidase C1A
Glutathione S-transferase Mu 1
Glutathione S-transferase Mu 2
Aldo-keto reductase family 1 member C3

Antileukoproteinase
Progesterone receptor
Progesterone receptor
Mineralocorticoid receptor
4-aminobutyrate aminotransferase, mitochondrial
Gamma-aminobutyric-acid receptor subunit alpha-1
null
null
null
Cytochrome P450-cam
Lysozyme
Bacillolysin
Nicotinate-nucleotide--dimethylbenzimidazole phosphoribosyltransferase
Nuclear receptor coactivator 2
Transcription factor p65
72 kDa type IV collagenase
Tumor necrosis factor
Interleukin-6
Caspase-3
Myc proto-oncogene protein
Intercellular adhesion molecule 1
Heparanase
Immediate early response 3-interacting protein 1
CD44 antigen
Progesterone receptor
Mineralocorticoid receptor
Cytochrome P450-cam
Nuclear receptor coactivator 2
Prostaglandin G/H synthase 1
Androgen receptor
Peroxisome proliferator-activated receptor gamma
Prostaglandin G/H synthase 2
Heat shock protein HSP 90-alpha
Phosphatidylinositol-4,5-bisphosphate 3-kinase catalytic subunit gamma isoform
Nuclear receptor coactivator 2
Dipeptidyl peptidase 4
Aldose reductase
Trypsin-1
DNA topoisomerase 2-alpha
Prothrombin
Potassium voltage-gated channel subfamily H member 2
Sodium channel protein type 5 subunit alpha
Coagulation factor X
Beta-2 adrenergic receptor
Stromelysin-1
cAMP-dependent protein kinase catalytic subunit alpha
Coagulation factor VII
Nitric-oxide synthase, endothelial
Retinoic acid receptor RXR-alpha
Acetylcholinesterase
Gamma-aminobutyric-acid receptor subunit alpha-1
Amine oxidase [flavin-containing] B
Transcription factor p65
Epidermal growth factor receptor
RAC-alpha serine/threonine-protein kinase
null
G1/S-specific cyclin-D1
Apoptosis regulator Bcl-2

Bcl-2-like protein 1
Proto-oncogene c-Fos
Cyclin-dependent kinase inhibitor 1
Eukaryotic translation initiation factor 6
Apoptosis regulator BAX
Caspase-9
Urokinase-type plasminogen activator
72 kDa type IV collagenase
Matrix metalloproteinase-9
Mitogen-activated protein kinase 1
Interleukin-10
Retinoblastoma-associated protein
Tumor necrosis factor
Transcription factor AP-1
Interleukin-6
Cyclin-dependent kinase inhibitor 2A, isoforms 1/2/3
Activator of 90 kDa heat shock protein ATPase homolog 1
Caspase-3
Cellular tumor antigen p53
ETS domain-containing protein Elk-1
NF-kappa-B inhibitor alpha
Ornithine decarboxylase
Xanthine dehydrogenase/oxidase
Caspase-8
DNA topoisomerase 1
RAF proto-oncogene serine/threonine-protein kinase
Superoxide dismutase [Cu-Zn]
Protein kinase C alpha type
Interstitial collagenase
Hypoxia-inducible factor 1-alpha
Signal transducer and activator of transcription 1-alpha/beta
Protein CBFA2T1
Probable E3 ubiquitin-protein ligase HERC5
Cell division control protein 2 homolog
78 kDa glucose-regulated protein
Receptor tyrosine-protein kinase erbB-2
Peroxisome proliferator-activated receptor gamma
Acetyl-CoA carboxylase 1
Heme oxygenase 1
Cytochrome P450 3A4
Caveolin-1
Myc proto-oncogene protein
Tissue factor
Gap junction alpha-1 protein
Cytochrome P450 1A1
Intercellular adhesion molecule 1
Interleukin-1 beta
Small inducible cytokine A2
E-selectin
Vascular cell adhesion protein 1
Prostaglandin E2 receptor, EP3 subtype
Interleukin-8
Protein kinase C beta type
Baculoviral IAP repeat-containing protein 5
Dual oxidase 2
Nitric oxide synthase, endothelial
Heat shock protein beta-1
Transforming growth factor beta-1

Maltase-glucoamylase, intestinal
Interleukin-2
Nuclear receptor subfamily 1 group I member 2
Cytochrome P450 1B1
G2/mitotic-specific cyclin-B1
Tissue-type plasminogen activator
Thrombomodulin
Plasminogen activator inhibitor 1
Interferon gamma
Arachidonate 5-lipoxygenase
Phosphatidylinositol-3,4,5-trisphosphate 3-phosphatase and dual-specificity protein phosphatase
Interleukin-1 alpha
Myeloperoxidase
DNA topoisomerase 2-alpha
Neutrophil cytosol factor 1
ATP-binding cassette sub-family G member 2
Hyaluronan synthase 2
Nuclear factor erythroid 2-related factor 2
NAD(P)H dehydrogenase [quinone] 1
Poly [ADP-ribose] polymerase 1
Aryl hydrocarbon receptor
26S proteasome non-ATPase regulatory subunit 3
Solute carrier family 2, facilitated glucose transporter member 4
Collagen alpha-1(III) chain
DNA gyrase subunit B
C-X-C motif chemokine 11
C-X-C motif chemokine 2
DDB1- and CUL4-associated factor 5
Nuclear receptor subfamily 1 group I member 3
Serine/threonine-protein kinase Chk2
Insulin receptor
Claudin-4
Peroxisome proliferator-activated receptor alpha
Peroxisome proliferator-activated receptor delta
Heat shock factor protein 1
C-reactive protein
C-X-C motif chemokine 10
Inhibitor of nuclear factor kappa-B kinase subunit alpha
Osteopontin
Runt-related transcription factor 2
Ras association domain-containing protein 1
Transcription factor E2F1
Transcription factor E2F2
Prostatic acid phosphatase
Cathepsin D
Insulin-like growth factor-binding protein 3
Insulin-like growth factor II
CD40 ligand
Interferon regulatory factor 1
Receptor tyrosine-protein kinase erbB-3
Serum paraoxonase/arylesterase 1
Type I iodothyronine deiodinase
Procollagen C-endopeptidase enhancer 1
Puromycin-sensitive aminopeptidase
Hexokinase-2
Homeobox protein Nkx-3.1
Ras GTPase-activating protein 1
Peroxidase C1A

Glutathione S-transferase Mu 1
Glutathione S-transferase Mu 2

e PTEN

PTEN

: PTEN

[illegible] PTEN
