# Supplementary material for: Nourishing Kidney Promoting Ovulation Decoction (NKPOD) Attenuates Polycystic Ovary Syndrome by Downregulating miRNA-224
Source: Evid Based Complement Alternat Med. 2023 Apr 20;2023:9402155. doi: 10.1155/2023/9402155 (PMC10139811; doi:10.1155/2023/9402155)
Supplement: Supplementary Materials — Table S1: the components and possible targets of NKPOD. Table S2: the common targets of the GeneCards Database and CTD. Table S3: the common targets related to NKPOD and PCOS. Table S4: GO biological terms. [file 9402155.f1.zip › Table S2.pdf]

**Table S2** The common targets of Genecards Database and CTD Database
PEX6
PMM2
PEX1
COMT
RUNX1
GRIN1
POLG
FOXP2
BDNF
FMR1
MAOA
VWF
CACNA1C
INS
DRD2
PEX7
PTEN
PRL
CFTR
JAK2
IL6
HTR1A
IGF1
LHCGR
GNRH1
MYH9
TNF
POR
CYP19A1
NR5A1
APOE
CREBBP
LEP
PLAU
KCNQ2
ESR1
MTHFR
FSHR
POMC
CRH
CHD7
AR
TP53
ITGB3
BRCA2
F5
PRRT2
BPTF
ALB
TH
ITGA2
GBA
CGA
SERPINA1
HFE
NR3C2
LMNA

IL1B
FGFR1
MEFV
BMP15
OXT
GHR
F2
NR4A2
KCNQ3
SHBG
MSH2
CRP
ACE
ERCC6
NOS3
GH1
CDH23
MAPK1
FLI1
NR3C1
IL10
CYP2D6
IGF2
SLC6A2
BRCA1
NGF
SNRPN
CHRNA7
DBH
AKT1
CYP17A1
HDAC8
PLA2G6
GDF9
SOX3
SETD2
INSR
STAT3
APOB
WWOX
F8
PPARG
KIT
SMC1A
VEGFA
CDH2
SOX9
AFF2
GNAS
SERPINE1
SLC1A1
PIK3CA
GDNF
GP1BB
MIR21
WT1
OXTR
GALT

NPY
NR0B1
ESR2
GJB2
TTR
KCNQ1
AMH
PAFAH1B1
INSL6
FGA
CREB1
KCNN2
SERPINC1
ELN
SMAD4
GATA4
ADIPOQ
SOD1
HMBS
TGFB1
BRAF
CXCL8
MEN1
XIAP
KCNE2
SLC37A4
STON1-GTF2A1L
TPO
AGT
KCNB1
IGFBP3
REN
MMP9
SRY
CRHR1
GJA1
XK
H2AC18
FSHB
CD36
GNRHR
IL1RN
CCL2
KISS1
MPO
IGFBP1
IL2
CYP11A1
FOS
SST
MIR22
MMP2
DCC
TLR4
SMC3
LDLR
CP
FOXP3

EDNRB
KCNMA1
LHB
NOS1
ACTB
HSPG2
IGF1R
CACNB2
MIR132
SMAD3
CCR6
COL4A1
PTGS2
DNAH1
TERT
FAS
NTF3
FGF8
MVK
CASP3
KCNN3
F7
CHEK2
CYP3A4
NLRP3
EDN1
MIR17
HAMP
PDGFRA
PLG
EGFR
F10
PGR
TGFBR1
RAD21
GLI2
LEPR
APOA1
PCNA
CAMK2G
PLA2G4A
HSD17B4
STAR
NTRK1
OTX2
ADCYAP1
PCSK9
STAT1
PNKD
CYP2C9
CYP2C19
EGF
KCNH2
TF
RETN
EIF2B5
BMP6
BMP2

NOS2
CAT
IL2RA
FGG
SLC25A13
CYP11B1
HP
PON1
VDR
MIR29A
PSMD12
F3
ACHE
SERPINF1
KISS1R
BGLAP
GSTM1
IL1A
RUNX2
IL4
GATA2
STS
FOXL2
CDKN1C
ODC1
MMP1
TFR2
NCAM1
SLC29A3
STK11
PLAT
TIMP1
ABCB1
CACNA1D
HLA-A
CLU
BMP4
KCNA1
FGB
F11
GJB1
PPARA
GCK
AMHR2
ARSA
DNM1L
IRS1
FAAH
HSD3B2
H19
SEMA3A
MME
ERBB4
HERC2
ADRB2
ENG
GP9
CRHR2

LOX
CD4
EDNRA
RBP4
DES
SOX10
RB1
POLG2
SMARCB1
AFP
PROK2
NFKB1
BTK
TG
CYP1B1
FGF2
MYC
ZFPM2
TBL1XR1
CYP1A2
STIM1
AVPR1B
G6PD
SIRT1
IL1R1
GAL
ALDH3A2
CDON
BMPR1B
HSD11B1
PPARGC1A
HSPB1
NPM1
ETS1
CACNA1S
ANOS1
EIF2B2
NDN
ACP5
IL5
SOD2
AQP4
STAG3
FANCA
MMP13
NBEAL2
PDE4D
HMGCR
GSR
FOXC1
NOG
IL18
CDKN2A
CSF2
NGFR
GATA6
DHH
STUB1

VHL
KCNJ2
BNC1
PRKAR1A
NOTCH2
TGFB2
KIF11
CDH1
CYP3A5
LCAT
TFRC
ERBB2
TLR2
ERCC8
PDGFB
GHRH
WNT4
MIR24-1
ICAM1
CAV1
SRC
TKT
USP8
AGPS
MDM2
GAPDH
HSPD1
MIR222
HESX1
TEK
LRP2
CCL5
KCNA4
GABRB1
CDKN1B
KITLG
CXCL12
CTSD
TACR3
TRPV1
NF2
CCND1
SCP2
GSTP1
FGF14
COX5A
IGFBP2
MIR143
GLUL
HPGD
TRH
PIBF1
CYP2E1
DMPK
PC
FLT1
BCOR
ZMPSTE24

RGS2
MIR93
KNG1
CSF1
PHKA2
SRD5A1
MAP3K1
FBLN5
AKT2
NPY5R
ERBB3
HSD11B2
WAS
PRLR
INHA
MKRN3
CYP2A6
PSTPIP1
AXL
PTGIS
ITGA4
AIP
SLC6A9
PRSS23
BBS9
ACTC1
NR1H2
CACNB4
WNT7A
KCNAB2
CRYAB
CARTPT
F13A1
EIF2B3
MIR200B
CCL11
IRS2
BRD4
KCNE1
GDF5
PDE6B
CXCR4
AQP1
NOTCH4
PSMC3IP
TNNI3
GSTT1
STAT5B
CYP27B1
FKBP4
MIR30A
MIR182
PIK3R1
JUN
DUSP6
PLA2G7
EDN2
SMARCE1

MMP14
CALB2
BMPR2
TNFRSF1B
GHSR
SMAD2
CYB5A
SLC2A4
ADM
CCND2
ADCYAP1R1
ROBO1
KDR
FANCM
FEZF1
DKK1
CYBA
VIM
THBS1
SCARB1
SPIDR
PSMB8
IL15
BCL2
CTDP1
AGER
BAP1
EIF2B4
P4HB
CNR2
WIPF1
SPRY4
TIMP3
PTGS1
TPI1
GATA3
MAPK10
TIMP2
HMGB1
HMGA2
CCL3
RAD54L
POMGNT2
MAPK3
SLCO1B1
ADAMTS1
NRP1
CRHBP
FOXO1
SERPINA6
SLC2A2
CASP1
CYP2B6
IL6R
BRDT
MIR23A
KCND3
RAC1

PGF
CDKN1A
FYN
SMAD6
DCN
FLRT3
CCDC141
LIPE
ADCY10
HSP90AA1
CCN2
GJA5
CDKN2B
RGS5
MCFD2
SMO
HSD3B1
EGR1
PRPF8
HSPA4
RNF216
HDAC2
IL6ST
KLK3
LIF
WDR11
CD46
LMAN1
NAMPT
BCL2L1
SELL
HSPA8
CYP1A1
ANG
DPP4
NR1I2
HDAC1
CPOX
SP1
YARS1
IGF2R
MUC1
ZP3
ANGPT2
KCNC1
CPE
CCL4
SERPINA7
INHBA
EREG
IL4R
FGD4
FOXO3
KCNQ5
ETHE1
LHX4
ITGB2
SLPI

NDNF
NSMF
KCNA5
IL7
TNFAIP6
NR2F2
ACE2
EZR
CXCL1
CPLX2
FST
DGAT1
PGK1
ITGB4
NRIP1
FGF17
CD59
MIR423
PPARD
BMP7
FRMD7
ITGB1
PTX3
CD44
AGO1
ALOX12
PODXL
DENND1A
RARA
CD52
SYCE1
NOBOX
ADAM17
NPPC
TRPV6
GPER1
NES
KCNK3
MIR141
KCNH5
FLT4
RARRES2
SRA1
BLVRB
PBX1
MIRLET7B
LIFR
HS6ST1
DMRT3
TRAF7
HAPLN1
VAMP7
CCNH
SAA1
ADCY1
NQO1
POF1B
CLPP

IGFBP4
PCSK5
ITIH1
ANTXR1
SV2A
ZP2
MSR1
CD14
FABP3
LTF
HOXA10
PTK2B
AKR1C2
AHR
CKB
ACP1
NR0B2
ADIPOR2
PML
CD69
AMBP
JAG2
ESRRB
GJA4
MUC5B
MIRLET7I
FDXR
IL17RD
ADAMTS4
PTGES
UBE2T
LAMA1
ITGA1
NR4A1
SPAM1
MCL1
PCSK1
TRPV5
CCR2
PLAUR
RSPO1
GPR149
NCOA3
DIAPH2
CDK4
CYP2R1
MKI67
ZBTB16
PCK1
ACKR2
H6PD
KCNN1
ALPP
TXN
KHDRBS1
BECN1
MUC16
FGF1

SMARCA5
ZP1
TFPI
BIRC5
DPY19L2
MIR200A
HYAL1
MAX
GC
F13B
APLN
CASP7
TGFA
ESRRA
INHBB
PRDX2
BCL2A1
EFNB1
APAF1
CPQ
CDKN2C
A2M
CGB3
PRKAA2
HYAL3
CCNB1
DIO2
KCNQ4
TRMT10A
IRF2BP2
ITGAX
AKR1C3
PLIN2
ITGA3
SOD3
CYP2C8
NR5A2
POU5F1
CKM
GAS5
MXI1
CACNB3
CDH5
ITGA6
PAPSS2
SLC8A1
SUMO1
IGFBP5
KCNC2
SELENBP1
HAP1
LNPEP
BAK1
FIGLA
APOC2
CD24
ADAM12
VTN

PTGER4
PLIN1
CCS
GNAI3
MMP19
CCL7
HFM1
KCNC4
CD55
TJP1
IL1R2
IL12B
PCK2
PHKG2
MEF2A
KCNS3
TRAF3IP2
MX1
CEBPB
CDK1
HSD17B1
KCNG1
APPL1
NABP1
CLDN5
FEN1
ANPEP
ALCAM
KCNJ12
SERPINA12
FUBP1
KDM1A
PTGER3
IGFBP6
KCNK2
ADAMTS6
ID2
SAA2
AKR1C1
THSD4
AQP3
MIR202
BAD
CDC25C
IGFBP7
SPIN1
ID1
CCND3
NCOA2
PAM
HSPE1
ATF2
CACNB1
CTSL
SULT1E1
MCM8
RPL14
SKP2

RCBTB1
PTPN3
PRSS35
FOXA2
HPX
ADIPOR1
SERPINB2
PDE3A
ERAL1
SULT2A1
NME1
PTPA
RPS6
PYCARD
HSD17B13
RPA2
STK4
PTMA
PRKACA
KCNAB1
GNRHR2
DAB2
BIRC2
TMSB4X
CAV2
S100A10
KCNH7
NR4A3
KCNA3
GPX3
VEGFB
ADAMTS9
RPS16
LSM4
TES
PDGFA
PABPC1L
DNMT3L
PLCZ1
E2F1
BOK
KCNG2
KCNJ4
CD58
MGAT1
PTGFR
RPL19
HSPA2
SAA4
ITGAV
CYP51A1
ID3
EPHA7
GJC1
ID4
NRF1
TGFBR3
HAS2

TPT1
SERPINE2
S100A6
SCG5
ASTL
NCOA1
WFDC2
GAS2
MED1
SCG2
PLK2
PTGER2
ECD
MARK4
GADD45A
TYRO3
HSD17B12
CIDEC
BSG
INPP5J
ECE2
ILK
RPL10A
SLCO2B1
PGRMC1
LHX8
KCNG3
PAPLN
KCND1
TNFRSF12A
PPFIA1
DHRS4
SIK2
LRRC41
CCNG1
MUC6
RACGAP1
MAP2K6
MXD4
ZP4
NPVF
SPARCL1
MSLN
ARF6
HYAL2
PPM1L
MRPL32
JUND
KCNV1
RAB4A
MIR429
PLCH1
STC1
AKAP11
FGR
HPGDS
HMGB2
DAZL

IPO7
HSF2BP
F2RL2
FOXD1
RGCC
ZAR1
TROAP
HAS1
WEE1
GPR173
PZP
AFM
AKAP1
E2F4
ACR
ZC3HAV1
STC2
TFAP4
EFNB3
TFDP1
FOXO4
ZFP36
SLC2A12
PTP4A1
HSD17B6
ADAM2
ITIH2
