# Supplementary material for: Nourishing Kidney Promoting Ovulation Decoction (NKPOD) Attenuates Polycystic Ovary Syndrome by Downregulating miRNA-224
Source: Evid Based Complement Alternat Med. 2023 Apr 20;2023:9402155. doi: 10.1155/2023/9402155 (PMC10139811; doi:10.1155/2023/9402155)
Supplement: Supplementary Materials — Table S1: the components and possible targets of NKPOD. Table S2: the common targets of the GeneCards Database and CTD. Table S3: the common targets related to NKPOD and PCOS. Table S4: GO biological terms. [file 9402155.f1.zip › Table S3.pdf]

Table S3 The common targets related to NKPOD and PCOS

MAOA
PLAU
ESR1
AR
TP53
NR3C2
IL1B
F2
NOS3
MAPK1
NR3C1
IGF2
SLC6A2
CHRNA7
AKT1
PPARG
SERPINE1
ESR2
SOD1
CXCL8
XIAP
IGFBP3
MMP9
GJA1
CCL2
MPO
FOS
MMP2
PTGS2
CASP3
F7
CHEK2
CYP3A4
EGFR
F10
PGR
PCNA
PLA2G4A
STAT1
KCNH2
NOS2
IL2RA
PON1
F3
ACHE
GSTM1
IL1A
RUNX2
IL4
ODC1
MMP1
PLAT
PPARA
ADRB2
RB1
CYP1B1
MYC

G6PD
HSPB1
ERBB2
ICAM1
CAV1
MDM2
CTSD
CCND1
ERBB3
JUN
SLC2A4
KDR
BCL2
PTGS1
CYP2B6
IL6R
CDKN1A
HSP90AA1
BCL2L1
CYP1A1
DPP4
NR1I2
SLPI
TNFAIP6
PPARD
CD44
NQO1
CD14
AHR
PTGES
MCL1
CDK4
BIRC5
CASP7
CCNB1
AKR1C3
CDK1
NCOA2
PDE3A
PRKACA
E2F1
HAS2
NCOA1
