# Supplementary material for: Nourishing Kidney Promoting Ovulation Decoction (NKPOD) Attenuates Polycystic Ovary Syndrome by Downregulating miRNA-224
Source: Evid Based Complement Alternat Med. 2023 Apr 20;2023:9402155. doi: 10.1155/2023/9402155 (PMC10139811; doi:10.1155/2023/9402155)
Supplement: Supplementary Materials — Table S1: the components and possible targets of NKPOD. Table S2: the common targets of the GeneCards Database and CTD. Table S3: the common targets related to NKPOD and PCOS. Table S4: GO biological terms. [file 9402155.f1.zip › Table S4.pdf]

Table S4 GO biological terms

ONTOLOGY	ID	Description	GeneRatio	BgRatio
BP	GO:0042493	response to drug	27/100	359/18862
BP	GO:0070482	response to oxygen levels	24/100	385/18862
BP	GO:0062197	cellular response to chemical stress	23/100	347/18862
BP	GO:0072593	reactive oxygen species metabolic process	21/100	281/18862
BP	GO:0010038	response to metal ion	22/100	352/18862
BP	GO:1901654	response to ketone	18/100	193/18862
BP	GO:0032496	response to lipopolysaccharide	21/100	326/18862
BP	GO:0002237	response to molecule of bacterial origin	21/100	346/18862
BP	GO:0001666	response to hypoxia	21/100	348/18862
BP	GO:0006367	transcription initiation from RNA polymerase II promoter	17/100	187/18862
BP	GO:0036293	response to decreased oxygen levels	21/100	360/18862
BP	GO:0009991	response to extracellular stimulus	23/100	477/18862
BP	GO:0009636	response to toxic substance	18/100	239/18862
BP	GO:0048545	response to steroid hormone	20/100	330/18862
BP	GO:0006352	DNA-templated transcription, initiation	18/100	249/18862
BP	GO:2000045	regulation of G1/S transition of mitotic cell cycle	16/100	174/18862
BP	GO:0006979	response to oxidative stress	22/100	444/18862
BP	GO:0034599	cellular response to oxidative stress	19/100	299/18862
BP	GO:0031667	response to nutrient levels	22/100	451/18862

BP	GO:1903409	reactive oxygen species biosynthetic process	14/100	123/18862
BP	GO:2000377	regulation of reactive oxygen species metabolic process	16/100	192/18862
BP	GO:1902806	regulation of cell cycle G1/S phase transition	16/100	195/18862
BP	GO:0050673	epithelial cell proliferation	21/100	428/18862
BP	GO:0048511	rhythmic process	18/100	294/18862
BP	GO:2001233	regulation of apoptotic signaling pathway	19/100	348/18862
BP	GO:0090068	positive regulation of cell cycle process	18/100	303/18862
BP	GO:0000302	response to reactive oxygen species	16/100	224/18862
BP	GO:2001234	negative regulation of apoptotic signaling pathway	16/100	224/18862
BP	GO:2000134	negative regulation of G1/S transition of mitotic cell cycle	13/100	119/18862
BP	GO:0000082	G1/S transition of mitotic cell cycle	17/100	275/18862
BP	GO:0097305	response to alcohol	16/100	233/18862
BP	GO:1902807	negative regulation of cell cycle G1/S phase transition	13/100	124/18862
BP	GO:0034614	cellular response to reactive oxygen species	14/100	159/18862
BP	GO:0044843	cell cycle G1/S phase transition	17/100	298/18862
BP	GO:0097191	extrinsic apoptotic signaling pathway	15/100	217/18862
BP	GO:0050678	regulation of epithelial cell proliferation	18/100	374/18862
BP	GO:0097193	intrinsic apoptotic signaling pathway	16/100	283/18862
BP	GO:0045787	positive regulation of cell cycle	18/100	395/18862
BP	GO:0071466	cellular response to xenobiotic stimulus	12/100	125/18862
BP	GO:0044706	multi-multicellular organism process	14/100	204/18862
BP	GO:0009410	response to xenobiotic stimulus	12/100	131/18862
BP	GO:0006809	nitric oxide biosynthetic process	10/100	73/18862
BP	GO:2000379	positive regulation of reactive oxygen species metabolic process	11/100	101/18862
BP	GO:2001237	negative regulation of extrinsic apoptotic signaling pathway	11/100	103/18862
BP	GO:0046209	nitric oxide metabolic process	10/100	77/18862

BP	GO:0007565	female pregnancy	13/100	177/18862
BP	GO:2001057	reactive nitrogen species metabolic process	10/100	78/18862
BP	GO:0033002	muscle cell proliferation	14/100	222/18862
BP	GO:0071156	regulation of cell cycle arrest	11/100	108/18862
BP	GO:0062012	regulation of small molecule metabolic process	18/100	437/18862
BP	GO:0043491	protein kinase B signaling	15/100	273/18862
BP	GO:1901990	regulation of mitotic cell cycle phase transition	18/100	439/18862
BP	GO:0009411	response to UV	12/100	146/18862
BP	GO:0071276	cellular response to cadmium	8/100	38/18862
BP	GO:0006631	fatty acid metabolic process	17/100	392/18862
BP	GO:0007050	cell cycle arrest	14/100	235/18862
BP	GO:0046686	response to cadmium ion	9/100	61/18862
BP	GO:2001236	regulation of extrinsic apoptotic signaling pathway	12/100	154/18862
BP	GO:0070997	neuron death	16/100	342/18862
BP	GO:0031571	mitotic G1 DNA damage checkpoint	9/100	62/18862
BP	GO:0044783	G1 DNA damage checkpoint	9/100	63/18862
BP	GO:0044819	mitotic G1/S transition checkpoint	9/100	63/18862
BP	GO:0006690	icosanoid metabolic process	11/100	123/18862
BP	GO:0048660	regulation of smooth muscle cell proliferation	12/100	160/18862
BP	GO:0070141	response to UV-A	6/100	14/18862
BP	GO:0048659	smooth muscle cell proliferation	12/100	162/18862
BP	GO:1901987	regulation of cell cycle phase transition	18/100	478/18862
BP	GO:0071496	cellular response to external stimulus	15/100	303/18862
BP	GO:0038034	signal transduction in absence of ligand	9/100	67/18862
BP	GO:0097192	extrinsic apoptotic signaling pathway in absence of ligand	9/100	67/18862
BP	GO:0050727	regulation of inflammatory response	16/100	366/18862
BP	GO:0071900	regulation of protein serine/threonine kinase activity	18/100	492/18862
BP	GO:0045930	negative regulation of mitotic cell cycle	15/100	321/18862

BP	GO:0009314	response to radiation	17/100	447/18862
BP	GO:0030522	intracellular receptor signaling pathway	14/100	274/18862
BP	GO:0071214	cellular response to abiotic stimulus	15/100	330/18862
BP	GO:0104004	cellular response to environmental stimulus	15/100	330/18862
BP	GO:1903037	regulation of leukocyte cell-cell adhesion	15/100	330/18862
BP	GO:0002526	acute inflammatory response	10/100	107/18862
BP	GO:0032102	negative regulation of response to external stimulus	16/100	394/18862
BP	GO:0071453	cellular response to oxygen levels	13/100	231/18862
BP	GO:0071216	cellular response to biotic stimulus	13/100	233/18862
BP	GO:0048608	reproductive structure development	16/100	405/18862
BP	GO:0061458	reproductive system development	16/100	408/18862
BP	GO:0042110	T cell activation	17/100	474/18862
BP	GO:0071158	positive regulation of cell cycle arrest	9/100	82/18862
BP	GO:0033559	unsaturated fatty acid metabolic process	10/100	115/18862
BP	GO:1903793	positive regulation of anion transport	17/100	478/18862
BP	GO:0006977	DNA damage response, signal transduction by p53 class mediator resulting in cell cycle arrest	8/100	56/18862
BP	GO:0071222	cellular response to lipopolysaccharide	12/100	197/18862
BP	GO:0072431	signal transduction involved in mitotic G1 DNA damage checkpoint	8/100	57/18862
BP	GO:1902400	intracellular signal transduction involved in G1 DNA damage checkpoint	8/100	57/18862
BP	GO:1901214	regulation of neuron death	14/100	302/18862
BP	GO:1901991	negative regulation of mitotic cell cycle phase transition	13/100	250/18862
BP	GO:0007568	aging	14/100	304/18862
BP	GO:0001676	long-chain fatty acid metabolic process	10/100	120/18862
BP	GO:0019369	arachidonic acid metabolic process	8/100	59/18862
BP	GO:1902402	signal transduction involved in mitotic DNA damage checkpoint	8/100	59/18862
BP	GO:1902403	signal transduction involved in mitotic DNA integrity checkpoint	8/100	59/18862
BP	GO:2001242	regulation of intrinsic apoptotic signaling pathway	11/100	160/18862

BP	GO:0007159	leukocyte cell-cell adhesion	15/100	366/18862
BP	GO:0072413	signal transduction involved in mitotic cell cycle checkpoint	8/100	61/18862
BP	GO:0071219	cellular response to molecule of bacterial origin	12/100	209/18862
BP	GO:0032355	response to estradiol	10/100	125/18862
BP	GO:0035265	organ growth	11/100	166/18862
BP	GO:0007623	circadian rhythm	12/100	212/18862
BP	GO:1901655	cellular response to ketone	9/100	92/18862
BP	GO:0048638	regulation of developmental growth	14/100	319/18862
BP	GO:0035690	cellular response to drug	8/100	63/18862
BP	GO:1901988	negative regulation of cell cycle phase transition	13/100	268/18862
BP	GO:0030193	regulation of blood coagulation	8/100	66/18862
BP	GO:1900046	regulation of hemostasis	8/100	67/18862
BP	GO:0046677	response to antibiotic	7/100	43/18862
BP	GO:0009266	response to temperature stimulus	12/100	228/18862
BP	GO:0051402	neuron apoptotic process	12/100	230/18862
BP	GO:0016049	cell growth	16/100	470/18862
BP	GO:0044773	mitotic DNA damage checkpoint	9/100	102/18862
BP	GO:0001558	regulation of cell growth	15/100	406/18862
BP	GO:0050818	regulation of coagulation	8/100	71/18862
BP	GO:0062013	positive regulation of small molecule metabolic process	10/100	141/18862
BP	GO:1903039	positive regulation of leukocyte cell-cell adhesion	12/100	234/18862
BP	GO:0072401	signal transduction involved in DNA integrity checkpoint	8/100	73/18862
BP	GO:0072422	signal transduction involved in DNA damage checkpoint	8/100	73/18862
BP	GO:0030330	DNA damage response, signal transduction by p53 class mediator	9/100	106/18862
BP	GO:0044774	mitotic DNA integrity checkpoint	9/100	106/18862
BP	GO:0071248	cellular response to metal ion	11/100	189/18862
BP	GO:0006692	prostanoid metabolic process	7/100	48/18862
BP	GO:0006693	prostaglandin metabolic process	7/100	48/18862
BP	GO:0072395	signal transduction involved in cell cycle checkpoint	8/100	76/18862
BP	GO:0051896	regulation of protein kinase B signaling	12/100	247/18862
BP	GO:0031960	response to corticosteroid	10/100	152/18862
BP	GO:0048145	regulation of fibroblast proliferation	8/100	79/18862

BP	GO:1902893	regulation of pri-miRNA transcription by RNA polymerase II	7/100	51/18862
BP	GO:0071383	cellular response to steroid hormone stimulus	11/100	201/18862
BP	GO:0022407	regulation of cell-cell adhesion	15/100	437/18862
BP	GO:0048144	fibroblast proliferation	8/100	80/18862
BP	GO:0050999	regulation of nitric-oxide synthase activity	7/100	52/18862
BP	GO:0061614	pri-miRNA transcription by RNA polymerase II	7/100	52/18862
BP	GO:1904019	epithelial cell apoptotic process	9/100	116/18862
BP	GO:0071456	cellular response to hypoxia	11/100	206/18862
BP	GO:0007093	mitotic cell cycle checkpoint	10/100	159/18862
BP	GO:0001516	prostaglandin biosynthetic process	6/100	31/18862
BP	GO:0046457	prostanoid biosynthetic process	6/100	31/18862
BP	GO:0045931	positive regulation of mitotic cell cycle	9/100	118/18862
BP	GO:0009416	response to light stimulus	13/100	319/18862
BP	GO:0006805	xenobiotic metabolic process	9/100	120/18862
BP	GO:1901099	negative regulation of signal transduction in absence of	6/100	32/18862
BP	GO:2001240	negative regulation of extrinsic apoptotic signaling pathway in absence of ligand	6/100	32/18862
BP	GO:0031649	heat generation	5/100	16/18862
BP	GO:0060135	maternal process involved in female pregnancy	7/100	56/18862
BP	GO:0036294	cellular response to decreased oxygen levels	11/100	214/18862
BP	GO:0071241	cellular response to inorganic substance	11/100	216/18862
BP	GO:0043276	anoikis	6/100	34/18862
BP	GO:0045765	regulation of angiogenesis	13/100	335/18862
BP	GO:0001659	temperature homeostasis	10/100	171/18862
BP	GO:0022409	positive regulation of cell-cell adhesion	12/100	276/18862
BP	GO:0001503	ossification	14/100	401/18862
BP	GO:0019216	regulation of lipid metabolic process	14/100	402/18862
BP	GO:1901992	positive regulation of mitotic cell cycle phase transition	8/100	91/18862
BP	GO:0050920	regulation of chemotaxis	11/100	224/18862
BP	GO:1901342	regulation of vasculature development	13/100	341/18862
BP	GO:0045766	positive regulation of angiogenesis	10/100	175/18862
BP	GO:0051897	positive regulation of protein kinase B signaling	10/100	175/18862

BP	GO:1904018	positive regulation of vasculature development	10/100	175/18862
BP	GO:0007596	blood coagulation	13/100	342/18862
BP	GO:0042770	signal transduction in response to DNA damage	9/100	131/18862
BP	GO:0061041	regulation of wound healing	9/100	131/18862
BP	GO:0042063	gliogenesis	12/100	283/18862
BP	GO:0008217	regulation of blood pressure	10/100	177/18862
BP	GO:0007599	hemostasis	13/100	346/18862
BP	GO:0042698	ovulation cycle	7/100	62/18862
BP	GO:0050817	coagulation	13/100	347/18862
BP	GO:0048732	gland development	14/100	413/18862
BP	GO:2001243	negative regulation of intrinsic apoptotic signaling pathway	8/100	95/18862
BP	GO:0051384	response to glucocorticoid	9/100	135/18862
BP	GO:0048661	positive regulation of smooth muscle cell proliferation	8/100	96/18862
BP	GO:0031668	cellular response to extracellular stimulus	11/100	235/18862
BP	GO:0032768	regulation of monooxygenase activity	7/100	65/18862
BP	GO:0051098	regulation of binding	13/100	357/18862
BP	GO:0010565	regulation of cellular ketone metabolic process	10/100	185/18862
BP	GO:0030308	negative regulation of cell growth	10/100	185/18862
BP	GO:0050921	positive regulation of	9/100	139/18862
BP	GO:0045785	positive regulation of cell adhesion	14/100	425/18862
BP	GO:0010948	negative regulation of cell cycle process	13/100	359/18862
BP	GO:0008630	intrinsic apoptotic signaling pathway in response to DNA damage	8/100	100/18862
BP	GO:0070663	regulation of leukocyte proliferation	11/100	241/18862
BP	GO:0010212	response to ionizing radiation	9/100	142/18862
BP	GO:0046620	regulation of organ growth	8/100	102/18862
BP	GO:0045926	negative regulation of growth	11/100	245/18862
BP	GO:0031099	regeneration	10/100	192/18862
BP	GO:0009755	hormone-mediated signaling pathway	10/100	193/18862
BP	GO:2001239	regulation of extrinsic apoptotic signaling pathway in absence of ligand	6/100	42/18862
BP	GO:0000077	DNA damage checkpoint	9/100	148/18862

BP	GO:0055093	response to hyperoxia	5/100	22/18862
BP	GO:0071902	positive regulation of protein serine/threonine kinase activity	12/100	311/18862
BP	GO:0043523	regulation of neuron apoptotic process	10/100	197/18862
BP	GO:0043627	response to estrogen	7/100	71/18862
BP	GO:0051222	positive regulation of protein transport	12/100	312/18862
BP	GO:0070661	leukocyte proliferation	12/100	312/18862
BP	GO:1901989	positive regulation of cell cycle phase transition	8/100	107/18862
BP	GO:2000278	regulation of DNA biosynthetic process	8/100	107/18862
BP	GO:0042180	cellular ketone metabolic process	11/100	254/18862
BP	GO:0008631	intrinsic apoptotic signaling pathway in response to oxidative stress	6/100	44/18862
BP	GO:0031100	animal organ regeneration	7/100	73/18862
BP	GO:0050679	positive regulation of epithelial cell proliferation	10/100	203/18862
BP	GO:0002685	regulation of leukocyte migration	10/100	205/18862
BP	GO:0072332	intrinsic apoptotic signaling pathway by p53 class mediator	7/100	75/18862
BP	GO:0031570	DNA integrity checkpoint	9/100	156/18862
BP	GO:0072331	signal transduction by p53 class mediator	11/100	263/18862
BP	GO:1901653	cellular response to peptide	13/100	391/18862
BP	GO:0006953	acute-phase response	6/100	46/18862
BP	GO:0048146	positive regulation of fibroblast proliferation	6/100	46/18862
BP	GO:0001660	fever generation	4/100	10/18862
BP	GO:0050863	regulation of T cell activation	12/100	327/18862
BP	GO:0000075	cell cycle checkpoint	10/100	209/18862
BP	GO:1904951	positive regulation of establishment of protein localization	12/100	328/18862
BP	GO:0031669	cellular response to nutrient levels	10/100	210/18862
BP	GO:0008202	steroid metabolic process	12/100	329/18862
BP	GO:1990748	cellular detoxification	8/100	115/18862
BP	GO:0008406	gonad development	10/100	212/18862
BP	GO:0009408	response to heat	9/100	161/18862
BP	GO:1903034	regulation of response to wounding	9/100	164/18862
BP	GO:0002688	regulation of leukocyte chemotaxis	8/100	119/18862
BP	GO:0120254	olefinic compound metabolic process	8/100	119/18862

BP	GO:0045137	development of primary sexual characteristics	10/100	217/18862
BP	GO:0007584	response to nutrient	9/100	165/18862
BP	GO:0019371	cyclooxygenase pathway	4/100	11/18862
BP	GO:0071695	anatomical structure maturation	10/100	218/18862
BP	GO:0048469	cell maturation	9/100	166/18862
BP	GO:0007566	embryo implantation	6/100	50/18862
BP	GO:0006633	fatty acid biosynthetic process	9/100	168/18862
BP	GO:0050670	regulation of lymphocyte proliferation	10/100	221/18862
BP	GO:2001020	regulation of response to DNA damage stimulus	10/100	221/18862
BP	GO:0097237	cellular response to toxic substance	8/100	122/18862
BP	GO:0006636	unsaturated fatty acid biosynthetic process	6/100	51/18862
BP	GO:1904645	response to amyloid-beta	6/100	51/18862
BP	GO:0032944	regulation of mononuclear cell proliferation	10/100	223/18862
BP	GO:0046651	lymphocyte proliferation	11/100	282/18862
BP	GO:0032943	mononuclear cell proliferation	11/100	285/18862
BP	GO:0010039	response to iron ion	5/100	28/18862
BP	GO:0045471	response to ethanol	8/100	126/18862
BP	GO:0010332	response to gamma radiation	6/100	53/18862
BP	GO:0035296	regulation of tube diameter	8/100	127/18862
BP	GO:0097746	blood vessel diameter maintenance	8/100	127/18862
BP	GO:0035150	regulation of tube size	8/100	128/18862
BP	GO:0036296	response to increased oxygen levels	5/100	29/18862
BP	GO:0010631	epithelial cell migration	12/100	357/18862
BP	GO:0001936	regulation of endothelial cell proliferation	9/100	177/18862
BP	GO:0014909	smooth muscle cell migration	7/100	88/18862
BP	GO:0034644	cellular response to UV	7/100	88/18862
BP	GO:0001836	release of cytochrome c from mitochondria	6/100	55/18862
BP	GO:0090132	epithelium migration	12/100	360/18862
BP	GO:0046456	icosanoid biosynthetic process	6/100	56/18862
BP	GO:0043434	response to peptide hormone	13/100	435/18862
BP	GO:0030879	mammary gland development	8/100	132/18862
BP	GO:0090130	tissue migration	12/100	365/18862
BP	GO:0001819	positive regulation of cytokine production	13/100	437/18862
BP	GO:0043401	steroid hormone mediated signaling pathway	8/100	134/18862
BP	GO:0055123	digestive system development	8/100	134/18862

BP	GO:0045907	positive regulation of vasoconstriction	5/100	31/18862
BP	GO:0001890	placenta development	8/100	135/18862
BP	GO:2000351	regulation of endothelial cell apoptotic process	6/100	58/18862
BP	GO:0051090	regulation of DNA-binding transcription factor activity	13/100	444/18862
BP	GO:1904035	regulation of epithelial cell apoptotic process	7/100	94/18862
BP	GO:0045428	regulation of nitric oxide biosynthetic process	6/100	59/18862
BP	GO:0098754	detoxification	8/100	138/18862
BP	GO:0003018	vascular process in circulatory system	10/100	245/18862
BP	GO:0032310	prostaglandin secretion	4/100	14/18862
BP	GO:0034349	glial cell apoptotic process	4/100	14/18862
BP	GO:1905952	regulation of lipid localization	9/100	189/18862
BP	GO:2001244	positive regulation of intrinsic apoptotic signaling pathway	6/100	60/18862
BP	GO:0018105	peptidyl-serine phosphorylation	11/100	310/18862
BP	GO:0001935	endothelial cell proliferation	9/100	191/18862
BP	GO:0071478	cellular response to radiation	9/100	191/18862
BP	GO:0080164	regulation of nitric oxide metabolic process	6/100	61/18862
BP	GO:0019217	regulation of fatty acid metabolic process	7/100	98/18862
BP	GO:1904031	positive regulation of cyclin-dependent protein kinase	5/100	34/18862
BP	GO:1903426	regulation of reactive oxygen species biosynthetic process	7/100	99/18862
BP	GO:0006869	lipid transport	13/100	461/18862
BP	GO:0061180	mammary gland epithelium development	6/100	63/18862
BP	GO:0034612	response to tumor necrosis factor	11/100	320/18862
BP	GO:0060249	anatomical structure homeostasis	13/100	466/18862
BP	GO:0000079	regulation of cyclin-dependent protein serine/threonine kinase activity	7/100	101/18862
BP	GO:0014812	muscle cell migration	7/100	101/18862
BP	GO:0072577	endothelial cell apoptotic process	6/100	64/18862
BP	GO:0030198	extracellular matrix organization	12/100	393/18862
BP	GO:0098869	cellular oxidant detoxification	7/100	102/18862
BP	GO:0043062	extracellular structure organization	12/100	394/18862
BP	GO:0070665	positive regulation of leukocyte proliferation	8/100	148/18862
BP	GO:0045229	external encapsulating structure organization	12/100	396/18862
BP	GO:0009612	response to mechanical stimulus	9/100	202/18862

BP	GO:0001667	ameboidal-type cell migration	13/100	473/18862
BP	GO:0007548	sex differentiation	10/100	262/18862
BP	GO:0032147	activation of protein kinase activity	11/100	328/18862
BP	GO:0015908	fatty acid transport	8/100	150/18862
BP	GO:0002696	positive regulation of leukocyte activation	12/100	401/18862
BP	GO:0030336	negative regulation of cell migration	11/100	330/18862
BP	GO:1904029	regulation of cyclin-dependent protein kinase activity	7/100	105/18862
BP	GO:0018209	peptidyl-serine modification	11/100	333/18862
BP	GO:0021700	developmental maturation	10/100	268/18862
BP	GO:0014065	phosphatidylinositol 3-kinase signaling	8/100	154/18862
BP	GO:0032094	response to food	5/100	38/18862
BP	GO:0045429	positive regulation of nitric oxide biosynthetic process	5/100	38/18862
BP	GO:0045923	positive regulation of fatty acid metabolic process	5/100	38/18862
BP	GO:0015732	prostaglandin transport	4/100	17/18862
BP	GO:0090399	replicative senescence	4/100	17/18862
BP	GO:0010001	glial cell differentiation	9/100	210/18862
BP	GO:0032368	regulation of lipid transport	8/100	156/18862
BP	GO:1903131	mononuclear cell differentiation	12/100	411/18862
BP	GO:0032963	collagen metabolic process	7/100	109/18862
BP	GO:0050867	positive regulation of cell activation	12/100	412/18862
BP	GO:1904407	positive regulation of nitric oxide metabolic process	5/100	39/18862
BP	GO:0050870	positive regulation of T cell activation	9/100	212/18862
BP	GO:0006801	superoxide metabolic process	6/100	70/18862
BP	GO:0051341	regulation of oxidoreductase activity	7/100	110/18862
BP	GO:0051100	negative regulation of binding	8/100	159/18862
BP	GO:1902895	positive regulation of pri-miRNA transcription by RNA polymerase II	5/100	40/18862
BP	GO:0043467	regulation of generation of precursor metabolites and energy	8/100	160/18862
BP	GO:0050680	negative regulation of epithelial cell proliferation	8/100	160/18862
BP	GO:0050728	negative regulation of inflammatory response	8/100	160/18862
BP	GO:2000146	negative regulation of cell motility	11/100	345/18862
BP	GO:0033077	T cell differentiation in thymus	6/100	73/18862
BP	GO:0014706	striated muscle tissue development	11/100	351/18862
BP	GO:0007569	cell aging	7/100	115/18862

BP	GO:0051271	negative regulation of cellular component movement	11/100	352/18862
BP	GO:0061045	negative regulation of wound healing	6/100	74/18862
BP	GO:0051052	regulation of DNA metabolic process	11/100	353/18862
BP	GO:0051251	positive regulation of lymphocyte activation	11/100	356/18862
BP	GO:0072330	monocarboxylic acid biosynthetic process	9/100	224/18862
BP	GO:0008637	apoptotic mitochondrial	7/100	118/18862
BP	GO:0035094	response to nicotine	5/100	43/18862
BP	GO:0042771	intrinsic apoptotic signaling pathway in response to DNA damage by p53 class mediator	5/100	44/18862
BP	GO:0018108	peptidyl-tyrosine phosphorylation	11/100	369/18862
BP	GO:0048771	tissue remodeling	8/100	175/18862
BP	GO:0060537	muscle tissue development	11/100	371/18862
BP	GO:0018212	peptidyl-tyrosine modification	11/100	372/18862
BP	GO:0014910	regulation of smooth muscle cell migration	6/100	81/18862
BP	GO:0048565	digestive tract development	7/100	125/18862
BP	GO:0051972	regulation of telomerase activity	5/100	47/18862
BP	GO:0030810	positive regulation of nucleotide biosynthetic process	4/100	22/18862
BP	GO:1900373	positive regulation of purine nucleotide biosynthetic process	4/100	22/18862
BP	GO:0040013	negative regulation of locomotion	11/100	377/18862
BP	GO:1904705	regulation of vascular associated smooth muscle cell proliferation	6/100	83/18862
BP	GO:1990874	vascular associated smooth muscle cell proliferation	6/100	83/18862
BP	GO:0060326	cell chemotaxis	10/100	306/18862
BP	GO:0002673	regulation of acute inflammatory response	5/100	48/18862
BP	GO:0030195	negative regulation of blood coagulation	5/100	48/18862
BP	GO:2000191	regulation of fatty acid transport	5/100	48/18862
BP	GO:0042176	regulation of protein catabolic process	11/100	383/18862
BP	GO:0035264	multicellular organism growth	7/100	130/18862
BP	GO:1900047	negative regulation of hemostasis	5/100	49/18862
BP	GO:0019430	removal of superoxide radicals	4/100	23/18862
BP	GO:0030194	positive regulation of blood coagulation	4/100	23/18862
BP	GO:0071157	negative regulation of cell cycle arrest	4/100	23/18862
BP	GO:1900048	positive regulation of	4/100	23/18862
BP	GO:0030217	T cell differentiation	9/100	246/18862
BP	GO:0010959	regulation of metal ion transport	9/100	247/18862
BP	GO:0002931	response to ischemia	5/100	50/18862

BP	GO:1904036	negative regulation of epithelial cell apoptotic process	5/100	50/18862
BP	GO:0002687	positive regulation of leukocyte migration	7/100	133/18862
BP	GO:0048872	homeostasis of number of cells	9/100	249/18862
BP	GO:0008584	male gonad development	7/100	134/18862
BP	GO:0071482	cellular response to light	7/100	134/18862
BP	GO:0050820	positive regulation of coagulation	4/100	24/18862
BP	GO:2000209	regulation of anoikis	4/100	24/18862
BP	GO:0042542	response to hydrogen peroxide	7/100	135/18862
BP	GO:0046546	development of primary male sexual characteristics	7/100	135/18862
BP	GO:0050671	positive regulation of lymphocyte proliferation	7/100	135/18862
BP	GO:0070371	ERK1 and ERK2 cascade	10/100	320/18862
BP	GO:0033273	response to vitamin	6/100	89/18862
BP	GO:0036473	cell death in response to oxidative stress	6/100	89/18862
BP	GO:0032946	positive regulation of mononuclear cell proliferation	7/100	136/18862
BP	GO:0045927	positive regulation of growth	9/100	253/18862
BP	GO:0050819	negative regulation of coagulation	5/100	52/18862
BP	GO:1903035	negative regulation of response to wounding	6/100	90/18862
BP	GO:0048015	phosphatidylinositol-mediated signaling	8/100	192/18862
BP	GO:0015849	organic acid transport	10/100	324/18862
BP	GO:0042730	fibrinolysis	4/100	25/18862
BP	GO:0050927	positive regulation of positive chemotaxis	4/100	25/18862
BP	GO:0071450	cellular response to oxygen radical	4/100	25/18862
BP	GO:0071451	cellular response to superoxide	4/100	25/18862
BP	GO:0002690	positive regulation of leukocyte chemotaxis	6/100	91/18862
BP	GO:0071897	DNA biosynthetic process	8/100	194/18862
BP	GO:1905475	regulation of protein localization to membrane	8/100	194/18862
BP	GO:0042098	T cell proliferation	8/100	195/18862
BP	GO:1901216	positive regulation of neuron death	6/100	92/18862
BP	GO:0010506	regulation of autophagy	10/100	328/18862
BP	GO:0042594	response to starvation	8/100	196/18862
BP	GO:0043393	regulation of protein binding	8/100	196/18862
BP	GO:0048017	inositol lipid-mediated signaling	8/100	196/18862
BP	GO:0043525	positive regulation of neuron apoptotic process	5/100	54/18862
BP	GO:0008585	female gonad development	6/100	93/18862
BP	GO:0050926	regulation of positive	4/100	26/18862
BP	GO:0050730	regulation of peptidyl-tyrosine phosphorylation	9/100	262/18862

BP	GO:1903428	positive regulation of reactive oxygen species biosynthetic process	5/100	55/18862
BP	GO:0014074	response to purine-containing compound	7/100	144/18862
BP	GO:0010971	positive regulation of G2/M transition of mitotic cell cycle	4/100	27/18862
BP	GO:0032091	negative regulation of protein binding	6/100	96/18862
BP	GO:0038127	ERBB signaling pathway	7/100	145/18862
BP	GO:0071901	negative regulation of protein serine/threonine kinase activity	7/100	145/18862
BP	GO:0046545	development of primary female sexual characteristics	6/100	97/18862
BP	GO:0051146	striated muscle cell differentiation	9/100	269/18862
BP	GO:0043281	regulation of cysteine-type endopeptidase activity involved in apoptotic process	8/100	205/18862
BP	GO:0000303	response to superoxide	4/100	28/18862
BP	GO:0001893	maternal placenta development	4/100	28/18862
BP	GO:0010575	positive regulation of vascular endothelial growth factor production	4/100	28/18862
BP	GO:0071480	cellular response to gamma radiation	4/100	28/18862
BP	GO:0072376	protein activation cascade	4/100	28/18862
BP	GO:0072378	blood coagulation, fibrin clot formation	4/100	28/18862
BP	GO:1902175	regulation of oxidative stress-induced intrinsic apoptotic signaling pathway	4/100	28/18862
BP	GO:1902253	regulation of intrinsic apoptotic signaling pathway by p53 class mediator	4/100	28/18862
BP	GO:0031663	lipopolysaccharide-mediated signaling pathway	5/100	58/18862
BP	GO:1903532	positive regulation of secretion by cell	9/100	271/18862
BP	GO:0045834	positive regulation of lipid metabolic process	7/100	150/18862
BP	GO:0010821	regulation of mitochondrion organization	7/100	151/18862
BP	GO:0000305	response to oxygen radical	4/100	29/18862
BP	GO:0019058	viral life cycle	10/100	348/18862
BP	GO:0006006	glucose metabolic process	8/100	210/18862
BP	GO:0044839	cell cycle G2/M phase transition	9/100	276/18862
BP	GO:0032890	regulation of organic acid transport	5/100	60/18862
BP	GO:0040014	regulation of multicellular organism growth	5/100	60/18862
BP	GO:0010887	negative regulation of cholesterol storage	3/100	10/18862
BP	GO:0033083	regulation of immature T cell proliferation	3/100	10/18862
BP	GO:0043200	response to amino acid	6/100	102/18862

BP	GO:0046661	male sex differentiation	7/100	153/18862
BP	GO:0019229	regulation of vasoconstriction	5/100	61/18862
BP	GO:0030888	regulation of B cell proliferation	5/100	61/18862
BP	GO:0070542	response to fatty acid	5/100	61/18862
BP	GO:0042391	regulation of membrane potential	11/100	431/18862
BP	GO:0045737	positive regulation of cyclin-dependent protein serine/threonine kinase activity	4/100	30/18862
BP	GO:1902751	positive regulation of cell cycle G2/M phase transition	4/100	30/18862
BP	GO:0010573	vascular endothelial growth factor production	5/100	62/18862
BP	GO:0032409	regulation of transporter activity	9/100	283/18862
BP	GO:0009267	cellular response to starvation	7/100	157/18862
BP	GO:2000378	negative regulation of reactive oxygen species metabolic process	5/100	63/18862
BP	GO:0034405	response to fluid shear stress	4/100	31/18862
BP	GO:1902749	regulation of cell cycle G2/M phase transition	8/100	219/18862
BP	GO:0033138	positive regulation of peptidyl-serine phosphorylation	6/100	107/18862
BP	GO:0016241	regulation of macroautophagy	7/100	160/18862
BP	GO:0045444	fat cell differentiation	8/100	221/18862
BP	GO:0032306	regulation of prostaglandin secretion	3/100	11/18862
BP	GO:0032308	positive regulation of prostaglandin secretion	3/100	11/18862
BP	GO:0033079	immature T cell proliferation	3/100	11/18862
BP	GO:0071492	cellular response to UV-A	3/100	11/18862
BP	GO:0010165	response to X-ray	4/100	32/18862
BP	GO:0038128	ERBB2 signaling pathway	4/100	32/18862
BP	GO:0001649	osteoblast differentiation	8/100	223/18862
BP	GO:0010632	regulation of epithelial cell migration	9/100	293/18862
BP	GO:0042759	long-chain fatty acid biosynthetic process	4/100	33/18862
BP	GO:0046320	regulation of fatty acid oxidation	4/100	33/18862
BP	GO:0030595	leukocyte chemotaxis	8/100	226/18862
BP	GO:0007162	negative regulation of cell adhesion	9/100	295/18862
BP	GO:0051047	positive regulation of secretion	9/100	295/18862
BP	GO:1903829	positive regulation of cellular protein localization	9/100	295/18862
BP	GO:0046660	female sex differentiation	6/100	111/18862
BP	GO:0051348	negative regulation of transferase activity	9/100	296/18862
BP	GO:0071356	cellular response to tumor necrosis factor	9/100	296/18862
BP	GO:0016125	sterol metabolic process	7/100	165/18862
BP	GO:0006066	alcohol metabolic process	10/100	373/18862
BP	GO:0007259	receptor signaling pathway via JAK-STAT	7/100	166/18862
BP	GO:0052547	regulation of peptidase activity	11/100	455/18862

BP	GO:0030518	intracellular steroid hormone receptor signaling pathway	6/100	113/18862
BP	GO:2000352	negative regulation of endothelial cell apoptotic process	4/100	34/18862
BP	GO:2000116	regulation of cysteine-type endopeptidase activity	8/100	230/18862
BP	GO:0044849	estrous cycle	3/100	12/18862
BP	GO:0042129	regulation of T cell proliferation	7/100	168/18862
BP	GO:0016101	diterpenoid metabolic process	6/100	114/18862
BP	GO:0055021	regulation of cardiac muscle tissue growth	5/100	70/18862
BP	GO:0031348	negative regulation of defense response	8/100	236/18862
BP	GO:0019932	second-messenger-mediated signaling	9/100	307/18862
BP	GO:0051881	regulation of mitochondrial membrane potential	5/100	71/18862
BP	GO:0014037	Schwann cell differentiation	4/100	36/18862
BP	GO:0043029	T cell homeostasis	4/100	36/18862
BP	GO:0051924	regulation of calcium ion transport	8/100	238/18862
BP	GO:0016264	gap junction assembly	3/100	13/18862
BP	GO:0031650	regulation of heat generation	3/100	13/18862
BP	GO:0070486	leukocyte aggregation	3/100	13/18862
BP	GO:0071236	cellular response to antibiotic	3/100	13/18862
BP	GO:0033627	cell adhesion mediated by integrin	5/100	72/18862
BP	GO:0010634	positive regulation of epithelial cell migration	7/100	175/18862
BP	GO:0097696	receptor signaling pathway via STAT	7/100	175/18862
BP	GO:0001101	response to acid chemical	6/100	119/18862
BP	GO:0010906	regulation of glucose metabolic process	6/100	119/18862
BP	GO:0009895	negative regulation of catabolic process	9/100	312/18862
BP	GO:0071346	cellular response to interferon-gamma	7/100	177/18862
BP	GO:0015718	monocarboxylic acid transport	7/100	178/18862
BP	GO:0007422	peripheral nervous system development	5/100	74/18862
BP	GO:0090316	positive regulation of intracellular protein transport	7/100	179/18862
BP	GO:0071398	cellular response to fatty acid	4/100	38/18862
BP	GO:0071548	response to dexamethasone	4/100	38/18862
BP	GO:0001937	negative regulation of endothelial cell proliferation	5/100	75/18862
BP	GO:0043536	positive regulation of blood vessel endothelial cell migration	5/100	75/18862
BP	GO:0051235	maintenance of location	9/100	319/18862
BP	GO:0006721	terpenoid metabolic process	6/100	124/18862
BP	GO:0030212	hyaluronan metabolic process	4/100	39/18862
BP	GO:2000279	negative regulation of DNA biosynthetic process	4/100	39/18862
BP	GO:0051205	protein insertion into membrane	5/100	77/18862
BP	GO:0060420	regulation of heart growth	5/100	77/18862
BP	GO:0019318	hexose metabolic process	8/100	250/18862

BP	GO:2001235	positive regulation of apoptotic signaling pathway	6/100	126/18862
BP	GO:0071560	cellular response to transforming growth factor beta	8/100	251/18862
BP	GO:0045776	negative regulation of blood pressure	4/100	40/18862
BP	GO:0060443	mammary gland morphogenesis	4/100	40/18862
BP	GO:0150077	regulation of neuroinflammatory response	4/100	40/18862
BP	GO:0014066	regulation of phosphatidylinositol 3-kinase signaling	6/100	127/18862
BP	GO:1905477	positive regulation of protein localization to membrane	6/100	127/18862
BP	GO:0042326	negative regulation of phosphorylation	10/100	407/18862
BP	GO:0046394	carboxylic acid biosynthetic process	9/100	327/18862
BP	GO:0042310	vasoconstriction	5/100	79/18862
BP	GO:0046321	positive regulation of fatty acid oxidation	3/100	15/18862
BP	GO:0010469	regulation of signaling receptor activity	7/100	188/18862
BP	GO:1902042	negative regulation of extrinsic apoptotic signaling pathway via death domain receptors	4/100	41/18862
BP	GO:0046683	response to organophosphorus	6/100	130/18862
BP	GO:0071559	response to transforming growth factor beta	8/100	257/18862
BP	GO:0002446	neutrophil mediated immunity	11/100	499/18862
BP	GO:0071674	mononuclear cell migration	7/100	190/18862
BP	GO:0042119	neutrophil activation	11/100	500/18862
BP	GO:0000086	G2/M transition of mitotic cell cycle	8/100	258/18862
BP	GO:0022617	extracellular matrix disassembly	5/100	81/18862
BP	GO:0140353	lipid export from cell	5/100	81/18862
BP	GO:0046688	response to copper ion	4/100	42/18862
BP	GO:1900371	regulation of purine nucleotide biosynthetic process	4/100	42/18862
BP	GO:0016053	organic acid biosynthetic process	9/100	335/18862
BP	GO:0001894	tissue homeostasis	8/100	260/18862
BP	GO:2001021	negative regulation of response to DNA damage stimulus	5/100	82/18862
BP	GO:0050731	positive regulation of peptidyl-tyrosine phosphorylation	7/100	193/18862
BP	GO:0030099	myeloid cell differentiation	10/100	419/18862
BP	GO:0030808	regulation of nucleotide biosynthetic process	4/100	43/18862
BP	GO:0032309	icosanoid secretion	4/100	43/18862
BP	GO:0045454	cell redox homeostasis	4/100	43/18862
BP	GO:0045840	positive regulation of mitotic nuclear division	4/100	43/18862
BP	GO:0016042	lipid catabolic process	9/100	340/18862
BP	GO:0051783	regulation of nuclear division	6/100	135/18862

BP	GO:0008625	extrinsic apoptotic signaling pathway via death domain receptors	5/100	84/18862
BP	GO:0010822	positive regulation of mitochondrion organization	5/100	84/18862
BP	GO:0051091	positive regulation of DNA-binding transcription factor activity	8/100	266/18862
BP	GO:0046427	positive regulation of receptor signaling pathway via JAK-STAT	4/100	44/18862
BP	GO:0090199	regulation of release of cytochrome c from	4/100	44/18862
BP	GO:0150076	neuroinflammatory response	4/100	44/18862
BP	GO:0034341	response to interferon-gamma	7/100	197/18862
BP	GO:0052548	regulation of endopeptidase activity	10/100	426/18862
BP	GO:0051054	positive regulation of DNA metabolic process	7/100	198/18862
BP	GO:0019915	lipid storage	5/100	85/18862
BP	GO:0032370	positive regulation of lipid transport	5/100	85/18862
BP	GO:0045861	negative regulation of proteolysis	9/100	346/18862
BP	GO:0006978	DNA damage response, signal transduction by p53 class mediator resulting in transcription of p21 class mediator	3/100	17/18862
BP	GO:0030730	sequestering of triglyceride	3/100	17/18862
BP	GO:0032305	positive regulation of icosanoid secretion	3/100	17/18862
BP	GO:0060749	mammary gland alveolus development	3/100	17/18862
BP	GO:0061377	mammary gland lobule development	3/100	17/18862
BP	GO:2000811	negative regulation of anoikis	3/100	17/18862
BP	GO:1903038	negative regulation of leukocyte cell-cell adhesion	6/100	138/18862
BP	GO:0017144	drug metabolic process	4/100	45/18862
BP	GO:0032570	response to progesterone	4/100	45/18862
BP	GO:1900087	positive regulation of G1/S transition of mitotic cell cycle	4/100	45/18862
BP	GO:0051899	membrane depolarization	5/100	87/18862
BP	GO:0005996	monosaccharide metabolic process	8/100	272/18862
BP	GO:0045981	positive regulation of nucleotide metabolic process	4/100	46/18862
BP	GO:0070849	response to epidermal growth factor	4/100	46/18862
BP	GO:1900544	positive regulation of purine nucleotide metabolic process	4/100	46/18862
BP	GO:0010389	regulation of G2/M transition of mitotic cell cycle	7/100	203/18862
BP	GO:0046890	regulation of lipid biosynthetic process	7/100	203/18862
BP	GO:0002573	myeloid leukocyte differentiation	7/100	204/18862
BP	GO:0097306	cellular response to alcohol	5/100	89/18862
BP	GO:0010885	regulation of cholesterol storage	3/100	18/18862

BP	GO:0033189	response to vitamin A	3/100	18/18862
BP	GO:0042772	DNA damage response, signal transduction resulting in transcription	3/100	18/18862
BP	GO:0051546	keratinocyte migration	3/100	18/18862
BP	GO:0009409	response to cold	4/100	47/18862
BP	GO:0030574	collagen catabolic process	4/100	47/18862
BP	GO:0030857	negative regulation of epithelial cell differentiation	4/100	47/18862
BP	GO:0042572	retinol metabolic process	4/100	47/18862
BP	GO:1904707	positive regulation of vascular associated smooth muscle cell proliferation	4/100	47/18862
BP	GO:0006720	isoprenoid metabolic process	6/100	143/18862
BP	GO:0033135	regulation of peptidyl-serine phosphorylation	6/100	143/18862
BP	GO:0048754	branching morphogenesis of an epithelial tube	6/100	143/18862
BP	GO:0006109	regulation of carbohydrate metabolic process	7/100	206/18862
BP	GO:0015980	energy derivation by oxidation of organic compounds	8/100	278/18862
BP	GO:0032868	response to insulin	8/100	278/18862
BP	GO:0043542	endothelial cell migration	8/100	278/18862
BP	GO:0002064	epithelial cell development	7/100	207/18862
BP	GO:0010675	regulation of cellular carbohydrate metabolic process	6/100	144/18862
BP	GO:0106106	cold-induced thermogenesis	6/100	144/18862
BP	GO:0120161	regulation of cold-induced thermogenesis	6/100	144/18862
BP	GO:0050708	regulation of protein secretion	8/100	279/18862
BP	GO:1902105	regulation of leukocyte differentiation	8/100	279/18862
BP	GO:0030098	lymphocyte differentiation	9/100	358/18862
BP	GO:0033628	regulation of cell adhesion mediated by integrin	4/100	48/18862
BP	GO:0051204	protein insertion into mitochondrial membrane	4/100	48/18862
BP	GO:1904894	positive regulation of receptor signaling pathway via STAT	4/100	48/18862
BP	GO:0051592	response to calcium ion	6/100	145/18862
BP	GO:0060333	interferon-gamma-mediated signaling pathway	5/100	91/18862
BP	GO:0009615	response to virus	9/100	359/18862
BP	GO:0055017	cardiac muscle tissue growth	5/100	92/18862
BP	GO:0001933	negative regulation of protein phosphorylation	9/100	362/18862
BP	GO:0042692	muscle cell differentiation	9/100	362/18862
BP	GO:0071622	regulation of granulocyte chemotaxis	4/100	49/18862
BP	GO:0002544	chronic inflammatory response	3/100	19/18862
BP	GO:0032303	regulation of icosanoid secretion	3/100	19/18862
BP	GO:0009896	positive regulation of catabolic process	10/100	450/18862
BP	GO:0008203	cholesterol metabolic process	6/100	149/18862
BP	GO:0097194	execution phase of apoptosis	5/100	94/18862
BP	GO:0060485	mesenchyme development	8/100	287/18862

BP	GO:0003012	muscle system process	10/100	453/18862
BP	GO:0043535	regulation of blood vessel endothelial cell migration	6/100	150/18862
BP	GO:0044262	cellular carbohydrate metabolic process	8/100	288/18862
BP	GO:0071715	icosanoid transport	4/100	51/18862
BP	GO:0010878	cholesterol storage	3/100	20/18862
BP	GO:0019373	epoxygenase P450 pathway	3/100	20/18862
BP	GO:0046697	decidualization	3/100	20/18862
BP	GO:0046827	positive regulation of protein export from nucleus	3/100	20/18862
BP	GO:0051412	response to corticosterone	3/100	20/18862
BP	GO:0060444	branching involved in mammary gland duct morphogenesis	3/100	20/18862
BP	GO:0071379	cellular response to prostaglandin stimulus	3/100	20/18862
BP	GO:0042100	B cell proliferation	5/100	96/18862
BP	GO:0097529	myeloid leukocyte migration	7/100	218/18862
BP	GO:0044409	entry into host	6/100	153/18862
BP	GO:1990845	adaptive thermogenesis	6/100	153/18862
BP	GO:0032388	positive regulation of intracellular transport	7/100	219/18862
BP	GO:0051701	biological process involved in interaction with host	7/100	219/18862
BP	GO:0120162	positive regulation of cold-induced thermogenesis	5/100	97/18862
BP	GO:0031647	regulation of protein stability	8/100	295/18862
BP	GO:0001541	ovarian follicle development	4/100	53/18862
BP	GO:0090151	establishment of protein localization to mitochondrial membrane	4/100	53/18862
BP	GO:0034976	response to endoplasmic reticulum stress	8/100	296/18862
BP	GO:0038083	peptidyl-tyrosine autophosphorylation	3/100	21/18862
BP	GO:0060965	negative regulation of gene silencing by miRNA	3/100	21/18862
BP	GO:0072111	cell proliferation involved in kidney development	3/100	21/18862
BP	GO:1902254	negative regulation of intrinsic apoptotic signaling pathway by p53 class mediator	3/100	21/18862
BP	GO:0042102	positive regulation of T cell proliferation	5/100	99/18862
BP	GO:0043279	response to alkaloid	5/100	99/18862
BP	GO:0030856	regulation of epithelial cell differentiation	6/100	156/18862
BP	GO:0016485	protein processing	7/100	224/18862
BP	GO:0060419	heart growth	5/100	100/18862
BP	GO:0046622	positive regulation of organ growth	4/100	54/18862
BP	GO:1902652	secondary alcohol metabolic process	6/100	158/18862
BP	GO:0070372	regulation of ERK1 and ERK2 cascade	8/100	301/18862

BP	GO:0046425	regulation of receptor signaling pathway via JAK-STAT	5/100	101/18862
BP	GO:0062014	negative regulation of small molecule metabolic process	5/100	101/18862
BP	GO:0046777	protein autophosphorylation	7/100	226/18862
BP	GO:0031331	positive regulation of cellular catabolic process	9/100	384/18862
BP	GO:0010888	negative regulation of lipid storage	3/100	22/18862
BP	GO:0045844	positive regulation of striated muscle tissue development	3/100	22/18862
BP	GO:0048636	positive regulation of muscle organ development	3/100	22/18862
BP	GO:0032637	interleukin-8 production	5/100	103/18862
BP	GO:0019748	secondary metabolic process	4/100	56/18862
BP	GO:0042743	hydrogen peroxide metabolic process	4/100	56/18862
BP	GO:1902808	positive regulation of cell cycle G1/S phase transition	4/100	56/18862
BP	GO:0043406	positive regulation of MAP kinase activity	7/100	230/18862
BP	GO:0043405	regulation of MAP kinase activity	8/100	307/18862
BP	GO:0002791	regulation of peptide secretion	8/100	308/18862
BP	GO:0002260	lymphocyte homeostasis	4/100	57/18862
BP	GO:0032757	positive regulation of interleukin-8 production	4/100	57/18862
BP	GO:0051785	positive regulation of nuclear division	4/100	57/18862
BP	GO:0031639	plasminogen activation	3/100	23/18862
BP	GO:0042738	exogenous drug catabolic process	3/100	23/18862
BP	GO:0060149	negative regulation of posttranscriptional gene silencing	3/100	23/18862
BP	GO:0060353	regulation of cell adhesion molecule production	3/100	23/18862
BP	GO:0060967	negative regulation of gene silencing by RNA	3/100	23/18862
BP	GO:1901863	positive regulation of muscle tissue development	3/100	23/18862
BP	GO:0060562	epithelial tube morphogenesis	8/100	309/18862
BP	GO:0043312	neutrophil degranulation	10/100	485/18862
BP	GO:0010574	regulation of vascular endothelial growth factor	4/100	58/18862
BP	GO:0007611	learning or memory	7/100	235/18862
BP	GO:0030001	metal ion transport	10/100	486/18862
BP	GO:0006275	regulation of DNA replication	5/100	107/18862
BP	GO:0048640	negative regulation of developmental growth	5/100	107/18862
BP	GO:0002283	neutrophil activation involved in immune response	10/100	488/18862
BP	GO:0086064	cell communication by electrical coupling involved in cardiac conduction	3/100	24/18862
BP	GO:0031294	lymphocyte costimulation	4/100	59/18862

BP	GO:0090303	positive regulation of wound healing	4/100	59/18862
BP	GO:1902041	regulation of extrinsic apoptotic signaling pathway via death domain receptors	4/100	59/18862
BP	GO:0001523	retinoid metabolic process	5/100	108/18862
BP	GO:0007088	regulation of mitotic nuclear division	5/100	109/18862
BP	GO:0050994	regulation of lipid catabolic process	4/100	60/18862
BP	GO:0071384	cellular response to corticosteroid stimulus	4/100	60/18862
BP	GO:0048639	positive regulation of developmental growth	6/100	170/18862
BP	GO:0002683	negative regulation of immune system process	9/100	403/18862
BP	GO:1904892	regulation of receptor signaling pathway via STAT	5/100	110/18862
BP	GO:0001655	urogenital system development	8/100	320/18862
BP	GO:0035902	response to immobilization stress	3/100	25/18862
BP	GO:0042737	drug catabolic process	3/100	25/18862
BP	GO:1903798	regulation of production of miRNAs involved in gene silencing by miRNA	3/100	25/18862
BP	GO:0046824	positive regulation of nucleocytoplasmic transport	4/100	61/18862
BP	GO:0022612	gland morphogenesis	5/100	111/18862
BP	GO:0071675	regulation of mononuclear cell migration	5/100	111/18862
BP	GO:1905954	positive regulation of lipid localization	5/100	111/18862
BP	GO:0006469	negative regulation of protein kinase activity	7/100	243/18862
BP	GO:0090257	regulation of muscle system process	7/100	243/18862
BP	GO:0061138	morphogenesis of a branching epithelium	6/100	173/18862
BP	GO:0006816	calcium ion transport	9/100	409/18862
BP	GO:0071375	cellular response to peptide hormone stimulus	8/100	325/18862
BP	GO:0043534	blood vessel endothelial cell migration	6/100	175/18862
BP	GO:0052126	movement in host environment	6/100	175/18862
BP	GO:0033598	mammary gland epithelial cell proliferation	3/100	26/18862
BP	GO:0060352	cell adhesion molecule production	3/100	26/18862
BP	GO:0070920	regulation of production of small RNA involved in gene silencing by RNA	3/100	26/18862
BP	GO:1900739	regulation of protein insertion into mitochondrial membrane involved in apoptotic signaling pathway	3/100	26/18862

BP	GO:1900740	positive regulation of protein insertion into mitochondrial membrane involved in apoptotic signaling pathway	3/100	26/18862
BP	GO:0048662	negative regulation of smooth muscle cell proliferation	4/100	63/18862
BP	GO:0033157	regulation of intracellular protein transport	7/100	248/18862
BP	GO:0006898	receptor-mediated endocytosis	8/100	328/18862
BP	GO:1903706	regulation of hemopoiesis	9/100	415/18862
BP	GO:1901796	regulation of signal transduction by p53 class mediator	6/100	177/18862
BP	GO:0045165	cell fate commitment	7/100	251/18862
BP	GO:0002360	T cell lineage commitment	3/100	27/18862
BP	GO:0007263	nitric oxide mediated signal transduction	3/100	27/18862
BP	GO:0071479	cellular response to ionizing radiation	4/100	65/18862
BP	GO:2000573	positive regulation of DNA biosynthetic process	4/100	65/18862
BP	GO:0071347	cellular response to interleukin-	6/100	180/18862
BP	GO:0002761	regulation of myeloid leukocyte differentiation	5/100	118/18862
BP	GO:0003014	renal system process	5/100	118/18862
BP	GO:0018107	peptidyl-threonine phosphorylation	5/100	118/18862
BP	GO:0051101	regulation of DNA binding	5/100	118/18862
BP	GO:0045600	positive regulation of fat cell differentiation	4/100	66/18862
BP	GO:0034766	negative regulation of ion transmembrane transport	5/100	119/18862
BP	GO:0007271	synaptic transmission,	3/100	28/18862
BP	GO:0060603	mammary gland duct morphogenesis	3/100	28/18862
BP	GO:0033555	multicellular organismal response to stress	4/100	67/18862
BP	GO:0050766	positive regulation of phagocytosis	4/100	67/18862
BP	GO:0050918	positive chemotaxis	4/100	67/18862
BP	GO:1901224	positive regulation of NIK/NF-kappaB signaling	4/100	67/18862
BP	GO:1905953	negative regulation of lipid localization	4/100	67/18862
BP	GO:0034605	cellular response to heat	5/100	120/18862
BP	GO:0034763	negative regulation of transmembrane transport	5/100	120/18862
BP	GO:1904062	regulation of cation transmembrane transport	8/100	340/18862
BP	GO:1903201	regulation of oxidative stress-induced cell death	4/100	68/18862
BP	GO:0001763	morphogenesis of a branching structure	6/100	186/18862
BP	GO:0007219	Notch signaling pathway	6/100	186/18862
BP	GO:0044282	small molecule catabolic process	9/100	431/18862
BP	GO:0071549	cellular response to dexamethasone stimulus	3/100	29/18862

BP	GO:0050688	regulation of defense response to virus	4/100	69/18862
BP	GO:0022408	negative regulation of cell-cell adhesion	6/100	189/18862
BP	GO:0071621	granulocyte chemotaxis	5/100	124/18862
BP	GO:0001844	protein insertion into mitochondrial membrane involved in apoptotic signaling pathway	3/100	30/18862
BP	GO:0034694	response to prostaglandin	3/100	30/18862
BP	GO:0055022	negative regulation of cardiac muscle tissue growth	3/100	30/18862
BP	GO:0061117	negative regulation of heart growth	3/100	30/18862
BP	GO:1902230	negative regulation of intrinsic apoptotic signaling pathway in response to DNA damage	3/100	30/18862
BP	GO:0051051	negative regulation of transport	9/100	438/18862
BP	GO:0033673	negative regulation of kinase activity	7/100	267/18862
BP	GO:0022898	regulation of transmembrane transporter activity	7/100	268/18862
BP	GO:0018210	peptidyl-threonine modification	5/100	127/18862
BP	GO:0014855	striated muscle cell proliferation	4/100	72/18862
BP	GO:0043154	negative regulation of cysteine-type endopeptidase activity involved in apoptotic process	4/100	72/18862
BP	GO:1903036	positive regulation of response to wounding	4/100	72/18862
BP	GO:0002230	positive regulation of defense response to virus by host	3/100	31/18862
BP	GO:0010644	cell communication by electrical coupling	3/100	31/18862
BP	GO:0048147	negative regulation of fibroblast proliferation	3/100	31/18862
BP	GO:0097421	liver regeneration	3/100	31/18862
BP	GO:1901797	negative regulation of signal transduction by p53 class mediator	3/100	31/18862
BP	GO:2000193	positive regulation of fatty acid transport	3/100	31/18862
BP	GO:1901215	negative regulation of neuron death	6/100	195/18862
BP	GO:0019079	viral genome replication	5/100	129/18862
BP	GO:0050714	positive regulation of protein secretion	5/100	129/18862
BP	GO:0043903	regulation of biological process involved in symbiotic interaction	6/100	197/18862
BP	GO:0007178	transmembrane receptor protein serine/threonine kinase signaling pathway	8/100	358/18862
BP	GO:0046825	regulation of protein export from nucleus	3/100	32/18862
BP	GO:0051385	response to mineralocorticoid	3/100	32/18862
BP	GO:0071312	cellular response to alkaloid	3/100	32/18862
BP	GO:0071260	cellular response to mechanical stimulus	4/100	74/18862
BP	GO:0034504	protein localization to nucleus	7/100	275/18862

BP	GO:0007292	female gamete generation	5/100	131/18862
BP	GO:0050890	cognition	7/100	276/18862
BP	GO:0046902	regulation of mitochondrial membrane permeability	4/100	75/18862
BP	GO:0050866	negative regulation of cell activation	6/100	200/18862
BP	GO:0010595	positive regulation of endothelial cell migration	5/100	132/18862
BP	GO:0001662	behavioral fear response	3/100	33/18862
BP	GO:0042573	retinoic acid metabolic process	3/100	33/18862
BP	GO:0051973	positive regulation of telomerase activity	3/100	33/18862
BP	GO:0009306	protein secretion	8/100	367/18862
BP	GO:0045862	positive regulation of proteolysis	8/100	367/18862
BP	GO:0007249	I-kappaB kinase/NF-kappaB signaling	7/100	282/18862
BP	GO:0035592	establishment of protein localization to extracellular region	8/100	368/18862
BP	GO:0002209	behavioral defense response	3/100	34/18862
BP	GO:0010543	regulation of platelet activation	3/100	34/18862
BP	GO:0043516	regulation of DNA damage response, signal transduction by p53 class mediator	3/100	34/18862
BP	GO:0016579	protein deubiquitination	7/100	283/18862
BP	GO:0045913	positive regulation of carbohydrate metabolic process	4/100	78/18862
BP	GO:0034754	cellular hormone metabolic process	5/100	136/18862
BP	GO:0045598	regulation of fat cell differentiation	5/100	136/18862
BP	GO:0046942	carboxylic acid transport	7/100	284/18862
BP	GO:0070555	response to interleukin-1	6/100	206/18862
BP	GO:0034764	positive regulation of transmembrane transport	6/100	207/18862
BP	GO:0034767	positive regulation of ion transmembrane transport	6/100	207/18862
BP	GO:0001776	leukocyte homeostasis	4/100	79/18862
BP	GO:0001892	embryonic placenta development	4/100	79/18862
BP	GO:0000083	regulation of transcription involved in G1/S transition of mitotic cell cycle	3/100	35/18862
BP	GO:0006739	NADP metabolic process	3/100	35/18862
BP	GO:0048009	insulin-like growth factor receptor signaling pathway	3/100	35/18862
BP	GO:1901030	positive regulation of mitochondrial outer membrane permeabilization involved in apoptotic signaling pathway	3/100	35/18862
BP	GO:0048738	cardiac muscle tissue development	6/100	208/18862
BP	GO:0071692	protein localization to extracellular region	8/100	375/18862
BP	GO:2000117	negative regulation of cysteine-type endopeptidase activity	4/100	80/18862
BP	GO:0090276	regulation of peptide hormone secretion	6/100	209/18862

BP	GO:0070374	positive regulation of ERK1 and ERK2 cascade	6/100	210/18862
BP	GO:1903522	regulation of blood circulation	7/100	290/18862
BP	GO:0046718	viral entry into host cell	5/100	140/18862
BP	GO:0014912	negative regulation of smooth muscle cell migration	3/100	36/18862
BP	GO:0030224	monocyte differentiation	3/100	36/18862
BP	GO:0032892	positive regulation of organic acid transport	3/100	36/18862
BP	GO:0042311	vasodilation	3/100	36/18862
BP	GO:0071392	cellular response to estradiol stimulus	3/100	36/18862
BP	GO:2000144	positive regulation of DNA-templated transcription,	3/100	36/18862
BP	GO:1904064	positive regulation of cation transmembrane transport	5/100	141/18862
BP	GO:0006970	response to osmotic stress	4/100	82/18862
BP	GO:0051604	protein maturation	7/100	293/18862
BP	GO:0072655	establishment of protein localization to mitochondrion	5/100	142/18862
BP	GO:0042596	fear response	3/100	37/18862
BP	GO:0045740	positive regulation of DNA replication	3/100	37/18862
BP	GO:0046621	negative regulation of organ growth	3/100	37/18862
BP	GO:0060969	negative regulation of gene silencing	3/100	37/18862
BP	GO:1902229	regulation of intrinsic apoptotic signaling pathway in response to DNA damage	3/100	37/18862
BP	GO:0010507	negative regulation of autophagy	4/100	83/18862
BP	GO:0045069	regulation of viral genome replication	4/100	83/18862
BP	GO:0090287	regulation of cellular response to growth factor stimulus	7/100	296/18862
BP	GO:0140014	mitotic nuclear division	7/100	296/18862
BP	GO:0007006	mitochondrial membrane organization	5/100	144/18862
BP	GO:0035051	cardiocyte differentiation	5/100	144/18862
BP	GO:1900407	regulation of cellular response to oxidative stress	4/100	84/18862
BP	GO:0002262	myeloid cell homeostasis	5/100	145/18862
BP	GO:0008210	estrogen metabolic process	3/100	38/18862
BP	GO:0010742	macrophage derived foam cell differentiation	3/100	38/18862
BP	GO:0032885	regulation of polysaccharide biosynthetic process	3/100	38/18862
BP	GO:0090077	foam cell differentiation	3/100	38/18862
BP	GO:0070585	protein localization to mitochondrion	5/100	146/18862
BP	GO:0046889	positive regulation of lipid biosynthetic process	4/100	85/18862
BP	GO:0050829	defense response to Gram-negative bacterium	4/100	85/18862
BP	GO:1900182	positive regulation of protein localization to nucleus	4/100	85/18862

BP	GO:0070646	protein modification by small protein removal	7/100	300/18862
BP	GO:0048285	organelle fission	9/100	486/18862
BP	GO:0007519	skeletal muscle tissue development	5/100	147/18862
BP	GO:0016202	regulation of striated muscle tissue development	3/100	39/18862
BP	GO:0016572	histone phosphorylation	3/100	39/18862
BP	GO:0097530	granulocyte migration	5/100	148/18862
BP	GO:0009743	response to carbohydrate	6/100	221/18862
BP	GO:0031284	positive regulation of guanylate cyclase activity	2/100	10/18862
BP	GO:0031652	positive regulation of heat generation	2/100	10/18862
BP	GO:0032070	regulation of deoxyribonuclease activity	2/100	10/18862
BP	GO:0033327	Leydig cell differentiation	2/100	10/18862
BP	GO:0042135	neurotransmitter catabolic process	2/100	10/18862
BP	GO:0051918	negative regulation of	2/100	10/18862
BP	GO:0060736	prostate gland growth	2/100	10/18862
BP	GO:0072203	cell proliferation involved in metanephros development	2/100	10/18862
BP	GO:0090066	regulation of anatomical structure size	9/100	493/18862
BP	GO:0002532	production of molecular mediator involved in inflammatory response	4/100	88/18862
BP	GO:0030890	positive regulation of B cell proliferation	3/100	40/18862
BP	GO:0002793	positive regulation of peptide secretion	5/100	151/18862
BP	GO:0045732	positive regulation of protein catabolic process	6/100	225/18862
BP	GO:0032869	cellular response to insulin stimulus	6/100	226/18862
BP	GO:0002790	peptide secretion	8/100	401/18862
BP	GO:0002251	organ or tissue specific immune response	3/100	41/18862
BP	GO:0010907	positive regulation of glucose metabolic process	3/100	41/18862
BP	GO:0033574	response to testosterone	3/100	41/18862
BP	GO:0050691	regulation of defense response to virus by host	3/100	41/18862
BP	GO:1901861	regulation of muscle tissue development	3/100	41/18862
BP	GO:1904646	cellular response to amyloid-beta	3/100	41/18862
BP	GO:0016236	macroautophagy	7/100	311/18862
BP	GO:0003073	regulation of systemic arterial blood pressure	4/100	90/18862
BP	GO:0014068	positive regulation of phosphatidylinositol 3-kinase signaling	4/100	90/18862
BP	GO:0090559	regulation of membrane permeability	4/100	90/18862
BP	GO:0000187	activation of MAPK activity	5/100	154/18862

BP	GO:1902107	positive regulation of leukocyte differentiation	5/100	154/18862
BP	GO:1903708	positive regulation of hemopoiesis	5/100	154/18862
BP	GO:0042593	glucose homeostasis	6/100	229/18862
BP	GO:0050804	modulation of chemical synaptic transmission	8/100	405/18862
BP	GO:0033500	carbohydrate homeostasis	6/100	230/18862
BP	GO:0044242	cellular lipid catabolic process	6/100	230/18862
BP	GO:0099177	regulation of trans-synaptic signaling	8/100	406/18862
BP	GO:0014075	response to amine	3/100	42/18862
BP	GO:0032965	regulation of collagen biosynthetic process	3/100	42/18862
BP	GO:0071364	cellular response to epidermal growth factor stimulus	3/100	42/18862
BP	GO:0010594	regulation of endothelial cell migration	6/100	231/18862
BP	GO:0006527	arginine catabolic process	2/100	11/18862
BP	GO:0010623	programmed cell death involved in cell development	2/100	11/18862
BP	GO:0033197	response to vitamin E	2/100	11/18862
BP	GO:0045899	positive regulation of RNA polymerase II transcription preinitiation complex assembly	2/100	11/18862
BP	GO:0051974	negative regulation of telomerase activity	2/100	11/18862
BP	GO:0030168	platelet activation	5/100	157/18862
BP	GO:0060538	skeletal muscle organ development	5/100	157/18862
BP	GO:0014013	regulation of gliogenesis	4/100	93/18862
BP	GO:0051591	response to cAMP	4/100	93/18862
BP	GO:1902882	regulation of response to oxidative stress	4/100	93/18862
BP	GO:0022602	ovulation cycle process	3/100	43/18862
BP	GO:0046006	regulation of activated T cell proliferation	3/100	43/18862
BP	GO:0048599	oocyte development	3/100	43/18862
BP	GO:0048634	regulation of muscle organ development	3/100	43/18862
BP	GO:0070266	necroptotic process	3/100	43/18862
BP	GO:2000142	regulation of DNA-templated transcription, initiation	3/100	43/18862
BP	GO:0043271	negative regulation of ion transport	7/100	319/18862
BP	GO:0048568	embryonic organ development	8/100	412/18862
BP	GO:0034308	primary alcohol metabolic process	4/100	94/18862
BP	GO:0050764	regulation of phagocytosis	4/100	94/18862
BP	GO:0032680	regulation of tumor necrosis factor production	5/100	160/18862
BP	GO:0060711	labyrinthine layer development	3/100	44/18862
BP	GO:1901028	regulation of mitochondrial outer membrane permeabilization involved in apoptotic signaling pathway	3/100	44/18862
BP	GO:0010522	regulation of calcium ion transport into cytosol	4/100	95/18862

BP	GO:0030307	positive regulation of cell	5/100	161/18862
BP	GO:0043255	regulation of carbohydrate biosynthetic process	4/100	96/18862
BP	GO:0048010	vascular endothelial growth factor receptor signaling pathway	4/100	96/18862
BP	GO:1901184	regulation of ERBB signaling pathway	4/100	96/18862
BP	GO:0032640	tumor necrosis factor	5/100	162/18862
BP	GO:0006775	fat-soluble vitamin metabolic process	3/100	45/18862
BP	GO:0032881	regulation of polysaccharide metabolic process	3/100	45/18862
BP	GO:0048538	thymus development	3/100	45/18862
BP	GO:0034250	positive regulation of cellular amide metabolic process	5/100	163/18862
BP	GO:0032677	regulation of interleukin-8 production	4/100	97/18862
BP	GO:0045807	positive regulation of endocytosis	4/100	97/18862
BP	GO:0007077	mitotic nuclear envelope disassembly	2/100	12/18862
BP	GO:0033629	negative regulation of cell adhesion mediated by integrin	2/100	12/18862
BP	GO:0042178	xenobiotic catabolic process	2/100	12/18862
BP	GO:0051549	positive regulation of keratinocyte migration	2/100	12/18862
BP	GO:0061052	negative regulation of cell growth involved in cardiac muscle cell development	2/100	12/18862
BP	GO:0071107	response to parathyroid hormone	2/100	12/18862
BP	GO:0072584	caveolin-mediated endocytosis	2/100	12/18862
BP	GO:1903800	positive regulation of production of miRNAs involved in gene silencing by miRNA	2/100	12/18862
BP	GO:2001171	positive regulation of ATP biosynthetic process	2/100	12/18862
BP	GO:1903555	regulation of tumor necrosis factor superfamily cytokine production	5/100	164/18862
BP	GO:0031341	regulation of cell killing	4/100	98/18862
BP	GO:0002686	negative regulation of leukocyte migration	3/100	46/18862
BP	GO:0007595	lactation	3/100	46/18862
BP	GO:0010823	negative regulation of mitochondrion organization	3/100	46/18862
BP	GO:0050798	activated T cell proliferation	3/100	46/18862
BP	GO:0050873	brown fat cell differentiation	3/100	46/18862
BP	GO:0071354	cellular response to interleukin-	3/100	46/18862
BP	GO:0101023	vascular endothelial cell proliferation	3/100	46/18862
BP	GO:1905562	regulation of vascular endothelial cell proliferation	3/100	46/18862
BP	GO:0046395	carboxylic acid catabolic process	6/100	243/18862
BP	GO:0010951	negative regulation of endopeptidase activity	6/100	245/18862

BP	GO:0071706	tumor necrosis factor superfamily cytokine production	5/100	167/18862
BP	GO:0045833	negative regulation of lipid metabolic process	4/100	100/18862
BP	GO:1903076	regulation of protein localization to plasma membrane	4/100	100/18862
BP	GO:0009994	oocyte differentiation	3/100	47/18862
BP	GO:0010712	regulation of collagen metabolic process	3/100	47/18862
BP	GO:1903053	regulation of extracellular matrix organization	3/100	47/18862
BP	GO:0030072	peptide hormone secretion	6/100	247/18862
BP	GO:0001701	in utero embryonic	7/100	335/18862
BP	GO:0002674	negative regulation of acute inflammatory response	2/100	13/18862
BP	GO:0010745	negative regulation of macrophage derived foam cell differentiation	2/100	13/18862
BP	GO:0031282	regulation of guanylate cyclase activity	2/100	13/18862
BP	GO:0031392	regulation of prostaglandin biosynthetic process	2/100	13/18862
BP	GO:0033127	regulation of histone phosphorylation	2/100	13/18862
BP	GO:0043568	positive regulation of insulin-like growth factor receptor signaling pathway	2/100	13/18862
BP	GO:0072683	T cell extravasation	2/100	13/18862
BP	GO:1902947	regulation of tau-protein kinase activity	2/100	13/18862
BP	GO:0051099	positive regulation of binding	5/100	170/18862
BP	GO:0045639	positive regulation of myeloid cell differentiation	4/100	102/18862
BP	GO:0071887	leukocyte apoptotic process	4/100	102/18862
BP	GO:0051302	regulation of cell division	5/100	171/18862
BP	GO:0032526	response to retinoic acid	4/100	103/18862
BP	GO:0046822	regulation of nucleocytoplasmic transport	4/100	103/18862
BP	GO:0000280	nuclear division	8/100	436/18862
BP	GO:0060043	regulation of cardiac muscle cell proliferation	3/100	49/18862
BP	GO:0070231	T cell apoptotic process	3/100	49/18862
BP	GO:0019395	fatty acid oxidation	4/100	105/18862
BP	GO:0062207	regulation of pattern recognition receptor signaling pathway	4/100	105/18862
BP	GO:0070741	response to interleukin-6	3/100	50/18862
BP	GO:0010649	regulation of cell communication by electrical	2/100	14/18862
BP	GO:0010889	regulation of sequestering of triglyceride	2/100	14/18862
BP	GO:0030213	hyaluronan biosynthetic process	2/100	14/18862
BP	GO:0032966	negative regulation of collagen biosynthetic process	2/100	14/18862
BP	GO:0036295	cellular response to increased oxygen levels	2/100	14/18862
BP	GO:0045898	regulation of RNA polymerase II transcription preinitiation complex assembly	2/100	14/18862

BP	GO:0045986	negative regulation of smooth muscle contraction	2/100	14/18862
BP	GO:0051547	regulation of keratinocyte migration	2/100	14/18862
BP	GO:0051917	regulation of fibrinolysis	2/100	14/18862
BP	GO:0097284	hepatocyte apoptotic process	2/100	14/18862
BP	GO:1901722	regulation of cell proliferation involved in kidney development	2/100	14/18862
BP	GO:1902894	negative regulation of pri-miRNA transcription by RNA polymerase II	2/100	14/18862
BP	GO:1901222	regulation of NIK/NF-kappaB signaling	4/100	106/18862
BP	GO:0010466	negative regulation of peptidase activity	6/100	257/18862
BP	GO:0032964	collagen biosynthetic process	3/100	51/18862
BP	GO:0097300	programmed necrotic cell death	3/100	51/18862
BP	GO:0016054	organic acid catabolic process	6/100	258/18862
BP	GO:0045637	regulation of myeloid cell differentiation	6/100	258/18862
BP	GO:2001257	regulation of cation channel activity	5/100	177/18862
BP	GO:0050796	regulation of insulin secretion	5/100	178/18862
BP	GO:0032386	regulation of intracellular transport	7/100	350/18862
BP	GO:0001938	positive regulation of endothelial cell proliferation	4/100	108/18862
BP	GO:0046883	regulation of hormone secretion	6/100	260/18862
BP	GO:0051607	defense response to virus	6/100	260/18862
BP	GO:0140546	defense response to symbiont	6/100	260/18862
BP	GO:0035196	production of miRNAs involved in gene silencing by miRNA	3/100	52/18862
BP	GO:0001906	cell killing	5/100	179/18862
BP	GO:0007369	gastrulation	5/100	179/18862
BP	GO:1904659	glucose transmembrane transport	4/100	109/18862
BP	GO:0032872	regulation of stress-activated MAPK cascade	5/100	181/18862
BP	GO:0002070	epithelial cell maturation	2/100	15/18862
BP	GO:0002827	positive regulation of T-helper 1 type immune response	2/100	15/18862
BP	GO:0009404	toxin metabolic process	2/100	15/18862
BP	GO:0010713	negative regulation of collagen metabolic process	2/100	15/18862
BP	GO:0016114	terpenoid biosynthetic process	2/100	15/18862
BP	GO:0030397	membrane disassembly	2/100	15/18862
BP	GO:0035635	entry of bacterium into host cell	2/100	15/18862
BP	GO:0042574	retinal metabolic process	2/100	15/18862
BP	GO:0042976	activation of Janus kinase activity	2/100	15/18862
BP	GO:0043518	negative regulation of DNA damage response, signal transduction by p53 class mediator	2/100	15/18862
BP	GO:0048308	organelle inheritance	2/100	15/18862
BP	GO:0048313	Golgi inheritance	2/100	15/18862
BP	GO:0051081	nuclear envelope disassembly	2/100	15/18862
BP	GO:0051770	positive regulation of nitric-oxide synthase biosynthetic	2/100	15/18862

BP	GO:0071380	cellular response to prostaglandin E stimulus	2/100	15/18862
BP	GO:0071391	cellular response to estrogen stimulus	2/100	15/18862
BP	GO:0072075	metanephric mesenchyme development	2/100	15/18862
BP	GO:1902166	negative regulation of intrinsic apoptotic signaling pathway in response to DNA damage by p53 class mediator	2/100	15/18862
BP	GO:1903799	negative regulation of production of miRNAs involved in gene silencing by miRNA	2/100	15/18862
BP	GO:2000402	negative regulation of lymphocyte migration	2/100	15/18862
BP	GO:2001028	positive regulation of endothelial cell chemotaxis	2/100	15/18862
BP	GO:2001279	regulation of unsaturated fatty acid biosynthetic process	2/100	15/18862
BP	GO:0034440	lipid oxidation	4/100	110/18862
BP	GO:0006984	ER-nucleus signaling pathway	3/100	53/18862
BP	GO:0009165	nucleotide biosynthetic process	6/100	264/18862
BP	GO:0002286	T cell activation involved in immune response	4/100	111/18862
BP	GO:0010676	positive regulation of cellular carbohydrate metabolic process	3/100	54/18862
BP	GO:0086002	cardiac muscle cell action potential involved in contraction	3/100	54/18862
BP	GO:0008645	hexose transmembrane transport	4/100	112/18862
BP	GO:0070302	regulation of stress-activated protein kinase signaling cascade	5/100	184/18862
BP	GO:1901293	nucleoside phosphate biosynthetic process	6/100	267/18862
BP	GO:0031589	cell-substrate adhesion	7/100	359/18862
BP	GO:0006986	response to unfolded protein	5/100	185/18862
BP	GO:0009749	response to glucose	5/100	185/18862
BP	GO:0038061	NIK/NF-kappaB signaling	5/100	185/18862
BP	GO:0031349	positive regulation of defense response	7/100	361/18862
BP	GO:0071385	cellular response to glucocorticoid stimulus	3/100	55/18862
BP	GO:0097345	mitochondrial outer membrane permeabilization	3/100	55/18862
BP	GO:0050792	regulation of viral process	5/100	186/18862
BP	GO:0030258	lipid modification	6/100	270/18862
BP	GO:0015749	monosaccharide transmembrane transport	4/100	114/18862
BP	GO:0006098	pentose-phosphate shunt	2/100	16/18862
BP	GO:0010224	response to UV-B	2/100	16/18862
BP	GO:0019372	lipoxygenase pathway	2/100	16/18862
BP	GO:0030728	ovulation	2/100	16/18862
BP	GO:0034116	positive regulation of heterotypic cell-cell adhesion	2/100	16/18862
BP	GO:0045725	positive regulation of glycogen biosynthetic process	2/100	16/18862
BP	GO:0051709	regulation of killing of cells of other organism	2/100	16/18862

BP	GO:1903209	positive regulation of oxidative stress-induced cell death	2/100	16/18862
BP	GO:0010883	regulation of lipid storage	3/100	56/18862
BP	GO:0031050	dsRNA processing	3/100	56/18862
BP	GO:0043388	positive regulation of DNA binding	3/100	56/18862
BP	GO:0070918	production of small RNA involved in gene silencing by RNA	3/100	56/18862
BP	GO:0001704	formation of primary germ layer	4/100	115/18862
BP	GO:0007009	plasma membrane organization	4/100	115/18862
BP	GO:0030218	erythrocyte differentiation	4/100	115/18862
BP	GO:1903510	mucopolysaccharide metabolic process	4/100	115/18862
BP	GO:0002697	regulation of immune effector process	8/100	465/18862
BP	GO:0033044	regulation of chromosome organization	6/100	273/18862
BP	GO:0034219	carbohydrate transmembrane transport	4/100	116/18862
BP	GO:0006694	steroid biosynthetic process	5/100	190/18862
BP	GO:0009746	response to hexose	5/100	190/18862
BP	GO:0030520	intracellular estrogen receptor signaling pathway	3/100	57/18862
BP	GO:0031295	T cell costimulation	3/100	57/18862
BP	GO:0048008	platelet-derived growth factor receptor signaling pathway	3/100	57/18862
BP	GO:0086065	cell communication involved in cardiac conduction	3/100	57/18862
BP	GO:0010544	negative regulation of platelet activation	2/100	17/18862
BP	GO:0043217	myelin maintenance	2/100	17/18862
BP	GO:0050665	hydrogen peroxide biosynthetic process	2/100	17/18862
BP	GO:0071850	mitotic cell cycle arrest	2/100	17/18862
BP	GO:0090201	negative regulation of release of cytochrome c from	2/100	17/18862
BP	GO:1902165	regulation of intrinsic apoptotic signaling pathway in response to DNA damage by p53 class mediator	2/100	17/18862
BP	GO:0042752	regulation of circadian rhythm	4/100	118/18862
BP	GO:0043500	muscle adaptation	4/100	118/18862
BP	GO:0051928	positive regulation of calcium ion transport	4/100	118/18862
BP	GO:0002763	positive regulation of myeloid leukocyte differentiation	3/100	58/18862
BP	GO:0031638	zymogen activation	3/100	58/18862
BP	GO:0060038	cardiac muscle cell proliferation	3/100	58/18862
BP	GO:1903749	positive regulation of establishment of protein localization to mitochondrion	3/100	58/18862
BP	GO:0050864	regulation of B cell activation	5/100	193/18862
BP	GO:0050868	negative regulation of T cell activation	4/100	119/18862
BP	GO:0017038	protein import	5/100	194/18862
BP	GO:0023061	signal release	8/100	475/18862
BP	GO:0001822	kidney development	6/100	280/18862

BP	GO:0006260	DNA replication	6/100	280/18862
BP	GO:0006940	regulation of smooth muscle contraction	3/100	59/18862
BP	GO:1900542	regulation of purine nucleotide metabolic process	4/100	120/18862
BP	GO:1901888	regulation of cell junction assembly	5/100	195/18862
BP	GO:0015711	organic anion transport	7/100	376/18862
BP	GO:0034284	response to monosaccharide	5/100	196/18862
BP	GO:0034101	erythrocyte homeostasis	4/100	121/18862
BP	GO:0060964	regulation of gene silencing by miRNA	4/100	121/18862
BP	GO:1904375	regulation of protein localization to cell periphery	4/100	121/18862
BP	GO:0016239	positive regulation of macroautophagy	3/100	60/18862
BP	GO:1902110	positive regulation of mitochondrial membrane permeability involved in apoptotic process	3/100	60/18862
BP	GO:0006740	NADPH regeneration	2/100	18/18862
BP	GO:0032026	response to magnesium ion	2/100	18/18862
BP	GO:0032930	positive regulation of superoxide anion generation	2/100	18/18862
BP	GO:0035743	CD4-positive, alpha-beta T cell cytokine production	2/100	18/18862
BP	GO:0060252	positive regulation of glial cell proliferation	2/100	18/18862
BP	GO:0060716	labyrinthine layer blood vessel development	2/100	18/18862
BP	GO:0070293	renal absorption	2/100	18/18862
BP	GO:0070875	positive regulation of glycogen metabolic process	2/100	18/18862
BP	GO:2000696	regulation of epithelial cell differentiation involved in kidney development	2/100	18/18862
BP	GO:0006140	regulation of nucleotide metabolic process	4/100	122/18862
BP	GO:0019722	calcium-mediated signaling	5/100	198/18862
BP	GO:0006909	phagocytosis	7/100	381/18862
BP	GO:0070059	intrinsic apoptotic signaling pathway in response to endoplasmic reticulum stress	3/100	61/18862
BP	GO:1905330	regulation of morphogenesis of an epithelium	3/100	61/18862
BP	GO:0035821	modulation of process of other organism	4/100	123/18862
BP	GO:0010952	positive regulation of peptidase activity	5/100	200/18862
BP	GO:0060284	regulation of cell development	8/100	485/18862
BP	GO:0051053	negative regulation of DNA metabolic process	4/100	124/18862
BP	GO:0060147	regulation of posttranscriptional gene silencing	4/100	124/18862
BP	GO:0001501	skeletal system development	8/100	486/18862
BP	GO:0072001	renal system development	6/100	288/18862

BP	GO:1902686	mitochondrial outer membrane permeabilization involved in programmed cell death	3/100	62/18862
BP	GO:0001952	regulation of cell-matrix adhesion	4/100	125/18862
BP	GO:0060966	regulation of gene silencing by RNA	4/100	125/18862
BP	GO:0007179	transforming growth factor beta receptor signaling pathway	5/100	202/18862
BP	GO:0010720	positive regulation of cell development	6/100	290/18862
BP	GO:0002689	negative regulation of leukocyte chemotaxis	2/100	19/18862
BP	GO:0048245	eosinophil chemotaxis	2/100	19/18862
BP	GO:0048643	positive regulation of skeletal muscle tissue development	2/100	19/18862
BP	GO:0051900	regulation of mitochondrial depolarization	2/100	19/18862
BP	GO:0072074	kidney mesenchyme development	2/100	19/18862
BP	GO:0035637	multicellular organismal	5/100	203/18862
BP	GO:0007272	ensheathment of neurons	4/100	126/18862
BP	GO:0008366	axon ensheathment	4/100	126/18862
BP	GO:0014015	positive regulation of	3/100	63/18862
BP	GO:0034248	regulation of cellular amide metabolic process	8/100	494/18862
BP	GO:0006749	glutathione metabolic process	3/100	64/18862
BP	GO:0032507	maintenance of protein location in cell	3/100	64/18862
BP	GO:0035794	positive regulation of mitochondrial membrane permeability	3/100	64/18862
BP	GO:0051148	negative regulation of muscle cell differentiation	3/100	64/18862
BP	GO:0045667	regulation of osteoblast differentiation	4/100	128/18862
BP	GO:0030100	regulation of endocytosis	5/100	206/18862
BP	GO:0035966	response to topologically incorrect protein	5/100	206/18862
BP	GO:0030073	insulin secretion	5/100	207/18862
BP	GO:0006525	arginine metabolic process	2/100	20/18862
BP	GO:0031998	regulation of fatty acid beta-oxidation	2/100	20/18862
BP	GO:0032928	regulation of superoxide anion generation	2/100	20/18862
BP	GO:0051767	nitric-oxide synthase biosynthetic process	2/100	20/18862
BP	GO:0051769	regulation of nitric-oxide synthase biosynthetic process	2/100	20/18862
BP	GO:1902004	positive regulation of amyloid-beta formation	2/100	20/18862
BP	GO:1902176	negative regulation of oxidative stress-induced intrinsic apoptotic signaling pathway	2/100	20/18862
BP	GO:0070265	necrotic cell death	3/100	65/18862
BP	GO:0072507	divalent inorganic cation homeostasis	8/100	499/18862
BP	GO:0006766	vitamin metabolic process	4/100	130/18862

BP	GO:1900180	regulation of protein localization to nucleus	4/100	130/18862
BP	GO:1903844	regulation of cellular response to transforming growth factor beta stimulus	4/100	130/18862
BP	GO:0016051	carbohydrate biosynthetic process	5/100	209/18862
BP	GO:0051651	maintenance of location in cell	5/100	209/18862
BP	GO:0042130	negative regulation of T cell proliferation	3/100	66/18862
BP	GO:1902108	regulation of mitochondrial membrane permeability involved in apoptotic process	3/100	66/18862
BP	GO:2000272	negative regulation of signaling receptor activity	3/100	66/18862
BP	GO:0031640	killing of cells of other organism	3/100	67/18862
BP	GO:0032922	circadian regulation of gene expression	3/100	67/18862
BP	GO:0046879	hormone secretion	6/100	302/18862
BP	GO:0002053	positive regulation of mesenchymal cell proliferation	2/100	21/18862
BP	GO:0002363	alpha-beta T cell lineage commitment	2/100	21/18862
BP	GO:0010875	positive regulation of cholesterol efflux	2/100	21/18862
BP	GO:0045655	regulation of monocyte differentiation	2/100	21/18862
BP	GO:0061042	vascular wound healing	2/100	21/18862
BP	GO:2001169	regulation of ATP biosynthetic process	2/100	21/18862
BP	GO:0050729	positive regulation of inflammatory response	4/100	133/18862
BP	GO:0090101	negative regulation of transmembrane receptor protein serine/threonine kinase signaling pathway	4/100	134/18862
BP	GO:0002548	monocyte chemotaxis	3/100	68/18862
BP	GO:0045685	regulation of glial cell differentiation	3/100	68/18862
BP	GO:1900015	regulation of cytokine production involved in inflammatory response	3/100	68/18862
BP	GO:0022604	regulation of cell	6/100	305/18862
BP	GO:0043280	positive regulation of cysteine-type endopeptidase activity involved in apoptotic process	4/100	135/18862
BP	GO:0043524	negative regulation of neuron apoptotic process	4/100	135/18862
BP	GO:0060048	cardiac muscle contraction	4/100	135/18862
BP	GO:0002534	cytokine production involved in inflammatory response	3/100	69/18862
BP	GO:0046626	regulation of insulin receptor signaling pathway	3/100	69/18862
BP	GO:0072088	nephron epithelium morphogenesis	3/100	69/18862
BP	GO:1905710	positive regulation of membrane permeability	3/100	69/18862

BP	GO:0031281	positive regulation of cyclase activity	2/100	22/18862
BP	GO:0032069	regulation of nuclease activity	2/100	22/18862
BP	GO:0033081	regulation of T cell differentiation in thymus	2/100	22/18862
BP	GO:0035584	calcium-mediated signaling using intracellular calcium	2/100	22/18862
BP	GO:0042359	vitamin D metabolic process	2/100	22/18862
BP	GO:0043369	CD4-positive or CD8-positive, alpha-beta T cell lineage commitment	2/100	22/18862
BP	GO:0043457	regulation of cellular respiration	2/100	22/18862
BP	GO:0044346	fibroblast apoptotic process	2/100	22/18862
BP	GO:0045932	negative regulation of muscle contraction	2/100	22/18862
BP	GO:0051882	mitochondrial depolarization	2/100	22/18862
BP	GO:1901522	positive regulation of transcription from RNA polymerase II promoter involved in cellular response to chemical stimulus	2/100	22/18862
BP	GO:0003158	endothelium development	4/100	137/18862
BP	GO:0010810	regulation of cell-substrate adhesion	5/100	218/18862
BP	GO:0042445	hormone metabolic process	5/100	218/18862
BP	GO:0007204	positive regulation of cytosolic calcium ion concentration	6/100	310/18862
BP	GO:0001889	liver development	4/100	138/18862
BP	GO:0072006	nephron development	4/100	138/18862
BP	GO:0009914	hormone transport	6/100	312/18862
BP	GO:0033692	cellular polysaccharide biosynthetic process	3/100	71/18862
BP	GO:0070227	lymphocyte apoptotic process	3/100	71/18862
BP	GO:0072028	nephron morphogenesis	3/100	71/18862
BP	GO:0010640	regulation of platelet-derived growth factor receptor signaling pathway	2/100	23/18862
BP	GO:0034695	response to prostaglandin E	2/100	23/18862
BP	GO:0036499	PERK-mediated unfolded protein response	2/100	23/18862
BP	GO:0045723	positive regulation of fatty acid biosynthetic process	2/100	23/18862
BP	GO:0051349	positive regulation of lyase activity	2/100	23/18862
BP	GO:0061050	regulation of cell growth involved in cardiac muscle cell development	2/100	23/18862
BP	GO:0071676	negative regulation of mononuclear cell migration	2/100	23/18862
BP	GO:0072677	eosinophil migration	2/100	23/18862
BP	GO:2000353	positive regulation of endothelial cell apoptotic	2/100	23/18862
BP	GO:0061008	hepaticobiliary system development	4/100	140/18862
BP	GO:0015909	long-chain fatty acid transport	3/100	72/18862
BP	GO:0035924	cellular response to vascular endothelial growth factor stimulus	3/100	72/18862

BP	GO:0048708	astrocyte differentiation	3/100	72/18862
BP	GO:0086003	cardiac muscle cell contraction	3/100	72/18862
BP	GO:1903747	regulation of establishment of protein localization to mitochondrion	3/100	72/18862
BP	GO:0031056	regulation of histone modification	4/100	141/18862
BP	GO:0007517	muscle organ development	6/100	317/18862
BP	GO:0031016	pancreas development	3/100	73/18862
BP	GO:0042246	tissue regeneration	3/100	73/18862
BP	GO:0044344	cellular response to fibroblast growth factor stimulus	4/100	142/18862
BP	GO:0002831	regulation of response to biotic stimulus	7/100	420/18862
BP	GO:0006606	protein import into nucleus	4/100	143/18862
BP	GO:0008286	insulin receptor signaling pathway	4/100	143/18862
BP	GO:0001556	oocyte maturation	2/100	24/18862
BP	GO:0007095	mitotic G2 DNA damage checkpoint	2/100	24/18862
BP	GO:0043567	regulation of insulin-like growth factor receptor signaling pathway	2/100	24/18862
BP	GO:0060669	embryonic placenta morphogenesis	2/100	24/18862
BP	GO:2001026	regulation of endothelial cell chemotaxis	2/100	24/18862
BP	GO:0050806	positive regulation of synaptic transmission	4/100	144/18862
BP	GO:0060968	regulation of gene silencing	4/100	144/18862
BP	GO:0006305	DNA alkylation	3/100	75/18862
BP	GO:0006306	DNA methylation	3/100	75/18862
BP	GO:0006635	fatty acid beta-oxidation	3/100	75/18862
BP	GO:0034329	cell junction assembly	7/100	425/18862
BP	GO:0045580	regulation of T cell	4/100	146/18862
BP	GO:0007160	cell-matrix adhesion	5/100	230/18862
BP	GO:0086001	cardiac muscle cell action potential	3/100	76/18862
BP	GO:0022011	myelination in peripheral nervous system	2/100	25/18862
BP	GO:0032292	peripheral nervous system axon ensheathment	2/100	25/18862
BP	GO:0048641	regulation of skeletal muscle tissue development	2/100	25/18862
BP	GO:0050995	negative regulation of lipid catabolic process	2/100	25/18862
BP	GO:0051123	RNA polymerase II preinitiation complex assembly	2/100	25/18862
BP	GO:0051156	glucose 6-phosphate metabolic process	2/100	25/18862
BP	GO:0060330	regulation of response to interferon-gamma	2/100	25/18862
BP	GO:0060334	regulation of interferon-gamma-mediated signaling pathway	2/100	25/18862
BP	GO:0060740	prostate gland epithelium morphogenesis	2/100	25/18862

BP	GO:0071459	protein localization to chromosome, centromeric region	2/100	25/18862
BP	GO:1902993	positive regulation of amyloid precursor protein catabolic process	2/100	25/18862
BP	GO:1905563	negative regulation of vascular endothelial cell proliferation	2/100	25/18862
BP	GO:1990000	amyloid fibril formation	2/100	25/18862
BP	GO:0048762	mesenchymal cell differentiation	5/100	231/18862
BP	GO:0007043	cell-cell junction assembly	4/100	147/18862
BP	GO:0007605	sensory perception of sound	4/100	147/18862
BP	GO:0042113	B cell activation	6/100	326/18862
BP	GO:0032481	positive regulation of type I interferon production	3/100	77/18862
BP	GO:0034121	regulation of toll-like receptor signaling pathway	3/100	77/18862
BP	GO:0055013	cardiac muscle cell development	3/100	77/18862
BP	GO:0071774	response to fibroblast growth factor	4/100	148/18862
BP	GO:1903900	regulation of viral life cycle	4/100	148/18862
BP	GO:0008643	carbohydrate transport	4/100	149/18862
BP	GO:0061337	cardiac conduction	4/100	149/18862
BP	GO:0000271	polysaccharide biosynthetic process	3/100	78/18862
BP	GO:0001570	vasculogenesis	3/100	78/18862
BP	GO:0050886	endocrine process	3/100	78/18862
BP	GO:0010464	regulation of mesenchymal cell proliferation	2/100	26/18862
BP	GO:0034114	regulation of heterotypic cell-cell adhesion	2/100	26/18862
BP	GO:0042104	positive regulation of activated T cell proliferation	2/100	26/18862
BP	GO:0048873	homeostasis of number of cells within a tissue	2/100	26/18862
BP	GO:2000810	regulation of bicellular tight junction assembly	2/100	26/18862
BP	GO:1903364	positive regulation of cellular protein catabolic process	4/100	150/18862
BP	GO:0008360	regulation of cell shape	4/100	151/18862
BP	GO:2001056	positive regulation of cysteine-type endopeptidase activity	4/100	151/18862
BP	GO:0060402	calcium ion transport into	4/100	152/18862
BP	GO:0045445	myoblast differentiation	3/100	80/18862
BP	GO:0051250	negative regulation of lymphocyte activation	4/100	153/18862
BP	GO:0002227	innate immune response in mucosa	2/100	27/18862
BP	GO:0002675	positive regulation of acute inflammatory response	2/100	27/18862
BP	GO:0002825	regulation of T-helper 1 type immune response	2/100	27/18862
BP	GO:0009065	glutamine family amino acid catabolic process	2/100	27/18862
BP	GO:0050996	positive regulation of lipid catabolic process	2/100	27/18862
BP	GO:0060512	prostate gland morphogenesis	2/100	27/18862

BP	GO:0071624	positive regulation of granulocyte chemotaxis	2/100	27/18862
BP	GO:0090200	positive regulation of release of cytochrome c from	2/100	27/18862
BP	GO:0140467	integrated stress response signaling	2/100	27/18862
BP	GO:2000463	positive regulation of excitatory postsynaptic potential	2/100	27/18862
BP	GO:0050672	negative regulation of lymphocyte proliferation	3/100	81/18862
BP	GO:0006874	cellular calcium ion homeostasis	7/100	442/18862
BP	GO:0001656	metanephros development	3/100	82/18862
BP	GO:0032945	negative regulation of mononuclear cell proliferation	3/100	82/18862
BP	GO:0006913	nucleocytoplasmic transport	6/100	340/18862
BP	GO:0061448	connective tissue development	5/100	243/18862
BP	GO:0001782	B cell homeostasis	2/100	28/18862
BP	GO:0008299	isoprenoid biosynthetic process	2/100	28/18862
BP	GO:0014044	Schwann cell development	2/100	28/18862
BP	GO:0062098	regulation of programmed necrotic cell death	2/100	28/18862
BP	GO:0086011	membrane repolarization during action potential	2/100	28/18862
BP	GO:0095500	acetylcholine receptor signaling pathway	2/100	28/18862
BP	GO:0043122	regulation of I-kappaB kinase/NF-kappaB signaling	5/100	244/18862
BP	GO:0055006	cardiac cell development	3/100	83/18862
BP	GO:0051169	nuclear transport	6/100	343/18862
BP	GO:0030203	glycosaminoglycan metabolic process	4/100	158/18862
BP	GO:0042742	defense response to bacterium	6/100	344/18862
BP	GO:0051480	regulation of cytosolic calcium ion concentration	6/100	344/18862
BP	GO:0051092	positive regulation of NF-kappaB transcription factor	4/100	159/18862
BP	GO:0051147	regulation of muscle cell differentiation	4/100	159/18862
BP	GO:2000241	regulation of reproductive process	4/100	159/18862
BP	GO:0040018	positive regulation of multicellular organism growth	2/100	29/18862
BP	GO:0042133	neurotransmitter metabolic process	2/100	29/18862
BP	GO:0060055	angiogenesis involved in wound healing	2/100	29/18862
BP	GO:0060261	positive regulation of transcription initiation from RNA polymerase II promoter	2/100	29/18862
BP	GO:0071168	protein localization to chromatin	2/100	29/18862
BP	GO:0072539	T-helper 17 cell differentiation	2/100	29/18862
BP	GO:1900027	regulation of ruffle assembly	2/100	29/18862
BP	GO:1900117	regulation of execution phase of apoptosis	2/100	29/18862
BP	GO:1901380	negative regulation of potassium ion transmembrane	2/100	29/18862
BP	GO:1903579	negative regulation of ATP metabolic process	2/100	29/18862

BP	GO:2000637	positive regulation of gene silencing by miRNA	2/100	29/18862
BP	GO:0042509	regulation of tyrosine phosphorylation of STAT protein	3/100	85/18862
BP	GO:0048477	oogenesis	3/100	85/18862
BP	GO:0055074	calcium ion homeostasis	7/100	454/18862
BP	GO:0000723	telomere maintenance	4/100	161/18862
BP	GO:0006937	regulation of muscle contraction	4/100	161/18862
BP	GO:0055067	monovalent inorganic cation homeostasis	4/100	161/18862
BP	GO:0051346	negative regulation of hydrolase activity	7/100	456/18862
BP	GO:0007589	body fluid secretion	3/100	86/18862
BP	GO:0030512	negative regulation of transforming growth factor beta receptor signaling pathway	3/100	86/18862
BP	GO:0031058	positive regulation of histone modification	3/100	86/18862
BP	GO:0032755	positive regulation of interleukin-6 production	3/100	86/18862
BP	GO:0034637	cellular carbohydrate biosynthetic process	3/100	86/18862
BP	GO:0051781	positive regulation of cell	3/100	86/18862
BP	GO:0090092	regulation of transmembrane receptor protein serine/threonine kinase signaling pathway	5/100	251/18862
BP	GO:1903362	regulation of cellular protein catabolic process	5/100	251/18862
BP	GO:0032635	interleukin-6 production	4/100	162/18862
BP	GO:0001975	response to amphetamine	2/100	30/18862
BP	GO:0005979	regulation of glycogen biosynthetic process	2/100	30/18862
BP	GO:0008209	androgen metabolic process	2/100	30/18862
BP	GO:0010962	regulation of glucan biosynthetic process	2/100	30/18862
BP	GO:0032743	positive regulation of interleukin-2 production	2/100	30/18862
BP	GO:0046685	response to arsenic-containing substance	2/100	30/18862
BP	GO:0051968	positive regulation of synaptic transmission, glutamatergic	2/100	30/18862
BP	GO:0060045	positive regulation of cardiac muscle cell proliferation	2/100	30/18862
BP	GO:0060148	positive regulation of posttranscriptional gene silencing	2/100	30/18862
BP	GO:0060674	placenta blood vessel development	2/100	30/18862
BP	GO:0070102	interleukin-6-mediated signaling pathway	2/100	30/18862
BP	GO:0090183	regulation of kidney development	2/100	30/18862
BP	GO:1905144	response to acetylcholine	2/100	30/18862
BP	GO:1905145	cellular response to	2/100	30/18862
BP	GO:0009308	amine metabolic process	4/100	163/18862
BP	GO:0051170	import into nucleus	4/100	163/18862

BP	GO:0006112	energy reserve metabolic process	3/100	87/18862
BP	GO:0032760	positive regulation of tumor necrosis factor production	3/100	87/18862
BP	GO:1904063	negative regulation of cation transmembrane transport	3/100	87/18862
BP	GO:0006936	muscle contraction	6/100	352/18862
BP	GO:2001252	positive regulation of chromosome organization	4/100	164/18862
BP	GO:0031334	positive regulation of protein-containing complex assembly	5/100	254/18862
BP	GO:0090150	establishment of protein localization to membrane	6/100	354/18862
BP	GO:0007260	tyrosine phosphorylation of STAT protein	3/100	88/18862
BP	GO:0032436	positive regulation of proteasomal ubiquitin-dependent protein catabolic process	3/100	88/18862
BP	GO:0060993	kidney morphogenesis	3/100	88/18862
BP	GO:0070664	negative regulation of leukocyte proliferation	3/100	88/18862
BP	GO:0072080	nephron tubule development	3/100	88/18862
BP	GO:0031330	negative regulation of cellular catabolic process	5/100	255/18862
BP	GO:0008544	epidermis development	7/100	463/18862
BP	GO:0003180	aortic valve morphogenesis	2/100	31/18862
BP	GO:0032373	positive regulation of sterol transport	2/100	31/18862
BP	GO:0032376	positive regulation of cholesterol transport	2/100	31/18862
BP	GO:0045879	negative regulation of smoothened signaling pathway	2/100	31/18862
BP	GO:1901976	regulation of cell cycle checkpoint	2/100	31/18862
BP	GO:0042058	regulation of epidermal growth factor receptor signaling pathway	3/100	89/18862
BP	GO:0046849	bone remodeling	3/100	89/18862
BP	GO:0032412	regulation of ion transmembrane transporter	5/100	257/18862
BP	GO:0050954	sensory perception of mechanical stimulus	4/100	167/18862
BP	GO:0046470	phosphatidylcholine metabolic process	3/100	90/18862
BP	GO:0061326	renal tubule development	3/100	90/18862
BP	GO:0070301	cellular response to hydrogen peroxide	3/100	90/18862
BP	GO:1903557	positive regulation of tumor necrosis factor superfamily cytokine production	3/100	90/18862
BP	GO:0055001	muscle cell development	4/100	168/18862
BP	GO:0010661	positive regulation of muscle cell apoptotic process	2/100	32/18862
BP	GO:0010743	regulation of macrophage derived foam cell differentiation	2/100	32/18862
BP	GO:0035767	endothelial cell chemotaxis	2/100	32/18862

BP	GO:0045736	negative regulation of cyclin-dependent protein serine/threonine kinase activity	2/100	32/18862
BP	GO:0048384	retinoic acid receptor signaling pathway	2/100	32/18862
BP	GO:0090022	regulation of neutrophil chemotaxis	2/100	32/18862
BP	GO:0090322	regulation of superoxide metabolic process	2/100	32/18862
BP	GO:0034502	protein localization to chromosome	3/100	91/18862
BP	GO:0007281	germ cell development	5/100	261/18862
BP	GO:0051403	stress-activated MAPK cascade	5/100	261/18862
BP	GO:0045185	maintenance of protein location	3/100	92/18862
BP	GO:0003298	physiological muscle hypertrophy	2/100	33/18862
BP	GO:0003301	physiological cardiac muscle hypertrophy	2/100	33/18862
BP	GO:0015721	bile acid and bile salt transport	2/100	33/18862
BP	GO:0032770	positive regulation of monooxygenase activity	2/100	33/18862
BP	GO:0055094	response to lipoprotein particle	2/100	33/18862
BP	GO:0060251	regulation of glial cell proliferation	2/100	33/18862
BP	GO:0061049	cell growth involved in cardiac muscle cell development	2/100	33/18862
BP	GO:0072538	T-helper 17 type immune response	2/100	33/18862
BP	GO:1904030	negative regulation of cyclin-dependent protein kinase	2/100	33/18862
BP	GO:1905332	positive regulation of morphogenesis of an epithelium	2/100	33/18862
BP	GO:0006022	aminoglycan metabolic process	4/100	172/18862
BP	GO:0002042	cell migration involved in sprouting angiogenesis	3/100	93/18862
BP	GO:0051153	regulation of striated muscle cell differentiation	3/100	93/18862
BP	GO:0060401	cytosolic calcium ion transport	4/100	173/18862
BP	GO:0042982	amyloid precursor protein metabolic process	3/100	94/18862
BP	GO:0032200	telomere organization	4/100	174/18862
BP	GO:0072503	cellular divalent inorganic cation homeostasis	7/100	480/18862
BP	GO:0008156	negative regulation of DNA replication	2/100	34/18862
BP	GO:0010614	negative regulation of cardiac muscle hypertrophy	2/100	34/18862
BP	GO:0060306	regulation of membrane repolarization	2/100	34/18862
BP	GO:0071402	cellular response to lipoprotein particle stimulus	2/100	34/18862
BP	GO:0090050	positive regulation of cell migration involved in sprouting angiogenesis	2/100	34/18862
BP	GO:0090277	positive regulation of peptide hormone secretion	3/100	95/18862
BP	GO:0097711	ciliary basal body-plasma membrane docking	3/100	95/18862

BP	GO:0045619	regulation of lymphocyte differentiation	4/100	175/18862
BP	GO:0010256	endomembrane system organization	7/100	482/18862
BP	GO:0006941	striated muscle contraction	4/100	176/18862
BP	GO:0015850	organic hydroxy compound transport	5/100	271/18862
BP	GO:0008608	attachment of spindle microtubules to kinetochore	2/100	35/18862
BP	GO:0016242	negative regulation of macroautophagy	2/100	35/18862
BP	GO:0042554	superoxide anion generation	2/100	35/18862
BP	GO:0060260	regulation of transcription initiation from RNA polymerase II promoter	2/100	35/18862
BP	GO:0086005	ventricular cardiac muscle cell action potential	2/100	35/18862
BP	GO:0098926	postsynaptic signal transduction	2/100	35/18862
BP	GO:1900016	negative regulation of cytokine production involved in inflammatory response	2/100	35/18862
BP	GO:1903427	negative regulation of reactive oxygen species biosynthetic process	2/100	35/18862
BP	GO:1903960	negative regulation of anion transmembrane transport	2/100	35/18862
BP	GO:0002027	regulation of heart rate	3/100	97/18862
BP	GO:0043502	regulation of muscle adaptation	3/100	97/18862
BP	GO:0006839	mitochondrial transport	5/100	274/18862
BP	GO:0002369	T cell cytokine production	2/100	36/18862
BP	GO:0003176	aortic valve development	2/100	36/18862
BP	GO:0014741	negative regulation of muscle hypertrophy	2/100	36/18862
BP	GO:0031572	G2 DNA damage checkpoint	2/100	36/18862
BP	GO:0043267	negative regulation of potassium ion transport	2/100	36/18862
BP	GO:0043368	positive T cell selection	2/100	36/18862
BP	GO:0006959	humoral immune response	6/100	380/18862
BP	GO:0002824	positive regulation of adaptive immune response based on somatic recombination of immune receptors built from immunoglobulin superfamily domains	3/100	99/18862
BP	GO:0019233	sensory perception of pain	3/100	99/18862
BP	GO:0044728	DNA methylation or demethylation	3/100	99/18862
BP	GO:0022406	membrane docking	4/100	181/18862
BP	GO:0043409	negative regulation of MAPK cascade	4/100	181/18862
BP	GO:0031098	stress-activated protein kinase signaling cascade	5/100	276/18862
BP	GO:0002695	negative regulation of leukocyte activation	4/100	182/18862
BP	GO:0010950	positive regulation of endopeptidase activity	4/100	182/18862
BP	GO:0061136	regulation of proteasomal protein catabolic process	4/100	182/18862

BP	GO:0006939	smooth muscle contraction	3/100	100/18862
BP	GO:0060079	excitatory postsynaptic potential	3/100	100/18862
BP	GO:1905269	positive regulation of chromatin organization	3/100	100/18862
BP	GO:0002385	mucosal immune response	2/100	37/18862
BP	GO:0010737	protein kinase A signaling	2/100	37/18862
BP	GO:0044818	mitotic G2/M transition checkpoint	2/100	37/18862
BP	GO:0070873	regulation of glycogen metabolic process	2/100	37/18862
BP	GO:0070897	transcription preinitiation complex assembly	2/100	37/18862
BP	GO:0060047	heart contraction	5/100	279/18862
BP	GO:0022037	metencephalon development	3/100	101/18862
BP	GO:1902106	negative regulation of leukocyte differentiation	3/100	101/18862
BP	GO:1902275	regulation of chromatin organization	4/100	185/18862
BP	GO:0044264	cellular polysaccharide metabolic process	3/100	102/18862
BP	GO:0003091	renal water homeostasis	2/100	38/18862
BP	GO:0010463	mesenchymal cell proliferation	2/100	38/18862
BP	GO:0048246	macrophage chemotaxis	2/100	38/18862
BP	GO:0051154	negative regulation of striated muscle cell differentiation	2/100	38/18862
BP	GO:0060612	adipose tissue development	2/100	38/18862
BP	GO:1904706	negative regulation of vascular associated smooth muscle cell proliferation	2/100	38/18862
BP	GO:0030593	neutrophil chemotaxis	3/100	103/18862
BP	GO:0045333	cellular respiration	4/100	187/18862
BP	GO:0002456	T cell mediated immunity	3/100	104/18862
BP	GO:0002821	positive regulation of adaptive immune response	3/100	104/18862
BP	GO:2000060	positive regulation of ubiquitin-dependent protein catabolic process	3/100	104/18862
BP	GO:0033146	regulation of intracellular estrogen receptor signaling pathway	2/100	39/18862
BP	GO:0045742	positive regulation of epidermal growth factor receptor signaling pathway	2/100	39/18862
BP	GO:0045746	negative regulation of Notch signaling pathway	2/100	39/18862
BP	GO:0060416	response to growth hormone	2/100	39/18862
BP	GO:0071470	cellular response to osmotic stress	2/100	39/18862
BP	GO:0086091	regulation of heart rate by cardiac conduction	2/100	39/18862
BP	GO:0002285	lymphocyte activation involved in immune response	4/100	189/18862
BP	GO:0099565	chemical synaptic transmission, postsynaptic	3/100	105/18862
BP	GO:1903707	negative regulation of hemopoiesis	3/100	105/18862
BP	GO:0050821	protein stabilization	4/100	191/18862
BP	GO:0009062	fatty acid catabolic process	3/100	106/18862

BP	GO:0072009	nephron epithelium development	3/100	106/18862
BP	GO:0003015	heart process	5/100	289/18862
BP	GO:0042908	xenobiotic transport	2/100	40/18862
BP	GO:0055023	positive regulation of cardiac muscle tissue growth	2/100	40/18862
BP	GO:1904037	positive regulation of epithelial cell apoptotic process	2/100	40/18862
BP	GO:0046632	alpha-beta T cell differentiation	3/100	107/18862
BP	GO:2001022	positive regulation of response to DNA damage stimulus	3/100	107/18862
BP	GO:0008593	regulation of Notch signaling pathway	3/100	108/18862
BP	GO:0010939	regulation of necrotic cell death	2/100	41/18862
BP	GO:0030850	prostate gland development	2/100	41/18862
BP	GO:0042088	T-helper 1 type immune response	2/100	41/18862
BP	GO:0051602	response to electrical stimulus	2/100	41/18862
BP	GO:1901186	positive regulation of ERBB signaling pathway	2/100	41/18862
BP	GO:1903580	positive regulation of ATP metabolic process	2/100	41/18862
BP	GO:1905314	semi-lunar valve development	2/100	41/18862
BP	GO:1901800	positive regulation of proteasomal protein catabolic process	3/100	109/18862
BP	GO:0006164	purine nucleotide biosynthetic process	4/100	197/18862
BP	GO:0003197	endocardial cushion development	2/100	42/18862
BP	GO:0031670	cellular response to nutrient	2/100	42/18862
BP	GO:0048806	genitalia development	2/100	42/18862
BP	GO:0072595	maintenance of protein localization in organelle	2/100	42/18862
BP	GO:1902622	regulation of neutrophil migration	2/100	42/18862
BP	GO:0006575	cellular modified amino acid metabolic process	4/100	198/18862
BP	GO:0031623	receptor internalization	3/100	111/18862
BP	GO:0021782	glial cell development	3/100	112/18862
BP	GO:0030278	regulation of ossification	3/100	112/18862
BP	GO:0032411	positive regulation of transporter activity	3/100	112/18862
BP	GO:0006734	NADH metabolic process	2/100	43/18862
BP	GO:0097178	ruffle assembly	2/100	43/18862
BP	GO:2000273	positive regulation of signaling receptor activity	2/100	43/18862
BP	GO:0001909	leukocyte mediated cytotoxicity	3/100	113/18862
BP	GO:0005976	polysaccharide metabolic process	3/100	113/18862
BP	GO:0055007	cardiac muscle cell	3/100	114/18862
BP	GO:0003254	regulation of membrane depolarization	2/100	44/18862
BP	GO:0010863	positive regulation of phospholipase C activity	2/100	44/18862
BP	GO:0010874	regulation of cholesterol efflux	2/100	44/18862
BP	GO:0035987	endodermal cell differentiation	2/100	44/18862
BP	GO:0060324	face development	2/100	44/18862

BP	GO:0060421	positive regulation of heart growth	2/100	44/18862
BP	GO:0098815	modulation of excitatory postsynaptic potential	2/100	44/18862
BP	GO:0030004	cellular monovalent inorganic cation homeostasis	3/100	115/18862
BP	GO:0031396	regulation of protein ubiquitination	4/100	205/18862
BP	GO:0014911	positive regulation of smooth muscle cell migration	2/100	45/18862
BP	GO:0031018	endocrine pancreas development	2/100	45/18862
BP	GO:0042551	neuron maturation	2/100	45/18862
BP	GO:0048512	circadian behavior	2/100	45/18862
BP	GO:0060986	endocrine hormone secretion	2/100	45/18862
BP	GO:0086009	membrane repolarization	2/100	45/18862
BP	GO:0003044	regulation of systemic arterial blood pressure mediated by a chemical signal	2/100	46/18862
BP	GO:0005978	glycogen biosynthetic process	2/100	46/18862
BP	GO:0007622	rhythmic behavior	2/100	46/18862
BP	GO:0009250	glucan biosynthetic process	2/100	46/18862
BP	GO:0034198	cellular response to amino acid starvation	2/100	46/18862
BP	GO:1900274	regulation of phospholipase C activity	2/100	46/18862
BP	GO:2001258	negative regulation of cation channel activity	2/100	46/18862
BP	GO:0002221	pattern recognition receptor signaling pathway	4/100	208/18862
BP	GO:0072522	purine-containing compound biosynthetic process	4/100	208/18862
BP	GO:0051960	regulation of nervous system development	6/100	422/18862
BP	GO:0072676	lymphocyte migration	3/100	118/18862
BP	GO:0002703	regulation of leukocyte mediated immunity	4/100	209/18862
BP	GO:0045216	cell-cell junction organization	4/100	209/18862
BP	GO:0045446	endothelial cell differentiation	3/100	119/18862
BP	GO:1903578	regulation of ATP metabolic process	3/100	119/18862
BP	GO:0046034	ATP metabolic process	5/100	313/18862
BP	GO:0008089	anterograde axonal transport	2/100	47/18862
BP	GO:0030225	macrophage differentiation	2/100	47/18862
BP	GO:0031279	regulation of cyclase activity	2/100	47/18862
BP	GO:0045581	negative regulation of T cell differentiation	2/100	47/18862
BP	GO:0090329	regulation of DNA-dependent DNA replication	2/100	47/18862
BP	GO:0010508	positive regulation of autophagy	3/100	120/18862
BP	GO:0070252	actin-mediated cell contraction	3/100	120/18862
BP	GO:0045088	regulation of innate immune response	5/100	315/18862
BP	GO:0006304	DNA modification	3/100	121/18862
BP	GO:0002762	negative regulation of myeloid leukocyte differentiation	2/100	48/18862
BP	GO:0014009	glial cell proliferation	2/100	48/18862

BP	GO:0035850	epithelial cell differentiation involved in kidney development	2/100	48/18862
BP	GO:0038084	vascular endothelial growth factor signaling pathway	2/100	48/18862
BP	GO:0042149	cellular response to glucose starvation	2/100	48/18862
BP	GO:0043330	response to exogenous dsRNA	2/100	48/18862
BP	GO:0043616	keratinocyte proliferation	2/100	48/18862
BP	GO:0045058	T cell selection	2/100	48/18862
BP	GO:0052372	modulation by symbiont of entry into host	2/100	48/18862
BP	GO:0060688	regulation of morphogenesis of a branching structure	2/100	48/18862
BP	GO:0061028	establishment of endothelial barrier	2/100	48/18862
BP	GO:1902003	regulation of amyloid-beta formation	2/100	48/18862
BP	GO:1990090	cellular response to nerve growth factor stimulus	2/100	48/18862
BP	GO:0035270	endocrine system development	3/100	122/18862
BP	GO:1903050	regulation of proteolysis involved in cellular protein catabolic process	4/100	215/18862
BP	GO:0007173	epidermal growth factor receptor signaling pathway	3/100	123/18862
BP	GO:1990266	neutrophil migration	3/100	123/18862
BP	GO:0032873	negative regulation of stress-activated MAPK cascade	2/100	49/18862
BP	GO:0043124	negative regulation of I-kappaB kinase/NF-kappaB signaling	2/100	49/18862
BP	GO:0045661	regulation of myoblast differentiation	2/100	49/18862
BP	GO:0070303	negative regulation of stress-activated protein kinase signaling cascade	2/100	49/18862
BP	GO:0072132	mesenchyme morphogenesis	2/100	49/18862
BP	GO:1990928	response to amino acid starvation	2/100	49/18862
BP	GO:0042552	myelination	3/100	124/18862
BP	GO:0046887	positive regulation of hormone secretion	3/100	124/18862
BP	GO:0002705	positive regulation of leukocyte mediated immunity	3/100	125/18862
BP	GO:0045744	negative regulation of G protein-coupled receptor signaling pathway	2/100	50/18862
BP	GO:0045912	negative regulation of carbohydrate metabolic process	2/100	50/18862
BP	GO:0048260	positive regulation of receptor-mediated endocytosis	2/100	50/18862
BP	GO:0051339	regulation of lyase activity	2/100	50/18862
BP	GO:1903202	negative regulation of oxidative stress-induced cell death	2/100	50/18862
BP	GO:1990089	response to nerve growth factor	2/100	50/18862
BP	GO:0000186	activation of MAPKK activity	2/100	51/18862
BP	GO:0019674	NAD metabolic process	2/100	51/18862

BP	GO:0017015	regulation of transforming growth factor beta receptor signaling pathway	3/100	127/18862
BP	GO:0030968	endoplasmic reticulum unfolded protein response	3/100	127/18862
BP	GO:0071333	cellular response to glucose stimulus	3/100	127/18862
BP	GO:1903052	positive regulation of proteolysis involved in cellular protein catabolic process	3/100	127/18862
BP	GO:0002576	platelet degranulation	3/100	128/18862
BP	GO:0032479	regulation of type I interferon production	3/100	128/18862
BP	GO:1990778	protein localization to cell periphery	5/100	329/18862
BP	GO:0001658	branching involved in ureteric bud morphogenesis	2/100	52/18862
BP	GO:0006998	nuclear envelope organization	2/100	52/18862
BP	GO:0043392	negative regulation of DNA binding	2/100	52/18862
BP	GO:0071827	plasma lipoprotein particle organization	2/100	52/18862
BP	GO:0032606	type I interferon production	3/100	129/18862
BP	GO:0046328	regulation of JNK cascade	3/100	129/18862
BP	GO:0071331	cellular response to hexose stimulus	3/100	129/18862
BP	GO:0043254	regulation of protein-containing complex assembly	6/100	446/18862
BP	GO:0071326	cellular response to monosaccharide stimulus	3/100	130/18862
BP	GO:0016570	histone modification	6/100	448/18862
BP	GO:0001706	endoderm formation	2/100	53/18862
BP	GO:0003179	heart valve morphogenesis	2/100	53/18862
BP	GO:0010524	positive regulation of calcium ion transport into cytosol	2/100	53/18862
BP	GO:1901185	negative regulation of ERBB signaling pathway	2/100	53/18862
BP	GO:1905517	macrophage migration	2/100	53/18862
BP	GO:0072073	kidney epithelium development	3/100	131/18862
BP	GO:0072329	monocarboxylic acid catabolic process	3/100	131/18862
BP	GO:0032434	regulation of proteasomal ubiquitin-dependent protein catabolic process	3/100	132/18862
BP	GO:0030199	collagen fibril organization	2/100	54/18862
BP	GO:0042304	regulation of fatty acid biosynthetic process	2/100	54/18862
BP	GO:0043331	response to dsRNA	2/100	54/18862
BP	GO:0045727	positive regulation of translation	3/100	133/18862
BP	GO:0050768	negative regulation of neurogenesis	3/100	133/18862
BP	GO:0031529	ruffle organization	2/100	55/18862
BP	GO:0045620	negative regulation of lymphocyte differentiation	2/100	55/18862
BP	GO:1902991	regulation of amyloid precursor protein catabolic process	2/100	55/18862
BP	GO:0001508	action potential	3/100	135/18862
BP	GO:0006754	ATP biosynthetic process	2/100	56/18862

BP	GO:0051353	positive regulation of oxidoreductase activity	2/100	56/18862
BP	GO:0071825	protein-lipid complex subunit organization	2/100	56/18862
BP	GO:0060078	regulation of postsynaptic membrane potential	3/100	136/18862
BP	GO:0016569	covalent chromatin modification	6/100	461/18862
BP	GO:0030183	B cell differentiation	3/100	137/18862
BP	GO:0031644	regulation of nervous system process	3/100	137/18862
BP	GO:0034205	amyloid-beta formation	2/100	57/18862
BP	GO:0051961	negative regulation of nervous system development	3/100	138/18862
BP	GO:0071322	cellular response to carbohydrate stimulus	3/100	138/18862
BP	GO:1903320	regulation of protein modification by small protein conjugation or removal	4/100	237/18862
BP	GO:0030902	hindbrain development	3/100	139/18862
BP	GO:0055076	transition metal ion homeostasis	3/100	139/18862
BP	GO:0050767	regulation of neurogenesis	5/100	348/18862
BP	GO:0060675	ureteric bud morphogenesis	2/100	58/18862
BP	GO:0061005	cell differentiation involved in kidney development	2/100	58/18862
BP	GO:0038093	Fc receptor signaling pathway	4/100	239/18862
BP	GO:0016331	morphogenesis of embryonic epithelium	3/100	140/18862
BP	GO:0030071	regulation of mitotic metaphase/anaphase transition	2/100	59/18862
BP	GO:0032663	regulation of interleukin-2 production	2/100	59/18862
BP	GO:0033260	nuclear DNA replication	2/100	59/18862
BP	GO:0043030	regulation of macrophage activation	2/100	59/18862
BP	GO:0051055	negative regulation of lipid biosynthetic process	2/100	59/18862
BP	GO:0072171	mesonephric tubule morphogenesis	2/100	59/18862
BP	GO:0098930	axonal transport	2/100	59/18862
BP	GO:0019730	antimicrobial humoral response	3/100	142/18862
BP	GO:0010518	positive regulation of phospholipase activity	2/100	60/18862
BP	GO:0032722	positive regulation of chemokine production	2/100	60/18862
BP	GO:0034113	heterotypic cell-cell adhesion	2/100	60/18862
BP	GO:0046888	negative regulation of hormone secretion	2/100	60/18862
BP	GO:0048857	neural nucleus development	2/100	60/18862
BP	GO:0051926	negative regulation of calcium ion transport	2/100	60/18862
BP	GO:0140115	export across plasma membrane	2/100	60/18862
BP	GO:1903078	positive regulation of protein localization to plasma membrane	2/100	60/18862
BP	GO:1902850	microtubule cytoskeleton organization involved in mitosis	3/100	143/18862
BP	GO:0008277	regulation of G protein-coupled receptor signaling pathway	3/100	144/18862

BP	GO:0022412	cellular process involved in reproduction in multicellular organism	5/100	357/18862
BP	GO:0008016	regulation of heart contraction	4/100	245/18862
BP	GO:0003170	heart valve development	2/100	61/18862
BP	GO:0007091	metaphase/anaphase transition of mitotic cell cycle	2/100	61/18862
BP	GO:0032623	interleukin-2 production	2/100	61/18862
BP	GO:0048645	animal organ formation	2/100	61/18862
BP	GO:0070613	regulation of protein processing	2/100	61/18862
BP	GO:0001678	cellular glucose homeostasis	3/100	145/18862
BP	GO:1903169	regulation of calcium ion transmembrane transport	3/100	145/18862
BP	GO:0001654	eye development	5/100	359/18862
BP	GO:0016525	negative regulation of angiogenesis	3/100	146/18862
BP	GO:0002437	inflammatory response to antigenic stimulus	2/100	62/18862
BP	GO:0006521	regulation of cellular amino acid metabolic process	2/100	62/18862
BP	GO:0019933	cAMP-mediated signaling	2/100	62/18862
BP	GO:0032835	glomerulus development	2/100	62/18862
BP	GO:0045453	bone resorption	2/100	62/18862
BP	GO:0051898	negative regulation of protein kinase B signaling	2/100	62/18862
BP	GO:0061912	selective autophagy	2/100	62/18862
BP	GO:1902099	regulation of metaphase/anaphase transition of cell cycle	2/100	62/18862
BP	GO:0090263	positive regulation of canonical Wnt signaling pathway	3/100	147/18862
BP	GO:0010498	proteasomal protein catabolic process	6/100	483/18862
BP	GO:0150063	visual system development	5/100	363/18862
BP	GO:0006081	cellular aldehyde metabolic process	2/100	63/18862
BP	GO:0045670	regulation of osteoclast differentiation	2/100	63/18862
BP	GO:0071677	positive regulation of mononuclear cell migration	2/100	63/18862
BP	GO:1903317	regulation of protein maturation	2/100	63/18862
BP	GO:2000401	regulation of lymphocyte migration	2/100	63/18862
BP	GO:2000181	negative regulation of blood vessel morphogenesis	3/100	148/18862
BP	GO:1901617	organic hydroxy compound biosynthetic process	4/100	251/18862
BP	GO:0034620	cellular response to unfolded protein	3/100	149/18862
BP	GO:0046631	alpha-beta T cell activation	3/100	149/18862
BP	GO:1901343	negative regulation of vasculature development	3/100	149/18862
BP	GO:0010965	regulation of mitotic sister chromatid separation	2/100	64/18862
BP	GO:0033344	cholesterol efflux	2/100	64/18862
BP	GO:0035914	skeletal muscle cell	2/100	64/18862
BP	GO:0042093	T-helper cell differentiation	2/100	64/18862

BP	GO:0044784	metaphase/anaphase transition of cell cycle	2/100	64/18862
BP	GO:0044786	cell cycle DNA replication	2/100	64/18862
BP	GO:0050922	negative regulation of chemotaxis	2/100	64/18862
BP	GO:0030048	actin filament-based movement	3/100	150/18862
BP	GO:0050777	negative regulation of immune response	3/100	150/18862
BP	GO:0050871	positive regulation of B cell activation	3/100	150/18862
BP	GO:0002460	adaptive immune response based on somatic recombination of immune receptors built from immunoglobulin superfamily domains	5/100	367/18862
CC	GO:0045121	membrane raft	16/100	323/19520
CC	GO:0098857	membrane microdomain	16/100	323/19520
CC	GO:0000307	cyclin-dependent protein kinase holoenzyme complex	7/100	43/19520
CC	GO:1902554	serine/threonine protein kinase complex	7/100	89/19520
CC	GO:1902911	protein kinase complex	7/100	104/19520
CC	GO:0044853	plasma membrane raft	6/100	111/19520
CC	GO:0061695	transferase complex, transferring phosphorus-containing groups	8/100	253/19520
CC	GO:0062023	collagen-containing extracellular matrix	10/100	423/19520
CC	GO:0031968	organelle outer membrane	7/100	220/19520
CC	GO:0019867	outer membrane	7/100	222/19520
CC	GO:0005819	spindle	9/100	381/19520
CC	GO:0031233	intrinsic component of external side of plasma membrane	3/100	23/19520
CC	GO:0090575	RNA polymerase II transcription regulator complex	6/100	170/19520
CC	GO:0005667	transcription regulator complex	9/100	409/19520
CC	GO:0031983	vesicle lumen	8/100	328/19520
CC	GO:0005925	focal adhesion	9/100	416/19520
CC	GO:0030055	cell-substrate junction	9/100	423/19520
CC	GO:1904813	ficolin-1-rich granule lumen	5/100	124/19520
CC	GO:0016324	apical plasma membrane	8/100	351/19520
CC	GO:0005741	mitochondrial outer membrane	6/100	195/19520
CC	GO:0030139	endocytic vesicle	7/100	307/19520
CC	GO:0009897	external side of plasma membrane	8/100	402/19520
CC	GO:0043209	myelin sheath	3/100	42/19520
CC	GO:0045177	apical part of cell	8/100	414/19520
CC	GO:0034774	secretory granule lumen	7/100	322/19520
CC	GO:0060205	cytoplasmic vesicle lumen	7/100	326/19520
CC	GO:0005635	nuclear envelope	8/100	462/19520
CC	GO:0101002	ficolin-1-rich granule	5/100	185/19520
CC	GO:1904724	tertiary granule lumen	3/100	55/19520
CC	GO:0005769	early endosome	7/100	378/19520
CC	GO:0005771	multivesicular body	3/100	62/19520
CC	GO:0031965	nuclear membrane	6/100	295/19520
CC	GO:0071682	endocytic vesicle lumen	2/100	20/19520

CC	GO:0031258	lamellipodium membrane	2/100	22/19520
CC	GO:0005876	spindle microtubule	3/100	71/19520
CC	GO:0005901	caveola	3/100	80/19520
CC	GO:0120111	neuron projection cytoplasm	3/100	86/19520
CC	GO:0070820	tertiary granule	4/100	164/19520
MF	GO:0004879	nuclear receptor activity	11/100	52/18337
MF	GO:0098531	ligand-activated transcription factor activity	11/100	52/18337
MF	GO:0140297	DNA-binding transcription factor binding	17/100	376/18337
MF	GO:0003707	steroid hormone receptor activity	7/100	26/18337
MF	GO:0044389	ubiquitin-like protein ligase binding	14/100	312/18337
MF	GO:0061629	RNA polymerase II-specific DNA-binding transcription factor binding	13/100	271/18337
MF	GO:0001223	transcription coactivator binding	6/100	26/18337
MF	GO:0017171	serine hydrolase activity	11/100	188/18337
MF	GO:0004252	serine-type endopeptidase activity	10/100	168/18337
MF	GO:0031625	ubiquitin protein ligase binding	12/100	293/18337
MF	GO:0008236	serine-type peptidase activity	10/100	186/18337
MF	GO:0020037	heme binding	9/100	140/18337
MF	GO:0005496	steroid binding	8/100	100/18337
MF	GO:0046906	tetrapyrrole binding	9/100	150/18337
MF	GO:0016705	oxidoreductase activity, acting on paired donors, with incorporation or reduction of molecular oxygen	9/100	160/18337
MF	GO:0016709	oxidoreductase activity, acting on paired donors, with incorporation or reduction of molecular oxygen, NAD(P)H as one donor, and incorporation of one atom of oxygen	6/100	48/18337
MF	GO:0004175	endopeptidase activity	13/100	438/18337
MF	GO:0001221	transcription coregulator	6/100	61/18337
MF	GO:0097718	disordered domain specific binding	5/100	34/18337
MF	GO:0019902	phosphatase binding	9/100	193/18337
MF	GO:0004497	monooxygenase activity	7/100	101/18337
MF	GO:0070491	repressing transcription factor binding	6/100	71/18337
MF	GO:0001046	core promoter sequence-specific DNA binding	5/100	40/18337
MF	GO:0001091	RNA polymerase II general transcription initiation factor binding	4/100	19/18337
MF	GO:0001216	DNA-binding transcription activator activity	12/100	447/18337

MF	GO:0016209	antioxidant activity	6/100	86/18337
MF	GO:0016538	cyclin-dependent protein serine/threonine kinase regulator activity	5/100	50/18337
MF	GO:0019887	protein kinase regulator activity	8/100	190/18337
MF	GO:0019838	growth factor binding	7/100	137/18337
MF	GO:0035257	nuclear hormone receptor binding	7/100	140/18337
MF	GO:0016922	nuclear receptor binding	6/100	97/18337
MF	GO:0019903	protein phosphatase binding	7/100	148/18337
MF	GO:0051400	BH domain binding	3/100	10/18337
MF	GO:0070513	death domain binding	3/100	11/18337
MF	GO:0019207	kinase regulator activity	8/100	220/18337
MF	GO:0001228	DNA-binding transcription activator activity, RNA polymerase II-specific	11/100	443/18337
MF	GO:0008353	RNA polymerase II CTD heptapeptide repeat kinase activity	3/100	12/18337
MF	GO:0016712	oxidoreductase activity, acting on paired donors, with incorporation or reduction of molecular oxygen, reduced flavin or flavoprotein as one donor, and incorporation of one atom of oxygen	4/100	35/18337
MF	GO:0051427	hormone receptor binding	7/100	173/18337
MF	GO:0008395	steroid hydroxylase activity	4/100	38/18337
MF	GO:0033613	activating transcription factor binding	5/100	78/18337
MF	GO:0030331	estrogen receptor binding	4/100	41/18337
MF	GO:0051879	Hsp90 protein binding	4/100	41/18337
MF	GO:0002020	protease binding	6/100	131/18337
MF	GO:0051117	ATPase binding	5/100	85/18337
MF	GO:0140296	general transcription initiation factor binding	4/100	45/18337
MF	GO:0005126	cytokine receptor binding	8/100	270/18337
MF	GO:0004601	peroxidase activity	4/100	53/18337
MF	GO:0016684	oxidoreductase activity, acting on peroxide as acceptor	4/100	57/18337
MF	GO:0001224	RNA polymerase II transcription coregulator binding	3/100	24/18337
MF	GO:0070330	aromatase activity	3/100	25/18337
MF	GO:0004714	transmembrane receptor protein tyrosine kinase activity	4/100	61/18337
MF	GO:0046982	protein heterodimerization activity	8/100	324/18337
MF	GO:0035035	histone acetyltransferase	3/100	27/18337
MF	GO:0001098	basal transcription machinery binding	4/100	69/18337
MF	GO:0001099	basal RNA polymerase II transcription machinery binding	4/100	69/18337
MF	GO:0030332	cyclin binding	3/100	30/18337
MF	GO:0051721	protein phosphatase 2A binding	3/100	31/18337
MF	GO:0030291	protein serine/threonine kinase inhibitor activity	3/100	32/18337
MF	GO:0001618	virus receptor activity	4/100	76/18337

MF	GO:0035258	steroid hormone receptor binding	4/100	77/18337
MF	GO:0140272	exogenous protein binding	4/100	77/18337
MF	GO:0001103	RNA polymerase II repressing transcription factor binding	3/100	35/18337
MF	GO:0019199	transmembrane receptor protein kinase activity	4/100	80/18337
MF	GO:0005178	integrin binding	5/100	142/18337
MF	GO:0030295	protein kinase activator activity	4/100	84/18337
MF	GO:0019825	oxygen binding	3/100	39/18337
MF	GO:0001094	TFIID-class transcription factor complex binding	2/100	10/18337
MF	GO:0033218	amide binding	8/100	391/18337
MF	GO:0019209	kinase activator activity	4/100	90/18337
MF	GO:0042277	peptide binding	7/100	315/18337
MF	GO:0004712	protein serine/threonine/tyrosine kinase	3/100	44/18337
MF	GO:0004861	cyclin-dependent protein serine/threonine kinase inhibitor activity	2/100	12/18337
MF	GO:0042166	acetylcholine binding	2/100	12/18337
MF	GO:0043295	glutathione binding	2/100	12/18337
MF	GO:0004896	cytokine receptor activity	4/100	97/18337
MF	GO:0070888	E-box binding	3/100	47/18337
MF	GO:0036041	long-chain fatty acid binding	2/100	13/18337
MF	GO:1900750	oligopeptide binding	2/100	13/18337
MF	GO:0001102	RNA polymerase II activating transcription factor binding	3/100	48/18337
MF	GO:0097153	cysteine-type endopeptidase activity involved in apoptotic process	2/100	15/18337
MF	GO:0050661	NADP binding	3/100	53/18337
MF	GO:0010181	FMN binding	2/100	16/18337
MF	GO:0042165	neurotransmitter binding	2/100	16/18337
MF	GO:0031406	carboxylic acid binding	5/100	184/18337
MF	GO:0043177	organic acid binding	4/100	114/18337
MF	GO:0043621	protein self-association	3/100	56/18337
MF	GO:0005149	interleukin-1 receptor binding	2/100	17/18337
MF	GO:0046965	retinoid X receptor binding	2/100	17/18337
MF	GO:0000979	RNA polymerase II core promoter sequence-specific DNA binding	2/100	18/18337
MF	GO:0005507	copper ion binding	3/100	60/18337
MF	GO:0008144	drug binding	3/100	61/18337
MF	GO:0031072	heat shock protein binding	4/100	123/18337
MF	GO:0048018	receptor ligand activity	8/100	486/18337
MF	GO:0004857	enzyme inhibitor activity	7/100	385/18337
MF	GO:0008391	arachidonic acid monooxygenase activity	2/100	20/18337
MF	GO:0070182	DNA polymerase binding	2/100	20/18337
MF	GO:0030546	signaling receptor activator activity	8/100	492/18337
MF	GO:0005518	collagen binding	3/100	68/18337
MF	GO:0005540	hyaluronic acid binding	2/100	22/18337
MF	GO:0043027	cysteine-type endopeptidase inhibitor activity involved in apoptotic process	2/100	22/18337

MF	GO:0070412	R-SMAD binding	2/100	22/18337
MF	GO:0004860	protein kinase inhibitor activity	3/100	69/18337
MF	GO:0004713	protein tyrosine kinase activity	4/100	135/18337
MF	GO:0140375	immune receptor activity	4/100	136/18337
MF	GO:0001227	DNA-binding transcription repressor activity, RNA polymerase II-specific	6/100	307/18337
MF	GO:0033293	monocarboxylic acid binding	3/100	71/18337
MF	GO:0001217	DNA-binding transcription repressor activity	6/100	309/18337
MF	GO:0019210	kinase inhibitor activity	3/100	73/18337
MF	GO:0070851	growth factor receptor binding	4/100	141/18337
MF	GO:0004190	aspartic-type endopeptidase activity	2/100	25/18337
MF	GO:0016247	channel regulator activity	4/100	145/18337
MF	GO:0061134	peptidase regulator activity	5/100	230/18337
MF	GO:0004364	glutathione transferase activity	2/100	26/18337
MF	GO:0017025	TBP-class protein binding	2/100	26/18337
MF	GO:0042974	retinoic acid receptor binding	2/100	26/18337
MF	GO:0070001	aspartic-type peptidase activity	2/100	26/18337
MF	GO:0004674	protein serine/threonine kinase activity	7/100	430/18337
MF	GO:0005506	iron ion binding	4/100	150/18337
MF	GO:0005125	cytokine activity	5/100	235/18337
MF	GO:0004693	cyclin-dependent protein serine/threonine kinase activity	2/100	29/18337
MF	GO:0097472	cyclin-dependent protein kinase activity	2/100	29/18337
MF	GO:0001540	amyloid-beta binding	3/100	84/18337
MF	GO:0042562	hormone binding	3/100	84/18337
MF	GO:0008013	beta-catenin binding	3/100	85/18337
MF	GO:0051219	phosphoprotein binding	3/100	87/18337
MF	GO:0001530	lipopolysaccharide binding	2/100	32/18337
MF	GO:1904680	peptide transmembrane transporter activity	2/100	35/18337
MF	GO:0044183	protein folding chaperone	2/100	36/18337
MF	GO:1990782	protein tyrosine kinase binding	3/100	98/18337
MF	GO:0004866	endopeptidase inhibitor activity	4/100	180/18337
MF	GO:0005504	fatty acid binding	2/100	37/18337
MF	GO:0051087	chaperone binding	3/100	101/18337
MF	GO:0016248	channel inhibitor activity	2/100	39/18337
MF	GO:0043539	protein serine/threonine kinase activator activity	2/100	39/18337
MF	GO:0030414	peptidase inhibitor activity	4/100	187/18337
MF	GO:0043028	cysteine-type endopeptidase regulator activity involved in apoptotic process	2/100	40/18337
MF	GO:0047485	protein N-terminus binding	3/100	106/18337
MF	GO:0004222	metalloendopeptidase activity	3/100	107/18337
MF	GO:0061135	endopeptidase regulator activity	4/100	192/18337
MF	GO:0005516	calmodulin binding	4/100	198/18337
MF	GO:0042826	histone deacetylase binding	3/100	113/18337

pvalue	p.adjust	qvalue
7.21E-24	2.47E-20	1.30E-20
2.59E-19	4.44E-16	2.35E-16
4.08E-19	4.65E-16	2.46E-16
1.45E-18	1.24E-15	6.57E-16
9.29E-18	5.70E-15	3.01E-15
9.99E-18	5.70E-15	3.01E-15
3.05E-17	1.49E-14	7.89E-15
1.02E-16	4.36E-14	2.31E-14
1.15E-16	4.36E-14	2.31E-14
1.34E-16	4.59E-14	2.43E-14
2.27E-16	7.08E-14	3.74E-14
4.50E-16	1.20E-13	6.33E-14
4.54E-16	1.20E-13	6.33E-14
6.14E-16	1.50E-13	7.93E-14
9.37E-16	2.01E-13	1.06E-13
9.40E-16	2.01E-13	1.06E-13
1.22E-15	2.46E-13	1.30E-13
1.49E-15	2.83E-13	1.50E-13
1.69E-15	3.04E-13	1.61E-13

3.25E-15	5.56E-13	2.94E-13
4.51E-15	7.34E-13	3.88E-13
5.76E-15	8.96E-13	4.74E-13
7.18E-15	1.07E-12	5.65E-13
1.70E-14	2.43E-12	1.28E-12
2.36E-14	3.24E-12	1.71E-12
2.87E-14	3.78E-12	2.00E-12
5.09E-14	6.22E-12	3.29E-12
5.09E-14	6.22E-12	3.29E-12
5.75E-14	6.79E-12	3.59E-12
8.34E-14	9.51E-12	5.03E-12
9.41E-14	1.04E-11	5.49E-12
9.89E-14	1.06E-11	5.59E-12
1.21E-13	1.26E-11	6.65E-12
3.09E-13	3.11E-11	1.64E-11
5.39E-13	5.27E-11	2.78E-11
1.04E-12	9.90E-11	5.24E-11
1.88E-12	1.74E-10	9.18E-11
2.61E-12	2.35E-10	1.24E-10
2.69E-12	2.36E-10	1.25E-10
3.74E-12	3.20E-10	1.69E-10
4.72E-12	3.94E-10	2.08E-10
5.22E-12	4.25E-10	2.25E-10
5.67E-12	4.51E-10	2.39E-10
7.06E-12	5.49E-10	2.90E-10
9.05E-12	6.88E-10	3.64E-10

9.82E-12	7.30E-10	3.86E-10
1.03E-11	7.53E-10	3.98E-10
1.17E-11	8.34E-10	4.41E-10
1.19E-11	8.34E-10	4.41E-10
1.40E-11	9.59E-10	5.07E-10
1.47E-11	9.88E-10	5.22E-10
1.51E-11	9.94E-10	5.26E-10
1.72E-11	1.11E-09	5.86E-10
2.01E-11	1.28E-09	6.75E-10
2.46E-11	1.53E-09	8.08E-10
2.52E-11	1.54E-09	8.15E-10
3.16E-11	1.89E-09	1.00E-09
3.22E-11	1.89E-09	1.00E-09
3.26E-11	1.89E-09	1.00E-09
3.69E-11	2.10E-09	1.11E-09
4.28E-11	2.36E-09	1.25E-09
4.28E-11	2.36E-09	1.25E-09
5.00E-11	2.70E-09	1.43E-09
5.05E-11	2.70E-09	1.43E-09
5.54E-11	2.91E-09	1.54E-09
5.85E-11	3.03E-09	1.60E-09
6.11E-11	3.12E-09	1.65E-09
6.43E-11	3.24E-09	1.71E-09
7.60E-11	3.72E-09	1.96E-09
7.60E-11	3.72E-09	1.96E-09
8.93E-11	4.30E-09	2.28E-09
9.78E-11	4.65E-09	2.46E-09
1.44E-10	6.77E-09	3.58E-09

1.90E-10	8.77E-09	4.64E-09
1.94E-10	8.84E-09	4.67E-09
2.12E-10	9.32E-09	4.93E-09
2.12E-10	9.32E-09	4.93E-09
2.12E-10	9.32E-09	4.93E-09
2.54E-10	1.10E-08	5.83E-09
2.64E-10	1.13E-08	5.97E-09
2.76E-10	1.17E-08	6.16E-09
3.07E-10	1.28E-08	6.77E-09
3.95E-10	1.63E-08	8.61E-09
4.40E-10	1.79E-08	9.47E-09
4.67E-10	1.88E-08	9.93E-09
4.88E-10	1.94E-08	1.03E-08
5.21E-10	2.05E-08	1.08E-08
5.30E-10	2.06E-08	1.09E-08
5.41E-10	2.08E-08	1.10E-08
5.67E-10	2.15E-08	1.14E-08
6.26E-10	2.33E-08	1.23E-08
6.26E-10	2.33E-08	1.23E-08
6.92E-10	2.55E-08	1.35E-08
7.29E-10	2.66E-08	1.40E-08
7.54E-10	2.72E-08	1.44E-08
7.94E-10	2.83E-08	1.50E-08
8.33E-10	2.88E-08	1.52E-08
8.33E-10	2.88E-08	1.52E-08
8.33E-10	2.88E-08	1.52E-08
8.56E-10	2.93E-08	1.55E-08

8.89E-10	3.01E-08	1.59E-08
1.10E-09	3.68E-08	1.95E-08
1.12E-09	3.71E-08	1.96E-08
1.19E-09	3.91E-08	2.07E-08
1.27E-09	4.13E-08	2.18E-08
1.31E-09	4.24E-08	2.24E-08
1.39E-09	4.43E-08	2.34E-08
1.41E-09	4.46E-08	2.36E-08
1.43E-09	4.49E-08	2.37E-08
1.70E-09	5.30E-08	2.80E-08
2.09E-09	6.45E-08	3.41E-08
2.37E-09	7.23E-08	3.82E-08
2.62E-09	7.94E-08	4.20E-08
3.00E-09	9.00E-08	4.76E-08
3.31E-09	9.85E-08	5.21E-08
3.38E-09	9.96E-08	5.27E-08
3.50E-09	1.02E-07	5.41E-08
3.65E-09	1.06E-07	5.60E-08
3.79E-09	1.09E-07	5.77E-08
3.86E-09	1.10E-07	5.82E-08
4.02E-09	1.14E-07	6.02E-08
4.75E-09	1.32E-07	6.99E-08
4.75E-09	1.32E-07	6.99E-08
4.93E-09	1.35E-07	7.13E-08
4.93E-09	1.35E-07	7.13E-08
4.98E-09	1.35E-07	7.16E-08
5.86E-09	1.57E-07	8.29E-08
5.86E-09	1.57E-07	8.29E-08
6.58E-09	1.75E-07	9.23E-08
7.39E-09	1.94E-07	1.03E-07
7.98E-09	2.09E-07	1.10E-07
8.99E-09	2.33E-07	1.23E-07

9.10E-09	2.34E-07	1.24E-07
9.48E-09	2.42E-07	1.28E-07
9.82E-09	2.49E-07	1.32E-07
9.94E-09	2.50E-07	1.32E-07
1.05E-08	2.60E-07	1.37E-07
1.05E-08	2.60E-07	1.37E-07
1.09E-08	2.70E-07	1.43E-07
1.22E-08	2.99E-07	1.58E-07
1.23E-08	2.99E-07	1.58E-07
1.26E-08	3.02E-07	1.60E-07
1.26E-08	3.02E-07	1.60E-07
1.27E-08	3.03E-07	1.60E-07
1.38E-08	3.26E-07	1.72E-07
1.48E-08	3.46E-07	1.83E-07
1.55E-08	3.58E-07	1.89E-07
1.55E-08	3.58E-07	1.89E-07
1.58E-08	3.63E-07	1.92E-07
1.78E-08	4.07E-07	2.15E-07
1.82E-08	4.11E-07	2.18E-07
2.00E-08	4.50E-07	2.38E-07
2.28E-08	5.09E-07	2.69E-07
2.46E-08	5.45E-07	2.88E-07
2.47E-08	5.45E-07	2.88E-07
2.54E-08	5.57E-07	2.95E-07
2.58E-08	5.63E-07	2.98E-07
2.66E-08	5.77E-07	3.05E-07
2.78E-08	5.98E-07	3.16E-07
2.91E-08	6.22E-07	3.29E-07
3.03E-08	6.42E-07	3.40E-07
3.08E-08	6.42E-07	3.40E-07
3.08E-08	6.42E-07	3.40E-07

3.08E-08	6.42E-07	3.40E-07
3.14E-08	6.51E-07	3.44E-07
3.18E-08	6.52E-07	3.45E-07
3.18E-08	6.52E-07	3.45E-07
3.35E-08	6.82E-07	3.61E-07
3.43E-08	6.94E-07	3.67E-07
3.60E-08	7.24E-07	3.83E-07
3.69E-08	7.38E-07	3.90E-07
3.72E-08	7.38E-07	3.90E-07
3.73E-08	7.38E-07	3.90E-07
3.91E-08	7.68E-07	4.06E-07
4.13E-08	8.08E-07	4.27E-07
4.24E-08	8.25E-07	4.36E-07
4.75E-08	9.19E-07	4.86E-07
5.15E-08	9.85E-07	5.21E-07
5.19E-08	9.85E-07	5.21E-07
5.21E-08	9.85E-07	5.21E-07
5.21E-08	9.85E-07	5.21E-07
5.32E-08	9.96E-07	5.26E-07
5.32E-08	9.96E-07	5.26E-07
5.54E-08	1.03E-06	5.45E-07
5.85E-08	1.08E-06	5.72E-07
6.14E-08	1.13E-06	5.98E-07
6.41E-08	1.17E-06	6.20E-07
6.84E-08	1.24E-06	6.58E-07
7.26E-08	1.32E-06	6.96E-07
7.39E-08	1.33E-06	7.04E-07
7.76E-08	1.39E-06	7.35E-07
8.58E-08	1.53E-06	8.08E-07
9.15E-08	1.62E-06	8.58E-07

9.28E-08	1.64E-06	8.66E-07
9.40E-08	1.64E-06	8.69E-07
9.41E-08	1.64E-06	8.69E-07
9.59E-08	1.67E-06	8.81E-07
9.73E-08	1.67E-06	8.85E-07
9.73E-08	1.67E-06	8.85E-07
9.94E-08	1.69E-06	8.95E-07
9.94E-08	1.69E-06	8.95E-07
1.05E-07	1.77E-06	9.38E-07
1.14E-07	1.93E-06	1.02E-06
1.16E-07	1.95E-06	1.03E-06
1.25E-07	2.08E-06	1.10E-06
1.37E-07	2.27E-06	1.20E-06
1.41E-07	2.33E-06	1.23E-06
1.44E-07	2.37E-06	1.25E-06
1.49E-07	2.43E-06	1.28E-06
1.49E-07	2.43E-06	1.28E-06
1.51E-07	2.43E-06	1.29E-06
1.51E-07	2.43E-06	1.29E-06
1.52E-07	2.45E-06	1.29E-06
1.62E-07	2.59E-06	1.37E-06
1.64E-07	2.60E-06	1.38E-06
1.67E-07	2.65E-06	1.40E-06
1.71E-07	2.70E-06	1.43E-06
1.73E-07	2.71E-06	1.43E-06
1.74E-07	2.72E-06	1.44E-06
1.87E-07	2.90E-06	1.54E-06
1.88E-07	2.92E-06	1.54E-06
2.20E-07	3.40E-06	1.80E-06
2.27E-07	3.47E-06	1.83E-06
2.27E-07	3.47E-06	1.83E-06

2.32E-07	3.51E-06	1.86E-06
2.32E-07	3.51E-06	1.86E-06
2.39E-07	3.60E-06	1.90E-06
2.42E-07	3.63E-06	1.92E-06
2.44E-07	3.65E-06	1.93E-06
2.51E-07	3.74E-06	1.98E-06
2.71E-07	4.01E-06	2.12E-06
2.75E-07	4.02E-06	2.13E-06
2.75E-07	4.02E-06	2.13E-06
2.75E-07	4.02E-06	2.13E-06
2.83E-07	4.11E-06	2.17E-06
2.83E-07	4.11E-06	2.17E-06
2.98E-07	4.31E-06	2.28E-06
2.99E-07	4.31E-06	2.28E-06
3.33E-07	4.76E-06	2.52E-06
3.38E-07	4.82E-06	2.55E-06
3.53E-07	5.01E-06	2.65E-06
3.58E-07	5.06E-06	2.68E-06
3.75E-07	5.26E-06	2.78E-06
3.75E-07	5.26E-06	2.78E-06
3.98E-07	5.56E-06	2.94E-06
4.06E-07	5.65E-06	2.99E-06
4.14E-07	5.74E-06	3.03E-06
4.21E-07	5.81E-06	3.07E-06
4.25E-07	5.82E-06	3.08E-06
4.25E-07	5.82E-06	3.08E-06
4.48E-07	6.11E-06	3.23E-06
4.53E-07	6.15E-06	3.25E-06
5.00E-07	6.76E-06	3.58E-06
5.03E-07	6.76E-06	3.58E-06
5.04E-07	6.76E-06	3.58E-06
5.24E-07	7.01E-06	3.70E-06
5.29E-07	7.05E-06	3.73E-06
5.65E-07	7.47E-06	3.95E-06
5.65E-07	7.47E-06	3.95E-06

5.77E-07	7.59E-06	4.01E-06
5.98E-07	7.84E-06	4.15E-06
6.18E-07	8.07E-06	4.27E-06
6.33E-07	8.24E-06	4.36E-06
6.68E-07	8.66E-06	4.58E-06
6.85E-07	8.85E-06	4.68E-06
7.07E-07	9.07E-06	4.80E-06
7.08E-07	9.07E-06	4.80E-06
7.15E-07	9.09E-06	4.81E-06
7.15E-07	9.09E-06	4.81E-06
7.30E-07	9.26E-06	4.90E-06
7.58E-07	9.57E-06	5.06E-06
7.65E-07	9.62E-06	5.09E-06
7.98E-07	9.96E-06	5.27E-06
7.98E-07	9.96E-06	5.27E-06
8.37E-07	1.04E-05	5.51E-06
8.88E-07	1.10E-05	5.82E-06
9.33E-07	1.15E-05	6.09E-06
9.51E-07	1.17E-05	6.19E-06
9.65E-07	1.18E-05	6.26E-06
1.02E-06	1.24E-05	6.56E-06
1.04E-06	1.27E-05	6.72E-06
1.09E-06	1.31E-05	6.94E-06
1.09E-06	1.31E-05	6.94E-06
1.09E-06	1.31E-05	6.94E-06
1.12E-06	1.34E-05	7.09E-06
1.14E-06	1.37E-05	7.22E-06
1.16E-06	1.39E-05	7.34E-06
1.17E-06	1.39E-05	7.36E-06
1.20E-06	1.42E-05	7.53E-06
1.24E-06	1.46E-05	7.71E-06
1.27E-06	1.50E-05	7.91E-06

1.29E-06	1.51E-05	7.96E-06
1.30E-06	1.52E-05	8.04E-06
1.33E-06	1.54E-05	8.16E-06
1.33E-06	1.54E-05	8.16E-06
1.41E-06	1.62E-05	8.59E-06
1.41E-06	1.62E-05	8.59E-06
1.42E-06	1.63E-05	8.61E-06
1.54E-06	1.76E-05	9.31E-06
1.60E-06	1.82E-05	9.63E-06
1.62E-06	1.84E-05	9.75E-06
1.65E-06	1.86E-05	9.85E-06
1.65E-06	1.86E-05	9.85E-06
1.65E-06	1.86E-05	9.85E-06
1.68E-06	1.88E-05	9.93E-06
1.68E-06	1.88E-05	9.93E-06
1.75E-06	1.96E-05	1.03E-05
1.79E-06	1.99E-05	1.05E-05
1.82E-06	2.01E-05	1.06E-05
1.82E-06	2.01E-05	1.06E-05
1.87E-06	2.05E-05	1.09E-05
1.89E-06	2.07E-05	1.10E-05
1.90E-06	2.07E-05	1.10E-05
1.90E-06	2.07E-05	1.10E-05
1.94E-06	2.11E-05	1.11E-05
2.06E-06	2.23E-05	1.18E-05
2.15E-06	2.31E-05	1.22E-05
2.16E-06	2.31E-05	1.22E-05
2.16E-06	2.31E-05	1.22E-05
2.16E-06	2.31E-05	1.22E-05
2.17E-06	2.31E-05	1.22E-05
2.44E-06	2.59E-05	1.37E-05
2.56E-06	2.71E-05	1.43E-05
2.61E-06	2.76E-05	1.46E-05

2.63E-06	2.77E-05	1.47E-05
2.64E-06	2.77E-05	1.47E-05
2.71E-06	2.83E-05	1.50E-05
2.93E-06	3.06E-05	1.62E-05
2.98E-06	3.10E-05	1.64E-05
3.10E-06	3.21E-05	1.70E-05
3.11E-06	3.21E-05	1.70E-05
3.49E-06	3.60E-05	1.90E-05
4.14E-06	4.25E-05	2.25E-05
4.21E-06	4.31E-05	2.28E-05
4.36E-06	4.45E-05	2.35E-05
4.47E-06	4.55E-05	2.41E-05
4.49E-06	4.56E-05	2.41E-05
4.55E-06	4.61E-05	2.44E-05
4.87E-06	4.91E-05	2.60E-05
5.06E-06	5.07E-05	2.68E-05
5.06E-06	5.07E-05	2.68E-05
5.07E-06	5.08E-05	2.68E-05
5.18E-06	5.15E-05	2.72E-05
5.18E-06	5.15E-05	2.72E-05
5.20E-06	5.16E-05	2.73E-05
5.41E-06	5.32E-05	2.81E-05
5.41E-06	5.32E-05	2.81E-05
5.41E-06	5.32E-05	2.81E-05
5.89E-06	5.77E-05	3.05E-05
5.91E-06	5.77E-05	3.05E-05
6.00E-06	5.85E-05	3.09E-05
6.10E-06	5.88E-05	3.11E-05
6.10E-06	5.88E-05	3.11E-05
6.10E-06	5.88E-05	3.11E-05
6.10E-06	5.88E-05	3.11E-05
6.39E-06	6.15E-05	3.25E-05
6.61E-06	6.33E-05	3.35E-05
6.64E-06	6.33E-05	3.35E-05

6.64E-06	6.33E-05	3.35E-05
6.86E-06	6.53E-05	3.45E-05
7.05E-06	6.68E-05	3.53E-05
7.21E-06	6.80E-05	3.59E-05
7.21E-06	6.80E-05	3.59E-05
7.28E-06	6.83E-05	3.61E-05
7.28E-06	6.83E-05	3.61E-05
7.57E-06	7.04E-05	3.72E-05
7.57E-06	7.04E-05	3.72E-05
7.57E-06	7.04E-05	3.72E-05
7.71E-06	7.15E-05	3.78E-05
7.77E-06	7.17E-05	3.79E-05
7.77E-06	7.17E-05	3.79E-05
7.95E-06	7.31E-05	3.87E-05
8.02E-06	7.35E-05	3.89E-05
8.08E-06	7.39E-05	3.91E-05
8.29E-06	7.57E-05	4.00E-05
8.33E-06	7.58E-05	4.01E-05
8.59E-06	7.76E-05	4.10E-05
8.64E-06	7.76E-05	4.10E-05
8.64E-06	7.76E-05	4.10E-05
8.64E-06	7.76E-05	4.10E-05
8.64E-06	7.76E-05	4.10E-05
8.84E-06	7.92E-05	4.19E-05
8.99E-06	8.01E-05	4.24E-05
8.99E-06	8.01E-05	4.24E-05
9.33E-06	8.30E-05	4.39E-05
9.42E-06	8.35E-05	4.41E-05
9.56E-06	8.45E-05	4.47E-05
9.69E-06	8.50E-05	4.50E-05
9.69E-06	8.50E-05	4.50E-05
9.69E-06	8.50E-05	4.50E-05
9.75E-06	8.53E-05	4.51E-05
1.00E-05	8.75E-05	4.63E-05
1.02E-05	8.85E-05	4.68E-05
1.06E-05	9.22E-05	4.87E-05

1.07E-05	9.25E-05	4.89E-05
1.16E-05	9.99E-05	5.28E-05
1.19E-05	0.000102443	5.42E-05
1.20E-05	0.000103479	5.47E-05
1.21E-05	0.000103479	5.47E-05
1.21E-05	0.000103479	5.47E-05
1.28E-05	0.000109026	5.77E-05
1.31E-05	0.00011149	5.90E-05
1.34E-05	0.000114045	6.03E-05
1.38E-05	0.000114977	6.08E-05
1.38E-05	0.000114977	6.08E-05
1.38E-05	0.000114977	6.08E-05
1.38E-05	0.000114977	6.08E-05
1.38E-05	0.000114977	6.08E-05
1.38E-05	0.000114977	6.08E-05
1.38E-05	0.000114977	6.08E-05
1.38E-05	0.000114977	6.08E-05
1.39E-05	0.000115113	6.09E-05
1.39E-05	0.000115113	6.09E-05
1.51E-05	0.000124674	6.59E-05
1.57E-05	0.000129859	6.87E-05
1.60E-05	0.000130906	6.92E-05
1.60E-05	0.000130906	6.92E-05
1.60E-05	0.000130906	6.92E-05
1.61E-05	0.0001312	6.94E-05
1.64E-05	0.000133421	7.06E-05
1.64E-05	0.000133421	7.06E-05
1.69E-05	0.00013663	7.22E-05
1.69E-05	0.00013663	7.22E-05
1.70E-05	0.000137544	7.27E-05

1.72E-05	0.000138103	7.30E-05
1.78E-05	0.000142065	7.51E-05
1.78E-05	0.000142065	7.51E-05
1.78E-05	0.000142065	7.51E-05
1.78E-05	0.000142065	7.51E-05
1.83E-05	0.000145562	7.70E-05
1.83E-05	0.000145562	7.70E-05
1.93E-05	0.000152772	8.08E-05
1.96E-05	0.000154794	8.19E-05
2.03E-05	0.000159823	8.45E-05
2.09E-05	0.000164109	8.68E-05
2.10E-05	0.000164539	8.70E-05
2.16E-05	0.000169411	8.96E-05
2.24E-05	0.000174933	9.25E-05
2.29E-05	0.000178291	9.43E-05
2.31E-05	0.000178291	9.43E-05
2.31E-05	0.000178291	9.43E-05
2.31E-05	0.000178291	9.43E-05
2.31E-05	0.000178291	9.43E-05
2.31E-05	0.000178291	9.43E-05
2.39E-05	0.000183082	9.68E-05
2.39E-05	0.000183082	9.68E-05
2.46E-05	0.000188577	9.97E-05
2.57E-05	0.000196661	0.00010399
2.70E-05	0.0002047	0.000108241
2.70E-05	0.0002047	0.000108241
2.71E-05	0.0002047	0.000108241
2.72E-05	0.0002047	0.000108241
2.72E-05	0.0002047	0.000108241
2.72E-05	0.0002047	0.000108241
2.76E-05	0.000207414	0.000109676
2.79E-05	0.000208585	0.000110295
2.79E-05	0.000208585	0.000110295
2.79E-05	0.000208585	0.000110295
2.89E-05	0.000215478	0.00011394
2.90E-05	0.000215889	0.000114157
2.93E-05	0.000217581	0.000115052

3.05E-05	0.000225602	0.000119293
3.05E-05	0.000225602	0.000119293
3.07E-05	0.000226112	0.000119563
3.07E-05	0.000226112	0.000119563
3.13E-05	0.000230114	0.000121679
3.21E-05	0.000235001	0.000124263
3.49E-05	0.000255068	0.000134874
3.69E-05	0.000269201	0.000142347
3.71E-05	0.000269996	0.000142767
3.74E-05	0.000271505	0.000143566
3.85E-05	0.000278221	0.000147117
3.85E-05	0.000278221	0.000147117
3.92E-05	0.000282793	0.000149534
3.98E-05	0.00028485	0.000150622
3.98E-05	0.00028485	0.000150622
3.98E-05	0.00028485	0.000150622
3.98E-05	0.00028485	0.000150622
4.00E-05	0.000285696	0.000151069
4.07E-05	0.00028935	0.000153001
4.07E-05	0.00028935	0.000153001
4.08E-05	0.000289416	0.000153036
4.08E-05	0.000289416	0.000153036
4.20E-05	0.000297261	0.000157185
4.37E-05	0.000308487	0.000163121
4.53E-05	0.000319078	0.000168721
4.57E-05	0.000320828	0.000169646
4.70E-05	0.000329282	0.000174117
4.78E-05	0.000333825	0.000176519
4.78E-05	0.000333825	0.000176519
4.87E-05	0.00033883	0.000179166
4.87E-05	0.00033883	0.000179166
4.99E-05	0.00034654	0.000183242
5.15E-05	0.000356468	0.000188492
5.30E-05	0.000365986	0.000193525
5.30E-05	0.000365986	0.000193525
5.53E-05	0.000380012	0.000200942
5.53E-05	0.000380012	0.000200942
5.54E-05	0.000380162	0.000201021

5.63E-05	0.000385152	0.000203659
5.70E-05	0.000389419	0.000205916
5.87E-05	0.00039776	0.000210326
5.87E-05	0.00039776	0.000210326
5.87E-05	0.00039776	0.000210326
5.88E-05	0.00039776	0.000210326
5.88E-05	0.00039776	0.000210326
6.02E-05	0.000406566	0.000214983
6.04E-05	0.000407057	0.000215242
6.26E-05	0.000420575	0.00022239
6.28E-05	0.000421473	0.000222865
6.40E-05	0.00042892	0.000226803
6.48E-05	0.000433	0.00022896
6.70E-05	0.000446853	0.000236285
6.73E-05	0.000447951	0.000236866
6.75E-05	0.000448557	0.000237187
6.85E-05	0.000454049	0.00024009
6.87E-05	0.000454884	0.000240532
6.91E-05	0.000456733	0.000241509
7.05E-05	0.000464115	0.000245413
7.05E-05	0.000464115	0.000245413
7.13E-05	0.000467508	0.000247208
7.13E-05	0.000467508	0.000247208
7.27E-05	0.000475924	0.000251657
7.30E-05	0.000476536	0.000251981
7.48E-05	0.000487502	0.00025778
7.56E-05	0.00049153	0.000259909
7.67E-05	0.000497965	0.000263312
7.83E-05	0.000504668	0.000266857
7.83E-05	0.000504668	0.000266857
7.83E-05	0.000504668	0.000266857
7.83E-05	0.000504668	0.000266857
8.15E-05	0.000523946	0.00027705
8.26E-05	0.000530353	0.000280438

8.39E-05	0.000536768	0.00028383
8.39E-05	0.000536768	0.00028383
8.55E-05	0.000544517	0.000287928
8.58E-05	0.000544517	0.000287928
8.58E-05	0.000544517	0.000287928
8.58E-05	0.000544517	0.000287928
8.59E-05	0.000544517	0.000287928
8.79E-05	0.000556277	0.000294147
8.87E-05	0.00055854	0.000295343
8.88E-05	0.00055854	0.000295343
8.88E-05	0.00055854	0.000295343
9.31E-05	0.000578232	0.000305756
9.32E-05	0.000578232	0.000305756
9.32E-05	0.000578232	0.000305756
9.32E-05	0.000578232	0.000305756
9.32E-05	0.000578232	0.000305756
9.32E-05	0.000578232	0.000305756
9.32E-05	0.000578232	0.000305756
9.33E-05	0.000578232	0.000305756
9.38E-05	0.000578232	0.000305756
9.38E-05	0.000578232	0.000305756
9.38E-05	0.000578232	0.000305756
9.92E-05	0.000610474	0.000322804
9.99E-05	0.00061357	0.000324442
0.000102297	0.000625105	0.000330541
0.000102297	0.000625105	0.000330541
0.000102297	0.000625105	0.000330541
0.000103639	0.000631055	0.000333687
0.000103639	0.000631055	0.000333687
0.000106861	0.000649518	0.00034345
0.000110496	0.000665048	0.000351662
0.000111357	0.000665048	0.000351662

0.000111357	0.000665048	0.000351662
0.000111357	0.000665048	0.000351662
0.000111357	0.000665048	0.000351662
0.00011136	0.000665048	0.000351662
0.00011136	0.000665048	0.000351662
0.00011136	0.000665048	0.000351662
0.00011136	0.000665048	0.000351662
0.00011136	0.000665048	0.000351662
0.000113506	0.000673428	0.000356093
0.000113506	0.000673428	0.000356093
0.000113506	0.000673428	0.000356093
0.00011355	0.000673428	0.000356093
0.00011613	0.000685166	0.0003623
0.00011613	0.000685166	0.0003623
0.00011613	0.000685166	0.0003623
0.00011702	0.000689229	0.000364448
0.000117938	0.000691069	0.000365421
0.000117938	0.000691069	0.000365421
0.000117938	0.000691069	0.000365421
0.000119041	0.000695153	0.000367581
0.000119041	0.000695153	0.000367581
0.000120477	0.000701749	0.000371069
0.000120991	0.000701749	0.000371069
0.000120991	0.000701749	0.000371069
0.000120991	0.000701749	0.000371069
0.000122507	0.000709338	0.000375081
0.000122768	0.000709647	0.000375245
0.000123038	0.000710012	0.000375438
0.000129282	0.000744787	0.000393826
0.000130999	0.000752109	0.000397698
0.000130999	0.000752109	0.000397698
0.000131213	0.000752109	0.000397698
0.000131728	0.000752542	0.000397927
0.000131728	0.000752542	0.000397927
0.000137809	0.000785971	0.000415603
0.000142207	0.000809703	0.000428152
0.000143103	0.000813451	0.000430134
0.000144581	0.000820491	0.000433857

0.000145461	0.000824118	0.000435774
0.000147504	0.00083431	0.000441164
0.000148069	0.000836128	0.000442125
0.00015351	0.000860395	0.000454957
0.000154378	0.000860395	0.000454957
0.000154378	0.000860395	0.000454957
0.000154378	0.000860395	0.000454957
0.000154378	0.000860395	0.000454957
0.000154378	0.000860395	0.000454957
0.000154378	0.000860395	0.000454957
0.000154378	0.000860395	0.000454957
0.000158027	0.000879298	0.000464952
0.00016121	0.000895552	0.000473547
0.000164338	0.000909974	0.000481173
0.000164338	0.000909974	0.000481173
0.000165818	0.000914303	0.000483462
0.000165818	0.000914303	0.000483462
0.000165921	0.000914303	0.000483462
0.000174485	0.000959951	0.000507599
0.000178425	0.00097609	0.000516134
0.000178425	0.00097609	0.000516134
0.000178558	0.00097609	0.000516134
0.000179416	0.00097609	0.000516134
0.000179416	0.00097609	0.000516134
0.000179416	0.00097609	0.000516134
0.000179416	0.00097609	0.000516134
0.00018261	0.00098901	0.000522965
0.00018261	0.00098901	0.000522965
0.000182658	0.00098901	0.000522965
0.000190491	0.001029796	0.000544532
0.00019142	0.001033187	0.000546325
0.000191919	0.001034248	0.000546886
0.000195741	0.001053187	0.0005569
0.000200111	0.001074001	0.000567907

0.000200551	0.001074001	0.000567907
0.000200551	0.001074001	0.000567907
0.000201156	0.001075555	0.000568728
0.000203627	0.001087072	0.000574818
0.000206946	0.001099643	0.000581466
0.000206946	0.001099643	0.000581466
0.000206946	0.001099643	0.000581466
0.000219806	0.001166165	0.000616641
0.000221093	0.001167561	0.000617379
0.000221093	0.001167561	0.000617379
0.000221093	0.001167561	0.000617379
0.000223933	0.001180739	0.000624347
0.000228755	0.001204307	0.000636809
0.000233841	0.001229191	0.000649968
0.000236817	0.001229193	0.000649968
0.000236817	0.001229193	0.000649968
0.000236817	0.001229193	0.000649968
0.000237074	0.001229193	0.000649968
0.000237074	0.001229193	0.000649968
0.000237074	0.001229193	0.000649968
0.000237074	0.001229193	0.000649968
0.000237074	0.001229193	0.000649968
0.000237074	0.001229193	0.000649968
0.000239019	0.001237404	0.00065431
0.000252047	0.001302879	0.000688932
0.000253331	0.001307537	0.000691395
0.000255287	0.001315652	0.000695686
0.000256223	0.001318489	0.000697186
0.00026251	0.001346791	0.000712152
0.00026251	0.001346791	0.000712152
0.000264749	0.001356245	0.00071715
0.000269901	0.00137825	0.000728786
0.000270656	0.00137825	0.000728786

0.000270656	0.00137825	0.000728786
0.000270656	0.00137825	0.000728786
0.000274107	0.001393752	0.000736984
0.00028609	0.00145252	0.000768059
0.000288816	0.001462023	0.000773084
0.000288816	0.001462023	0.000773084
0.000290584	0.001467999	0.000776243
0.000290854	0.001467999	0.000776243
0.000298465	0.001504195	0.000795383
0.00030246	0.001522085	0.000804843
0.000305528	0.001530772	0.000809436
0.000305528	0.001530772	0.000809436
0.000305528	0.001530772	0.000809436
0.000307834	0.001540071	0.000814353
0.000311243	0.001550326	0.000819776
0.000311243	0.001550326	0.000819776
0.000311243	0.001550326	0.000819776
0.000312734	0.001553232	0.000821313
0.000312734	0.001553232	0.000821313
0.000319155	0.001582823	0.00083696
0.000324108	0.001605064	0.00084872
0.000335532	0.001659236	0.000877365
0.000339395	0.001673499	0.000884907
0.000339395	0.001673499	0.000884907
0.000344054	0.001684337	0.000890638
0.000344054	0.001684337	0.000890638
0.000344054	0.001684337	0.000890638
0.000344054	0.001684337	0.000890638

0.000344054	0.001684337	0.000890638
0.000348539	0.001703857	0.00090096
0.000353622	0.001726239	0.000912795
0.000356755	0.001739052	0.00091957
0.000360453	0.001752936	0.000926912
0.000360627	0.001752936	0.000926912
0.000380142	0.001845174	0.000975685
0.000385575	0.001866247	0.000986828
0.000385575	0.001866247	0.000986828
0.000392959	0.001896621	0.001002889
0.000392959	0.001896621	0.001002889
0.000394412	0.001900956	0.001005181
0.00041267	0.001977812	0.001045821
0.00041267	0.001977812	0.001045821
0.00041267	0.001977812	0.001045821
0.00041267	0.001977812	0.001045821
0.000416621	0.001993953	0.001054356
0.000428978	0.002050229	0.001084113
0.000430187	0.002050277	0.001084138
0.000430187	0.002050277	0.001084138
0.000441283	0.002088619	0.001104413
0.000441283	0.002088619	0.001104413
0.000441283	0.002088619	0.001104413
0.000441283	0.002088619	0.001104413
0.000441283	0.002088619	0.001104413
0.000445768	0.002104025	0.001112559
0.000445768	0.002104025	0.001112559
0.000452896	0.002134724	0.001128792
0.00046697	0.002198034	0.001162269
0.000469383	0.002203332	0.00116507
0.000469383	0.002203332	0.00116507
0.000474167	0.002222739	0.001175332
0.000477983	0.002237561	0.00118317

0.000493705	0.002308002	0.001220418
0.000510811	0.002384716	0.001260982
0.000517929	0.002414648	0.00127681
0.000529055	0.002449832	0.001295414
0.000529055	0.002449832	0.001295414
0.000529055	0.002449832	0.001295414
0.000529055	0.002449832	0.001295414
0.000529055	0.002449832	0.001295414
0.000532453	0.002462235	0.001301973
0.000549794	0.002538995	0.001342561
0.000562123	0.002592431	0.001370817
0.000577565	0.002655204	0.00140401
0.000580441	0.002655204	0.00140401
0.000580441	0.002655204	0.00140401
0.000580441	0.002655204	0.00140401
0.000583493	0.002655204	0.00140401
0.000583493	0.002655204	0.00140401
0.000583493	0.002655204	0.00140401
0.000583493	0.002655204	0.00140401
0.000583493	0.002655204	0.00140401
0.000583493	0.002655204	0.00140401
0.000602182	0.002736609	0.001447055
0.000620093	0.002810541	0.001486149
0.000620093	0.002810541	0.001486149
0.000635302	0.002873708	0.001519549
0.000635709	0.002873708	0.001519549
0.000641386	0.002887925	0.001527067
0.000641386	0.002887925	0.001527067
0.000641386	0.002887925	0.001527067
0.000643952	0.002895667	0.001531161
0.00065472	0.002940225	0.001554722

0.000664928	0.002982154	0.001576894
0.000668882	0.002995962	0.001584195
0.000677486	0.003030533	0.001602475
0.000687615	0.003070588	0.001623655
0.000688235	0.003070588	0.001623655
0.00070282	0.003123443	0.001651604
0.00070282	0.003123443	0.001651604
0.00070282	0.003123443	0.001651604
0.000747265	0.00331236	0.001751499
0.000747265	0.00331236	0.001751499
0.000759048	0.003360237	0.001776815
0.000760577	0.003362657	0.001778094
0.000767881	0.00338184	0.001788238
0.000767881	0.00338184	0.001788238
0.000767881	0.00338184	0.001788238
0.000774969	0.00340867	0.001802425
0.0007855	0.003450555	0.001824573
0.000787629	0.003451046	0.001824832
0.000787629	0.003451046	0.001824832
0.000791154	0.003462058	0.001830655
0.000802216	0.003505981	0.001853881
0.00082267	0.003583131	0.001894676
0.00082267	0.003583131	0.001894676
0.000824057	0.003583131	0.001894676
0.000824057	0.003583131	0.001894676
0.000836652	0.0036195	0.001913907
0.000836652	0.0036195	0.001913907
0.000836652	0.0036195	0.001913907
0.000836652	0.0036195	0.001913907
0.000843524	0.00364462	0.00192719
0.000859213	0.003707727	0.001960559
0.000863931	0.003722379	0.001968307
0.000864784	0.003722379	0.001968307

0.000886456	0.003810869	0.002015099
0.000893969	0.003838346	0.002029628
0.000897373	0.003848131	0.002034802
0.000909216	0.003869821	0.002046271
0.000909216	0.003869821	0.002046271
0.000909216	0.003869821	0.002046271
0.000909216	0.003869821	0.002046271
0.000909216	0.003869821	0.002046271
0.000909216	0.003869821	0.002046271
0.000926502	0.003938499	0.002082586
0.00094773	0.004023735	0.002127657
0.000949188	0.004024934	0.002128291
0.000956331	0.004050203	0.002141653
0.000985651	0.004148708	0.00219374
0.000985651	0.004148708	0.00219374
0.000985651	0.004148708	0.00219374
0.000985651	0.004148708	0.00219374
0.000985651	0.004148708	0.00219374
0.000991703	0.004163936	0.002201792
0.000991703	0.004163936	0.002201792
0.001007065	0.004218087	0.002230426
0.001007065	0.004218087	0.002230426
0.001018124	0.004253995	0.002249413
0.001018124	0.004253995	0.002249413
0.001037092	0.004327963	0.002288526
0.001050111	0.004376954	0.002314431
0.001066039	0.004421799	0.002338144
0.001066039	0.004421799	0.002338144
0.001066039	0.004421799	0.002338144
0.001066039	0.004421799	0.002338144
0.001082838	0.004474283	0.002365896
0.001083922	0.004474283	0.002365896
0.001083922	0.004474283	0.002365896
0.001083922	0.004474283	0.002365896

0.001088523	0.004487862	0.002373077
0.001111153	0.004575649	0.002419496
0.001116315	0.004591384	0.002427817
0.001150455	0.004715209	0.002493292
0.001150455	0.004715209	0.002493292
0.001150555	0.004715209	0.002493292
0.001153548	0.004721821	0.002496789
0.001218023	0.004938477	0.002611351
0.001218023	0.004938477	0.002611351
0.001218023	0.004938477	0.002611351
0.001218023	0.004938477	0.002611351
0.001218023	0.004938477	0.002611351
0.001218023	0.004938477	0.002611351
0.001218023	0.004938477	0.002611351
0.001218023	0.004938477	0.002611351
0.001227254	0.004970017	0.002628029
0.0012333	0.004988596	0.002637853
0.001238975	0.005005634	0.002646863
0.001257952	0.00507631	0.002684234
0.001264612	0.005097176	0.002695268
0.001293615	0.005207943	0.002753839
0.001319381	0.005305429	0.002805387
0.001331674	0.005317373	0.002811703
0.001331674	0.005317373	0.002811703
0.001331674	0.005317373	0.002811703
0.001331674	0.005317373	0.002811703
0.001331674	0.005317373	0.002811703
0.001331674	0.005317373	0.002811703
0.001339489	0.005327903	0.002817271
0.001340539	0.005327903	0.002817271
0.001340539	0.005327903	0.002817271
0.001340539	0.005327903	0.002817271
0.001372592	0.005436353	0.002874617

0.001372592	0.005436353	0.002874617
0.001372592	0.005436353	0.002874617
0.001383688	0.005473967	0.002894506
0.001404945	0.005551643	0.002935579
0.001414753	0.005577518	0.002949261
0.001414753	0.005577518	0.002949261
0.001427012	0.005606369	0.002964517
0.001428625	0.005606369	0.002964517
0.001428625	0.005606369	0.002964517
0.001428625	0.005606369	0.002964517
0.001446348	0.00566942	0.002997857
0.001483554	0.005782144	0.003057463
0.001483554	0.005782144	0.003057463
0.001483554	0.005782144	0.003057463
0.001483554	0.005782144	0.003057463
0.001483554	0.005782144	0.003057463
0.001494769	0.005812614	0.003073574
0.001494769	0.005812614	0.003073574
0.00151337	0.005864951	0.003101249
0.00151337	0.005864951	0.003101249
0.00151337	0.005864951	0.003101249
0.001529898	0.00588899	0.003113961
0.001529898	0.00588899	0.003113961
0.001529898	0.00588899	0.003113961
0.001529898	0.00588899	0.003113961
0.001529898	0.00588899	0.003113961
0.001529898	0.00588899	0.003113961
0.00154889	0.005955397	0.003149075
0.00156528	0.006011661	0.003178826
0.001574271	0.006032647	0.003189923
0.001574271	0.006032647	0.003189923
0.00162478	0.006219237	0.003288587
0.001635565	0.00624451	0.003301951
0.001635565	0.00624451	0.003301951
0.001636857	0.00624451	0.003301951

0.00166991	0.006363508	0.003364875
0.001701154	0.006460988	0.003416419
0.001701154	0.006460988	0.003416419
0.001701154	0.006460988	0.003416419
0.001715954	0.00650997	0.00344232
0.001745692	0.006600838	0.003490369
0.001745692	0.006600838	0.003490369
0.001745692	0.006600838	0.003490369
0.001762924	0.006620543	0.003500789
0.001767186	0.006620543	0.003500789
0.001767186	0.006620543	0.003500789
0.00177412	0.006620543	0.003500789
0.00177412	0.006620543	0.003500789
0.00177412	0.006620543	0.003500789
0.00177412	0.006620543	0.003500789
0.00177412	0.006620543	0.003500789
0.00177412	0.006620543	0.003500789
0.00177412	0.006620543	0.003500789
0.00177412	0.006620543	0.003500789
0.00177412	0.006620543	0.003500789
0.00181083	0.006750176	0.003569336
0.001834976	0.00683274	0.003612994
0.001860348	0.006867432	0.003631338
0.001860348	0.006867432	0.003631338
0.001860348	0.006867432	0.003631338
0.001860348	0.006867432	0.003631338
0.001860348	0.006867432	0.003631338
0.001860348	0.006867432	0.003631338
0.001860348	0.006867432	0.003631338
0.001860348	0.006867432	0.003631338
0.001869026	0.006892032	0.003644346
0.001947713	0.007174462	0.003793688

0.001960285	0.007213006	0.003814069
0.00197593	0.007245112	0.003831046
0.00197593	0.007245112	0.003831046
0.001979597	0.007245112	0.003831046
0.001979597	0.007245112	0.003831046
0.001979597	0.007245112	0.003831046
0.002028888	0.00741758	0.003922243
0.002043844	0.007464284	0.003946939
0.002089453	0.007566251	0.004000857
0.002089453	0.007566251	0.004000857
0.002089453	0.007566251	0.004000857
0.002089453	0.007566251	0.004000857
0.002089453	0.007566251	0.004000857
0.002089453	0.007566251	0.004000857
0.002089453	0.007566251	0.004000857
0.002089453	0.007566251	0.004000857
0.002118577	0.007663604	0.004052335
0.002124208	0.007667763	0.004054534
0.002124208	0.007667763	0.004054534
0.002173357	0.00783691	0.004143975
0.002201153	0.007920449	0.004188148
0.002201153	0.007920449	0.004188148
0.002228358	0.00800666	0.004233735
0.002232131	0.00800666	0.004233735
0.002232131	0.00800666	0.004233735
0.002360777	0.008450395	0.004468371
0.002360777	0.008450395	0.004468371
0.002365541	0.008458602	0.004472711
0.002429287	0.008578969	0.004536358
0.002429287	0.008578969	0.004536358
0.002429287	0.008578969	0.004536358
0.002429287	0.008578969	0.004536358
0.002429287	0.008578969	0.004536358
0.002429287	0.008578969	0.004536358

0.002429287	0.008578969	0.004536358
0.002429287	0.008578969	0.004536358
0.002429287	0.008578969	0.004536358
0.002429287	0.008578969	0.004536358
0.002429287	0.008578969	0.004536358
0.002429287	0.008578969	0.004536358
0.002443501	0.00862027	0.004558198
0.002473832	0.008718284	0.004610025
0.002503793	0.008805735	0.004656267
0.002503793	0.008805735	0.004656267
0.00252206	0.008849523	0.004679421
0.00252206	0.008849523	0.004679421
0.002524002	0.008849523	0.004679421
0.002586209	0.00905835	0.004789844
0.002611772	0.009132199	0.004828894
0.002614896	0.009132199	0.004828894
0.002620637	0.009132199	0.004828894
0.002620637	0.009132199	0.004828894
0.002620637	0.009132199	0.004828894
0.002646946	0.009204739	0.004867251
0.002649523	0.009204739	0.004867251
0.002649523	0.009204739	0.004867251
0.002703613	0.009383126	0.004961578
0.002779516	0.00946062	0.005002555
0.00279336	0.00946062	0.005002555
0.00279336	0.00946062	0.005002555
0.00279336	0.00946062	0.005002555
0.00279336	0.00946062	0.005002555
0.00279336	0.00946062	0.005002555
0.00279336	0.00946062	0.005002555
0.00279336	0.00946062	0.005002555
0.00279336	0.00946062	0.005002555
0.00279336	0.00946062	0.005002555
0.00279336	0.00946062	0.005002555
0.00279336	0.00946062	0.005002555
0.00279336	0.00946062	0.005002555
0.00279336	0.00946062	0.005002555
0.00279336	0.00946062	0.005002555

0.00279336	0.00946062	0.005002555
0.00279336	0.00946062	0.005002555
0.00279336	0.00946062	0.005002555
0.00279336	0.00946062	0.005002555
0.00279336	0.00946062	0.005002555
0.00279336	0.00946062	0.005002555
0.00279336	0.00946062	0.005002555
0.00279336	0.00946062	0.005002555
0.002794373	0.00946062	0.005002555
0.002795057	0.00946062	0.005002555
0.002826454	0.009557436	0.005053749
0.0028872	0.009753207	0.005157268
0.002948183	0.009939589	0.005255823
0.002948183	0.009939589	0.005255823
0.002982117	0.010037491	0.005307591
0.002983088	0.010037491	0.005307591
0.002988604	0.010046171	0.005312181
0.00300661	0.010096781	0.005338942
0.003053281	0.010223411	0.005405901
0.003053281	0.010223411	0.005405901
0.003053281	0.010223411	0.005405901
0.003100257	0.010370558	0.005483709
0.003106378	0.010370758	0.005483814
0.003106378	0.010370758	0.005483814
0.003124659	0.010421621	0.00551071
0.003157626	0.010508478	0.005556638
0.003178314	0.010508478	0.005556638
0.00318141	0.010508478	0.005556638
0.00318141	0.010508478	0.005556638
0.00318141	0.010508478	0.005556638
0.00318141	0.010508478	0.005556638
0.00318141	0.010508478	0.005556638
0.00318141	0.010508478	0.005556638
0.00318141	0.010508478	0.005556638

0.00318141	0.010508478	0.005556638
0.003269696	0.010749928	0.005684311
0.003269696	0.010749928	0.005684311
0.003269696	0.010749928	0.005684311
0.003269696	0.010749928	0.005684311
0.003279639	0.010749928	0.005684311
0.003279639	0.010749928	0.005684311
0.003279639	0.010749928	0.005684311
0.003279639	0.010749928	0.005684311
0.003305135	0.01082313	0.005723019
0.003333699	0.01090623	0.00576696
0.003383145	0.011057425	0.005846908
0.003422263	0.011163952	0.005903237
0.003422263	0.011163952	0.005903237
0.00343819	0.0111733	0.00590818
0.00343819	0.0111733	0.00590818
0.00343819	0.0111733	0.00590818
0.00343819	0.0111733	0.00590818
0.003593177	0.011589663	0.006128343
0.003593177	0.011589663	0.006128343
0.003593177	0.011589663	0.006128343
0.003593177	0.011589663	0.006128343
0.003593177	0.011589663	0.006128343
0.003593177	0.011589663	0.006128343
0.003596792	0.011589663	0.006128343
0.003596792	0.011589663	0.006128343
0.003596792	0.011589663	0.006128343
0.003611909	0.011594703	0.006131008
0.003611909	0.011594703	0.006131008
0.003611909	0.011594703	0.006131008
0.003611909	0.011594703	0.006131008
0.003658481	0.011733199	0.006204242
0.003706977	0.011877598	0.006280596
0.00373976	0.011971432	0.006330213
0.003758062	0.012018773	0.006355247
0.003772966	0.012043927	0.006368547

0.003772966	0.012043927	0.006368547
0.003790905	0.012089913	0.006392864
0.003819432	0.012167449	0.006433863
0.003822328	0.012167449	0.006433863
0.003875795	0.012326182	0.006517797
0.003906196	0.012411331	0.006562822
0.00393418	0.012465523	0.006591478
0.00393418	0.012465523	0.006591478
0.00393418	0.012465523	0.006591478
0.003975225	0.012572293	0.006647935
0.003975225	0.012572293	0.006647935
0.004028404	0.01263538	0.006681294
0.004028404	0.01263538	0.006681294
0.004028404	0.01263538	0.006681294
0.004028404	0.01263538	0.006681294
0.004028404	0.01263538	0.006681294
0.004028404	0.01263538	0.006681294
0.004028404	0.01263538	0.006681294
0.004028404	0.01263538	0.006681294
0.004028404	0.01263538	0.006681294
0.004051243	0.012695379	0.00671302
0.004077878	0.012767154	0.006750973
0.004164604	0.013003969	0.006876195
0.004164918	0.013003969	0.006876195
0.004164918	0.013003969	0.006876195
0.004170642	0.01300997	0.006879369
0.004254897	0.013257581	0.007010299
0.004257768	0.013257581	0.007010299
0.004292399	0.013341136	0.007054481
0.004292399	0.013341136	0.007054481
0.004310431	0.013385022	0.007077687
0.004326139	0.013421621	0.00709704

0.004360029	0.013514511	0.007146158
0.004416536	0.013664906	0.007225683
0.004416536	0.013664906	0.007225683
0.004437343	0.01371688	0.007253166
0.00447334	0.013795108	0.007294531
0.004486837	0.013795108	0.007294531
0.004486837	0.013795108	0.007294531
0.004486837	0.013795108	0.007294531
0.004486837	0.013795108	0.007294531
0.004486837	0.013795108	0.007294531
0.004530629	0.013917246	0.007359115
0.004543073	0.013930464	0.007366104
0.004543073	0.013930464	0.007366104
0.004560604	0.013971697	0.007387907
0.004750077	0.014537966	0.007687337
0.004766686	0.014537966	0.007687337
0.004766686	0.014537966	0.007687337
0.004766686	0.014537966	0.007687337
0.004766686	0.014537966	0.007687337
0.004803436	0.014637006	0.007739707
0.004818876	0.014657951	0.007750782
0.004818876	0.014657951	0.007750782
0.004917792	0.014945546	0.007902856
0.004968221	0.015005518	0.007934568
0.004968221	0.015005518	0.007934568
0.004968221	0.015005518	0.007934568
0.004968221	0.015005518	0.007934568
0.004968221	0.015005518	0.007934568
0.004968221	0.015005518	0.007934568
0.004968221	0.015005518	0.007934568
0.004978317	0.015022753	0.007943681
0.005041897	0.01520121	0.008038045
0.005073655	0.015256631	0.00806735

0.005073655	0.015256631	0.00806735
0.005073655	0.015256631	0.00806735
0.005119942	0.015368808	0.008126666
0.005119942	0.015368808	0.008126666
0.005195541	0.015554805	0.008225017
0.005195541	0.015554805	0.008225017
0.005195541	0.015554805	0.008225017
0.005418397	0.016193672	0.008562836
0.005418397	0.016193672	0.008562836
0.005435542	0.016230737	0.008582435
0.005472306	0.016255408	0.00859548
0.005472306	0.016255408	0.00859548
0.005472306	0.016255408	0.00859548
0.005472306	0.016255408	0.00859548
0.005472306	0.016255408	0.00859548
0.005472306	0.016255408	0.00859548
0.005497826	0.016317052	0.008628076
0.005644323	0.016701621	0.008831427
0.005646924	0.016701621	0.008831427
0.005646924	0.016701621	0.008831427
0.005646924	0.016701621	0.008831427
0.005698118	0.01683848	0.008903795
0.005793406	0.017075829	0.009029299
0.005793406	0.017075829	0.009029299
0.005793406	0.017075829	0.009029299
0.005881162	0.017274966	0.009134598
0.005881162	0.017274966	0.009134598
0.005881162	0.017274966	0.009134598
0.005881162	0.017274966	0.009134598

0.005998842	0.017455813	0.009230226
0.005998842	0.017455813	0.009230226
0.005998842	0.017455813	0.009230226
0.005998842	0.017455813	0.009230226
0.005998842	0.017455813	0.009230226
0.005998842	0.017455813	0.009230226
0.005998842	0.017455813	0.009230226
0.005998842	0.017455813	0.009230226
0.005998842	0.017455813	0.009230226
0.005998842	0.017455813	0.009230226
0.005998842	0.017455813	0.009230226
0.006099413	0.017713858	0.009366674
0.00610305	0.017713858	0.009366674
0.00610305	0.017713858	0.009366674
0.006156228	0.017853061	0.009440282
0.006256376	0.018112791	0.009577621
0.006256376	0.018112791	0.009577621
0.006346799	0.018359043	0.009707833
0.006366913	0.018370639	0.009713965
0.006366913	0.018370639	0.009713965
0.006366913	0.018370639	0.009713965
0.006547582	0.018749644	0.009914374
0.006547582	0.018749644	0.009914374
0.006547582	0.018749644	0.009914374
0.006547582	0.018749644	0.009914374
0.006547582	0.018749644	0.009914374
0.006547582	0.018749644	0.009914374
0.006547582	0.018749644	0.009914374
0.006547582	0.018749644	0.009914374
0.006547582	0.018749644	0.009914374
0.006578317	0.018821906	0.009952584
0.006618499	0.018858038	0.00997169
0.006618499	0.018858038	0.00997169

0.006618499	0.018858038	0.00997169
0.006618499	0.018858038	0.00997169
0.006618499	0.018858038	0.00997169
0.006743333	0.019197742	0.010151318
0.00684202	0.019462504	0.010291318
0.006875937	0.01952652	0.010325168
0.006875937	0.01952652	0.010325168
0.006911072	0.019610024	0.010369323
0.007012552	0.019881484	0.010512865
0.007081553	0.020043899	0.010598746
0.007081553	0.020043899	0.010598746
0.00711828	0.020064871	0.010609835
0.00711828	0.020064871	0.010609835
0.00711828	0.020064871	0.010609835
0.00711828	0.020064871	0.010609835
0.00711828	0.020064871	0.010609835
0.007254794	0.02041604	0.010795525
0.007254794	0.02041604	0.010795525
0.007408501	0.020797285	0.010997119
0.007408501	0.020797285	0.010997119
0.007408501	0.020797285	0.010997119
0.007462157	0.020930739	0.011067686
0.007609629	0.021314305	0.011270507
0.007611362	0.021314305	0.011270507
0.00768369	0.021347889	0.011288265
0.007710693	0.021347889	0.011288265
0.007710693	0.021347889	0.011288265
0.007710693	0.021347889	0.011288265
0.007710693	0.021347889	0.011288265
0.007710693	0.021347889	0.011288265
0.007710693	0.021347889	0.011288265
0.007710693	0.021347889	0.011288265
0.007710693	0.021347889	0.011288265
0.007710693	0.021347889	0.011288265

0.007710693	0.021347889	0.011288265
0.007710693	0.021347889	0.011288265
0.007710693	0.021347889	0.011288265
0.007710693	0.021347889	0.011288265
0.007747756	0.021433162	0.011333356
0.007791261	0.02151872	0.011378596
0.007791261	0.02151872	0.011378596
0.007803332	0.021534679	0.011387035
0.007964857	0.021922032	0.011591858
0.007964857	0.021922032	0.011591858
0.007964857	0.021922032	0.011591858
0.007975725	0.021922032	0.011591858
0.007975725	0.021922032	0.011591858
0.008163039	0.022400899	0.011845072
0.008163039	0.022400899	0.011845072
0.008252031	0.022590761	0.011945467
0.008252031	0.022590761	0.011945467
0.008252031	0.022590761	0.011945467
0.00832458	0.022698575	0.012002476
0.00832458	0.022698575	0.012002476
0.00832458	0.022698575	0.012002476
0.00832458	0.022698575	0.012002476
0.00832458	0.022698575	0.012002476
0.008353222	0.02275854	0.012034185
0.008546291	0.023247541	0.012292757
0.008546291	0.023247541	0.012292757
0.008742262	0.023761733	0.012564649
0.008844513	0.024020573	0.012701518
0.008941154	0.024122811	0.012755579
0.0089597	0.024122811	0.012755579
0.0089597	0.024122811	0.012755579
0.0089597	0.024122811	0.012755579
0.0089597	0.024122811	0.012755579
0.0089597	0.024122811	0.012755579
0.0089597	0.024122811	0.012755579

0.0089597	0.024122811	0.012755579
0.0089597	0.024122811	0.012755579
0.0089597	0.024122811	0.012755579
0.0089597	0.024122811	0.012755579
0.009149873	0.02460151	0.013008704
0.009151877	0.02460151	0.013008704
0.009461347	0.025393512	0.013427496
0.009461347	0.025393512	0.013427496
0.009487417	0.025443528	0.013453943
0.009519701	0.025510116	0.013489154
0.009615816	0.025647173	0.013561626
0.009615816	0.025647173	0.013561626
0.009615816	0.025647173	0.013561626
0.009615816	0.025647173	0.013561626
0.009615816	0.025647173	0.013561626
0.009615816	0.025647173	0.013561626
0.009678941	0.025795432	0.013640022
0.009778958	0.026041708	0.013770247
0.009879641	0.026289371	0.013901205
0.009979996	0.026535777	0.014031499
0.010012919	0.026582008	0.014055945
0.010012919	0.026582008	0.014055945
0.010196755	0.027007196	0.014280774
0.010196755	0.027007196	0.014280774
0.010196755	0.027007196	0.014280774
0.010292693	0.027031155	0.014293444
0.010292693	0.027031155	0.014293444
0.010292693	0.027031155	0.014293444
0.010292693	0.027031155	0.014293444
0.010292693	0.027031155	0.014293444
0.010292693	0.027031155	0.014293444
0.010292693	0.027031155	0.014293444
0.010292693	0.027031155	0.014293444
0.010292693	0.027031155	0.014293444
0.010292693	0.027031155	0.014293444

0.010292693	0.027031155	0.014293444
0.010432687	0.027356825	0.01446565
0.010432687	0.027356825	0.01446565
0.010503516	0.027521464	0.014552708
0.010639394	0.027813603	0.014707184
0.010639394	0.027813603	0.014707184
0.010639394	0.027813603	0.014707184
0.010742233	0.028002281	0.014806952
0.010768849	0.028002281	0.014806952
0.010768849	0.028002281	0.014806952
0.010768849	0.028002281	0.014806952
0.010768849	0.028002281	0.014806952
0.010768849	0.028002281	0.014806952
0.010768849	0.028002281	0.014806952
0.010845346	0.028158402	0.014889506
0.010845346	0.028158402	0.014889506
0.010865304	0.028188832	0.014905596
0.010990096	0.028213134	0.014918446
0.010990096	0.028213134	0.014918446
0.010990096	0.028213134	0.014918446
0.010990096	0.028213134	0.014918446
0.010990096	0.028213134	0.014918446
0.010990096	0.028213134	0.014918446
0.010990096	0.028213134	0.014918446
0.010990096	0.028213134	0.014918446
0.010990096	0.028213134	0.014918446
0.010990096	0.028213134	0.014918446
0.010990096	0.028213134	0.014918446
0.010990096	0.028213134	0.014918446
0.010990096	0.028213134	0.014918446
0.010990096	0.028213134	0.014918446
0.011094295	0.028417527	0.015026525
0.011094295	0.028417527	0.015026525

0.011111236	0.028417527	0.015026525
0.011111236	0.028417527	0.015026525
0.011111236	0.028417527	0.015026525
0.011125735	0.028433358	0.015034896
0.011326381	0.028924535	0.015294618
0.011373479	0.029023151	0.015346765
0.011417115	0.029112793	0.015394165
0.01145987	0.029113343	0.015394456
0.01145987	0.029113343	0.015394456
0.01145987	0.029113343	0.015394456
0.01145987	0.029113343	0.015394456
0.01145987	0.029113343	0.015394456
0.01155336	0.029329077	0.015508531
0.011608984	0.029448438	0.015571646
0.011707792	0.029589413	0.015646191
0.011707792	0.029589413	0.015646191
0.011707792	0.029589413	0.015646191
0.011707792	0.029589413	0.015646191
0.011707792	0.029589413	0.015646191
0.011814767	0.029815732	0.015765863
0.011814767	0.029815732	0.015765863
0.01191893	0.030056433	0.01589314
0.012041364	0.03034282	0.016044574
0.012175947	0.030591844	0.016176253
0.012175947	0.030591844	0.016176253
0.012175947	0.030591844	0.016176253
0.012175947	0.030591844	0.016176253
0.012285982	0.030845656	0.016310463
0.012445553	0.031086629	0.016437883
0.012445553	0.031086629	0.016437883
0.012445553	0.031086629	0.016437883

0.012445553	0.031086629	0.016437883
0.012445553	0.031086629	0.016437883
0.012445553	0.031086629	0.016437883
0.012445553	0.031086629	0.016437883
0.012543428	0.031308249	0.016555071
0.01267357	0.031587004	0.01670247
0.01267357	0.031587004	0.01670247
0.012917224	0.032170845	0.017011192
0.013203148	0.032645357	0.017262102
0.013203148	0.032645357	0.017262102
0.013203148	0.032645357	0.017262102
0.013203148	0.032645357	0.017262102
0.013203148	0.032645357	0.017262102
0.013203148	0.032645357	0.017262102
0.013203148	0.032645357	0.017262102
0.013203148	0.032645357	0.017262102
0.013203148	0.032645357	0.017262102
0.013203148	0.032645357	0.017262102
0.013296276	0.032807167	0.017347664
0.013297353	0.032807167	0.017347664
0.013297353	0.032807167	0.017347664
0.013556874	0.033423359	0.017673491
0.013683828	0.033712065	0.017826153
0.013820708	0.034024794	0.017991516
0.013924154	0.034254819	0.018113148
0.013980353	0.03426989	0.018121118
0.013980353	0.03426989	0.018121118
0.013980353	0.03426989	0.018121118
0.013980353	0.03426989	0.018121118
0.013980353	0.03426989	0.018121118
0.014076664	0.034456612	0.018219852
0.014076664	0.034456612	0.018219852

0.014087792	0.034459203	0.018221222
0.014216829	0.034749991	0.018374984
0.014358139	0.035070344	0.018544379
0.0147006	0.035837484	0.018950025
0.014776941	0.035837484	0.018950025
0.014776941	0.035837484	0.018950025
0.014776941	0.035837484	0.018950025
0.014776941	0.035837484	0.018950025
0.014776941	0.035837484	0.018950025
0.014776941	0.035837484	0.018950025
0.014776941	0.035837484	0.018950025
0.014776941	0.035837484	0.018950025
0.014776941	0.035837484	0.018950025
0.01488147	0.036039909	0.019057062
0.01488147	0.036039909	0.019057062
0.015348792	0.03714538	0.019641609
0.015592689	0.037576184	0.019869409
0.015592689	0.037576184	0.019869409
0.015592689	0.037576184	0.019869409
0.015592689	0.037576184	0.019869409
0.015592689	0.037576184	0.019869409
0.015592689	0.037576184	0.019869409
0.015711735	0.037757032	0.019965037
0.015711868	0.037757032	0.019965037
0.015711868	0.037757032	0.019965037
0.015711868	0.037757032	0.019965037
0.015759245	0.037817767	0.019997152
0.015759245	0.037817767	0.019997152
0.015791408	0.037868395	0.020023923
0.016049425	0.038406387	0.0203084
0.016049425	0.038406387	0.0203084
0.016049425	0.038406387	0.0203084

0.016136691	0.038534374	0.020376077
0.016136691	0.038534374	0.020376077
0.016136691	0.038534374	0.020376077
0.016427377	0.039092131	0.020671006
0.016427377	0.039092131	0.020671006
0.016427377	0.039092131	0.020671006
0.016427377	0.039092131	0.020671006
0.016427377	0.039092131	0.020671006
0.016471228	0.039169245	0.020711782
0.016567943	0.039344553	0.020804481
0.016567943	0.039344553	0.020804481
0.016940094	0.040200417	0.021257042
0.017005632	0.040327978	0.021324493
0.017280785	0.040810797	0.021579796
0.017280785	0.040810797	0.021579796
0.017280785	0.040810797	0.021579796
0.017280785	0.040810797	0.021579796
0.017280785	0.040810797	0.021579796
0.017280785	0.040810797	0.021579796
0.017449766	0.041181448	0.021775788
0.017550757	0.041391241	0.021886722
0.017900353	0.042128617	0.022276629
0.017900353	0.042128617	0.022276629
0.017900353	0.042128617	0.022276629
0.018152694	0.042546932	0.022497824
0.018152694	0.042546932	0.022497824
0.018152694	0.042546932	0.022497824
0.018152694	0.042546932	0.022497824
0.018152694	0.042546932	0.022497824
0.018152694	0.042546932	0.022497824
0.018175027	0.042570119	0.022510085
0.0183574	0.042938497	0.022704874
0.0183574	0.042938497	0.022704874
0.018812987	0.043932577	0.023230521
0.018820911	0.043932577	0.023230521

0.018820911	0.043932577	0.023230521
0.018877612	0.044034894	0.023284623
0.01904289	0.044329775	0.02344055
0.01904289	0.044329775	0.02344055
0.01904289	0.044329775	0.02344055
0.019290893	0.044846085	0.023713562
0.019290893	0.044846085	0.023713562
0.01976735	0.04592252	0.024282756
0.019951158	0.046130312	0.024392631
0.019951158	0.046130312	0.024392631
0.019951158	0.046130312	0.024392631
0.019951158	0.046130312	0.024392631
0.019951158	0.046130312	0.024392631
0.019951158	0.046130312	0.024392631
0.019951158	0.046130312	0.024392631
0.020250286	0.04679033	0.024741634
0.020809783	0.048044429	0.025404772
0.020877284	0.048044429	0.025404772
0.020877284	0.048044429	0.025404772
0.020877284	0.048044429	0.025404772
0.020877284	0.048044429	0.025404772
0.020877284	0.048044429	0.025404772
0.02115477	0.048650285	0.025725135
0.021235607	0.04880339	0.025806093
0.021737997	0.049857524	0.026363494
0.021737997	0.049857524	0.026363494
0.021737997	0.049857524	0.026363494
0.021821059	0.049947602	0.026411125
0.021821059	0.049947602	0.026411125
0.021821059	0.049947602	0.026411125
0.022246875	0.050854245	0.026890537
0.022246875	0.050854245	0.026890537
0.022762241	0.051801288	0.027391311
0.022782273	0.051801288	0.027391311
0.022782273	0.051801288	0.027391311
0.022782273	0.051801288	0.027391311
0.022782273	0.051801288	0.027391311
0.022782273	0.051801288	0.027391311

0.022782273	0.051801288	0.027391311
0.022782273	0.051801288	0.027391311
0.023284096	0.052907157	0.027976069
0.023668297	0.05374037	0.028416653
0.023760719	0.05374037	0.028416653
0.023760719	0.05374037	0.028416653
0.023760719	0.05374037	0.028416653
0.023760719	0.05374037	0.028416653
0.023760719	0.05374037	0.028416653
0.023760719	0.05374037	0.028416653
0.02475619	0.055734002	0.029470839
0.02475619	0.055734002	0.029470839
0.02475619	0.055734002	0.029470839
0.02475619	0.055734002	0.029470839
0.02475619	0.055734002	0.029470839
0.02475619	0.055734002	0.029470839
0.02475619	0.055734002	0.029470839
0.024798773	0.055756505	0.029482738
0.024798773	0.055756505	0.029482738
0.024828837	0.055787445	0.029499098
0.024888588	0.055885006	0.029550686
0.02518275	0.056471409	0.029860762
0.02518275	0.056471409	0.029860762
0.025436389	0.056965525	0.030122039
0.025436389	0.056965525	0.030122039
0.025569996	0.057227291	0.030260455
0.025768483	0.05748354	0.030395953
0.025768483	0.05748354	0.030395953
0.025768483	0.05748354	0.030395953
0.025768483	0.05748354	0.030395953
0.025768483	0.05748354	0.030395953
0.02599067	0.057903692	0.03061812
0.02599067	0.057903692	0.03061812
0.026188176	0.058305751	0.030830719
0.026551428	0.059076064	0.031238043
0.026797395	0.059123589	0.031263173
0.026797395	0.059123589	0.031263173

0.026797395	0.059123589	0.031263173
0.026797395	0.059123589	0.031263173
0.026797395	0.059123589	0.031263173
0.026797395	0.059123589	0.031263173
0.026797395	0.059123589	0.031263173
0.026797395	0.059123589	0.031263173
0.026797395	0.059123589	0.031263173
0.026797395	0.059123589	0.031263173
0.026797395	0.059123589	0.031263173
0.026797395	0.059123589	0.031263173
0.026797395	0.059123589	0.031263173
0.027118659	0.059793848	0.03161759
0.027562152	0.060732571	0.032113965
0.02769236	0.060941	0.032224178
0.02769236	0.060941	0.032224178
0.027842726	0.061036392	0.032274619
0.027842726	0.061036392	0.032274619
0.027842726	0.061036392	0.032274619
0.027842726	0.061036392	0.032274619
0.027842726	0.061036392	0.032274619
0.027842726	0.061036392	0.032274619
0.028272524	0.061899282	0.032730895
0.028272524	0.061899282	0.032730895
0.028859147	0.063000274	0.033313073
0.028904275	0.063000274	0.033313073
0.028904275	0.063000274	0.033313073
0.028904275	0.063000274	0.033313073
0.028904275	0.063000274	0.033313073
0.028904275	0.063000274	0.033313073
0.028904275	0.063000274	0.033313073
0.029981846	0.065251943	0.034503704
0.029981846	0.065251943	0.034503704

0.030051742	0.065251943	0.034503704
0.030051742	0.065251943	0.034503704
0.030051742	0.065251943	0.034503704
0.030051742	0.065251943	0.034503704
0.030657702	0.066483305	0.035154819
0.030657702	0.066483305	0.035154819
0.030784408	0.066715797	0.035277755
0.031075242	0.067175918	0.035521056
0.031075242	0.067175918	0.035521056
0.031075242	0.067175918	0.035521056
0.031075242	0.067175918	0.035521056
0.031270092	0.067469266	0.035676172
0.031270092	0.067469266	0.035676172
0.031270092	0.067469266	0.035676172
0.03137155	0.067645523	0.035769373
0.031888906	0.06871778	0.036336357
0.031963107	0.06883433	0.036397986
0.032184269	0.069093204	0.036534873
0.032184269	0.069093204	0.036534873
0.032184269	0.069093204	0.036534873
0.032184269	0.069093204	0.036534873
0.032184269	0.069093204	0.036534873
0.032514134	0.069713888	0.036863076
0.032514134	0.069713888	0.036863076
0.033145767	0.071023679	0.037555663
0.033308733	0.071239054	0.037669548
0.033308733	0.071239054	0.037669548
0.033308733	0.071239054	0.037669548
0.033783796	0.072164889	0.038159108
0.033783796	0.072164889	0.038159108
0.034448445	0.07344709	0.038837106
0.034448445	0.07344709	0.038837106
0.034448445	0.07344709	0.038837106
0.035079003	0.074744924	0.03952337
0.035603213	0.075720445	0.040039203

0.035603213	0.075720445	0.040039203
0.035603213	0.075720445	0.040039203
0.035736159	0.075955985	0.040163751
0.03598659	0.076440789	0.040420104
0.036399668	0.077222358	0.04083338
0.036399668	0.077222358	0.04083338
0.036772851	0.077965734	0.041226459
0.037069519	0.078497459	0.041507623
0.037069519	0.078497459	0.041507623
0.037409038	0.079167427	0.041861887
0.037745699	0.079781213	0.042186442
0.037745699	0.079781213	0.042186442
0.037791724	0.079829184	0.042211808
0.03795717	0.0800798	0.042344328
0.03795717	0.0800798	0.042344328
0.038392511	0.080948351	0.042803598
0.038428197	0.0809737	0.042817002
0.039155986	0.082153149	0.043440666
0.039155986	0.082153149	0.043440666
0.039155986	0.082153149	0.043440666
0.039155986	0.082153149	0.043440666
0.039155986	0.082153149	0.043440666
0.039155986	0.082153149	0.043440666
0.039155986	0.082153149	0.043440666
0.03981209	0.083478537	0.044141501
0.040369116	0.084233607	0.044540764
0.040369116	0.084233607	0.044540764
0.040369116	0.084233607	0.044540764
0.040369116	0.084233607	0.044540764
0.040369116	0.084233607	0.044540764
0.040369116	0.084233607	0.044540764
0.040369116	0.084233607	0.044540764
0.040369116	0.084233607	0.044540764
0.040513459	0.084483276	0.044672783
0.04122109	0.085906559	0.045425382

0.041426882	0.086240437	0.045601929
0.041431701	0.086240437	0.045601929
0.041596377	0.08632068	0.04564436
0.041596377	0.08632068	0.04564436
0.041596377	0.08632068	0.04564436
0.041596377	0.08632068	0.04564436
0.041596377	0.08632068	0.04564436
0.041934969	0.086917907	0.045960159
0.041934969	0.086917907	0.045960159
0.042262595	0.087543946	0.046291194
0.042655081	0.088254201	0.046666761
0.042837589	0.088254201	0.046666761
0.042837589	0.088254201	0.046666761
0.042837589	0.088254201	0.046666761
0.042837589	0.088254201	0.046666761
0.042837589	0.088254201	0.046666761
0.042837589	0.088254201	0.046666761
0.042837589	0.088254201	0.046666761
0.042837589	0.088254201	0.046666761
0.043381411	0.089320811	0.047230759
0.043514477	0.089540914	0.047347144
0.043964593	0.090393958	0.047798214
0.044092572	0.090393958	0.047798214
0.044092572	0.090393958	0.047798214
0.044092572	0.090393958	0.047798214
0.044092572	0.090393958	0.047798214
0.044092572	0.090393958	0.047798214
0.044113942	0.090393958	0.047798214
0.044604122	0.091343689	0.04830041
0.044852659	0.091688052	0.048482501
0.044852659	0.091688052	0.048482501
0.044852659	0.091688052	0.048482501
0.045361149	0.092341375	0.048827962
0.045361149	0.092341375	0.048827962
0.045361149	0.092341375	0.048827962
0.045361149	0.092341375	0.048827962

0.045361149	0.092341375	0.048827962
0.045361149	0.092341375	0.048827962
0.045361149	0.092341375	0.048827962
0.045597546	0.092657245	0.048994988
0.045597546	0.092657245	0.048994988
0.045597546	0.092657245	0.048994988
0.045707457	0.092825471	0.049083941
8.37E-12	9.67E-10	7.45E-10
8.37E-12	9.67E-10	7.45E-10
2.07E-09	1.60E-07	1.23E-07
3.66E-07	2.11E-05	1.63E-05
1.06E-06	4.90E-05	3.77E-05
2.28E-05	0.00087747	0.000675746
4.75E-05	0.00156716	0.001206881
6.26E-05	0.001806601	0.001391276
0.000138352	0.00312913	0.002409765
0.000146309	0.00312913	0.002409765
0.000149006	0.00312913	0.002409765
0.000214454	0.004128244	0.00317919
0.000242175	0.00412913	0.003179872
0.000252437	0.00412913	0.003179872
0.000284138	0.00412913	0.003179872
0.000286	0.00412913	0.003179872
0.000323186	0.004391528	0.003381946
0.000443687	0.00542714	0.004179479
0.000446388	0.00542714	0.004179479
0.000503647	0.005817118	0.004479804
0.001021499	0.011236485	0.008653297
0.00107903	0.011329815	0.008725171
0.001295485	0.012427169	0.009570251
0.001301286	0.012427169	0.009570251
0.001344932	0.012427169	0.009570251
0.001443435	0.012824368	0.009876136
0.002580475	0.021761751	0.016758878
0.002637788	0.021761751	0.016758878
0.002821571	0.022475274	0.017308368
0.003310529	0.025491072	0.019630855
0.003963813	0.029536801	0.0227465
0.004120189	0.029742614	0.022904998
0.00464864	0.032540481	0.025059655

0.005614263	0.038143961	0.029374935
0.005794934	0.038246565	0.029453951
0.008059109	0.051712617	0.039824253
0.009819972	0.061308471	0.047214088
0.01008987	0.061335792	0.047235128
3.62E-15	7.67E-13	5.05E-13
3.62E-15	7.67E-13	5.05E-13
1.97E-11	2.79E-09	1.83E-09
7.00E-11	7.42E-09	4.88E-09
1.51E-09	1.28E-07	8.44E-08
2.72E-09	1.93E-07	1.27E-07
4.76E-09	2.89E-07	1.90E-07
6.29E-09	3.33E-07	2.19E-07
2.71E-08	1.28E-06	8.41E-07
6.64E-08	2.36E-06	1.56E-06
7.11E-08	2.36E-06	1.56E-06
7.19E-08	2.36E-06	1.56E-06
7.25E-08	2.36E-06	1.56E-06
1.30E-07	3.94E-06	2.59E-06
2.26E-07	6.11E-06	4.02E-06
2.30E-07	6.11E-06	4.02E-06
7.44E-07	1.86E-05	1.22E-05
9.85E-07	2.32E-05	1.53E-05
1.07E-06	2.32E-05	1.53E-05
1.10E-06	2.32E-05	1.53E-05
1.31E-06	2.65E-05	1.74E-05
2.43E-06	4.55E-05	2.99E-05
2.47E-06	4.55E-05	2.99E-05
3.03E-06	5.35E-05	3.52E-05
5.74E-06	9.73E-05	6.40E-05

7.47E-06	0.00011944	7.86E-05
7.61E-06	0.00011944	7.86E-05
9.46E-06	0.000143222	9.42E-05
1.00E-05	0.00014625	9.62E-05
1.15E-05	0.0001629	0.000107171
1.50E-05	0.000204705	0.000134675
1.66E-05	0.00021931	0.000144283
1.84E-05	0.000235994	0.000155259
2.52E-05	0.000313701	0.000206382
2.73E-05	0.000330582	0.000217488
2.97E-05	0.000349224	0.000229752
3.34E-05	0.000382831	0.000251863
3.83E-05	0.000427207	0.000281057
4.51E-05	0.000490555	0.000322734
5.33E-05	0.000565016	0.000371721
6.72E-05	0.000694676	0.000457024
7.22E-05	0.000712105	0.00046849
7.22E-05	0.000712105	0.00046849
8.16E-05	0.000785893	0.000517035
0.000101277	0.000954251	0.000627797
0.000104492	0.000963143	0.000633647
0.000115125	0.001038576	0.000683273
0.000198619	0.00175447	0.001154256
0.000263499	0.002280069	0.001500046
0.000293075	0.002485272	0.001635047
0.000331724	0.002757862	0.001814383
0.000342359	0.00279154	0.001836539
0.000396134	0.00316907	0.002084915
0.000418542	0.003286329	0.002162059
0.00054857	0.004153456	0.002732537
0.00054857	0.004153456	0.002732537
0.000574101	0.004270505	0.002809543
0.000633105	0.004628215	0.003044879
0.000695844	0.005000642	0.003289896
0.000790753	0.005587991	0.00367631

0.000830623	0.005680388	0.003737097
0.000830623	0.005680388	0.003737097
0.000907392	0.006106892	0.004017692
0.000958727	0.006351568	0.004178663
0.001083457	0.007067472	0.004649653
0.001150361	0.007390198	0.004861972
0.001247181	0.007892611	0.005192507
0.001287746	0.008029475	0.005282549
0.001342992	0.008252589	0.005429335
0.001485929	0.009000487	0.005921373
0.001690918	0.010097879	0.006643341
0.001772112	0.010435772	0.00686564
0.001875305	0.010601722	0.006974817
0.001875305	0.010601722	0.006974817
0.001875305	0.010601722	0.006974817
0.001957284	0.010919586	0.007183938
0.002144167	0.01180684	0.007767658
0.002208404	0.0118527	0.007797829
0.002208404	0.0118527	0.007797829
0.002278127	0.012074072	0.007943469
0.002951802	0.015451408	0.0101654
0.003025448	0.015643781	0.010291961
0.003361531	0.01677562	0.011036592
0.003361531	0.01677562	0.011036592
0.003363037	0.01677562	0.011036592
0.003513411	0.017242965	0.011344056
0.003538061	0.017242965	0.011344056
0.003796238	0.018085446	0.01189832
0.003796238	0.018085446	0.01189832
0.004255642	0.020033477	0.013179919
0.004299638	0.020033477	0.013179919
0.004504325	0.020759062	0.013657277
0.004605667	0.020997878	0.013814393
0.005092929	0.022884039	0.015055289
0.00512732	0.022884039	0.015055289
0.005247443	0.022937275	0.015090313
0.005247443	0.022937275	0.015090313
0.005474871	0.023687199	0.015583684
0.006102498	0.026047624	0.017136595
0.006334744	0.026047624	0.017136595
0.006334744	0.026047624	0.017136595

0.006334744	0.026047624	0.017136595
0.006354949	0.026047624	0.017136595
0.00638904	0.026047624	0.017136595
0.006555588	0.026472087	0.017415847
0.006714798	0.026859192	0.017670521
0.006878355	0.027181774	0.017882746
0.006923659	0.027181774	0.017882746
0.007426673	0.028645494	0.018845719
0.007431614	0.028645494	0.018845719
0.008140059	0.030988294	0.020387036
0.008185587	0.030988294	0.020387036
0.008535145	0.031844472	0.02095031
0.008787272	0.031844472	0.02095031
0.008787272	0.031844472	0.02095031
0.008787272	0.031844472	0.02095031
0.008787272	0.031844472	0.02095031
0.009179524	0.032767479	0.021557552
0.009196533	0.032767479	0.021557552
0.00931365	0.032908229	0.021650151
0.010861598	0.037267848	0.024518321
0.010861598	0.037267848	0.024518321
0.010899088	0.037267848	0.024518321
0.010899088	0.037267848	0.024518321
0.011253853	0.038173069	0.025113861
0.011983257	0.040324611	0.026529349
0.013129621	0.043834325	0.028838372
0.015584617	0.051624044	0.033963187
0.01644336	0.053732075	0.035350049
0.016474457	0.053732075	0.035350049
0.016977619	0.054950461	0.036151619
0.017321909	0.055640072	0.03660531
0.01784169	0.05687877	0.037420243
0.019137478	0.059993708	0.039469545
0.019137478	0.059993708	0.039469545
0.019243265	0.059993708	0.039469545
0.020074029	0.062126923	0.040872975
0.020257173	0.06223943	0.040946993
0.020760833	0.063328007	0.041663163
0.020971043	0.063512303	0.04178441
0.023166732	0.0696645	0.045831908
0.023926952	0.071443858	0.047002538

geneID	Count
TP53/IL1B/SLC6A2/PPARG/SOD1/FOS/CASP3/F7/CHEK2/CYP3A4/EGFR/KCNH2/NOS2/MYC/ICAM1/CCND1/JUN/BCL2/CYP2B6/CDKN1A/CYP1A1/NR1I2/NQO1/CDK4/CCNB1/PDE3A/NCOA1	27
PLAU/TP53/CHRNA7/AKT1/PPARG/MMP2/PTGS2/CASP3/F7/NOS2/PPARA/MYC/ICAM1/CAV1/MDM2/SLC2A4/BCL2/CDKN1A/CYP1A1/DPP4/PPARD/CDK4/CCNB1/E2F1	24
TP53/NOS3/MAPK1/AKT1/PPARG/SOD1/MMP9/MPO/FOS/MMP2/CASP3/EGFR/PCNA/CYP1B1/G6PD/HSPB1/CAV1/JUN/SLC2A4/BCL2/NQO1/MCL1/AKR1C3	23
TP53/IL1B/F2/NOS3/AKT1/SOD1/MPO/PTGS2/EGFR/NOS2/PPARA/CYP1B1/G6PD/ICAM1/CAV1/BCL2/CDKN1A/HSP90AA1/CYP1A1/NQO1/AKR1C3	21
MAPK1/AKT1/SOD1/MMP9/FOS/CASP3/EGFR/PCNA/IL1A/G6PD/ICAM1/CAV1/CCND1/JUN/BCL2/CYP1A1/NQO1/CD14/PTGES/CDK4/CCNB1/AKR1C3	22
AR/NR3C1/AKT1/PPARG/FOS/F7/EGFR/PCNA/ICAM1/CAV1/CCND1/CDKN1A/NQO1/AHR/CDK4/AKR1C3/NCOA2/NCOA1	18
IL1B/NOS3/MAPK1/AKT1/SERPINE1/CXCL8/GJA1/CCL2/MPO/FOS/CASP3/NOS2/IL1A/ICAM1/JUN/CYP1A1/SLPI/PPARD/CD14/PTGES/CDK4	21
IL1B/NOS3/MAPK1/AKT1/SERPINE1/CXCL8/GJA1/CCL2/MPO/FOS/CASP3/NOS2/IL1A/ICAM1/JUN/CYP1A1/SLPI/PPARD/CD14/PTGES/CDK4	21
PLAU/TP53/CHRNA7/AKT1/MMP2/PTGS2/CASP3/F7/NOS2/PPARA/MYC/ICAM1/CAV1/MDM2/SLC2A4/BCL2/CYP1A1/DPP4/PPARD/CCNB1/E2F1	21
ESR1/AR/TP53/NR3C2/NR3C1/PPARG/ESR2/PGR/RUNX2/PPARA/CCND1/CDKN1A/NR1I2/PPARD/CDK4/CCNB1/CDK	17
PLAU/TP53/CHRNA7/AKT1/MMP2/PTGS2/CASP3/F7/NOS2/PPARA/MYC/ICAM1/CAV1/MDM2/SLC2A4/BCL2/CYP1A1/DPP4/PPARD/CCNB1/E2F1	21
TP53/MAPK1/AKT1/PPARG/SOD1/MPO/FOS/F7/EGFR/PON1/PPARA/ADRB2/G6PD/ICAM1/CCND1/JUN/BCL2/CDKN1A/CYP1A1/PPARD/NQO1/AKR1C3/NCOA1	23
NOS3/MAPK1/SOD1/MPO/FOS/PTGS2/PON1/GSTM1/CYP1B1/BCL2/PTGS1/CDKN1A/CYP1A1/NQO1/AHR/PTGES/CDK4/CCNB1	18
ESR1/AR/NR3C2/NR3C1/ESR2/FOS/CASP3/EGFR/PGR/PCNA/PPARA/ICAM1/CAV1/CCND1/BCL2/CDKN1A/PPARD/AKR1C3/NCOA2/NCOA1	20
ESR1/AR/TP53/NR3C2/NR3C1/PPARG/ESR2/PGR/RUNX2/PPARA/CCND1/JUN/CDKN1A/NR1I2/PPARD/CDK4/CCNB1/CDK1	18
TP53/AKT1/CCL2/CHEK2/EGFR/PCNA/RB1/MDM2/CCND1/BCL2/CDKN1A/CYP1A1/CDK4/CCNB1/CDK1/E2F1	16
TP53/NOS3/MAPK1/AKT1/SOD1/MMP9/MPO/FOS/MMP2/PTGS2/CASP3/EGFR/PCNA/CYP1B1/G6PD/HSPB1/JUN/BCL2/PTGS1/NQO1/MCL1/AKR1C3	22
TP53/NOS3/MAPK1/AKT1/SOD1/MMP9/MPO/FOS/MMP2/EGFR/PCNA/CYP1B1/G6PD/HSPB1/JUN/BCL2/NQO1/MCL1/AKR1C3	19
TP53/MAPK1/AKT1/PPARG/SOD1/MPO/F7/EGFR/PON1/PPARA/ADRB2/G6PD/ICAM1/CCND1/JUN/BCL2/CDKN1A/CYP1A1/PPARD/NQO1/AKR1C3/NCOA1	22

IL1B/NOS3/AKT1/SOD1/MPO/PTGS2/NOS2/PPARA/CYP1B1/ICAM1/CAV1/HSP90AA1/CYP1A1/NQO1	14
TP53/IL1B/F2/AKT1/SOD1/PTGS2/EGFR/PPARA/CYP1B1/G6PD/ICAM1/CAV1/BCL2/CDKN1A/HSP90AA1/AKR1C3	16
TP53/AKT1/CCL2/CHEK2/EGFR/PCNA/RB1/MDM2/CCND1/BCL2/CDKN1A/CYP1A1/CDK4/CCNB1/CDK1/E2F1	16
ESR1/AR/MAPK1/IGF2/AKT1/PPARG/IGFBP3/GJA1/CCL2/EGFR/PGR/STAT1/F3/RB1/MYC/ERBB2/CAV1/CCND1/KDR/PPARD/HAS2	21
ESR1/TP53/PPARG/SERPINE1/CASP3/F7/EGFR/PGR/PCNA/NOS2/PPARA/JUN/AHR/CDK4/CDK1/NCOA2/HAS2/NCOA1	18
AR/TP53/IL1B/NOS3/AKT1/SERPINE1/SOD1/MMP9/IL1A/IL4/RB1/HSPB1/ICAM1/CAV1/MDM2/BCL2/BCL2L1/CD44/MCL1	19
TP53/IL1B/IGF2/AKT1/CHEK2/EGFR/PCNA/IL1A/RB1/MDM2/CCND1/CDKN1A/CYP1A1/CDK4/CCNB1/CDK1/PRKACA/E2F1	18
NOS3/MAPK1/AKT1/SOD1/MMP9/MPO/FOS/MMP2/CASP3/EGFR/PCNA/CYP1B1/JUN/BCL2/NQO1/AKR1C3	16
AR/IL1B/NOS3/AKT1/SERPINE1/MMP9/IL1A/IL4/RB1/HSPB1/ICAM1/MDM2/BCL2/BCL2L1/CD44/MCL1	16
TP53/CCL2/CHEK2/PCNA/RB1/MDM2/CCND1/BCL2/CDKN1A/CDK4/CCNB1/CDK1/E2F1	13
TP53/AKT1/CCL2/CHEK2/EGFR/PCNA/RB1/MYC/MDM2/CCND1/BCL2/CDKN1A/CYP1A1/CDK4/CCNB1/CDK1/E2F1	17
AKT1/PPARG/SOD1/FOS/F7/PPARA/G6PD/ICAM1/CCND1/SLC2A4/CDKN1A/NQO1/CD14/AHR/CDK4/AKR1C3	16
TP53/CCL2/CHEK2/PCNA/RB1/MDM2/CCND1/BCL2/CDKN1A/CDK4/CCNB1/CDK1/E2F1	13
NOS3/MAPK1/AKT1/SOD1/MMP9/MPO/FOS/MMP2/EGFR/PCNA/CYP1B1/JUN/NQO1/AKR1C3	14
TP53/AKT1/CCL2/CHEK2/EGFR/PCNA/RB1/MYC/MDM2/CCND1/BCL2/CDKN1A/CYP1A1/CDK4/CCNB1/CDK1/E2F1	17
AR/IL1B/NOS3/AKT1/SERPINE1/CASP3/IL1A/IL4/ICAM1/CAV1/ERBB3/BCL2/IL6R/BCL2L1/MCL1	15
AR/IGF2/AKT1/PPARG/GJA1/CCL2/EGFR/PGR/STAT1/F3/RB1/MYC/ERBB2/CAV1/CCND1/KDR/PPARD/HAS2	18
TP53/AKT1/SOD1/MMP9/CASP3/CHEK2/CYP1B1/HSPB1/CAV1/MDM2/BCL2/CDKN1A/BCL2L1/CD44/MCL1/E2F1	16
TP53/IL1B/IGF2/AKT1/CHEK2/EGFR/PCNA/IL1A/RB1/MDM2/CCND1/CDKN1A/CYP1A1/CDK4/CCNB1/CDK1/PRKACA/E2F1	18
CYP3A4/PCNA/GSTM1/RB1/CYP1B1/PTGS1/CYP2B6/CYP1A1/NR1I2/NQO1/AHR/E2F1	12
ESR1/AR/IL1B/MAPK1/AKT1/SOD1/MMP9/GJA1/FOS/MMP2/PGR/BCL2/CYP1A1/PPARD	14
CYP3A4/PCNA/GSTM1/RB1/CYP1B1/PTGS1/CYP2B6/CYP1A1/NR1I2/NQO1/AHR/E2F1	12
IL1B/NOS3/AKT1/PTGS2/NOS2/CYP1B1/ICAM1/CAV1/HSP90AA1/NQO1	10
TP53/IL1B/F2/AKT1/SOD1/PTGS2/EGFR/ICAM1/CDKN1A/HSP90AA1/AKR1C3	11
AR/IL1B/NOS3/AKT1/SERPINE1/IL1A/IL4/ICAM1/BCL2/BCL2L1/MCL1	11
IL1B/NOS3/AKT1/PTGS2/NOS2/CYP1B1/ICAM1/CAV1/HSP90AA1/NQO1	10

ESR1/AR/IL1B/MAPK1/AKT1/SOD1/MMP9/GJA1/FOS/MMP2/PGR/BCL2/PPARD	13
IL1B/NOS3/AKT1/PTGS2/NOS2/CYP1B1/ICAM1/CAV1/HSP90AA1/NQO1	10
MAPK1/AKT1/PPARG/IGFBP3/MMP9/GJA1/MMP2/EGFR/STAT1/JUN/IL6R/CDKN1A/PPARD/CCNB1	14
TP53/CHEK2/PCNA/MDM2/CCND1/CDKN1A/CDK4/CCNB1/CDK1/PRKACA/E2F1	11
TP53/IL1B/NOS3/IGF2/AKT1/PPARG/SOD1/IGFBP3/PTGS2/NOS2/IL4/ODC1/PPARA/CAV1/PPARD/NQO1/AKR1C3/NCOA2	18
ESR1/IL1B/IGF2/AKT1/CCL2/F7/EGFR/F10/F3/PPARA/ERBB2/ERBB3/KDR/HSP90AA1/AKR1C3	15
TP53/AKT1/CCL2/CHEK2/EGFR/PCNA/RB1/MDM2/CCND1/BCL2/CDKN1A/HSP90AA1/CYP1A1/CDK4/CCNB1/CDK1/PRKACA/E2F1	18
TP53/AKT1/MMP9/MMP2/CASP3/EGFR/PCNA/MMP1/MYC/CCND1/BCL2/CDKN1A	12
MAPK1/AKT1/SOD1/MMP9/FOS/EGFR/JUN/AKR1C3	8
IL1B/AKT1/PPARG/PTGS2/CYP3A4/PLA2G4A/PON1/GSTM1/PPARA/CYP1B1/CAV1/PTGS1/CYP2B6/CYP1A1/PPARD/PTGES/AKR1C3	17
TP53/CXCL8/CHEK2/PCNA/RB1/MYC/MDM2/CCND1/CDKN1A/CDK4/CCNB1/CDK1/PRKACA/E2F1	14
MAPK1/AKT1/SOD1/MMP9/FOS/EGFR/PCNA/JUN/AKR1C	9
AR/IL1B/NOS3/AKT1/SERPINE1/IL1A/IL4/ICAM1/CAV1/BCL2/BCL2L1/MCL1	12
TP53/CHRNA7/AKT1/SOD1/XIAP/CCL2/FOS/CASP3/PPARA/RB1/G6PD/ERBB3/JUN/BCL2/NQO1/MCL1	16
TP53/CHEK2/PCNA/MDM2/CCND1/CDKN1A/CCNB1/CDK1/E2F1	9
TP53/CHEK2/PCNA/MDM2/CCND1/CDKN1A/CCNB1/CDK1/E2F1	9
TP53/CHEK2/PCNA/MDM2/CCND1/CDKN1A/CCNB1/CDK1/E2F1	9
IL1B/PTGS2/PLA2G4A/PON1/GSTM1/CYP1B1/PTGS1/CYP2B6/CYP1A1/PTGES/AKR1C3	11
AKT1/PPARG/IGFBP3/MMP9/GJA1/MMP2/EGFR/STAT1/JUN/IL6R/CDKN1A/PPARD	12
AKT1/MMP9/MMP2/EGFR/MMP1/CCND1	6
AKT1/PPARG/IGFBP3/MMP9/GJA1/MMP2/EGFR/STAT1/JUN/IL6R/CDKN1A/PPARD	12
TP53/AKT1/CCL2/CHEK2/EGFR/PCNA/RB1/MDM2/CCND1/BCL2/CDKN1A/HSP90AA1/CYP1A1/CDK4/CCNB1/CDK1/PRKACA/E2F1	18
TP53/IL1B/MAPK1/AKT1/PPARG/GJA1/FOS/EGFR/PPARA/ICAM1/JUN/BCL2/CDKN1A/AKR1C3/NCOA1	15
IL1B/AKT1/CASP3/IL1A/IL4/ERBB3/BCL2/BCL2L1/MCL1	9
IL1B/AKT1/CASP3/IL1A/IL4/ERBB3/BCL2/BCL2L1/MCL1	9
ESR1/IL1B/PPARG/SERPINE1/SOD1/XIAP/MMP9/PTGS2/EGFR/IL2RA/IL4/PPARA/RB1/TNFAIP6/PPARD/PTGES	16
IL1B/MAPK1/IGF2/CHRNA7/AKT1/SOD1/CASP3/EGFR/ADRB2/RB1/HSPB1/ERBB2/CAV1/CCND1/CDKN1A/CDK4/CCNB1/CDK1	18
TP53/CCL2/CHEK2/EGFR/PCNA/RB1/MDM2/CCND1/BCL2/CDKN1A/BCL2L1/CDK4/CCNB1/CDK1/E2F1	15

TP53/AKT1/MMP9/FOS/MMP2/CASP3/CHEK2/EGFR/PCNA/MMP1/MYC/ICAM1/MDM2/CCND1/JUN/BCL2/CDKN1A	17
ESR1/AR/NR3C2/NR3C1/PPARG/ESR2/XIAP/PGR/PPARA/NR1I2/PPARD/AHR/AKR1C3/NCOA1	14
TP53/IL1B/AKT1/MMP9/GJA1/MMP2/CASP3/CHEK2/EGFR/PCNA/MMP1/MYC/MDM2/SLC2A4/CDKN1A	15
TP53/IL1B/AKT1/MMP9/GJA1/MMP2/CASP3/CHEK2/EGFR/PCNA/MMP1/MYC/MDM2/SLC2A4/CDKN1A	15
IL1B/IGF2/AKT1/CCL2/CASP3/IL2RA/IL1A/IL4/PPARA/ERBB2/ICAM1/CAV1/DPP4/CD44/HAS2	15
IL1B/F2/PPARG/PTGS2/F3/IL1A/IL4/ICAM1/IL6R/PTGES	10
PLAU/F2/NOS3/PPARG/SERPINE1/SOD1/GJA1/CCL2/IL2RA/IL4/PLAT/PPARA/RB1/DPP4/TNFAIP6/PPARD	16
TP53/AKT1/PPARG/PTGS2/MYC/ICAM1/CAV1/MDM2/SLC2A4/BCL2/PPARD/CCNB1/E2F1	13
TP53/IL1B/MAPK1/AKT1/SERPINE1/CXCL8/CCL2/NOS2/IL1A/ICAM1/PPARD/CD14/CDK4	13
ESR1/AR/MAPK1/IGF2/AKT1/PPARG/SOD1/GJA1/CASP3/PGR/ICAM1/CCND1/BCL2/PPARD/AKR1C3/NCOA1	16
ESR1/AR/MAPK1/IGF2/AKT1/PPARG/SOD1/GJA1/CASP3/PGR/ICAM1/CCND1/BCL2/PPARD/AKR1C3/NCOA1	16
TP53/IL1B/IGF2/AKT1/SOD1/CCL2/CASP3/IL2RA/IL1A/IL4/ERBB2/ICAM1/CAV1/BCL2/IL6R/DPP4/CD44	17
TP53/CHEK2/PCNA/MDM2/CDKN1A/CCNB1/CDK1/PRKACA/E2F1	9
IL1B/PTGS2/PLA2G4A/GSTM1/CYP1B1/PTGS1/CYP2B6/CYP1A1/PTGES/AKR1C3	10
TP53/IL1B/MAPK1/AKT1/GJA1/EGFR/PLA2G4A/PON1/ACHE/IL1A/ERBB2/CAV1/BCL2/PPARD/PTGES/PRKACA/E2F1	17
TP53/CHEK2/PCNA/MDM2/CDKN1A/CCNB1/CDK1/E2F1	8
IL1B/MAPK1/AKT1/SERPINE1/CXCL8/CCL2/NOS2/IL1A/ICAM1/PPARD/CD14/CDK4	12
TP53/CHEK2/PCNA/MDM2/CDKN1A/CCNB1/CDK1/E2F1	8
TP53/CHEK2/PCNA/MDM2/CDKN1A/CCNB1/CDK1/E2F1	8
TP53/CHRNA7/AKT1/SOD1/CCL2/FOS/CASP3/PPARA/G6PD/ERBB3/JUN/BCL2/NQO1/MCL1	14
TP53/CCL2/CHEK2/PCNA/RB1/MDM2/CCND1/BCL2/CDKN1A/CDK4/CCNB1/CDK1/E2F1	13
TP53/MAPK1/AKT1/SERPINE1/SOD1/MPO/FOS/CHEK2/ICAM1/JUN/BCL2/CDKN1A/CYP1A1/NQO1	14
PTGS2/CYP3A4/PLA2G4A/GSTM1/CYP1B1/PTGS1/CYP2B6/CYP1A1/PTGES/AKR1C3	10
PTGS2/PLA2G4A/CYP1B1/PTGS1/CYP2B6/CYP1A1/PTGES/AKR1C3	8
TP53/CHEK2/PCNA/MDM2/CDKN1A/CCNB1/CDK1/E2F1	8
TP53/CHEK2/PCNA/MDM2/CDKN1A/CCNB1/CDK1/E2F1	8
TP53/AKT1/SOD1/MMP9/HSPB1/CAV1/MDM2/BCL2/BCL2L1/CD44/MCL1	11

IL1B/IGF2/AKT1/CCL2/CASP3/IL2RA/IL1A/IL4/PPARA/ERBB2/ICAM1/CAV1/DPP4/CD44/HAS2	15
TP53/CHEK2/PCNA/MDM2/CDKN1A/CCNB1/CDK1/E2F1	8
IL1B/MAPK1/AKT1/SERPINE1/CXCL8/CCL2/NOS2/IL1A/ICAM1/PPARD/CD14/CDK4	12
ESR1/ESR2/GJA1/CASP3/F7/EGFR/PCNA/CCND1/NQO1/NCOA1	10
ESR1/AR/MAPK1/IGF2/AKT1/SOD1/GJA1/PPARA/G6PD/BCL2/CCNB1	11
TP53/PPARG/SERPINE1/F7/EGFR/NOS2/PPARA/JUN/AHR/CDK4/CDK1/NCOA2	12
AR/NR3C1/AKT1/PPARG/EGFR/ICAM1/AHR/CDK4/AKR1C3	9
AR/MAPK1/IGF2/AKT1/SOD1/GJA1/PPARA/ADRB2/G6PD/BCL2/CDKN1A/PPARD/CDK4/CCNB1	14
TP53/IL1B/CHEK2/EGFR/KCNH2/NOS2/MYC/NCOA1	8
TP53/CCL2/CHEK2/PCNA/RB1/MDM2/CCND1/BCL2/CDKN1A/CDK4/CCNB1/CDK1/E2F1	13
PLAU/F2/NOS3/SERPINE1/F7/F3/PLAT/CAV1	8
PLAU/F2/NOS3/SERPINE1/F7/F3/PLAT/CAV1	8
TP53/SOD1/CASP3/PLA2G4A/MDM2/HSP90AA1/CYP1A1	7
NOS3/MAPK1/AKT1/PPARG/SOD1/FOS/IL1A/ADRB2/CDKN1A/HSP90AA1/CD14/PRKACA	12
TP53/SOD1/XIAP/CCL2/CASP3/RB1/G6PD/ERBB3/JUN/BCL2/NQO1/MCL1	12
TP53/F2/AKT1/PPARG/ESR2/IGFBP3/GJA1/EGFR/PPARA/RB1/G6PD/ERBB2/BCL2/CDKN1A/HSP90AA1/PPARD	16
TP53/CHEK2/PCNA/MDM2/CCND1/CDKN1A/CCNB1/CDK1/E2F1	9
TP53/F2/AKT1/PPARG/ESR2/IGFBP3/GJA1/EGFR/PPARA/RB1/G6PD/ERBB2/BCL2/CDKN1A/PPARD	15
PLAU/F2/NOS3/SERPINE1/F7/F3/PLAT/CAV1	8
IL1B/NOS3/IGF2/AKT1/PPARG/PTGS2/NOS2/IL4/PPARA/PPARD	10
IL1B/IGF2/AKT1/CCL2/IL2RA/IL1A/IL4/ICAM1/CAV1/DPP4/CD44/HAS2	12
TP53/CHEK2/PCNA/MDM2/CDKN1A/CCNB1/CDK1/E2F1	8
TP53/CHEK2/PCNA/MDM2/CDKN1A/CCNB1/CDK1/E2F1	8
TP53/CHEK2/PCNA/MDM2/CDKN1A/CD44/CCNB1/CDK1/E2F1	9
TP53/CHEK2/PCNA/MDM2/CCND1/CDKN1A/CCNB1/CDK1/E2F1	9
MAPK1/AKT1/SOD1/MMP9/FOS/EGFR/JUN/CYP1A1/NQO1/CCNB1/AKR1C3	11
IL1B/PTGS2/PLA2G4A/GSTM1/PTGS1/PTGES/AKR1C3	7
IL1B/PTGS2/PLA2G4A/GSTM1/PTGS1/PTGES/AKR1C3	7
TP53/CHEK2/PCNA/MDM2/CDKN1A/CCNB1/CDK1/E2F1	8
ESR1/IGF2/AKT1/F7/EGFR/F10/F3/PPARA/ERBB2/ERBB3/HSP90AA1/AKR1C3	12
NR3C1/FOS/CASP3/EGFR/PCNA/ICAM1/CCND1/BCL2/CDKN1A/AKR1C3	10
ESR1/TP53/PPARG/MYC/CDKN1A/CDK4/CCNB1/E2F1	8

TP53/NR3C1/PPARG/FOS/PPARA/JUN/PPARD	7
ESR1/AR/NR3C2/NR3C1/ESR2/EGFR/PGR/PPARA/ICAM1/PPARD/AKR1C3	11
IL1B/IGF2/AKT1/CCL2/CASP3/IL2RA/IL1A/IL4/PPARA/ERBB2/ICAM1/CAV1/DPP4/CD44/HAS2	15
ESR1/TP53/PPARG/MYC/CDKN1A/CDK4/CCNB1/E2F1	8
IL1B/NOS3/AKT1/EGFR/IL1A/CAV1/HSP90AA1	7
TP53/NR3C1/PPARG/FOS/PPARA/JUN/PPARD	7
SERPINE1/CCL2/IL4/PPARA/RB1/ICAM1/KDR/AKR1C3/E2F	9
TP53/AKT1/PTGS2/MYC/ICAM1/MDM2/SLC2A4/BCL2/PPARD/CCNB1/E2F1	11
TP53/CHEK2/PCNA/MDM2/CCND1/CDKN1A/BCL2L1/CCNB1/CDK1/E2F1	10
IL1B/PTGS2/PLA2G4A/PTGS1/PTGES/AKR1C3	6
IL1B/PTGS2/PLA2G4A/PTGS1/PTGES/AKR1C3	6
AKT1/EGFR/RB1/MDM2/CCND1/CYP1A1/CDK4/CCNB1/CDK1	9
TP53/AKT1/MMP9/FOS/MMP2/CASP3/EGFR/PCNA/MMP1/MYC/CCND1/BCL2/CDKN1A	13
CYP3A4/GSTM1/CYP1B1/PTGS1/CYP2B6/CYP1A1/NR1I2/NQO1/AHR	9
IL1B/AKT1/IL1A/BCL2/BCL2L1/MCL1	6
IL1B/AKT1/IL1A/BCL2/BCL2L1/MCL1	6
IL1B/PTGS2/IL1A/ADRB2/PTGES	5
ESR1/AR/MAPK1/AKT1/GJA1/PGR/PPARD	7
TP53/AKT1/PTGS2/MYC/ICAM1/MDM2/SLC2A4/BCL2/PPARD/CCNB1/E2F1	11
MAPK1/AKT1/SOD1/MMP9/FOS/EGFR/JUN/CYP1A1/NQO1/CCNB1/AKR1C3	11
AKT1/CHEK2/CAV1/BCL2/MCL1/E2F1	6
IL1B/NOS3/CHRNA7/PPARG/SERPINE1/CXCL8/STAT1/F3/IL1A/CYP1B1/HSPB1/ERBB2/KDR	13
IL1B/GJA1/PTGS2/ACHE/IL1A/IL4/ADRB2/RB1/CAV1/PTGE	10
IL1B/IGF2/AKT1/CCL2/IL2RA/IL1A/IL4/ICAM1/CAV1/DPP4/CD44/HAS2	12
MAPK1/IGF2/AKT1/PPARG/IGFBP3/MMP9/MMP2/EGFR/ACHE/RUNX2/ADRB2/BCL2/IL6R/PRKACA	14
IL1B/F2/AKT1/PPARG/SOD1/PTGS2/PPARA/CAV1/CYP1A1/PPARD/CDK4/AKR1C3/NCOA2/NCOA1	14
AKT1/EGFR/RB1/CCND1/CYP1A1/CDK4/CCNB1/CDK1	8
MAPK1/SERPINE1/CXCL8/CCL2/F7/F3/IL4/HSPB1/KDR/IL6R/DPP4	11
IL1B/NOS3/CHRNA7/PPARG/SERPINE1/CXCL8/STAT1/F3/IL1A/CYP1B1/HSPB1/ERBB2/KDR	13
IL1B/NOS3/CHRNA7/SERPINE1/CXCL8/F3/IL1A/CYP1B1/HSPB1/KDR	10
ESR1/IGF2/F7/EGFR/F10/F3/ERBB2/ERBB3/HSP90AA1/AKR1C3	10

IL1B/NOS3/CHRNA7/SERPINE1/CXCL8/F3/IL1A/CYP1B1/HSPB1/KDR	10
PLAU/F2/NOS3/MAPK1/SERPINE1/F7/F10/PLA2G4A/F3/PLAT/HSPB1/CAV1/PRKACA	13
TP53/CHEK2/PCNA/MDM2/CDKN1A/CD44/CCNB1/CDK1/E2F1	9
PLAU/F2/NOS3/SERPINE1/GJA1/F7/F3/PLAT/CAV1	9
IL1B/F2/MAPK1/AKT1/PPARG/SOD1/CCL2/EGFR/ERBB2/ERBB3/CDK1/E2F1	12
AR/NOS3/PPARG/SOD1/GJA1/PTGS2/NOS2/PPARA/ADRB2/PTGS1	10
PLAU/F2/NOS3/MAPK1/SERPINE1/F7/F10/PLA2G4A/F3/PLAT/HSPB1/CAV1/PRKACA	13
ESR1/CASP3/EGFR/PGR/PCNA/HAS2/NCOA1	7
PLAU/F2/NOS3/MAPK1/SERPINE1/F7/F10/PLA2G4A/F3/PLAT/HSPB1/CAV1/PRKACA	13
ESR1/AR/MAPK1/IGF2/AKT1/SOD1/EGFR/PGR/PCNA/CAV1/CCND1/BCL2/CYP1A1/NCOA1	14
AKT1/MMP9/HSPB1/MDM2/BCL2/BCL2L1/CD44/MCL1	8
NR3C1/FOS/CASP3/EGFR/PCNA/ICAM1/CCND1/BCL2/CDKN1A	9
AKT1/MMP9/GJA1/MMP2/EGFR/STAT1/JUN/IL6R	8
TP53/MAPK1/PPARG/FOS/PPARA/ICAM1/JUN/BCL2/CDKN1A/AKR1C3/NCOA1	11
IL1B/NOS3/AKT1/EGFR/IL1A/CAV1/HSP90AA1	7
AKT1/PPARG/MMP9/PON1/PPARA/ADRB2/RB1/CAV1/JUN/CDKN1A/SLPI/PRKACA/E2F1	13
IL1B/AKT1/PPARG/PTGS2/ODC1/PPARA/CAV1/PPARD/NQO1/AKR1C3	10
TP53/PPARG/ESR2/GJA1/PPARA/RB1/G6PD/BCL2/CDKN1A/PPARD	10
MAPK1/SERPINE1/CXCL8/F7/F3/IL4/HSPB1/KDR/IL6R	9
IL1B/IGF2/AKT1/CCL2/IL2RA/IL1A/IL4/ERBB2/ICAM1/CAV1/KDR/DPP4/CD44/HAS2	14
TP53/CCL2/CHEK2/PCNA/RB1/MDM2/CCND1/BCL2/CDKN1A/CDK4/CCNB1/CDK1/E2F1	13
TP53/CHEK2/BCL2/CDKN1A/BCL2L1/CD44/MCL1/E2F1	8
IL1B/MAPK1/IGF2/CASP3/IL2RA/IL1A/IL4/ERBB2/BCL2/CDKN1A/AHR	11
TP53/CASP3/CHEK2/MYC/ICAM1/MDM2/CCND1/BCL2/CDKN1A	9
MAPK1/IGF2/AKT1/SOD1/GJA1/PPARA/G6PD/CCNB1	8
TP53/PPARG/ESR2/GJA1/PPARA/ADRB2/RB1/G6PD/BCL2/CDKN1A/PPARD	11
PPARG/F7/EGFR/PCNA/CCND1/BCL2/CDKN1A/PPARD/CDK4/CCNB1	10
ESR1/AR/NR3C2/NR3C1/PPARG/ESR2/PGR/PPARA/PPARD/NCOA1	10
IL1B/AKT1/IL1A/BCL2/BCL2L1/MCL1	6
TP53/CHEK2/PCNA/MDM2/CCND1/CDKN1A/CCNB1/CDK1/E2F1	9

PPARG/CAV1/CDKN1A/CYP1A1/CDK4	5
IL1B/MAPK1/IGF2/CHRNA7/AKT1/SOD1/EGFR/ADRB2/ERBB2/CCND1/CCNB1/CDK1	12
TP53/SOD1/CCL2/CASP3/G6PD/ERBB3/JUN/BCL2/NQO1/MCL1	10
ESR1/AR/MAPK1/PPARG/F7/CAV1/CCND1	7
TP53/IL1B/MAPK1/GJA1/EGFR/ACHE/IL1A/ERBB2/BCL2/PPARD/PRKACA/E2F1	12
TP53/IL1B/MAPK1/IGF2/CASP3/IL2RA/IL1A/IL4/ERBB2/BCL2/CDKN1A/AHR	12
AKT1/EGFR/RB1/CCND1/CYP1A1/CDK4/CCNB1/CDK1	8
TP53/MAPK1/PPARG/GJA1/PCNA/MYC/CDKN1A/HSP90AA1	8
IL1B/AKT1/PPARG/PTGS2/ODC1/PPARA/CAV1/CYP2B6/PPARD/NQO1/AKR1C3	11
AKT1/SOD1/CYP1B1/HSPB1/BCL2/MCL1	6
PPARG/F7/EGFR/PCNA/CCND1/CDKN1A/CDK4	7
AR/IGF2/AKT1/EGFR/F3/MYC/ERBB2/CCND1/KDR/HAS2	10
MAPK1/AKT1/SERPINE1/CXCL8/CCL2/F7/IL4/ICAM1/IL6R/DPP4	10
TP53/CHEK2/MDM2/BCL2/CDKN1A/CD44/E2F1	7
TP53/CHEK2/PCNA/MDM2/CCND1/CDKN1A/CCNB1/CDK1/E2F1	9
TP53/AKT1/CHEK2/PCNA/MDM2/BCL2/CDKN1A/CD44/CCNB1/CDK1/E2F1	11
TP53/IL1B/IGF2/AKT1/PPARG/GJA1/ADRB2/ICAM1/CAV1/MDM2/SLC2A4/CDK4/PRKACA	13
IL1B/F2/PTGS2/IL1A/IL6R/PTGES	6
ESR1/MYC/CDKN1A/CDK4/CCNB1/E2F1	6
IL1B/PTGS2/IL1A/PTGES	4
IL1B/IGF2/AKT1/SOD1/CCL2/CASP3/IL2RA/IL1A/IL4/ERBB2/CAV1/DPP4	12
TP53/CHEK2/PCNA/MDM2/CCND1/CDKN1A/BCL2L1/CCNB1/CDK1/E2F1	10
TP53/IL1B/MAPK1/GJA1/EGFR/ACHE/IL1A/ERBB2/BCL2/PPARD/PRKACA/E2F1	12
TP53/MAPK1/PPARG/PPARA/ICAM1/JUN/BCL2/CDKN1A/AKR1C3/NCOA1	10
ESR1/SOD1/CYP3A4/PON1/IL4/CYP1B1/G6PD/CYP2B6/CYP1A1/NR1I2/PPARD/AKR1C3	12
NOS3/SOD1/MPO/PTGS2/GSTM1/PTGS1/NQO1/PTGES	8
ESR1/AR/SOD1/CASP3/PGR/ICAM1/CCND1/BCL2/AKR1C3/NCOA1	10
NOS3/MAPK1/AKT1/SOD1/IL1A/CDKN1A/HSP90AA1/CD14/PRKACA	9
PLAU/F2/NOS3/SERPINE1/GJA1/F7/F3/PLAT/CAV1	9
MAPK1/SERPINE1/CXCL8/CCL2/F7/IL4/IL6R/DPP4	8
PTGS2/PLA2G4A/CYP1B1/PTGS1/CYP2B6/CYP1A1/PTGES/AKR1C3	8

ESR1/AR/SOD1/CASP3/PGR/ICAM1/CCND1/BCL2/AKR1C3/NCOA1	10
PPARG/F7/EGFR/CCND1/CYP1A1/PPARD/NQO1/AKR1C3/NCOA1	9
PTGS2/PTGS1/PTGES/AKR1C3	4
PPARG/MMP2/PGR/RB1/G6PD/BCL2/CDKN1A/CCNB1/PDE3A/PRKACA	10
PPARG/PGR/RB1/G6PD/BCL2/CDKN1A/CCNB1/PDE3A/PRKACA	9
IL1B/SOD1/MMP9/GJA1/MMP2/PPARD	6
IL1B/PTGS2/CYP3A4/PLA2G4A/GSTM1/PTGS1/CYP1A1/PTGES/AKR1C3	9
IL1B/IGF2/CASP3/IL2RA/IL1A/IL4/ERBB2/BCL2/CDKN1A/AHR	10
TP53/CHEK2/EGFR/PCNA/MYC/MDM2/BCL2/BCL2L1/CD44/MCL1	10
NOS3/SOD1/MPO/PTGS2/GSTM1/PTGS1/NQO1/PTGES	8
IL1B/PTGS2/PLA2G4A/PTGS1/PTGES/AKR1C3	6
CHRNA7/MMP9/GJA1/MMP2/ADRB2/ICAM1	6
IL1B/IGF2/CASP3/IL2RA/IL1A/IL4/ERBB2/BCL2/CDKN1A/AHR	10
TP53/IL1B/IGF2/CASP3/IL2RA/IL1A/IL4/ERBB2/BCL2/CDKN1A/AHR	11
TP53/IL1B/IGF2/CASP3/IL2RA/IL1A/IL4/ERBB2/BCL2/CDKN1A/AHR	11
G6PD/CCND1/BCL2/CYP1A1/CCNB1	5
SOD1/PPARA/G6PD/ICAM1/CCND1/SLC2A4/NQO1/CD14	8
TP53/CHEK2/MYC/MDM2/BCL2/CDKN1A	6
NOS3/AKT1/SOD1/GJA1/EGFR/ADRB2/ICAM1/CAV1	8
NOS3/AKT1/SOD1/GJA1/EGFR/ADRB2/ICAM1/CAV1	8
NOS3/AKT1/SOD1/GJA1/EGFR/ADRB2/ICAM1/CAV1	8
PPARG/CAV1/CDKN1A/CYP1A1/CDK4	5
NOS3/AKT1/PPARG/MMP9/PTGS2/IL4/CYP1B1/HSPB1/KDR/DPP4/PPARD/HAS2	12
IGF2/AKT1/PPARG/GJA1/CCL2/STAT1/F3/CAV1/KDR	9
PLAU/SERPINE1/IGFBP3/PLAT/BCL2/PPARD/HAS2	7
TP53/MMP9/MMP2/PCNA/MMP1/MYC/CDKN1A	7
TP53/AKT1/MMP9/JUN/BCL2/BCL2L1	6
NOS3/AKT1/PPARG/MMP9/PTGS2/IL4/CYP1B1/HSPB1/KDR/DPP4/PPARD/HAS2	12
IL1B/PTGS2/PLA2G4A/PTGS1/PTGES/AKR1C3	6
IL1B/IGF2/AKT1/PPARG/GJA1/F7/STAT1/PPARA/ICAM1/CAV1/SLC2A4/CDK4/PRKACA	13
ESR1/AR/MAPK1/AKT1/PGR/CAV1/CCND1/NCOA1	8
NOS3/AKT1/PPARG/MMP9/PTGS2/IL4/CYP1B1/HSPB1/KDR/DPP4/PPARD/HAS2	12
IL1B/SERPINE1/SOD1/PTGS2/STAT1/F3/IL1A/IL4/CYP1B1/HSPB1/IL6R/HSP90AA1/CD14	13
ESR1/AR/NR3C2/NR3C1/ESR2/PGR/PPARA/PPARD	8
IGF2/CXCL8/EGFR/RB1/BCL2/CDKN1A/CYP1A1/CCNB1	8

AKT1/GJA1/EGFR/ICAM1/CAV1	5
MAPK1/IGF2/AKT1/PPARG/SOD1/GJA1/PPARD/NCOA1	8
SERPINE1/CCL2/IL4/ICAM1/KDR/AKR1C3	6
ESR1/AR/IL1B/MAPK1/AKT1/PPARG/ESR2/FOS/RB1/CYP1B1/ICAM1/CAV1/JUN	13
SERPINE1/CCL2/IL4/PPARA/ICAM1/KDR/AKR1C3	7
IL1B/AKT1/PTGS2/ICAM1/CAV1/HSP90AA1	6
NOS3/SOD1/MPO/PTGS2/GSTM1/PTGS1/NQO1/PTGES	8
NOS3/AKT1/SOD1/GJA1/EGFR/ADRB2/ICAM1/CAV1/SLC2A4/PDE3A	10
IL1B/PLA2G4A/NOS2/PTGES	4
TP53/CCL2/CASP3/RB1	4
IL1B/AKT1/PPARG/PLA2G4A/PON1/PPARA/CAV1/PPARD/PTGES	9
TP53/SOD1/CAV1/BCL2/BCL2L1/MCL1	6
MAPK1/AKT1/CHEK2/EGFR/CAV1/BCL2/HSP90AA1/CD44/CCNB1/CDK1/PRKACA	11
IGF2/AKT1/PPARG/GJA1/CCL2/STAT1/F3/CAV1/KDR	9
TP53/MMP9/MMP2/CHEK2/PCNA/MMP1/MYC/MDM2/CDKN1A	9
IL1B/AKT1/PTGS2/ICAM1/CAV1/HSP90AA1	6
IL1B/AKT1/PPARG/PTGS2/PPARA/CAV1/PPARD	7
AKT1/EGFR/CCND1/CDKN1A/CCNB1	5
IL1B/AKT1/PTGS2/PPARA/ICAM1/CAV1/HSP90AA1	7
IL1B/AKT1/PPARG/GJA1/PLA2G4A/NOS2/PON1/PPARA/CAV1/PPARD/PTGES/NCOA2/NCOA1	13
ESR1/AR/MAPK1/AKT1/PGR/CCND1	6
TP53/MAPK1/AKT1/CXCL8/CCL2/CASP3/STAT1/ICAM1/SLC2A4/CD14/HAS2	11
NOS3/MAPK1/SLC6A2/SOD1/GJA1/EGFR/PCNA/ADRB2/RB1/MYC/HSPB1/BCL2/HSP90AA1	13
AKT1/CASP3/EGFR/CCND1/CDKN1A/CDK4/CCNB1	7
PLAU/SERPINE1/IGFBP3/PLAT/BCL2/PPARD/HAS2	7
SERPINE1/CCL2/IL4/ICAM1/KDR/AKR1C3	6
SERPINE1/MMP9/MMP2/MMP1/RB1/CYP1B1/ICAM1/CAV1/KDR/DPP4/CD44/HAS2	12
NOS3/SOD1/MPO/PTGS2/PTGS1/NQO1/PTGES	7
SERPINE1/MMP9/MMP2/MMP1/RB1/CYP1B1/ICAM1/CAV1/KDR/DPP4/CD44/HAS2	12
IL1B/MAPK1/IGF2/IL2RA/IL1A/IL4/BCL2/CDKN1A	8
SERPINE1/MMP9/MMP2/MMP1/RB1/CYP1B1/ICAM1/CAV1/KDR/DPP4/CD44/HAS2	12
IL1B/AKT1/PPARG/GJA1/MPO/FOS/EGFR/JUN/CCNB1	9

NOS3/AKT1/PPARG/MMP9/GJA1/PTGS2/IL4/CYP1B1/HSPB1/KDR/DPP4/PPARD/HAS2	13
ESR1/AR/SOD1/CASP3/PGR/ICAM1/CCND1/BCL2/AKR1C3/NCOA1	10
IL1B/MAPK1/CHRNA7/AKT1/SOD1/EGFR/IL4/ADRB2/IL6R/CDK1/PRKACA	11
IL1B/AKT1/PPARG/GJA1/PLA2G4A/PPARA/PPARD/PTGES	8
IL1B/IGF2/AKT1/CCL2/PLA2G4A/IL2RA/IL1A/IL4/CAV1/BCL2/CDKN1A/DPP4	12
AKT1/PPARG/SERPINE1/IGFBP3/GJA1/CCL2/IL4/CYP1B1/BCL2/DPP4/PPARD	11
AKT1/CASP3/EGFR/CCND1/CDKN1A/CDK4/CCNB1	7
MAPK1/AKT1/CHEK2/EGFR/CAV1/BCL2/HSP90AA1/CD44/CCNB1/CDK1/PRKACA	11
PPARG/MMP2/PGR/RB1/G6PD/BCL2/CDKN1A/CCNB1/PDE3A/PRKACA	10
F2/MAPK1/AKT1/EGFR/ERBB2/ERBB3/KDR/PPARD	8
AKT1/MPO/PPARA/G6PD/CYP1A1	5
IL1B/AKT1/PTGS2/ICAM1/HSP90AA1	5
IL1B/PPARG/PTGS2/PPARA/PPARD	5
IL1B/PLA2G4A/NOS2/PTGES	4
TP53/SERPINE1/CHEK2/CDKN1A	4
F2/MAPK1/AKT1/PPARG/SOD1/EGFR/ERBB2/ERBB3/CDK1	9
IL1B/AKT1/PPARG/PLA2G4A/PON1/PPARA/CAV1/PTGES	8
TP53/IL1B/PPARG/SOD1/IL2RA/IL1A/IL4/MYC/ERBB2/JUN/BCL2/IL6R	12
F2/PPARG/MMP9/MMP2/MMP1/CTSD/PPARD	7
IL1B/IGF2/AKT1/CCL2/PLA2G4A/IL2RA/IL1A/IL4/CAV1/BCL2/CDKN1A/DPP4	12
IL1B/AKT1/PTGS2/ICAM1/HSP90AA1	5
IL1B/IGF2/AKT1/CCL2/IL2RA/IL1A/IL4/CAV1/DPP4	9
NOS3/SOD1/MPO/EGFR/NOS2/NQO1	6
IL1B/NOS3/AKT1/EGFR/IL1A/CAV1/HSP90AA1	7
AKT1/PPARA/ADRB2/CAV1/JUN/CDKN1A/SLPI/E2F1	8
TP53/NR3C1/PPARG/FOS/JUN	5
TP53/IGF2/AKT1/NOS2/IL4/PPARA/CCNB1/CDK1	8
AR/PPARG/GJA1/CCL2/STAT1/RB1/CAV1/PPARD	8
PPARG/SOD1/IL2RA/IL4/PPARA/RB1/TNFAIP6/PPARD	8
AKT1/PPARG/SERPINE1/IGFBP3/GJA1/CCL2/IL4/CYP1B1/BCL2/DPP4/PPARD	11
TP53/IL1B/SOD1/IL1A/ERBB2/BCL2	6
MAPK1/IGF2/GJA1/FOS/PPARA/RB1/G6PD/CAV1/ERBB3/BCL2/CCNB1	11
TP53/SERPINE1/SOD1/CHEK2/ICAM1/BCL2/CDKN1A	7

AKT1/PPARG/SERPINE1/IGFBP3/GJA1/CCL2/IL4/CYP1B1/BCL2/DPP4/PPARD	11
PLAU/F2/NOS3/SERPINE1/GJA1/PLAT	6
TP53/MAPK1/AKT1/PPARG/GJA1/EGFR/PCNA/IL4/MYC/CDKN1A/HSP90AA1	11
IL1B/IGF2/AKT1/CCL2/IL2RA/IL1A/IL4/CAV1/BCL2/CDKN1A/DPP4	11
IL1B/PTGS2/CYP3A4/PLA2G4A/GSTM1/PTGS1/CYP1A1/PTGES/AKR1C3	9
TP53/AKT1/MMP9/JUN/BCL2/BCL2L1/E2F1	7
MAPK1/CHRNA7/CASP3/PPARA/BCL2	5
TP53/CHEK2/BCL2/CDKN1A/CD44	5
TP53/IGF2/EGFR/IL4/ERBB2/ICAM1/CAV1/ERBB3/KDR/IL6R/CD44	11
TP53/NOS3/GJA1/MMP2/EGFR/IL1A/ADRB2/CAV1	8
MAPK1/IGF2/GJA1/FOS/PPARA/RB1/G6PD/CAV1/ERBB3/BCL2/CCNB1	11
TP53/IGF2/EGFR/IL4/ERBB2/ICAM1/CAV1/ERBB3/KDR/IL6R/CD44	11
PLAU/SERPINE1/IGFBP3/BCL2/PPARD/HAS2	6
CXCL8/EGFR/RB1/BCL2/CDKN1A/CYP1A1/CCNB1	7
TP53/MAPK1/PPARG/MYC/HSP90AA1	5
NOS3/NOS2/IL4/PPARA	4
NOS3/NOS2/IL4/PPARA	4
AKT1/PPARG/SERPINE1/IGFBP3/GJA1/CCL2/IL4/CYP1B1/BCL2/DPP4/PPARD	11
PPARG/MMP9/GJA1/MMP2/JUN/CDKN1A	6
PPARG/MMP9/GJA1/MMP2/JUN/CDKN1A	6
MAPK1/SERPINE1/CXCL8/CCL2/F7/IL4/HSPB1/KDR/IL6R/DPP4	10
IL1B/PPARG/PTGS2/IL4/PTGES	5
PLAU/F2/NOS3/SERPINE1/PLAT	5
IL1B/AKT1/PLA2G4A/PPARA/PTGES	5
IL1B/AKT1/GJA1/CHEK2/EGFR/NOS2/ODC1/CAV1/MDM2/HSP90AA1/PRKACA	11
AR/TP53/IGF2/SOD1/ADRB2/BCL2/CDK4	7
PLAU/F2/NOS3/SERPINE1/PLAT	5
NOS3/SOD1/MPO/NQO1	4
F2/SERPINE1/F7/F3	4
CHEK2/MDM2/CCND1/CDK4	4
F2/SERPINE1/F7/F3	4
TP53/IL1B/SOD1/IL2RA/IL1A/IL4/ERBB2/BCL2/IL6R	9
F2/GJA1/CCL2/KCNH2/G6PD/ICAM1/CAV1/BCL2/PRKACA	9
TP53/GJA1/CAV1/BCL2/NQO1	5

SERPINE1/IL4/PPARA/ICAM1/KDR	5
MAPK1/SERPINE1/CXCL8/F7/IL4/ICAM1/IL6R	7
NOS3/AKT1/SOD1/CASP3/STAT1/IL2RA/RB1/G6PD/BCL2	9
ESR1/AR/ICAM1/CCND1/BCL2/AKR1C3/NCOA1	7
TP53/MMP9/MMP2/PCNA/MMP1/MYC/CDKN1A	7
F2/SERPINE1/F7/F3	4
CHEK2/CAV1/BCL2/MCL1	4
SOD1/CASP3/PCNA/CYP1B1/JUN/BCL2/NQO1	7
ESR1/AR/ICAM1/CCND1/BCL2/AKR1C3/NCOA1	7
IL1B/IGF2/IL2RA/IL1A/IL4/BCL2/CDKN1A	7
IL1B/MAPK1/CHRNA7/CCL2/EGFR/MYC/ERBB2/ICAM1/KDR/CD44	10
PPARG/F7/EGFR/CCND1/CYP1A1/PPARD	6
AKT1/SOD1/CYP1B1/HSPB1/BCL2/MCL1	6
IL1B/IGF2/IL2RA/IL1A/IL4/BCL2/CDKN1A	7
F2/MAPK1/IGF2/AKT1/EGFR/ERBB2/BCL2/PPARD/CCNB1	9
PLAU/F2/NOS3/SERPINE1/PLAT	5
PLAU/F2/NOS3/SERPINE1/GJA1/PLAT	6
F2/MAPK1/AKT1/EGFR/ERBB2/ERBB3/KDR/PPARD	8
IL1B/AKT1/PPARG/GJA1/PLA2G4A/PPARA/PPARD/PTGES/NCOA2/NCOA1	10
PLAU/F2/SERPINE1/PLAT	4
CXCL8/F7/F3/KDR	4
NOS3/SOD1/MPO/NQO1	4
NOS3/SOD1/MPO/NQO1	4
MAPK1/SERPINE1/CXCL8/F7/IL4/IL6R	6
TP53/MAPK1/PPARG/GJA1/PCNA/MYC/CDKN1A/HSP90AA1	8
AR/TP53/AKT1/EGFR/ERBB2/BCL2/BCL2L1/E2F1	8
TP53/IL1B/IGF2/CASP3/IL2RA/IL1A/IL4/ERBB2	8
TP53/FOS/CASP3/JUN/NQO1/MCL1	6
TP53/AKT1/CASP3/IL4/ADRB2/HSPB1/KDR/BCL2/MCL1/PRKACA	10
TP53/MAPK1/PPARG/PPARA/JUN/BCL2/CDKN1A/AKR1C3	8
AKT1/MMP9/PPARA/ADRB2/CAV1/CDKN1A/SLPI/PRKACA	8
F2/MAPK1/AKT1/EGFR/ERBB2/ERBB3/KDR/PPARD	8
TP53/CASP3/JUN/NQO1/MCL1	5
ESR1/SOD1/CASP3/PGR/ICAM1/BCL2	6
CXCL8/F7/F3/KDR	4
TP53/IGF2/EGFR/IL4/ICAM1/CAV1/ERBB3/IL6R/CD44	9

IL1B/AKT1/PTGS2/ICAM1/HSP90AA1	5
PPARG/SOD1/FOS/STAT1/JUN/AHR/PDE3A	7
CCND1/CDK4/CCNB1/CDK1	4
AKT1/PPARA/ADRB2/CAV1/CDKN1A/SLPI	6
MAPK1/AKT1/MMP9/EGFR/ERBB2/ERBB3/HSP90AA1	7
IL1B/AKT1/CASP3/RB1/HSPB1/CAV1/CDKN1A	7
ESR1/SOD1/CASP3/PGR/ICAM1/BCL2	6
IGF2/AKT1/CASP3/IL4/PPARA/RB1/G6PD/BCL2/CCNB1	9
AKT1/PPARG/XIAP/MMP9/F3/MYC/CTSD/CD44	8
NOS3/SOD1/MPO/NQO1	4
MAPK1/AKT1/GJA1/PPARD	4
IL1B/PTGS2/IL1A/CYP1B1	4
TP53/CHEK2/MDM2/CDKN1A	4
F2/F7/F10/F3	4
F2/F7/F10/F3	4
AKT1/SOD1/HSPB1/MCL1	4
TP53/MDM2/BCL2/CD44	4
IL1B/MAPK1/AKT1/CCL2/CD14	5
IL1B/GJA1/EGFR/PLA2G4A/ACHE/IL1A/IL4/PPARD/PTGES	9
IL1B/F2/AKT1/PPARG/PTGS2/PPARA/PPARD	7
TP53/AKT1/MMP9/KDR/BCL2/BCL2L1/E2F1	7
NOS3/SOD1/MPO/NQO1	4
CXCL8/CCL2/EGFR/PPARA/ICAM1/CAV1/BCL2/DPP4/SLPI/CDK1	10
TP53/IGF2/AKT1/IGFBP3/PPARA/G6PD/PPARD/NCOA2	8
TP53/CHEK2/CCND1/CDKN1A/HSP90AA1/CDK4/CCNB1/CDK1/PRKACA	9
IL1B/AKT1/PLA2G4A/PPARA/PTGES	5
IGF2/SOD1/ADRB2/BCL2/CDK4	5
PPARG/PPARA/PPARD	3
IL1B/IL1A/ERBB2	3
MMP2/CASP3/F7/PCNA/ICAM1/NQO1	6

ESR1/AR/ICAM1/CCND1/BCL2/AKR1C3/NCOA1	7
AKT1/GJA1/EGFR/ICAM1/CAV1	5
CASP3/IL4/BCL2/CDKN1A/AHR	5
PON1/CDK4/CCNB1/AKR1C3/E2F1	5
CHRNA7/AKT1/SOD1/GJA1/KCNH2/ADRB2/CAV1/JUN/KDR/BCL2/BCL2L1	11
AKT1/EGFR/CCND1/CCNB1	4
CCND1/CDK4/CCNB1/CDK1	4
IL1B/PTGS2/IL1A/CYP1B1/IL6R	5
PPARG/MMP9/GJA1/CCL2/PON1/ADRB2/CAV1/BCL2/PRKACA	9
TP53/MAPK1/PPARA/JUN/BCL2/CDKN1A/AKR1C3	7
TP53/PPARA/G6PD/CAV1/BCL2	5
NOS3/AKT1/GJA1/HAS2	4
TP53/CCND1/CDKN1A/HSP90AA1/CDK4/CCNB1/CDK1/PRKACA	8
AKT1/EGFR/CAV1/BCL2/HSP90AA1/CD44	6
TP53/AKT1/CASP3/IL4/ADRB2/KDR/PRKACA	7
AKT1/PPARG/PTGS2/ADRB2/CCND1/SLC2A4/PPARD/E2F1	8
IL1B/PLA2G4A/PTGES	3
IL1B/PLA2G4A/PTGES	3
IL1B/IL1A/ERBB2	3
MMP9/MMP2/MMP1	3
TP53/CASP3/CCND1/CDKN1A	4
EGFR/ERBB2/ERBB3/HSP90AA1	4
IGF2/AKT1/PPARG/IGFBP3/ACHE/RUNX2/IL6R/PRKACA	8
NOS3/AKT1/PPARG/MMP9/PTGS2/IL4/HSPB1/KDR/HAS2	9
PTGS2/CYP3A4/GSTM1/CYP1A1	4
AKT1/PPARG/PPARA/PPARD	4
MAPK1/SERPINE1/CXCL8/CCL2/F7/IL4/IL6R/DPP4	8
AKT1/SERPINE1/CASP3/IL2RA/IL4/PPARA/CYP1B1/ERBB2/ERBB3	9
IL1B/GJA1/EGFR/PLA2G4A/ACHE/IL1A/IL4/PPARD/PTGES	9
TP53/F2/MAPK1/AKT1/EGFR/ERBB2/BCL2/CDK1/E2F1	9
ESR1/SOD1/CASP3/PGR/ICAM1/BCL2	6
TP53/IL1B/AKT1/PPARG/CASP3/RB1/HSPB1/CAV1/CDKN1A	9
TP53/MAPK1/AKT1/CXCL8/CCL2/STAT1/ICAM1/SLC2A4/HAS2	9
SOD1/CYP3A4/PON1/IL4/CYP1B1/G6PD/PPARD	7
SOD1/CYP3A4/PLA2G4A/PON1/IL4/CYP1B1/G6PD/CYP1A1/PPARD/AKR1C3	10
F2/CCL2/STAT1/IL4/CYP1B1/CAV1/IL6R	7
AKT1/PPARG/SERPINE1/XIAP/MMP9/F3/MYC/CAV1/CTSD/SLPI/CD44	11

ESR1/AR/NR3C2/NR3C1/ESR2/PGR	6
SERPINE1/IL4/ICAM1/KDR	4
AKT1/PPARG/XIAP/MMP9/F3/MYC/CTSD/CD44	8
PCNA/HAS2/NCOA1	3
IL1B/IGF2/CASP3/IL2RA/IL1A/IL4/ERBB2	7
CYP3A4/EGFR/CYP1B1/CYP1A1/PPARD/AKR1C3	6
MAPK1/GJA1/PPARA/G6PD/CCNB1	5
PPARG/SOD1/IL2RA/IL4/PPARA/RB1/TNFAIP6/PPARD	8
NOS3/CXCL8/EGFR/NOS2/ERBB3/KDR/AHR/PDE3A/PRKACA	9
AKT1/SOD1/KDR/BCL2/BCL2L1	5
AKT1/SOD1/ERBB3/CDK1	4
AKT1/CASP3/IL2RA/BCL2	4
F2/GJA1/CCL2/G6PD/ICAM1/CAV1/BCL2/PRKACA	8
IL1B/GJA1/CAV1	3
IL1B/PTGS2/PTGES	3
IL1B/CD44/HAS2	3
TP53/PLA2G4A/MDM2	3
PLAU/SERPINE1/CYP1B1/ICAM1/DPP4	5
NOS3/AKT1/MMP9/PTGS2/HSPB1/KDR/HAS2	7
F2/CCL2/STAT1/IL4/CYP1B1/CAV1/IL6R	7
MMP2/CASP3/F7/PCNA/ICAM1/NQO1	6
TP53/IGF2/AKT1/IGFBP3/PPARA/NCOA2	6
TP53/IL1B/AKT1/EGFR/NOS2/PPARA/BCL2/MCL1/E2F1	9
TP53/PPARG/CCL2/STAT1/NOS2/ICAM1/CD44	7
IL1B/GJA1/PLA2G4A/NOS2/PTGES/NCOA2/NCOA1	7
AKT1/SOD1/ERBB2/ERBB3/CDK1	5
TP53/IL1B/MAPK1/ERBB2/BCL2/PRKACA/E2F1	7
CDK4/CCNB1/AKR1C3/E2F1	4
NR3C1/EGFR/PCNA/ICAM1	4
PPARG/GJA1/CCL2/STAT1/CAV1	5
NOS3/AKT1/PTGS2/HSPB1/KDR	5
IL1B/F2/AKT1/PPARG/GJA1/PPARA/CAV1/PPARD/PRKACA	9
CYP3A4/EGFR/CYP1B1/CYP1A1/PPARD/AKR1C3	6
IL1B/AKT1/CD44/HAS2	4
TP53/PPARG/GJA1/CDKN1A	4
TP53/EGFR/BCL2/HSP90AA1/E2F1	5
MAPK1/GJA1/PPARA/G6PD/CCNB1	5
TP53/IGF2/AKT1/IGFBP3/PPARA/G6PD/PPARD/NCOA2	8

TP53/SOD1/CAV1/BCL2/BCL2L1/MCL1	6
TP53/NR3C1/FOS/IL4/PPARA/CAV1/JUN/PDE3A	8
NOS3/NOS2/PPARA/ADRB2	4
ESR1/AR/PGR/CAV1	4
IL1B/MMP9/PTGS2/IL4	4
F2/MAPK1/EGFR/ERBB3/KDR/PPARD	6
TP53/AKT1/EGFR/ERBB2/BCL2/E2F1	6
IL1B/AKT1/IGFBP3/CASP3/PPARA/RB1/HSPB1/CAV1/CDKN1A/CCNB1	10
IL1B/PTGS2/CYP3A4/PLA2G4A/GSTM1/PTGS1/CYP1A1/PTGES/AKR1C3	9
AKT1/GJA1/EGFR/ICAM1/CAV1	5
PPARG/PPARA/PPARD	3
PLAU/PPARG/SERPINE1/ESR2/CCL2/PPARA/ADRB2	7
NOS3/SERPINE1/ICAM1/BCL2L1	4
SOD1/FOS/STAT1/JUN/AHR/PDE3A	6
TP53/NR3C1/FOS/IL4/PPARA/CAV1/JUN/PDE3A	8
PLAU/F2/MAPK1/MMP9/MPO/CTSD/HSP90AA1/SLPI/TNFAIP6/CD44/CD14	11
MAPK1/AKT1/SERPINE1/CCL2/IL4/ICAM1/IL6R	7
PLAU/MAPK1/CXCL8/MMP9/MPO/CTSD/HSP90AA1/SLPI/TNFAIP6/CD44/CD14	11
CHEK2/CCND1/CDKN1A/HSP90AA1/CDK4/CCNB1/CDK1/PRKACA	8
MMP9/MMP2/MMP1/DPP4/CD44	5
IL1B/GJA1/PLA2G4A/NOS2/PTGES	5
SOD1/IL1A/ICAM1/CYP1A1	4
NOS3/NOS2/IL4/PPARA	4
IL1B/PTGS2/CYP3A4/PLA2G4A/GSTM1/PTGS1/CYP1A1/PTGES/AKR1C3	9
NOS3/SOD1/GJA1/EGFR/ADRB2/RB1/HSPB1/BCL2	8
CHEK2/MDM2/BCL2/BCL2L1/CD44	5
TP53/IGF2/IL4/ICAM1/ERBB3/IL6R/CD44	7
PPARG/MMP9/FOS/CASP3/STAT1/IL4/RB1/MYC/G6PD/JU	10
NOS3/NOS2/IL4/PPARA	4
IL1B/PLA2G4A/NOS2/PTGES	4
NOS3/MPO/NOS2/NQO1	4
IL1B/IGF2/IL1A/RB1	4
IL1B/AKT1/CYP3A4/PLA2G4A/PPARA/CYP1B1/PPARD/CDK4/AKR1C3	9
IL1B/IGF2/IL1A/RB1/CCNB1/PDE3A	6

NOS3/SERPINE1/ICAM1/BCL2/BCL2L1	5
TP53/MMP9/KDR/BCL2/E2F1	5
ESR1/AR/IL1B/AKT1/PPARG/ESR2/ICAM1/CAV1	8
F2/IL4/CYP1B1/IL6R	4
TP53/AKT1/MMP9/BCL2L1	4
IL1B/MMP9/PTGS2/IL4	4
TP53/PPARG/CCL2/STAT1/NOS2/ICAM1/CD44	7
AKT1/PPARG/SERPINE1/XIAP/MMP9/F3/MYC/CTSD/SLPI/CD44	10
MAPK1/AKT1/EGFR/PCNA/IL4/MYC/HSP90AA1	7
IL1B/PPARG/PPARA/CAV1/PPARD	5
IL1B/PLA2G4A/PON1/CAV1/PTGES	5
TP53/F2/AKT1/SERPINE1/XIAP/MMP9/PLAT/SLPI/CD44	9
TP53/CHEK2/CDKN1A	3
IL1B/PPARG/PPARA	3
IL1B/PLA2G4A/PTGES	3
ESR1/AR/CCND1	3
ESR1/AR/CCND1	3
CAV1/BCL2/MCL1	3
AKT1/CASP3/IL2RA/IL4/PPARA/ERBB2	6
CYP3A4/CYP2B6/CYP1A1/NR1I2	4
FOS/CAV1/NCOA2/NCOA1	4
AKT1/EGFR/CCND1/CYP1A1	4
KCNH2/CAV1/JUN/KDR/BCL2	5
TP53/IGF2/AKT1/IGFBP3/PPARA/G6PD/PPARD/NCOA2	8
NOS3/NOS2/IL4/PPARA	4
MAPK1/AKT1/EGFR/ERBB2	4
NOS3/NOS2/IL4/PPARA	4
CCND1/CDKN1A/HSP90AA1/CDK4/CCNB1/CDK1/PRKACA	7
IL1B/AKT1/SOD1/PTGS2/PPARA/CDK4/AKR1C3	7
PPARG/MMP9/FOS/IL4/RB1/MYC/JUN	7
AKT1/PPARG/AHR/CDK4/AKR1C3	5
PPARG/PPARA/PPARD	3

PPARG/CYP1A1/PPARD	3
TP53/CHEK2/CDKN1A	3
MMP9/PPARD/HAS2	3
PPARG/FOS/ADRB2/HSP90AA1	4
MMP9/MMP2/MMP1/CTSD	4
MMP9/STAT1/CAV1/CCND1	4
CYP3A4/CYP1B1/CYP1A1/AKR1C3	4
MMP9/GJA1/MMP2/JUN	4
CYP3A4/EGFR/CYP1B1/CYP1A1/PPARD/AKR1C3	6
AKT1/EGFR/CAV1/BCL2/HSP90AA1/CD44	6
ESR1/AR/PGR/MYC/KDR/BCL2	6
TP53/IGF2/AKT1/IGFBP3/PPARA/NCOA2/HAS2	7
TP53/IGF2/AKT1/NOS2/IL4/MYC/CCNB1/CDK1	8
IL1B/IGF2/AKT1/PPARG/PPARA/ICAM1/SLC2A4/CDK4	8
NOS3/AKT1/PPARG/PTGS2/CYP1B1/HSPB1/KDR/DPP4	8
ESR1/AR/IL1B/SOD1/PGR/ICAM1/CDKN1A	7
TP53/IGF2/AKT1/IGFBP3/PPARA/NCOA2	6
GJA1/ACHE/IL4/ADRB2/RB1/CAV1	6
GJA1/ACHE/IL4/ADRB2/RB1/CAV1	6
IL1B/GJA1/EGFR/NOS2/ACHE/IL1A/DPP4/PPARD	8
SOD1/FOS/IL2RA/IL4/RB1/MYC/ERBB2/JUN	8
TP53/IL1B/SOD1/IL2RA/IL1A/IL4/ERBB2/BCL2/IL6R	9
PLAU/SERPINE1/CYP1B1/DPP4	4
TP53/BCL2/HSP90AA1/E2F1	4
F2/IL4/CYP1B1/IL6R	4
FOS/EGFR/CAV1/CCND1/PTGES/AKR1C3	6
TP53/PPARG/STAT1/ICAM1/CD44	5
STAT1/IL2RA/IL4/ODC1/HSPB1/BCL2/HSP90AA1/BCL2L1/CYP1A1	9
MAPK1/GJA1/PPARA/G6PD/CCNB1	5
IL1B/AKT1/IGFBP3/CASP3/RB1/HSPB1/CAV1/CDKN1A/CCNB1	9
IGF2/AKT1/CASP3/IL4/PPARA/RB1/G6PD/BCL2/CCNB1	9
MAPK1/CXCL8/IL4/DPP4	4
GJA1/IL4/PTGES	3
IL1B/PLA2G4A/PTGES	3
IL1B/AKT1/GJA1/IL4/PPARA/ADRB2/CAV1/MDM2/KDR/HSP90AA1	10
SOD1/CYP3A4/PON1/IL4/G6PD/PPARD	6
TP53/AKT1/CASP3/BCL2L1/CASP7	5
IL1B/NOS3/MAPK1/STAT1/MYC/ERBB3/BCL2/HAS2	8

IL1B/NOS3/SOD1/GJA1/KCNH2/PPARA/ADRB2/G6PD/CAV1/PRKACA	10
NOS3/AKT1/PPARG/PTGS2/HSPB1/KDR	6
TP53/IGF2/AKT1/IGFBP3/PLA2G4A/PPARA/NCOA2/HAS2	8
IL1B/PLA2G4A/NOS2/PTGES	4
PPARG/PPARA/PPARD	3
CYP1B1/CYP2B6/CYP1A1	3
MAPK1/GJA1/PPARD	3
TP53/IL1B/PRKACA	3
FOS/CCND1/CDKN1A	3
ESR1/AR/PGR	3
AKT1/PPARG/AKR1C3	3
CASP3/IL4/BCL2/CDKN1A/AHR	5
MAPK1/SERPINE1/CXCL8/CCL2/IL4/IL6R/DPP4	7
CXCL8/EGFR/ICAM1/CAV1/DPP4/CDK1	6
GJA1/ACHE/IL4/ADRB2/RB1/CAV1	6
TP53/IL1B/MAPK1/ERBB2/BCL2/PRKACA/E2F1	7
CXCL8/EGFR/ICAM1/CAV1/BCL2L1/DPP4/CDK1	7
GJA1/ACHE/IL4/ADRB2/CAV1	5
TP53/MAPK1/CASP3/CHEK2/MDM2/BCL2/CDKN1A/HSP90AA1	8
ESR1/SOD1/ICAM1/BCL2	4
TP53/BCL2/HSP90AA1/E2F1	4
TP53/CXCL8/CCL2/CAV1/CCND1/JUN/BCL2/BCL2L1	8
EGFR/CAV1/KDR	3
ESR1/TP53/PPARG	3
STAT1/MYC/IL6R	3
MDM2/BCL2/CD44	3
IL1B/IGF2/IL2RA/IL1A/IL4	5
PPARG/CASP3/ICAM1/NQO1/CASP7	5
IL1B/SERPINE1/MMP9/STAT1/CAV1/CCND1	6
PLAU/SERPINE1/CASP3/F7/F3/PLAT/PRKACA	7
MAPK1/GJA1/PPARA/G6PD/CCNB1	5
MAPK1/IGF2/AKT1/CCNB1	4
SOD1/CYP3A4/PON1/IL4/G6PD/PPARD	6
IL1B/CHRNA7/CCL2/EGFR/ERBB2/ICAM1/KDR/CD44	8

F2/IL4/CYP1B1/CAV1/IL6R	5
TP53/AKT1/SOD1/PPARA/AKR1C3	5
AKT1/CHEK2/EGFR/ERBB2/CAV1/KDR/PRKACA	7
IL1B/AKT1/IL4/PPARA/ADRB2/CAV1/MDM2/KDR/HSP90AA1	9
PPARG/PPARA/PPARD	3
IGF2/ERBB3/BCL2	3
IGF2/ERBB3/BCL2	3
IL1B/SERPINE1/NOS2/F3/CD14	5
CYP1B1/BCL2/CYP1A1/AKR1C3	4
SOD1/MPO/EGFR/CYP1A1	4
AKT1/EGFR/CCND1/CYP1A1	4
IL1B/MAPK1/CHRNA7/SOD1/EGFR/ERBB2/CDK1	7
IL1B/MAPK1/CHRNA7/SOD1/EGFR/ERBB2/CAV1/CDK1	8
IL1B/GJA1/EGFR/NOS2/ACHE/IL1A/DPP4/PPARD	8
AKT1/CASP3/IL2RA/BCL2	4
IL1B/SERPINE1/F3/CD14	4
IL1B/IGF2/IL1A/RB1	4
PLAU/SERPINE1/PLAT	3
CYP3A4/CYP2B6/NR1I2	3
ESR1/TP53/PPARG	3
IL1B/CXCL8/CAV1	3
ESR1/TP53/PPARG	3
IGF2/ERBB3/BCL2	3
ESR1/AR/CASP3/PGR/MYC/KDR/BCL2/PRKACA	8
PLAU/MAPK1/MMP9/MPO/CTSD/HSP90AA1/SLPI/TNFAIP6/CD44/CD14	10
IL1B/PTGS2/IL1A/CYP1B1	4
MAPK1/CHRNA7/FOS/CASP3/EGFR/JUN/SLC2A4	7
F2/CHRNA7/GJA1/CCL2/KCNH2/G6PD/ICAM1/CAV1/BCL2/PRKACA	10
TP53/CHEK2/EGFR/PCNA/JUN	5
GJA1/PPARA/ADRB2/G6PD/CDKN1A	5
PLAU/MAPK1/MMP9/MPO/CTSD/HSP90AA1/SLPI/TNFAIP6/CD44/CD14	10
GJA1/CAV1/PRKACA	3
AKT1/IL4/CAV1/DPP4	4

F2/SERPINE1/F7/F3	4
NOS3/SERPINE1/ICAM1/BCL2L1	4
CYP3A4/CYP1B1/CYP1A1/PPARD/AKR1C3	5
IL1B/IGF2/IL1A/RB1/CCNB1	5
IL1B/AKT1/PPARA/CDK4	4
NR3C1/EGFR/ICAM1/AKR1C3	4
MAPK1/IGF2/AKT1/BCL2/PPARD/CCNB1	6
AKT1/PPARG/CCL2/CASP3/IL2RA/IL4/MYC/ERBB2/DPP4	9
F2/IL4/CYP1B1/CAV1/IL6R	5
ESR1/AR/MMP9/STAT1/MYC/BCL2/IL6R/HAS2	8
PPARG/FOS/CYP1A1	3
CYP3A4/CYP2B6/NR1I2	3
ESR1/TP53/EGFR	3
TP53/IL1B/MAPK1/PRKACA	4
ESR1/AR/PGR/CAV1/BCL2	5
MAPK1/AKT1/SERPINE1/CCL2/IL4	5
IL1B/PLA2G4A/PON1/CAV1/PTGES	5
IL1B/AKT1/CASP3/RB1/HSPB1/CAV1/CDKN1A	7
NOS3/SOD1/PPARA/ADRB2/G6PD/CAV1/PRKACA	7
ESR1/AR/PGR/MYC/KDR/BCL2	6
F2/CHRNA7/GJA1/CCL2/G6PD/ICAM1/CAV1/BCL2/PRKAC	9
IL1B/IGF2/AKT1/PPARG/CAV1/SLC2A4/CDK4/PRKACA	8
NOS3/AKT1/PPARG/PTGS2/HSPB1/KDR	6
CXCL8/EGFR/ICAM1/CAV1/DPP4/CDK1	6
ESR1/MAPK1/CCND1	3
IL1B/CXCL8/CAV1	3
ESR1/TP53/EGFR	3
TP53/BCL2/E2F1	3

TP53/BCL2/E2F1	3
PPARG/IGFBP3/CDKN1A/PPARD	4
TP53/IL1B/MAPK1/ERBB2/BCL2/PRKACA/E2F1	7
SERPINE1/CXCL8/ACHE/IL4/ADRB2/CAV1/HSP90AA1/CD14	8
SOD1/FOS/STAT1/IL2RA/IL4/RB1/MYC/ERBB2/JUN	9
TP53/AKT1/CHEK2/MDM2/BCL2/CD44	6
AR/TP53/PPARG/CASP3/BCL2/IL6R/MCL1	7
TP53/BCL2/IL6R	3
NOS3/EGFR/NOS2	3
TP53/CHEK2/MDM2/CDKN1A	4
MAPK1/PCNA/MYC/HSP90AA1	4
IL1B/CXCL8/CCL2/IL1A/ICAM1/HAS2	6
FOS/IL4/RB1/MYC/JUN	5
GJA1/BCL2/AKR1C3/PRKACA/HAS2	5
MAPK1/AKT1/BCL2/CDK1/PRKACA	5
PPARG/MMP9/RB1/JUN/E2F1	5
AKT1/PPARG/PTGS2/PPARD	4
IL1B/AKT1/MMP9/KCNH2/CAV1	5
CHRNA7/ACHE/NQO1	3
ESR1/AR/PGR	3
SLC6A2/GJA1/BCL2/DPP4	4
IL1B/PPARG/SOD1/CCL2	4
CXCL8/F7/F3/KDR	4
NR3C2/IL1B/EGFR/CD14	4
AKT1/PPARG/PPARA/PPARD	4
MAPK1/IL1A/CDKN1A/HSP90AA1/PRKACA	5
IL1B/AKT1/MMP9/KCNH2/CAV1	5
F2/MMP9/CCL2/KCNH2/ADRB2/G6PD/CAV1/PRKACA	8
AKT1/SOD1/HSPB1/MCL1	4
ESR1/AR/PGR/MYC/KDR/BCL2	6
NOS3/AKT1/EGFR/STAT1/IL2RA/MYC	6
TP53/NOS3/AKT1/NOS2/PON1/PPARA/CYP1A1/PPARD/AKR1C3	9
NR3C1/EGFR/ICAM1	3

STAT1/IL2RA/IL4/HSP90AA1	4
AKT1/CASP3/IL2RA/IL4/PPARA/ERBB2	6
MAPK1/CXCL8/CCL2/IL4/DPP4	5
TP53/BCL2/E2F1	3
AKT1/PPARG/AKR1C3	3
GJA1/PPARA/G6PD	3
GJA1/PPARA/G6PD	3
BCL2/BCL2L1/CD44	3
IL1B/AKT1/MMP9/GJA1/KCNH2/ICAM1/CAV1/ERBB3/BCL2	9
IL1B/AKT1/CASP3/RB1/HSPB1/CAV1/CDKN1A	7
MMP9/GJA1/CCL2/ADRB2/CAV1/BCL2/PRKACA	7
MAPK1/AKT1/BCL2/CDK1/PRKACA	5
MAPK1/GJA1/PPARD/CCNB1	4
AKT1/XIAP/MMP9/CD44	4
F2/SERPINE1/F7/F3	4
STAT1/IL4/HSP90AA1	3
GJA1/CAV1/PRKACA	3
TP53/PPARG/MYC	3
EGFR/PCNA/CCND1	3
MDM2/BCL2/CD44	3
IL1B/PLA2G4A/PTGES	3
AKT1/SOD1/CCL2/PPARA/ERBB3/BCL2	6
CXCL8/CCL2/PPARA/BCL2/SLPI	5
GJA1/EGFR/ACHE/IL1A/PPARD	5
CXCL8/STAT1/PPARA/CAV1/BCL2/SLPI	6
TP53/PPARG/XIAP/FOS/RUNX2/PPARA/CAV1/JUN	8
TP53/IL1B/PRKACA	3
FOS/CCND1/CDKN1A	3
CASP3/ICAM1/CASP7	3
IL1B/AKT1/GJA1/EGFR	4
TP53/F2/MAPK1/AKT1/MDM2/CDKN1A/CDK1	7

PGR/BCL2/TNFAIP6/CCNB1/PDE3A	5
MAPK1/CHRNA7/FOS/CASP3/EGFR/JUN/SLC2A4	7
TP53/BCL2/BCL2L1/E2F1	4
F2/NOS3/CASP3/IL2RA/IL4/ERBB2	6
NOS3/AKT1/PTGS2/HSPB1/KDR	5
GJA1/BCL2/DPP4	3
CYP3A4/CYP1A1/AKR1C3	3
MAPK1/MYC/HSP90AA1	3
IL1B/GJA1/EGFR/NOS2/ACHE/IL1A/DPP4/PPARD	8
IL1B/AKT1/PPARG/F3/MYC/CAV1/MDM2/CTSD	8
ESR1/IL1B/AKT1/GJA1/STAT1/HSPB1/CD14	7
IL1B/GJA1/EGFR/NOS2/ACHE/IL1A/DPP4/PPARD	8
GJA1/BCL2/DPP4	3
F2/NOS3/PLA2G4A	3
TP53/MDM2/CD44	3
ESR1/AR/TP53/ADRB2/MYC/MDM2/CDK1	7
IGF2/AKT1/PPARA/HAS2	4
ESR1/CYP3A4/CYP1B1/CYP1A1/AKR1C3	5
AKT1/PPARG/PTGS2/PPARD/E2F1	5
IL1B/GJA1/PLA2G4A/NOS2/PTGES/NCOA2/NCOA1	7
IL1B/CXCL8/CCL2/IL1A/ICAM1/HAS2	6
F2/AKT1/CCL2/KCNH2/ADRB2/G6PD	6
F2/AKT1/CCL2/KCNH2/ADRB2/G6PD	6
AKT1/CASP3/IL2RA/BCL2	4
MAPK1/IGF2/AKT1/NCOA1	4
PCNA/RB1/E2F1	3
TP53/G6PD/NQO1	3
AR/AKT1/IGFBP3	3
TP53/BCL2/E2F1	3
MAPK1/GJA1/PPARA/G6PD/ERBB3/CCNB1	6
IL1B/GJA1/EGFR/NOS2/ACHE/IL1A/DPP4/PPARD	8
AKT1/XIAP/MMP9/CD44	4
IL1B/GJA1/EGFR/NOS2/DPP4/PPARD	6

CHRNA7/CCL2/EGFR/ICAM1/KDR/CD44	6
AKT1/GJA1/EGFR/KCNH2/ICAM1/CAV1/PRKACA	7
EGFR/ICAM1/CAV1/DPP4/CDK1	5
SERPINE1/IGFBP3/PPARD	3
PPARG/MYC/JUN	3
IL1B/PLA2G4A/PTGES	3
NOS3/SOD1/ADRB2	3
ESR1/ESR2/EGFR	3
ESR1/TP53/JUN	3
F2/CCL2/KCNH2/ADRB2/G6PD	5
TP53/CASP3/EGFR/SLC2A4	4
PLAU/SERPINE1/CASP3/F7/F3/PLAT/PRKACA	7
TP53/AKT1/BCL2/HSP90AA1/E2F1	5
GJA1/BCL2/DPP4	3
EGFR/PCNA/JUN	3
GJA1/PPARA/G6PD	3
ESR1/TP53/PPARG	3
BCL2/BCL2L1/CD44	3
TP53/AKT1/BCL2/MCL1	4
CXCL8/PPARA/BCL2/SLPI	4
TP53/IL1B/PPARG/XIAP/IL4/PPARA/CAV1	7
IL1B/IGF2/CHEK2/IL1A/RB1/CCNB1/CDK1	7
TP53/BCL2/HSP90AA1/BCL2L1/E2F1	5
MAPK1/EGFR/PPARA/G6PD/CCNB1	5
AKT1/SOD1/HSPB1/MCL1	4
SOD1/CASP3/STAT1/RB1/G6PD	5
CYP3A4/CYP1B1/CYP1A1	3
PPARG/STAT1/PPARA	3
IGF2/AKT1/HAS2	3
PPARG/STAT1/PPARA	3
TP53/AKT1/BCL2/HSP90AA1/E2F1	5
IL1B/AKT1/PTGS2/PPARA	4
F2/SERPINE1/NOS2/IL6R	4
F2/MAPK1/AKT1/CDK1	4

ESR1/AR/TP53/ADRB2/MYC/MDM2/CDK1	7
IL1B/IGF2/CHEK2/IL1A/RB1/KDR/CCNB1/CDK1/PDE3A	9
IGF2/FOS/RB1/CAV1/BCL2	5
IGF2/ERBB3/BCL2	3
IL1B/CCNB1/CDK1	3
MAPK1/CXCL8/CCL2/IL4/DPP4	5
GJA1/CASP3/ICAM1/PPARD/NQO1/PRKACA	6
NOS3/NOS2	2
IL1B/PTGS2	2
AKT1/PCNA	2
AR/CCND1	2
MAOA/ACHE	2
F2/SERPINE1	2
ESR1/AR	2
STAT1/MYC	2
NOS3/AKT1/SOD1/GJA1/EGFR/ADRB2/ICAM1/CAV1/CDK4	9
F2/SERPINE1/NOS2/PPARA	4
IL4/BCL2/CDKN1A	3
GJA1/EGFR/ACHE/IL1A/PPARD	5
IL1B/AKT1/GJA1/CAV1/MDM2/HSP90AA1	6
IL1B/IGF2/AKT1/PPARG/SLC2A4/CDK4	6
IL1B/GJA1/EGFR/NOS2/ACHE/IL1A/DPP4/PPARD	8
NOS2/IL4/IL6R	3
IGF2/AKT1/PPARA	3
AR/NQO1/CDK4	3
STAT1/IL4/HSP90AA1	3
IGF2/ERBB3/BCL2	3
GJA1/ADRB2/ICAM1	3
TP53/AKT1/CASP3/IL4/ADRB2/KDR/PRKACA	7
AR/NOS3/GJA1/ADRB2	4
F2/ERBB3/KDR/PPARD	4
TP53/BCL2/BCL2L1/E2F1	4
IL1B/MAPK1/CHRNA7/SOD1/CDK1	5

FOS/IL2RA/IL4/RB1/JUN	5
FOS/IL2RA/IL4/RB1/JUN	5
AKT1/PPARG/ICAM1/SLC2A4/PPARD/PRKACA	6
IL1B/MAPK1/CHRNA7/CCL2/EGFR/ACHE/ADRB2/PRKACA	8
AKT1/PPARG/ICAM1/SLC2A4/PPARD/PRKACA	6
AKT1/PLA2G4A/PPARA/CYP1B1/PPARD/AKR1C3	6
IL1B/MAPK1/CHRNA7/CCL2/EGFR/ACHE/ADRB2/PRKACA	8
SOD1/ICAM1/NQO1	3
F2/PPARG/PPARD	3
AKT1/EGFR/ERBB2	3
NOS3/AKT1/PPARG/PTGS2/HSPB1/KDR	6
NOS3/NOS2	2
IL1A/BCL2	2
PPARG/CCND1	2
ESR1/TP53	2
TP53/PPARG	2
F2/NOS3/MAPK1/PLA2G4A/HSPB1	5
IGF2/FOS/RB1/CAV1/BCL2	5
IL1B/F2/PPARG/E2F1	4
FOS/STAT1/JUN/AHR	4
AKT1/SOD1/HSPB1/MCL1	4
ESR1/CASP3/PGR	3
IGF2/CASP3/IL2RA	3
BCL2/CCNB1/PDE3A	3
IGF2/ERBB3/BCL2	3
TP53/CAV1/CD14	3
ESR1/TP53/JUN	3
IL1B/AKT1/MMP9/KCNH2/ICAM1/CAV1/BCL2	7
TP53/MAPK1/IGF2/AKT1/SOD1/CXCL8/KDR/NCOA1	8
CYP3A4/CYP1B1/CYP1A1/AKR1C3	4
IL1B/PPARG/SOD1/CCL2	4
CHRNA7/IL1A/IL4/HSPB1/CD14	5
MAPK1/AKT1/NCOA1	3
TP53/BCL2/E2F1	3
F2/CAV1/BCL2/PRKACA	4

F2/AKT1/EGFR/ERBB2/BCL2	5
IGF2/AKT1/PPARA/HAS2	4
IL1B/HSPB1/KDR/HSP90AA1	4
AKT1/MMP9/EGFR/ERBB2	4
CHRNA7/IL1A/IL4/HSPB1/CD14	5
CYP3A4/CYP1A1/PPARD	3
IGF2/AKT1/HAS2	3
MAPK1/SOD1/BCL2	3
MAPK1/CHRNA7/CASP3/ERBB2/CDK4	5
IL1B/SERPINE1/F3/CD14	4
PPARG/SERPINE1/IL4/CD14	4
CCNB1/CDK1	2
SERPINE1/CYP1B1	2
GSTM1/CYP1A1	2
MMP9/HAS2	2
PPARA/G6PD	2
GJA1/PRKACA	2
MAPK1/CAV1	2
TP53/EGFR	2
IL4/PPARA	2
CHRNA7/IL1A/IL4/HSPB1/CD14	5
NOS2/IL4/ICAM1/BCL2L1	4
AKT1/CCL2/DPP4	3
CAV1/CCND1/NCOA1	3
TP53/AKT1/BCL2L1	3
IGF2/CASP3/IL2RA	3
PTGS2/ADRB2/SLC2A4	3
STAT1/ICAM1/IL6R	3
IGF2/PPARG/CCL2	3
IGF2/PPARG/CCL2	3
NOS3/AKT1/NOS2/PON1/PPARA/PPARD	6
AKT1/SERPINE1/XIAP/MMP9/SLPI/CD44	6

CHRNA7/IL1A/IL4/HSPB1/CD14	5
IL1B/AKT1/SOD1/AKR1C3	4
AR/AKT1/EGFR/BCL2L1	4
BCL2/CCNB1/PDE3A	3
F2/PPARG/PPARD	3
RB1/DPP4/HAS2	3
IL1B/GJA1/EGFR/NOS2/DPP4/PPARD	6
AR/TP53/MAPK1/IGF2/AKT1/CCNB1/NCOA1	7
PPARG/IL4	2
PPARG/PPARA	2
NOS3/NOS2	2
IL1B/PTGS2	2
IL1B/CCNB1	2
AR/IGFBP3	2
CCL2/ICAM1	2
RB1/HSP90AA1	2
PPARG/MMP9/PON1/RB1/CAV1	5
FOS/STAT1/RB1/JUN	4
TP53/AKT1/CASP3/IL2RA	4
IL1B/IGF2/IL1A/MYC/BCL2L1	5
PPARG/GJA1/PTGES/NCOA1	4
TP53/IL1B/MAPK1/PRKACA	4
IL1B/IGF2/CHEK2/IL1A/RB1/CCNB1/CDK1/PDE3A	8
MAPK1/GJA1/CCNB1	3
TP53/AKT1/IL2RA	3
AKT1/PPARG/PPARA/PPARD	4
ESR1/XIAP/CAV1/CD14	4
STAT1/ICAM1/IL6R	3
GJA1/CAV1	2
PPARG/PPARA	2
IL1B/HAS2	2
PPARG/PPARD	2
PPARG/CAV1	2
ESR1/TP53	2

SOD1/ADRB2	2
MMP9/HAS2	2
F2/SERPINE1	2
PPARA/RB1	2
MYC/IL6R	2
PPARA/PPARD	2
NR3C2/IL1B/EGFR/CD14	4
AKT1/SERPINE1/XIAP/MMP9/SLPI/CD44	6
F2/PPARG/PPARD	3
TP53/CAV1/CD14	3
NOS3/AKT1/NOS2/PON1/PPARA/PPARD	6
FOS/STAT1/IL4/RB1/MYC/JUN	6
MMP9/CCL2/ADRB2/CAV1/PRKACA	5
IL1B/GJA1/NOS2/DPP4/PPARD	5
TP53/IL1B/MAPK1/ERBB2/BCL2/PRKACA/E2F1	7
IGF2/AKT1/F3/KDR	4
IL1B/GJA1/EGFR/NOS2/DPP4/PPARD	6
STAT1/IL2RA/IL4/BCL2/HSP90AA1/BCL2L1	6
STAT1/IL2RA/IL4/BCL2/HSP90AA1/BCL2L1	6
ESR1/TP53/EGFR	3
F2/NOS2/IL4/ICAM1/BCL2L1	5
TP53/MMP9/GJA1/MMP2/PRKACA	5
IL1B/AKT1/SLC2A4/PPARD	4
IL1B/MAPK1/AKT1/EGFR/MYC	5
PGR/CDKN1A	2
IL1B/PLA2G4A	2
CYP1B1/CYP1A1	2
PPARG/PPARD	2
CYP1A1/AKR1C3	2
CCNB1/CDK1	2
CXCL8/CAV1	2
CYP1B1/AKR1C3	2
IL4/IL6R	2
MDM2/CD44	2
MAPK1/CDK1	2
MAPK1/CDK1	2
CCNB1/CDK1	2
CCL2/KDR	2

AKT1/PPARG	2
ESR1/AR	2
STAT1/MYC	2
BCL2/CD44	2
ESR1/TP53	2
AKT1/CCL2	2
HSPB1/KDR	2
IL1B/PTGS2	2
AKT1/PPARG/PPARA/PPARD	4
TP53/CXCL8/CCL2	3
NOS3/MAPK1/PTGS2/NOS2/IL4/PPARA	6
TP53/IL4/ICAM1/IL6R	4
IGF2/AKT1/PPARA	3
GJA1/KCNH2/CAV1	3
IL1B/AKT1/SLC2A4/PPARD	4
IL1B/MAPK1/AKT1/EGFR/MYC	5
NOS3/MAPK1/PTGS2/NOS2/IL4/PPARA	6
PLAU/SERPINE1/KDR/BCL2/PPARD/CD44/HAS2	7
CXCL8/CCL2/HSPB1/CCND1/HSP90AA1	5
GJA1/CASP3/ICAM1/PPARD/PRKACA	5
NR3C2/IL1B/AKT1/EGFR/CD14	5
IL1B/SERPINE1/GJA1/PTGS2/EGFR/HSP90AA1/PRKACA	7
NR3C1/EGFR/ICAM1	3
TP53/BCL2/E2F1	3
CXCL8/STAT1/PPARA/BCL2/SLPI	5
AKT1/PPARG/CYP3A4/PPARA/CYP1A1/PPARD	6
IL1B/AKT1/SLC2A4/PPARD	4
TP53/G6PD	2
BCL2/CDKN1A	2
PTGS2/PON1	2
PGR/TNFAIP6	2
IL1B/CD44	2
IGF2/AKT1	2
NOS2/BCL2L1	2

SOD1/MCL1	2
PPARG/PPARA/PPARD	3
ESR1/TP53/EGFR	3
PPARG/MMP9/RB1	3
ESR1/TP53/EGFR	3
MMP9/GJA1/MMP2/PRKACA	4
AR/AKT1/SOD1/CAV1	4
CASP3/STAT1/RB1/G6PD	4
IL1B/AKT1/CD44/HAS2	4
IL1B/F2/STAT1/NOS2/IL2RA/IL4/ICAM1/HSP90AA1	8
TP53/IL1B/MAPK1/RB1/MYC/CCNB1	6
IL1B/AKT1/SLC2A4/PPARD	4
SOD1/CYP3A4/G6PD/CYP1A1/AKR1C3	5
GJA1/CASP3/ICAM1/PPARD/PRKACA	5
ESR1/AR/ESR2	3
AKT1/CAV1/DPP4	3
F7/F3/PLAT	3
GJA1/CAV1/PRKACA	3
F2/NOS3	2
AKT1/SOD1	2
SOD1/CYP1A1	2
TP53/CDKN1A	2
AKT1/BCL2L1	2
BCL2/CD44	2
TP53/PPARG/PPARA/CDK1	4
IL1B/NOS3/PPARA/G6PD	4
F2/CCL2/G6PD/CAV1	4
FOS/RB1/JUN	3
PLAU/SERPINE1/PLAT	3
MAPK1/GJA1/CCNB1	3
TP53/BCL2/E2F1	3
CASP3/IL4/BCL2/CDKN1A/AHR	5
CASP3/IL2RA/IL4/ERBB2	4
TP53/MAPK1/AKT1/CDKN1A/HSP90AA1	5
IL1B/GJA1/EGFR/PLA2G4A/NOS2/DPP4/PPARD/PTGES	8
MMP9/STAT1/MYC/BCL2/IL6R/HAS2	6

TP53/CHEK2/EGFR/PCNA/JUN/CDK1	6
SOD1/ADRB2/CAV1	3
NOS3/NOS2/IL4/PPARA	4
IL1B/GJA1/CAV1/KDR/PRKACA	5
IL1B/GJA1/PLA2G4A/NOS2/PTGES/NCOA2/NCOA1	7
GJA1/CASP3/ICAM1/PPARD/PRKACA	5
CASP3/STAT1/RB1/G6PD	4
ESR1/TP53/PPARG/EGFR	4
AR/AKT1/EGFR/BCL2L1	4
IL4/ADRB2/KDR	3
TP53/BCL2/E2F1	3
TP53/G6PD	2
CCND1/CD14	2
SOD1/EGFR	2
IL1B/IL4	2
IL1B/E2F1	2
MAPK1/AKT1	2
AKR1C3/HAS2	2
IGF2/AKT1	2
MMP9/STAT1	2
NOS3/NOS2/IL4/PPARA	4
CXCL8/EGFR/ERBB3/KDR/PRKACA	5
IL1B/MAPK1/PPARG/SOD1/CCL2/HSP90AA1/CD14	7
TP53/BCL2/BCL2L1	3
ESR1/AR/GJA1	3
NOS2/JUN/BCL2L1/SLPI	4
PPARG/F3/MYC/CAV1/CTSD	5
TP53/IL1B/F2/PPARG/BCL2/PDE3A/E2F1/HAS2	8
TP53/PPARG/GJA1/CDKN1A	4
ESR1/TP53/PPARG/EGFR	4
TP53/IGF2/MMP9/GJA1/MMP2/RUNX2/CD44/HAS2	8
MMP9/STAT1/MYC/BCL2/IL6R/HAS2	6

TP53/BCL2/E2F1	3
PLAU/SERPINE1/KDR/BCL2	4
ESR1/TP53/PPARG/EGFR	4
TP53/FOS/PPARA/CAV1/JUN	5
IL1B/PPARG/BCL2/PDE3A/E2F1/HAS2	6
CCL2/DPP4	2
CCL2/IL4	2
IGF2/BCL2	2
KDR/BCL2	2
STAT1/MYC	2
SOD1/GJA1/KCNH2/CAV1/PRKACA	5
AKT1/SOD1/ERBB2/PPARD	4
AKT1/SOD1/ERBB2/PPARD	4
IL1B/PPARG/E2F1	3
TP53/MAPK1/CHRNA7/AKT1/CASP3/HSPB1/ERBB2/CDK4	8
SOD1/GSTM1/G6PD	3
AKT1/GJA1/CAV1	3
TP53/BCL2/E2F1	3
IGF2/PPARA/G6PD	3
PPARG/RUNX2/IL6R/PRKACA	4
PPARG/SERPINE1/IL4/CAV1/CD14	5
CXCL8/CCL2/HSPB1/CCND1/HSP90AA1	5
IL1B/GJA1/NOS2/DPP4/PPARD	5
NOS3/NOS2	2
AKT1/PPARA	2
SOD1/EGFR	2
CCL2/KDR	2
CCL2/KDR	2
CHRNA7/CASP3	2
AKT1/HSPB1	2
TP53/CAV1/CD14	3
ESR1/F2/CHRNA7/GJA1/EGFR/CAV1/BCL2/PRKACA	8
CYP3A4/CYP1A1/PPARD/AKR1C3	4

F2/MAPK1/AKT1/CDK1	4
TP53/IL4/PPARA/CAV1	4
IGF2/AKT1/PPARA/G6PD/HAS2	5
F2/AKT1/GJA1/CAV1/PRKACA	5
CASP3/IL2RA/ERBB2	3
TP53/BCL2/E2F1	3
PPARG/ESR2/PPARA	3
F2/NOS2/BCL2L1	3
PPARA/AHR/NCOA2	3
IL1B/GJA1/EGFR/NOS2/DPP4/PPARD	6
STAT1/MYC	2
BCL2/IL6R	2
PON1/CAV1	2
MYC/JUN	2
SERPINE1/KDR	2
IL4/PPARA	2
IL1B/SERPINE1/PTGS2/EGFR	4
TP53/PPARG/PPARA/CAV1	4
SERPINE1/CCL2/IL6R	3
F2/PPARG/CDK1	3
F2/NOS2/PPARA	3
F2/CCL2/ICAM1/KDR/CD44/HAS2	6
PPARG/F3/MYC/CTSD	4
SOD1/CCL2/ERBB3/BCL2	4
GJA1/KCNH2/CAV1/PRKACA	4
F2/NOS2/PPARA	3
IL1B/IGF2/CDK4	3
STAT1/MYC/BCL2	3
TP53/BCL2/E2F1	3

NOS3/NOS2	2
AKT1/PCNA	2
SOD1/ERBB2	2
KDR/PRKACA	2
CYP3A4/CYP1A1	2
BCL2/IL6R	2
NOS2/IL4	2
TP53/MYC	2
SOD1/ADRB2	2
KDR/BCL2	2
TP53/RUNX2	2
IL1B/GJA1/ICAM1/KDR	4
PLAU/SERPINE1/KDR/BCL2/HAS2	5
ESR1/CYP3A4/CYP1B1/CYP1A1/AKR1C3	5
ESR1/F2/GJA1/CAV1/BCL2/PRKACA	6
EGFR/PCNA/CCND1/CYP1A1	4
STAT1/MYC/BCL2/IL6R	4
IL1B/GJA1/EGFR/NOS2/DPP4/PPARD	6
IGF2/AKT1/HAS2	3
TP53/AKT1/IL2RA	3
STAT1/MYC/BCL2	3
F7/F3	2
AKT1/PPARG	2
CXCL8/CCL2	2
IL1B/PTGS2	2
NOS3/NOS2	2
PPARA/G6PD	2
AKT1/CCL2	2
CCL2/IL4	2
CCL2/AKR1C3	2
EGFR/PCNA/CCND1/CYP1A1	4
AKT1/PPARG/PLA2G4A	3
AKT1/HSPB1/KDR	3

F2/MAPK1/EGFR	3
GJA1/KCNH2/CAV1	3
TP53/BCL2/E2F1	3
TP53/IL1B/IGF2/CCNB1	4
IGF2/FOS/RB1/CAV1/ERBB3/BCL2	6
IGF2/AKT1/IL6R	3
CDKN1A/PPARD/CCNB1	3
MAPK1/CXCL8/CCL2/CD44	4
PPARG/XIAP/STAT1/IL2RA/IL4/HSP90AA1/PRKACA	7
TP53/MAPK1/AKT1/CDKN1A	4
IL1B/IGF2/AKT1/CDK4	4
CCNB1/PDE3A	2
CDKN1A/CDK1	2
AR/IGFBP3	2
IGF2/NCOA1	2
HSPB1/KDR	2
MAPK1/CHRNA7/CCL2/EGFR	4
ESR1/TP53/PPARG/EGFR	4
FOS/MYC/CYP1A1	3
FOS/MYC/CYP1A1	3
AKT1/PPARA/PPARD	3
IL1B/GJA1/ACHE/CAV1/KDR/BCL2/PRKACA	7
SOD1/IL2RA/IL4/ERBB2	4
PLAU/SERPINE1/KDR/BCL2/CD44	5
GJA1/KCNH2/CAV1	3
AKT1/SOD1	2
AKT1/SOD1	2
IGF2/BCL2	2
IL1B/AKT1	2
ESR1/TP53	2
TP53/G6PD	2
PPARG/STAT1	2
PPARG/STAT1	2
ESR1/AR	2

RB1/CDK1	2
CHRNA7/CASP3	2
PPARG/CCL2	2
CHRNA7/MDM2	2
IL1B/MAPK1/STAT1/BCL2/HAS2	5
IL1B/GJA1/CAV1/PRKACA	4
SOD1/CASP3/ICAM1/BIRC5	4
TP53/CASP3/IL4/BCL2/CDKN1A/AHR	6
STAT1/HSP90AA1/CD14	3
ESR1/CAV1/CD14	3
PPARA/G6PD/CCNB1	3
MAPK1/CXCL8/CCL2/CD44	4
CXCL8/PPARA/BCL2/SLPI	4
IL1B/AKT1/SLC2A4/PPARD	4
GJA1/KCNH2/CAV1/PRKACA	4
IGF2/AKT1/HAS2	3
CAV1/KDR/HAS2	3
IL1B/NOS3/GJA1	3
STAT1/MYC	2
IL1B/CD44	2
IGF2/IL2RA	2
NOS3/BCL2	2
GJA1/PRKACA	2
AKT1/CAV1/MDM2/HSP90AA1	4
F2/CCL2/ICAM1/KDR	4
PPARG/F3/MYC/CTSD	4
F2/CAV1/BCL2/PRKACA	4
IGFBP3/RB1/PPARD	3
CASP3/IL2RA/IL4/ERBB2	4
NOS2/IL4	2
IL1B/PTGS2	2
IL1B/PLA2G4A	2
NOS3/NOS2	2
IL1B/PPARA	2
ESR1/AR	2

CXCL8/IL4	2
TP53/MMP9	2
CXCL8/CCL2	2
CHRNA7/ADRB2	2
CASP3/IL2RA/ERBB2	3
ESR1/F2/CHRNA7/GJA1/CAV1/BCL2/PRKACA	7
STAT1/MYC/BCL2	3
CASP3/IL2RA/ERBB2	3
TP53/IL1B/MAPK1/AKT1/CDKN1A/PRKACA	6
RUNX2/IL6R/PPARD/CD44/CDK4	5
CASP3/BCL2	2
CYP1A1/AKR1C3	2
AKT1/SOD1	2
TP53/CAV1	2
KCNH2/CAV1	2
CHRNA7/ACHE	2
ESR1/IL1B/GJA1/STAT1/HSPB1	5
PPARA/G6PD/CCNB1	3
TP53/IL1B/MAPK1/AKT1/CDKN1A/PRKACA	6
IL1B/AKT1/CD44/HAS2	4
F2/SERPINE1/MPO/NOS2/IL6R/SLPI	6
ESR1/F2/GJA1/CAV1/BCL2/PRKACA	6
AR/IL1B/ICAM1/CAV1	4
IGF2/PPARA/G6PD/BCL2	4
ESR1/AR/GJA1/PDE3A	4
IGF2/BCL2	2
MAOA/ACHE	2
SERPINE1/KDR	2
ESR1/TP53	2
ESR1/RB1	2
IL4/IL6R	2
ICAM1/CAV1	2
TP53/BCL2L1	2
KCNH2/CAV1	2
TP53/PPARA	2

TP53/EGFR	2
IL4/CAV1/IL6R	3
BCL2/CCNB1/PDE3A	3
ESR1/F2/CHRNA7/GJA1/CAV1/BCL2/PRKACA	7
MAPK1/PCNA/MYC/HSP90AA1	4
SOD1/ADRB2/CAV1/PRKACA	4
MAPK1/KCNH2/IL1A/BCL2	4
TP53/AKT1/SERPINE1/XIAP/MMP9/SLPI/CD44	7
CAV1/CCND1/NCOA1	3
TP53/PPARA/CAV1	3
TP53/IL1B/CCNB1	3
IL1B/IL1A/IL6R	3
IGF2/AKT1/HAS2	3
IL1B/IGF2/IL1A	3
TP53/PPARG/XIAP/PPARA/CAV1	5
AKT1/CAV1/MDM2/HSP90AA1/PRKACA	5
IL1B/NOS2/IL1A/IL6R	4
SOD1/ICAM1	2
IGF2/AKT1	2
ESR1/CYP3A4	2
IGF2/AKT1	2
IL1B/IL1A	2
CDKN1A/CYP1A1	2
CCL2/EGFR	2
MAPK1/CCNB1	2
TP53/EGFR	2
MAPK1/AKT1	2
STAT1/IL6R	2
STAT1/MYC	2
CHRNA7/ACHE	2
CHRNA7/ACHE	2
MAOA/ODC1/CYP1A1/NQO1	4
TP53/MAPK1/AKT1/CDKN1A	4

IGF2/AKT1/MYC	3
IL1A/HSPB1/CD14	3
MMP9/KCNH2/CAV1	3
SOD1/GJA1/KCNH2/ADRB2/CAV1/PRKACA	6
TP53/IL1B/MAPK1/CCNB1	4
ESR1/TP53/MMP1/ICAM1/HSP90AA1	5
TP53/EGFR/ERBB2/BCL2/HSP90AA1/E2F1	6
IL4/CAV1/IL6R	3
AKT1/CAV1/MDM2	3
STAT1/MYC/BCL2	3
CASP3/IL2RA/ERBB2	3
STAT1/MYC/BCL2	3
TP53/AKT1/BCL2/MCL1/E2F1	5
SOD1/CASP3/EGFR/PPARA/BCL2/PPARD/AKR1C3	7
NOS3/RB1	2
PON1/CAV1	2
PON1/CAV1	2
RB1/PRKACA	2
CHEK2/CCNB1	2
AKT1/MMP9/EGFR	3
GJA1/EGFR/ADRB2	3
MMP9/CCL2/ADRB2/CAV1/PRKACA	5
SOD1/CASP3/ICAM1/BIRC5	4
PLA2G4A/PON1/ACHE	3
STAT1/MYC/BCL2	3
PCNA/CYP1B1/NQO1	3
IL1A/HSPB1/CD14	3
PPARA/G6PD/BCL2/CCNB1	4
TP53/PPARG	2
PPARG/PPARA	2
HSPB1/KDR	2

CASP3/CDKN1A	2
PPARG/AKR1C3	2
CXCL8/DPP4	2
SOD1/EGFR	2
ESR1/RB1/CDK1	3
AKT1/BCL2/CCNB1/PDE3A/PRKACA	5
IL1B/MAPK1/AKT1/EGFR/MYC	5
AKT1/GJA1/CAV1	3
PPARA/G6PD	2
PPARA/G6PD	2
NCOA2/NCOA1	2
IL1B/AKT1	2
AKT1/PPARG	2
IL1B/E2F1	2
PPARA/G6PD	2
IL4/IL6R	2
CASP3/CDKN1A	2
AR/GJA1	2
IL1B/AKT1/CD44/HAS2	4
AKT1/PTGS2/KDR	3
PPARA/G6PD/BCL2	3
F2/CAV1/BCL2/PRKACA	4
CHRNA7/CASP3/ACHE	3
MAPK1/PCNA/MYC/HSP90AA1	4
ESR1/F2/CHRNA7/GJA1/CAV1/BCL2/PRKACA	7
TP53/CHEK2	2
PPARA/G6PD	2
KCNH2/CAV1	2
AKT1/PPARG	2
PTGS2/KDR	2
GJA1/EGFR/PPARD	3
HSP90AA1/CDK1/PRKACA	3

SOD1/IL2RA/IL4/ERBB2	4
AR/MAPK1/AKT1/SOD1/CAV1/CCNB1/CDK1	7
GJA1/KCNH2/CAV1/PRKACA	4
SLC6A2/PON1/CAV1/NCOA2/NCOA1	5
RB1/CCNB1	2
TP53/AKT1	2
SOD1/EGFR	2
ESR1/TP53	2
KCNH2/CAV1	2
CHRNA7/ACHE	2
F2/PPARA	2
PPARA/CAV1	2
IL1B/AKT1	2
KCNH2/CAV1/PRKACA	3
NOS3/PPARA/G6PD	3
TP53/BCL2/HSP90AA1/BCL2L1/E2F1	5
IL1B/IL4	2
NOS3/RB1	2
PPARA/G6PD	2
CDKN1A/CDK1	2
KCNH2/CAV1	2
BCL2/IL6R	2
IL1B/F2/CXCL8/CCL2/BCL2/SLPI	6
IL1B/PLA2G4A/IL4	3
MAPK1/CCL2/PTGES	3
FOS/MYC/CYP1A1	3
ICAM1/HSP90AA1/CDK1/PRKACA	4
IL1B/AKT1/MYC/CAV1	4
IL1B/MAPK1/AKT1/EGFR/MYC	5
CASP3/IL2RA/IL4/ERBB2	4
PPARG/F3/MYC/CTSD	4
AKT1/CAV1/MDM2/PRKACA	4

SOD1/ADRB2/CAV1	3
CHRNA7/AKT1/ADRB2	3
TP53/IL1B/CCNB1	3
NOS2/IL4	2
ADRB2/PRKACA	2
CDKN1A/CDK1	2
IGF2/AKT1	2
ESR1/TP53	2
SOD1/GJA1/KCNH2/CAV1/PRKACA	5
TP53/BCL2/NCOA1	3
IL4/MYC/ERBB2	3
TP53/IL1B/IGF2/CCNB1	4
IGF2/AKT1/HAS2	3
PRKACA/HAS2	2
STAT1/MYC	2
MAPK1/CCL2	2
PPARA/G6PD	2
PPARD/CDK4	2
PPARG/CDKN1A	2
CXCL8/CCL2/DPP4	3
NOS2/IL4/CCNB1/CDK1	4
IL1B/IL4/ICAM1	3
IL1B/PLA2G4A/IL4	3
AKT1/CAV1/MDM2	3
ESR1/AR	2
AKT1/MMP9	2
AKT1/EGFR	2
AKT1/F7	2
CASP3/SLC2A4	2
KCNH2/CAV1	2
TP53/IL4/ICAM1/IL6R	4
CHRNA7/AKT1/ADRB2	3
IL4/MYC/ERBB2	3
TP53/CHEK2/CDKN1A/HSP90AA1	4
AKT1/PPARA/PPARD	3

STAT1/MYC/BCL2	3
SOD1/GJA1/KCNH2/CAV1/PRKACA	5
GJA1/NR1I2	2
MAPK1/CCNB1	2
CCL2/AKR1C3	2
IL4/BCL2/IL6R	3
EGFR/PCNA/MYC	3
NOS3/AKT1/EGFR	3
TP53/CAV1	2
ESR1/AR	2
IL1B/PLA2G4A	2
NQO1/CD14	2
AKT1/MMP9	2
IL4/PPARA	2
NOS3/RB1	2
AKT1/CAV1/MDM2	3
NOS3/NOS2/IL4/PPARA	4
NOS3/ERBB3	2
PPARG/NCOA1	2
ESR1/AR	2
AKT1/GJA1	2
CXCL8/DPP4	2
SOD1/PLA2G4A/GSTM1/G6PD	4
CXCL8/ACHE/CAV1	3
AKT1/SOD1/EGFR	3
MAPK1/ADRB2/BCL2	3
CCL2/PON1/ADRB2	3
TP53/NQO1	2
ICAM1/CAV1	2
CCL2/ADRB2	2
F2/NOS2/ICAM1	3
IGF2/AKT1/HAS2	3
PPARA/G6PD/CCNB1	3
KDR/BCL2	2
ESR1/EGFR	2
PON1/CAV1	2
MMP9/MMP2	2
MAPK1/MMP2	2

MAPK1/CCNB1	2
CHRNA7/ADRB2	2
MAPK1/IL1A/BCL2	3
AKT1/XIAP/CAV1/HSP90AA1	4
BCL2/HAS2	2
AKT1/IL6R	2
RB1/BCL2	2
TP53/NCOA2	2
IL1B/GJA1	2
KCNH2/CAV1	2
NOS3/ADRB2	2
IGF2/AKT1	2
TP53/NCOA2	2
IGF2/AKT1	2
MAPK1/CDKN1A	2
ESR1/EGFR	2
MMP9/CAV1	2
ESR1/XIAP/CAV1/CD14	4
NOS3/NOS2/IL4/PPARA	4
TP53/IL1B/F2/AKT1/PPARG/E2F1	6
AKT1/CCL2/ICAM1	3
IL1B/NOS2/IL4/ICAM1	4
IL1B/GJA1/CAV1/PRKACA	4
IL1B/ICAM1/KDR	3
TP53/IL4/PPARA	3
TP53/IL4/PPARA/CCNB1/CDK1	5
SOD1/HSPB1	2
MMP9/RB1	2
NOS3/NOS2	2
IL4/ERBB2	2
CHEK2/PCNA	2
IL4/ADRB2/KDR	3
GJA1/KCNH2/CAV1	3
PPARG/XIAP/STAT1/HSP90AA1/PRKACA	5
FOS/MYC/CYP1A1	3
IL4/MYC	2
IL1B/E2F1	2

MMP9/STAT1	2
HSPB1/KDR	2
TP53/BCL2	2
MAPK1/CAV1	2
PPARD/HAS2	2
BCL2/IL6R	2
CXCL8/CAV1	2
ESR1/AR	2
IL1B/ICAM1	2
CHRNA7/CASP3	2
AKT1/E2F1	2
MAPK1/AKT1/IL6R	3
AKT1/CAV1/MDM2/PRKACA	4
AKT1/MMP9/EGFR	3
CXCL8/CCL2/DPP4	3
AKT1/MYC	2
ESR1/STAT1	2
IGFBP3/PPARD	2
AKT1/MYC	2
NOS3/MYC	2
MAPK1/CDKN1A	2
AKT1/SOD1/ERBB2	3
GJA1/EGFR/PPARD	3
IL1B/NOS2/IL4	3
CXCL8/ADRB2	2
TP53/PPARA	2
SERPINE1/IL4	2
NOS3/NOS2	2
AKT1/HSPB1	2
AKT1/E2F1	2
MAPK1/EGFR	2
TP53/NQO1	2

TP53/PPARA/CAV1	3
CXCL8/CCL2/CCND1	3
ICAM1/PPARD/PRKACA	3
AKT1/CAV1/MDM2	3
IGF2/SERPINE1/SOD1	3
STAT1/HSP90AA1/CD14	3
AR/AKT1/EGFR/CAV1/BCL2L1	5
MYC/BCL2	2
CCNB1/CDK1	2
JUN/E2F1	2
MPO/PRKACA	2
STAT1/HSP90AA1/CD14	3
IL1B/AKT1/EGFR	3
ICAM1/PPARD/PRKACA	3
ESR1/TP53/MMP1/RB1/ICAM1/HSP90AA1	6
ICAM1/PPARD/PRKACA	3
TP53/IL1B/IGF2/CCNB1/CDK1/NCOA1	6
MMP9/MMP2	2
NOS3/RB1	2
F2/CAV1	2
EGFR/ERBB2	2
MAPK1/CCL2	2
STAT1/MYC/BCL2	3
AKT1/PPARA/PPARD	3
AKT1/CAV1/MDM2	3
RB1/CYP1B1	2
IL1B/PTGS2	2
MAPK1/CAV1	2
MAPK1/ERBB2/CDK4	3
TP53/IL1B/F2	3
ICAM1/CAV1	2
IL4/ERBB2	2
CHRNA7/CASP3	2
GJA1/KCNH2/CAV1	3
IL4/PPARA	2

IL1B/AKT1	2
MPO/PRKACA	2
CHRNA7/AKT1/ADRB2	3
TP53/IL1B/IGF2/CCNB1/CDK1/NCOA1	6
TP53/IL4/BCL2	3
NOS3/CHRNA7/ADRB2	3
CHRNA7/CASP3	2
TP53/IL1B/F2	3
ICAM1/PPARD/PRKACA	3
AKT1/XIAP/CAV1/HSP90AA1	4
TP53/BCL2/NCOA1	3
SOD1/XIAP/MYC	3
TP53/IL1B/F2/PPARG/E2F1	5
MYC/BCL2	2
MMP9/STAT1	2
MAPK1/FOS/JUN/HSP90AA1	4
AR/CASP3/PRKACA	3
RB1/CCNB1	2
IL1B/IL1A	2
CHEK2/PCNA	2
PLA2G4A/IL4	2
SOD1/AKR1C3	2
MYC/BCL2	2
SOD1/HSPB1	2
F2/CXCL8/SLPI	3
ESR1/EGFR	2
IL1B/IL6R	2
IL1B/CD44	2
IL1B/GJA1	2
G6PD/BCL2	2
ICAM1/BCL2	2
GJA1/KCNH2	2
AKT1/EGFR	2
GJA1/CHEK2/CCNB1	3
F2/CXCL8/ADRB2	3

AKT1/BCL2/CCNB1/PDE3A/PRKACA	5
GJA1/KCNH2/CAV1/PRKACA	4
NOS3/RB1	2
RB1/CCNB1	2
IL1B/IL1A	2
AR/MAPK1	2
SERPINE1/PRKACA	2
ICAM1/PPARD/PRKACA	3
F2/G6PD/PRKACA	3
ACHE/CYP1B1/BCL2/CYP1A1/CDK4	5
PPARG/SERPINE1/STAT1	3
IL2RA/ICAM1	2
ODC1/NQO1	2
AHR/PDE3A	2
BCL2/IL6R	2
EGFR/ADRB2	2
AKT1/PPARA	2
TP53/ADRB2	2
RB1/CCNB1	2
XIAP/EGFR/CAV1	3
AKT1/CAV1/MDM2/CCNB1/CDK1/PRKACA	6
ACHE/CYP1B1/BCL2/CYP1A1/CDK4	5
CYP1B1/AKR1C3	2
FOS/IL4	2
SERPINE1/IL4	2
SERPINE1/PRKACA	2
AKT1/CCL2	2
PPARG/SERPINE1/STAT1	3
SOD1/CYP3A4/G6PD/AKR1C3	4
CXCL8/CCL2/CCND1	3
IL4/BCL2/IL6R	3
PPARG/SERPINE1/STAT1	3
RB1/CCNB1	2
PON1/CAV1	2
FOS/RB1	2
IL4/IL6R	2

RB1/CCNB1	2
CHEK2/PCNA	2
CCL2/DPP4	2
GJA1/KCNH2/CAV1	3
PPARG/IL2RA/IL4	3
IL4/BCL2/CDKN1A	3
IL1B/PLA2G4A/IL4/ICAM1/IL6R	5
NOS3/MAPK1/SLC6A2/CHRNA7/GJA1/CASP3/EGFR/ICAM1/CAV1/CTSD/SLC2A4/KDR/DPP4/CD14/PRKACA/HAS2	16
NOS3/MAPK1/SLC6A2/CHRNA7/GJA1/CASP3/EGFR/ICAM1/CAV1/CTSD/SLC2A4/KDR/DPP4/CD14/PRKACA/HAS2	16
PCNA/RB1/CCND1/CDKN1A/CDK4/CCNB1/CDK1	7
PCNA/RB1/CCND1/CDKN1A/CDK4/CCNB1/CDK1	7
PCNA/RB1/CCND1/CDKN1A/CDK4/CCNB1/CDK1	7
NOS3/MAPK1/CHRNA7/CAV1/PRKACA/HAS2	6
TP53/PCNA/RB1/CCND1/CDKN1A/CDK4/CCNB1/CDK1	8
F2/SERPINE1/MMP9/MMP2/F7/F3/ACHE/ICAM1/CTSD/SLPI	10
MAOA/GJA1/PTGS2/PGR/BCL2/BCL2L1/MCL1	7
MAOA/GJA1/PTGS2/PGR/BCL2/BCL2L1/MCL1	7
MAPK1/NR3C1/AKT1/XIAP/RB1/HSPB1/BIRC5/CCNB1/CDK1	9
F10/F3/CD14	3
TP53/PPARG/FOS/RB1/JUN/E2F1	6
TP53/PPARG/FOS/RB1/CCND1/JUN/AHR/CDK4/E2F1	9
MAPK1/IGF2/SERPINE1/MPO/EGFR/CTSD/HSP90AA1/SLPI	8
PLAU/MAPK1/GJA1/EGFR/HSPB1/ICAM1/CAV1/DPP4/CD4	9
PLAU/MAPK1/GJA1/EGFR/HSPB1/ICAM1/CAV1/DPP4/CD4	9
MAPK1/MMP9/CTSD/HSP90AA1/TNFAIP6	5
GJA1/EGFR/ADRB2/ERBB2/ERBB3/IL6R/DPP4/CD44	8
MAOA/GJA1/PGR/BCL2/BCL2L1/MCL1	6
NOS3/MPO/EGFR/CAV1/MDM2/HSP90AA1/DPP4	7
F2/F10/IL2RA/F3/ICAM1/SLC2A4/IL6R/CD14	8
ERBB2/BCL2/HSP90AA1	3
GJA1/EGFR/ADRB2/ERBB2/ERBB3/IL6R/DPP4/CD44	8
MAPK1/IGF2/SERPINE1/MPO/CTSD/HSP90AA1/SLPI	7
MAPK1/IGF2/SERPINE1/MPO/CTSD/HSP90AA1/SLPI	7
PTGS2/EGFR/PLA2G4A/CCND1/BCL2/BCL2L1/PTGES/CDK4	8
MAPK1/MMP9/CTSD/HSP90AA1/TNFAIP6	5
MMP9/CTSD/TNFAIP6	3
MAPK1/GJA1/EGFR/ADRB2/ERBB2/CAV1/KDR	7
GJA1/EGFR/SLC2A4	3
PTGS2/EGFR/CCND1/BCL2/BCL2L1/CDK4	6
MPO/HSP90AA1	2

DPP4/CD44	2
XIAP/BIRC5/CDK1	3
NOS3/MAPK1/CAV1	3
MAPK1/SOD1/HSPB1	3
PLAU/MMP9/CTSD/TNFAIP6	4
ESR1/AR/NR3C2/NR3C1/PPARG/ESR2/PGR/PPARA/NR1I2/PPARD/AHR	11
ESR1/AR/NR3C2/NR3C1/PPARG/ESR2/PGR/PPARA/NR1I2/PPARD/AHR	11
ESR1/AR/TP53/PPARG/FOS/PCNA/STAT1/PPARA/RB1/MYC/HSPB1/JUN/BCL2/NR1I2/PPARD/NCOA2/NCOA1	17
ESR1/NR3C2/NR3C1/ESR2/PGR/PPARA/PPARD	7
TP53/CHEK2/EGFR/STAT1/KCNH2/RB1/MDM2/ERBB3/JUN/BCL2/CDKN1A/HSP90AA1/CCNB1/PRKACA	14
ESR1/PPARG/FOS/PCNA/STAT1/PPARA/RB1/HSPB1/JUN/NR1I2/PPARD/NCOA2/NCOA1	13
ESR1/AR/PGR/PPARA/PPARD/AHR	6
PLAU/F2/MMP9/MMP2/F7/F10/F3/ACHE/MMP1/PLAT/DPP4	11
PLAU/F2/MMP9/MMP2/F7/F10/F3/MMP1/PLAT/DPP4	10
TP53/CHEK2/EGFR/KCNH2/RB1/MDM2/ERBB3/JUN/BCL2/CDKN1A/HSP90AA1/PRKACA	12
PLAU/F2/MMP9/MMP2/F7/F10/F3/MMP1/PLAT/DPP4	10
NOS3/MPO/PTGS2/CYP3A4/NOS2/CYP1B1/PTGS1/CYP2B6/CYP1A1	9
ESR1/AR/NR3C2/NR3C1/ESR2/CYP3A4/PGR/CAV1	8
NOS3/MPO/PTGS2/CYP3A4/NOS2/CYP1B1/PTGS1/CYP2B6/CYP1A1	9
NOS3/PTGS2/CYP3A4/NOS2/CYP1B1/PTGS1/CYP2B6/CYP1A1/AKR1C3	9
NOS3/CYP3A4/NOS2/CYP2B6/CYP1A1/AKR1C3	6
PLAU/F2/MMP9/MMP2/CASP3/F7/F10/F3/MMP1/PLAT/CTSD/DPP4/CASP7	13
ESR1/AR/PGR/PPARA/PPARD/AHR	6
TP53/GJA1/RB1/MDM2/HSP90AA1	5
TP53/MAPK1/AKT1/PPARG/SOD1/EGFR/PPARA/ERBB2/BCL2	9
NOS3/CYP3A4/NOS2/CYP1B1/CYP2B6/CYP1A1/AKR1C3	7
PPARG/STAT1/PPARA/MYC/BCL2/PPARD	6
TP53/NR3C1/FOS/STAT1/MYC	5
ESR1/AR/TP53/AHR	4
ESR1/AR/TP53/NR3C1/FOS/PGR/PPARA/MYC/MDM2/JUN/NR1I2/E2F1	12

SOD1/MPO/PTGS2/PTGS1/NQO1/PTGES	6
CASP3/CCND1/CDKN1A/CDK4/CCNB1	5
IGF2/CASP3/HSPB1/CCND1/ERBB3/CDKN1A/CDK4/CCNB1	8
IGFBP3/EGFR/IL2RA/ERBB2/ERBB3/KDR/IL6R	7
ESR1/PPARG/PCNA/STAT1/NR1I2/NCOA2/NCOA1	7
ESR1/PPARG/PCNA/NR1I2/NCOA2/NCOA1	6
TP53/AKT1/PPARG/SOD1/EGFR/ERBB2/BCL2	7
BCL2/BCL2L1/MCL1	3
BCL2/BCL2L1/MCL1	3
IGF2/CASP3/HSPB1/CCND1/ERBB3/CDKN1A/CDK4/CCNB1	8
ESR1/AR/TP53/NR3C1/FOS/PGR/PPARA/MYC/MDM2/JUN /NR1I2	11
MAPK1/CDK4/CDK1	3
CYP3A4/CYP1B1/CYP2B6/CYP1A1	4
ESR1/PPARG/PCNA/STAT1/NR1I2/NCOA2/NCOA1	7
CYP3A4/CYP1B1/CYP2B6/CYP1A1	4
PPARG/FOS/RB1/MYC/JUN	5
ESR1/PPARG/PCNA/NCOA1	4
NR3C1/KDR/CYP1A1/AHR	4
TP53/SERPINE1/CASP3/F3/BCL2/DPP4	6
ESR1/AR/EGFR/PGR/CAV1	5
ESR1/AR/TP53/AHR	4
IL1B/CXCL8/CCL2/CASP3/STAT1/IL1A/IL4/IL6R	8
MPO/PTGS2/PTGS1/PTGES	4
MPO/PTGS2/PTGS1/PTGES	4
ESR1/AR/PGR	3
CYP3A4/CYP1B1/CYP1A1	3
EGFR/ERBB2/ERBB3/KDR	4
TP53/ERBB2/CAV1/ERBB3/BCL2/BCL2L1/AHR/MCL1	8
TP53/PCNA/STAT1	3
ESR1/AR/TP53/AHR	4
ESR1/AR/TP53/AHR	4
CDKN1A/CDK4/CDK1	3
TP53/AKT1/BCL2	3
CASP3/HSPB1/CDKN1A	3
EGFR/ICAM1/DPP4/CDK1	4

ESR1/PPARG/PCNA/NCOA1	4
EGFR/ICAM1/DPP4/CDK1	4
PPARG/PPARA/PPARD	3
EGFR/ERBB2/ERBB3/KDR	4
IL1B/IGF2/EGFR/ICAM1/KDR	5
IGF2/ERBB3/CDKN1A/CCNB1	4
CYP3A4/CYP1B1/CYP1A1	3
TP53/AHR	2
CHRNA7/PPARG/PLA2G4A/ACHE/GSTM1/ADRB2/CD14/PTGES	8
IGF2/ERBB3/CDKN1A/CCNB1	4
CHRNA7/PPARG/ACHE/GSTM1/ADRB2/CD14/PTGES	7
MAPK1/AKT1/PRKACA	3
CASP3/CDKN1A	2
CHRNA7/ACHE	2
GSTM1/PTGES	2
IL2RA/F3/IL6R/CD44	4
PPARG/MYC/AHR	3
PPARG/PPARD	2
GSTM1/PTGES	2
FOS/RB1/JUN	3
CASP3/CASP7	2
NOS3/NOS2/G6PD	3
NOS3/NOS2	2
CHRNA7/ACHE	2
NOS3/PPARG/NOS2/PPARD/AKR1C3	5
NOS3/PPARG/NOS2/PPARD	4
TP53/PPARG/ACHE	3
IL1B/IL1A	2
PPARG/NCOA1	2
FOS/STAT1	2
TP53/SOD1/IL1A	3
PPARA/NR1I2/PPARD	3
NR3C1/KDR/CYP1A1/AHR	4
IL1B/F2/IGF2/CXCL8/CCL2/IL1A/IL4/DPP4	8
SERPINE1/XIAP/CASP3/HSPB1/CDKN1A/SLPI/BIRC5	7
CYP2B6/CYP1A1	2
PCNA/HSP90AA1	2
IL1B/F2/IGF2/CXCL8/CCL2/IL1A/IL4/DPP4	8
MMP9/ACHE/CD44	3
TNFAIP6/CD44	2
XIAP/BIRC5	2

FOS/JUN	2
CASP3/HSPB1/CDKN1A	3
EGFR/ERBB2/ERBB3/KDR	4
IL2RA/F3/IL6R/CD44	4

NR3C1/PPARG/PPARA/MYC/JUN/PPARD	6
PPARG/PPARD/AKR1C3	3

NR3C1/PPARG/PPARA/MYC/JUN/PPARD	6
CASP3/HSPB1/CDKN1A	3
IL1B/IL1A/IL4/IL6R	4

CASP3/CTSD	2
CHRNA7/ADRB2/CAV1/BCL2	4
SERPINE1/XIAP/CAV1/SLPI/BIRC5	5
GSTM1/PTGES	2
ESR1/AHR	2
PPARG/NCOA1	2
CASP3/CTSD	2

MAPK1/AKT1/CHEK2/EGFR/CDK4/CDK1/PRKACA	7
CYP3A4/CYP1B1/CYP2B6/CYP1A1	4
IL1B/CXCL8/CCL2/IL1A/IL4	5

CDK4/CDK1	2

CDK4/CDK1	2
CHRNA7/ACHE/ADRB2	3
AR/NR3C1/EGFR	3
ESR1/AR/GJA1	3
MAPK1/PLAT/RB1	3
F2/CD14	2

GJA1/MCL1	2
HSPB1/HSP90AA1	2
TP53/PCNA/HSP90AA1	3
SERPINE1/XIAP/SLPI/BIRC5	4
PPARG/PPARD	2
TP53/SOD1/BIRC5	3
CAV1/BCL2	2

IGF2/CCNB1	2
SERPINE1/XIAP/SLPI/BIRC5	4

XIAP/BIRC5	2
TP53/MDM2/NCOA1	3
MMP9/MMP2/MMP1	3
SERPINE1/XIAP/SLPI/BIRC5	4
NOS3/AKT1/EGFR/NOS2	4
TP53/CCND1/HSP90AA1	3
